# Sulfonium cations as versatile strongly π-acidic ligands†

**DOI:** 10.1039/d2sc00588c

**Published:** 2022-03-14

**Authors:** Ruiping Li, Nitsan Barel, Vasudevan Subramaniyan, Orit Cohen, Françoise Tibika, Yuri Tulchinsky

**Affiliations:** Institute of Chemistry, The Hebrew University of Jerusalem Jerusalem 9190401 Israel yuri.tulchinsky@mail.huji.ac.il

## Abstract

More than a century old, sulfonium cations are still intriguing species in the landscape of organic chemistry. On one hand they have found broad applications in organic synthesis and materials science, but on the other hand, while isoelectronic to the ubiquitous tertiary phosphine ligands, their own coordination chemistry has been neglected for the last three decades. Here we report the synthesis and full characterization of the first Rh(i) and Pt(ii) complexes of sulfonium. Moreover, for the first time, coordination of an aromatic sulfonium has been established. A thorough computational analysis of the exceptionally short S–Rh bonds obtained attests to the strongly π-accepting nature of sulfonium cations and places them among the best π-acceptor ligands available today. Our calculations also show that embedding within a pincer framework enhances their π-acidity even further. Therefore, in addition to the stability and modularity that these frameworks offer, our pincer complexes might open the way for sulfonium cations to become powerful tools in π-acid catalysis.

## Abstract 2

Back to the scene: while isolobal to the ubiquitous tertiary phosphines, sulfonium cations as ligands were neglected for decades. This work revives the coordination chemistry of these species showing their potential as ligands for π-acid catalysis.

## Graphical abstract

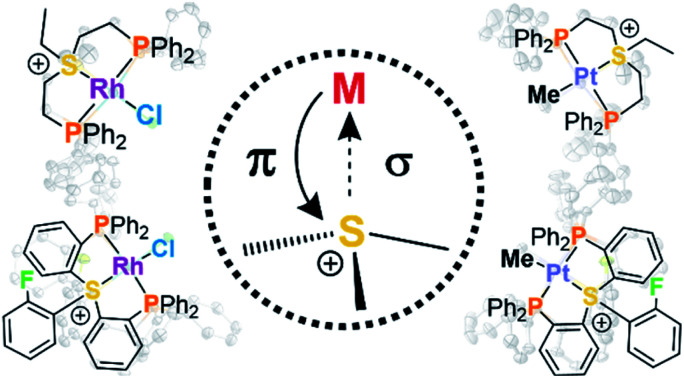

## Introduction

Rethinking the coordination chemistry of main group elements has often led to breakthroughs in metal-based homogeneous catalysis. For instance, extending the chemistry of B, Al, Ga, Sn, and Bi gave birth to the concept of σ-acceptor (aka Z-type) ligands.^1^ Peters,^2^ Lu^3^ and others^4^ have used complexes of these ligands for such fundamentally important processes as N_2_ fixation, CO_2_ reduction, and H_2_ activation.

The electron-withdrawing nature of Z-type ligands also offered new opportunities for π-acid catalysis, as demonstrated by Inagaki with borane-based pincer ligands,^5^ and Gabbai with ligands based on antimony,^6^ and carbenium cations.^7^ On the other hand, a significant advance in π-acid catalysis was achieved by Alcarazo by stretching the π-acceptor properties of phosphine^8^ and arsine^9^ to the extreme through the introduction of positively charged substituents.

While seeking to unravel new facets of main group chemistry, the coordination properties of another main-group species, sulfonium cations, have been greatly overlooked. Yet, sulfonium salts are at the forefront of fundamental and applied research due to their countless applications as precursors for sulfur ylides,^10^ alkyl and aryl group sources in cross-coupling reactions,^11^ photoacids,^12^ and many others.^13^

**Chart 1:**
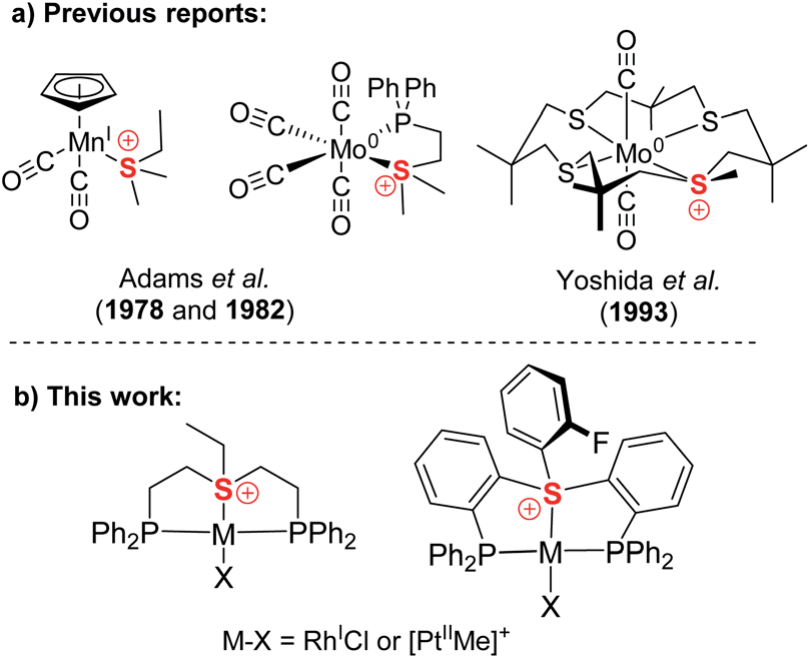
Previously reported sulfonium complexes (a) compared to the pincer type sulfonium complexes presented in the work (b).

Compared to isoelectronic and isostructural tertiary phosphines, sulfonium cations have their lone pair stabilized by their positive charge, while their low-lying S–C σ*-orbitals become available for accepting electron density. Therefore, together with sulfoxonium, they have attracted attention as non-metal Lewis acids^14^ and have been utilized as such for catalysis and anion sensing.^15^ However, while tertiary phosphines are perhaps the most iconic family of ligands, only three crystallographically characterized sulfonium complexes of Mo(0) and Mn(i) were reported decades ago (Chart 1a), where these ligands exhibited strongly π-acidic character.^16^ Yet, no sulfonium complexes relevant to catalysis have ever been reported, even though formation of transient metal-coordinated sulfonium intermediates during Pd catalyzed cross-coupling reactions of sulfonium salts has been suggested.^11*a*^

Here we report the first synthesis and characterization of a series of complexes of both aliphatic and aromatic sulfonium cations with Rh(i) and Pt(ii), two representatives of the Pt metal group,^17^ which lies at the core of today's homogeneous catalysis (Chart 1b). Our in-depth theoretical analysis of sulfonium–metal interaction demonstrated it to be dominated by π-back bonding. This strongly π-acidic character is further enhanced by the pincer frameworks, which also provide our complexes with structural robustness and modularity, both properties of pivotal importance in catalysis.^18^

## Results and discussion

### Ligand design and synthesis

**Chart 2:**
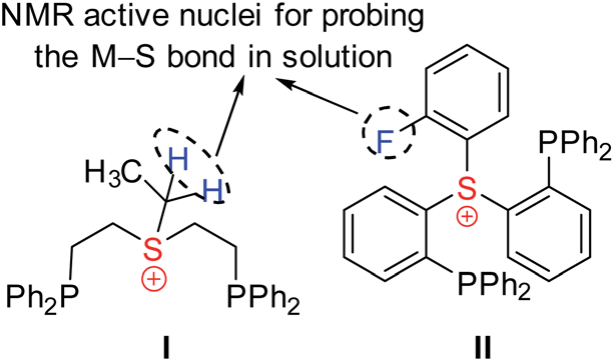
Design of sulfonium-based pincer ligands.

Obviously, coordination of the sulfonium cation is hindered by an electrostatic repulsion between its positive charge and that of a metal center (even if partial). So far, the preparation of sulfonium complexes has been achieved by alkylation of the corresponding sulfide complexes. We adopted here a more systematic approach, where the aliphatic or aromatic sulfonium moieties were incorporated within pincer frameworks (I and II, respectively in Chart 2), bearing chelating phosphine arms. A similar strategy was used earlier by Gandelman to achieve coordination of the nitrenium cation.^19^

We designed aliphatic and aromatic sulfonium ligands with NMR active nuclei in the vicinity of sulfur, namely methylene protons in I and a fluorine atom in II (Chart 2), that would allow detecting the formation of an S–M bond in solution, by tracing their chemical shifts and magnetic coupling to NMR-active metal centers, ^103^Rh and ^195^Pt.

**Scheme 1:**
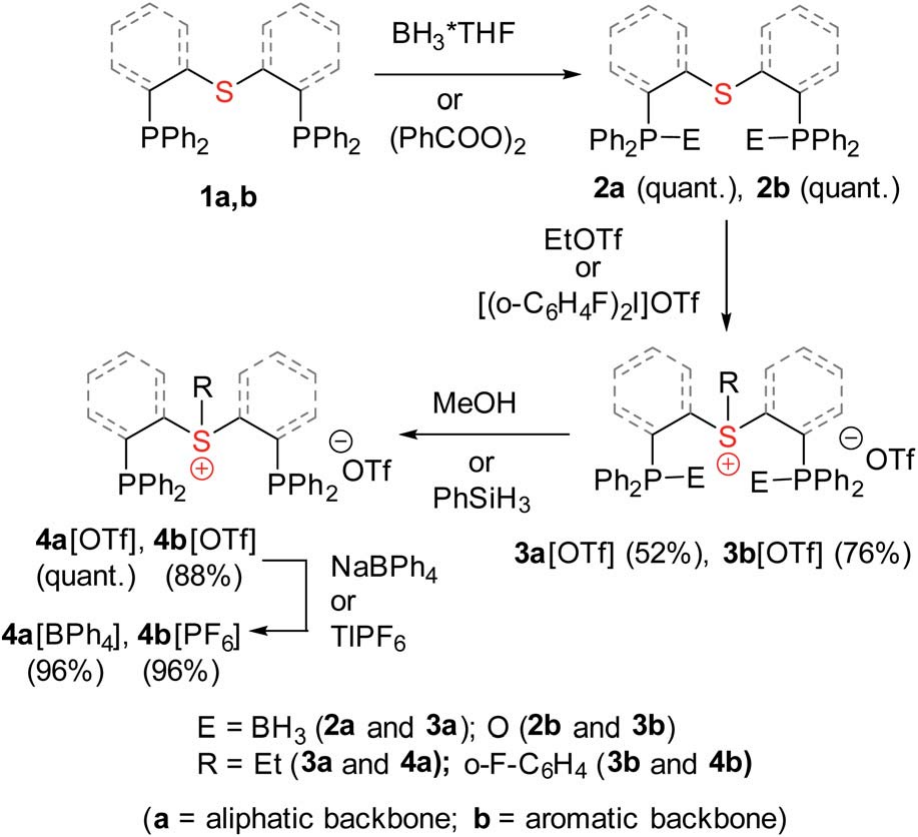
Synthesis of aliphatic and aromatic sulfonium pincer ligands.

**Fig. 1:**
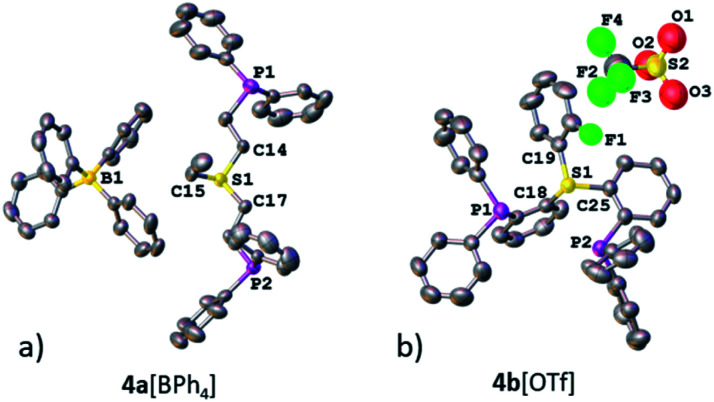
XRD structures of ligands 4a[BPh_4_] (a) and 4b[OTf] (b). Co-crystallized solvent molecules and hydrogen atoms are omitted for clarity.

Both sulfonium pincer ligands were prepared by alkylation or arylation of the corresponding bis-phosphine sulfide ligands^20^ with the phosphines protected as borane adducts or phosphine oxides in aliphatic and aromatic systems, respectively (Scheme 1), resulting after deprotection in ligands 4a[OTf] and 4b[OTf]. To obtain XRD structures of sulfonium ligands (Fig. 1) or their complexes (Fig. 3 and 4, *vide infra*) the triflate counterions were in some cases exchanged for tetraphenylborate or hexafluorophosphate.

### Synthesis and characterization of the Rh(i)–sulfonium complexes

**Scheme 2:**
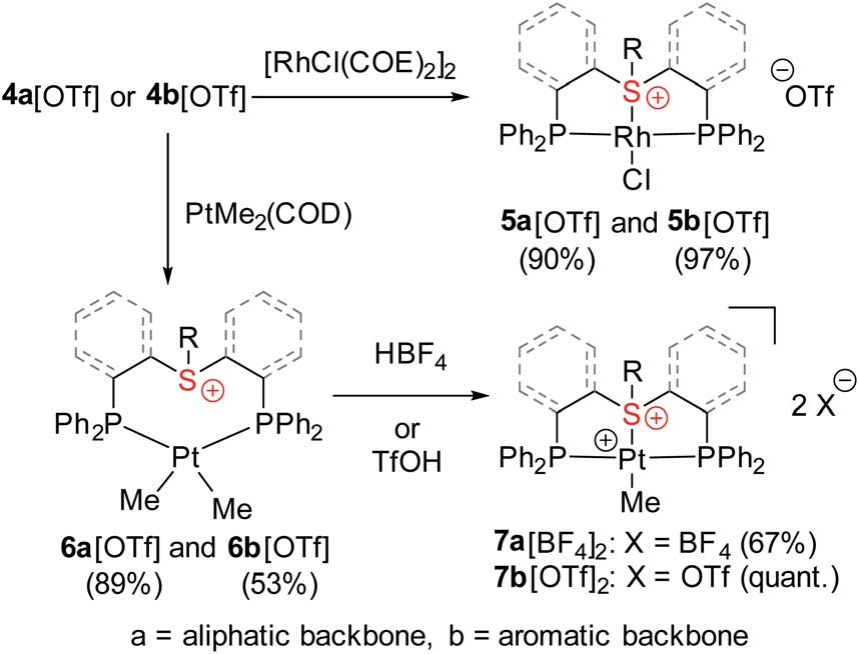
Synthesis of the Rh(i) and Pt(ii)–sulfonium complexes.

The coordinative behavior of the aliphatic sulfonium ligand 4a[OTf] towards Rh(i) was tested by reacting it with [RhCl(COE)_2_]_2_ (Scheme 2). A full conversion to a symmetric Rh(i) complex was evident by ^31^P NMR, as the chemical shift moved from a singlet at −18.2 ppm to a doublet at +46.6 ppm (^1^*J*_Rh–P_ = 127.8 Hz).

**Fig. 2:**
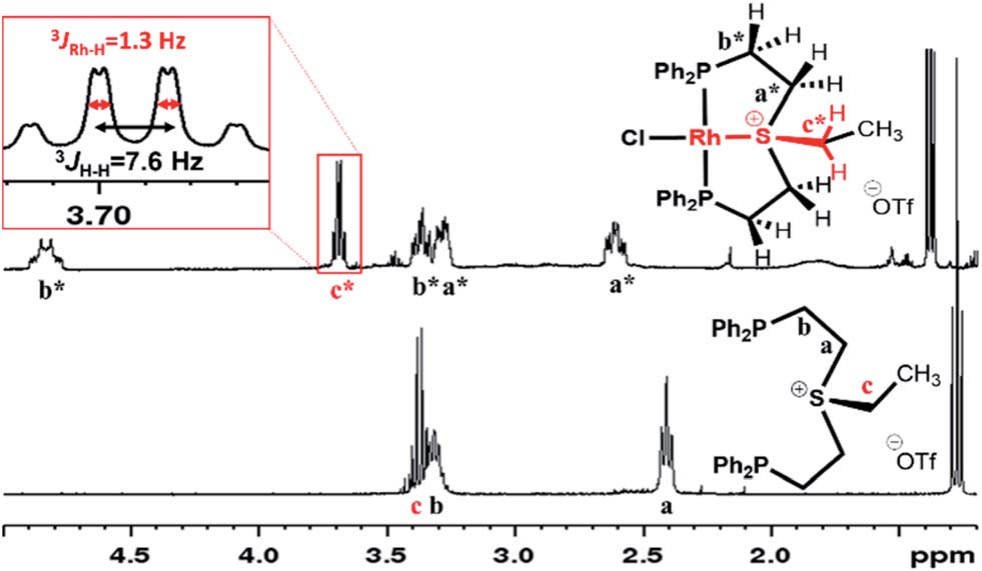
Aliphatic region of ^1^H NMR spectra of 4a[OTf] and 5a[OTf] in CD_2_Cl_2_.

In the ^1^H NMR spectrum, significant downfield shifts of all aliphatic signals are observed (Fig. 2). Each of the methylene protons signals **a** and **b** divides upon coordination into two (a* and b* pairs, respectively), indicating the formation of a rigid structure with no rotation around C–C bonds. Furthermore, an additional splitting of 1.3 Hz appears in the quartet assigned to the ethyl tail methylene protons (c*). By means of ^1^H–^103^Rh HMBC (Fig. S3†), this splitting has been attributed to a through-bond ^3^*J*_Rh–H_ interaction. The latter is only possible if sulfonium is coordinated to the Rh center.

Encouraged by these results, we then turned to the aromatic ligand 4b[OTf] (Scheme 2). Here also, a full conversion of the ligand to a symmetric Rh(i) complex 5b[OTf] was evident from the ^31^P NMR spectrum, where the chemical shift changed from a singlet at −13.0 ppm to a doublet of doublets at +48.7 ppm (^1^*J*_Rh–P_ = 126.0 Hz; ^5^*J*_F–P_ = 6.0 Hz). Interestingly, the ^31^P–^19^F interaction unobservable in the spectrum of the free ligand became noticeable after coordination, perhaps due to the additional rigidity of the formed complex.

The ^19^F NMR spectrum of 5b[OTf] showed only a small downfield shift compared to the free ligand (−104.1 *vs.* −105.3 ppm, respectively) and no additional splitting by ^103^Rh could be identified. Likewise, no ^19^F–^103^Rh interactions could be detected by HMBC, hence in this case, metal coordination to the aromatic sulfonium moiety could not be validated by NMR alone.

**Fig. 3:**
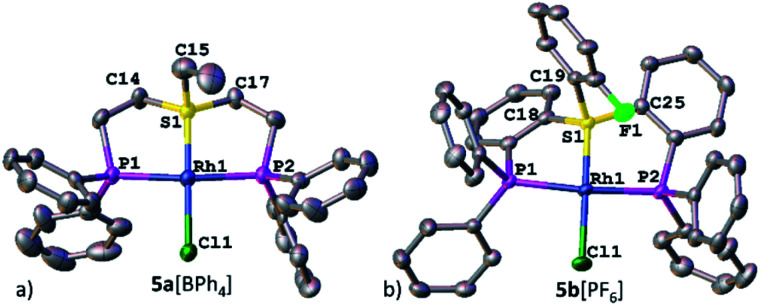
XRD structures of Rh(i)–sulfonium complexes, 5a[BPh_4_] (a) and 5b[PF_6_] (b). Co-crystallized solvent molecules, counter anions, and hydrogen atoms are omitted for clarity.

Nevertheless, the irrefutable evidence of sulfonium–Rh bonding in both systems was provided by XRD. Both complexes 5a[BPh_4_] and 5b[PF_6_] exhibited a slightly distorted square-planar geometry around the metal (with a *τ* parameter of 0.1, Table 1), typical of *d*^8^ complexes (Fig. 3a and b, respectively). Notably, the sulfonium–Rh(i) bond lengths of 2.126(2) and 2.112(1) Å observed in 5a[BPh_4_] and 5b[PF_6_], respectively, are among the shortest reported S–Rh bonds (Table 1). These are significantly shorter than in Rh(i) complexes with sulfides (>2.24 Å) and even with sulfoxides (typically, 2.159–2.291 Å).^21^ In fact, shorter Rh(i)–S bonds (2.069–2.100 Å) were only observed with the strongest π-acceptor ligands: SO_2_ ^22^ and the related *N*-sulfinylaniline.^23^ These exceptionally short S–Rh bonds in 5a[BPh_4_] and 5b[PF_6_] cannot be explained solely by the grip of the pincer framework. Indeed, in both the analogous aliphatic sulfoxide pincer complex 8 that we prepared for comparison (Fig. S17†) and the reported aromatic ones,^24^ the Rh–S bonds are still longer than in their sulfonium counterparts (2.135 and 2.134 Å, respectively).

**Table:** Selected bond lengths and angles and geometry indices of complexes

	Complex	S–M bond length (Å)	Rh–Cl or Pt–Me bond length (Å)	Average M–P bond length (Å)	S–M–X and P–M–P angles (°)	Geometry index (*τ*_4_)
Rh–Cl	5a[BPh_4_]	2.126(2)	2.340(2)	2.296(2)	179.44(9), 164.80(9)	0.11
	5b[PF_6_]	2.112(1)	2.324(1)	2.295(1)	178.8(1), 166.0(1)	0.10
	8	2.135(1)	2.369(1)	2.313(1)	172.4(1), 161.2(1)	0.18
Pt–Me	7a[BF_4_]_2_	2.258(1)	2.073(5)	2.304(1)	177.8(2), 167.3(1)	0.10
	7b[NTf_2_]_2_	2.261(1)	2.060(4)	2.292(1)	178.7(2), 165.0(1)	0.11
	9[BF_4_]	2.336(2)	2.087(7)	2.278(2)	177.6(3), 168.8(1)	0.10

Undoubtedly, these structures not only broaden the very limited pool of known sulfonium complexes but also proved for the first time the coordinating ability of an aromatic sulfonium cation. It is noteworthy, that unlike the α-cationic sulfides, which undergo oxidative addition with electron rich metals,^25^ the sulfonium complexes 5a[OTf] and 5b[OTf] remained stable as solids and in solutions.

### Synthesis and characterization of the Pt(ii)–sulfonium complexes

Having shown that stable complexes of sulfonium cations with the neutral RhCl fragment can be obtained, we wondered whether, similarly to cationic nitrenium^19*b*^ and arenium^26^ pincer ligands, our frameworks could also induce bonding between these cations and a net positively charged metal fragment, such as [PtMe]^+^. To achieve that, we first treated ligands 4a[OTf] and 4b[OTf] with Pt(COD)Me_2_ which resulted in coordination products (Scheme 2), as evident from their ^31^P NMR spectrum that exhibited downfield shifted peaks at 11.3 or 16.6 ppm with the characteristic ^195^Pt satellites (^1^*J*_Pt–P_ = 1813 and 1781 Hz, respectively). The ^1^H NMR signals at 0.42 and 0.65 ppm were assigned to the methyl protons, confirming the formation of PtMe_2_ complexes 6a[OTf] and 6b[OTf], respectively. Moreover, these signals appeared as doublets of doublets due to splitting by two magnetically inequivalent P atoms, a configuration only possible when methyl groups are oriented *cis* to each other (Fig. S1 and S2†). The neutral PtMe_2_ fragment in 6a[OTf] and 6b[OTf] was then transformed into a cation by protonolysis (by HBF_4_*OEt_2_ or HOTf) resulting in the clean formation of complexes 7a[BF_4_]_2_ and 7b[OTf]_2_ (Scheme 2), as attested by new peaks at 42.4 (^1^*J*_Pt–P_ = 2736 Hz) and 44.3 (^1^*J*_Pt–P_ = 2768 Hz) ppm, respectively, in ^31^P NMR. In the aromatic complex 7b[OTf]_2_, the ^31^P NMR signals were much sharper than in 6b[OTf], and similarly to the Rh(i) complex 5b[OTf], splitting due to the ^31^P–^19^F coupling (^5^*J*_P–F_ = 3.3 Hz) became observable.

Unlike complexes 6a[OTf] and 6b[OTf], in both 7a[BF_4_]_2_ and 7b[OTf]_2_, the ^1^H NMR signals at 1.20 and 1.56 ppm, corresponding to single methyls, appeared as triplets indicating magnetic equivalence of the two phosphines, which is only possible in a mutual *trans*-orientation (Fig. S1 and S2†). Moreover, the signals of the aliphatic protons in 7a[BF_4_]_2_ followed a pattern similar to that of 5a[OTf] (Fig. 2), suggesting an analogous structure (Fig. S1†). To further study sulfonium–Pt interaction in solution we applied ^1^H–^195^Pt HMBC, once again focusing on magnetic interaction between Pt and the methylene protons of the ethyl tail (Fig. S4†). While in 6a[OTf], this coupling constant is negligible (0.2 Hz, presumably due to ^6^*J*_Pt–H_), in 7a[BF_4_]_2_ it reaches 7.7 Hz (most likely, due to ^3^*J*_Pt–H_), suggesting the presence of a S–Pt bond in 7a[BF_4_]_2_, but not in 6a[OTf]. A similar conclusion about S–Pt bonding in 6b[OTf] and 7b[OTf]_2_ could be drawn by comparing their ^19^F–^195^Pt HMBC spectra (Fig. S5†), even though both complexes exhibited nearly identical chemical shifts in ^19^F NMR (−102.3 and −102.5 ppm, respectively). The former showed no ^19^F–^195^Pt correlation, while the latter revealed a prominent cross-peak with a coupling constant of 3.3 Hz, supporting the presence of a sulfonium–Pt bond.

**Fig. 4:**
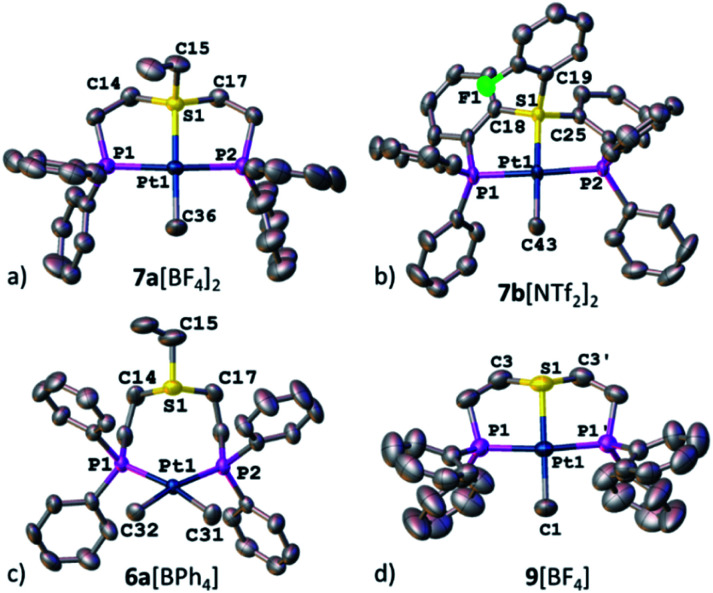
XRD structures of Pt(ii) complexes, 7a[BF_4_]_2_ (a), 7b[NTf_2_]_2_ (b), 6a[BPh_4_] (c), and 9[BF_4_] (d). Co-crystallized solvent molecules, counter anions, and hydrogen atoms are omitted for clarity.

Ultimately, the solid-state structures of 6a[BPh_4_], 7a[BF_4_]_2_, and 7b[NTf_2_]_2_ (the latter was prepared by treating 6b[OTf] with an excess of bistriflimide) were established by single crystal XRD (Fig. 4c, a and b, respectively). In 6a[BPh_4_], as expected from the NMR analysis, no Pt–S bond was observed, and the methyl groups indeed exhibited a *cis* configuration. In contrast, both 7a[BF_4_]_2_ and 7b[NTf_2_]_2_ exhibited Pt–S bonds of 2.258(1) and 2.261(1) Å, respectively (see Table 1). Surprisingly, despite electrostatic repulsion between the cationic sulfonium and the [PtMe]^+^ fragment, the Pt–S bond in 7a[BF_4_]_2_ is shorter than that in its neutral sulfide analog 9[BF_4_], 2.336(2) Å, prepared for comparison (Fig. 4d).

### Theoretical analysis of metal–sulfonium bonding and the influence of the pincer framework

The exceptionally short metal–sulfonium bonds observed in our Rh complexes prompted us to undertake a computational investigation by DFT. To gain a proper insight, we applied the energy decomposition analysis^27^ combined with the natural orbitals for chemical valence theory (EDA-NOCV) which provides a quantitative description of L–M bonding in a visual and chemically intuitive manner.^28,29^ In this method the overall interaction energy (Δ*E*_int_) between two molecular fragments (*e.g.* the sulfonium ligand and the rest of the complex) is assessed by means of EDA; then NOCV is applied to extract the total orbital interaction contribution (Δ*E*_orb_) and decompose it into individual constituents (Δ*E*_orb(*n*)_) according to their orbital symmetry. Each such constituent is then represented by a deformation density plot (Δ*ρ*_(*n*)_) that visualizes the redistribution of charge upon combination of the two molecular fragments.

**Fig. 5:**
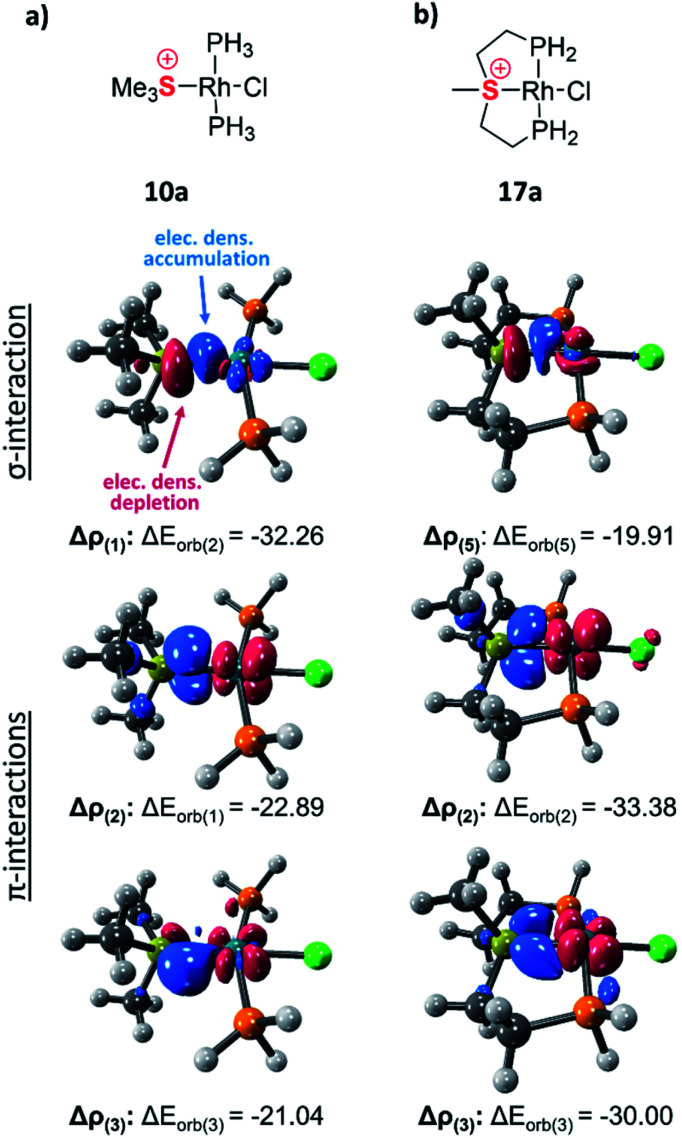
Selected deformation density plots of model complexes 10a (a) and 17a (b) (all energies are given in kcal mol^−1^).

First, we considered the Rh–S bonding interactions in the model monodentate aliphatic and aromatic sulfonium complexes 10a and 10b and compared them with analogous complexes of neutral phosphines, sulfides and sulfoxides, as well as with a few representative cationic ligands. By inspecting the deformation density plots of the most significant orbital interactions (Δ*E*_orb(*n*)_), we could identify a single σ-symmetric interaction that has a clear L → M donation character, and two π-symmetric ones (perpendicular and parallel to the coordination plane) corresponding to the M → L back-donation (see representative deformation density maps of 10a in Fig. 5a and for other maps see Tables S22 and S23†). Interestingly, in the only reported pincer complex of the isoelectronic telluronium cation the σ interaction is in an opposite direction, *i.e.*, it has a M → L character, thus classifying telluronium as a Z-type ligand.^30^ This difference in σ-bonding characteristics between sulfonium and telluronium can be rationalized by the so-called inert-pair effect,^31^ which in this case reflects the difference in energy of the 3s electrons of sulfonium compared to the 5s electrons in telluronium. In the latter the energy of this lone pair is too low to play any role in the bonding to the metal; this can only occur thanks to the donation from the metal's d orbital to the σ* orbitals of telluronium. Therefore, while isoelectronic, sulfonium and telluronium systems are not isolobal.

As evident from Table 2 in terms of their BDEs and σ-donation, sulfonium cations are nearly similar to sulfides and sulfoxides. However, sulfonium cations are significantly stronger π-acceptors, with π-back-bonding interaction being predominant. This is quite unusual and not the case even for the strongly π-acidic perfluorinated phosphines (in complexes 14a–c), where similarly to common phosphines (in 13a and 13b), σ-donation still prevails. This predominance of π-back-donation over σ-donation appears specific only to cationic ligands considered here. Compared to the latter, the π-acidity of sulfonium stands between that of N-heterocyclic nitrenium ([NHN]^+^, in 15a) and N-heterocyclic phosphenium ([NHP]^+^, in 15b), and is comparable to Alcarazo's tris-cationic phosphine PR^3+^ (in 15c).^32^

EDA-NOCV data for the monodentate [L(PH_3_)_2_MX]^*n*+^ complexes

**Table:** 

MX	RhCl							
Ligand types	Sulfonium cations		Sulfides		Sulfoxides		Phosphines	
Model complexes	10a	10b	11a	11b	12a	12b	13a	13b
L	SMe_3_^+^	SPh_3_^+^	SMe_2_	SPh_2_	DMSO	Ph_2_SO	PMe_3_	PPh_3_
BDE	−38.57	−36.96	−36.21	−38.19	−39.80	−44.55	−70.06	−57.77
σ-Bonding (% of σ/Δ*E*_orb_)	−32.26 (37.3%)	−31.13 (31.0%)	−35.03 (63.5%)	−34.39 (61.0%)	−39.82 (57.1%)	−40.73 (53.5%)	−56.69 (67.3%)	−53.31 (63.3%)
π-Backbonding^a^ (% of π/Δ*E*_orb_)	−43.93 (50.8%)	−39.40 (39.2%)	−14.89 (27.0%)	−16.10 (28.6%)	−24.42 (35.0%)	−27.55 (38.0%)	−22.06 (26.2%)	−24.19 (28.7%)

a All energies are given in kcal mol^−1^.

b Sum of the ⊥ and ‖ π-interactions.

**Table:** 

MX	RhCl						PtMe^+^	
Ligand type	Perfluorinated phosphines			Cationic ligands			Sulfonium cations	
Model complex	14a	14b	14c	15a	15b	15c	16a	16b
L	PF_3_	P(CF_3_)_3_	P(C_6_F_5_)_3_	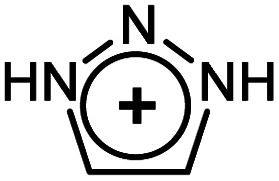 [NHN]^+^	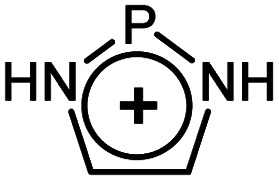 [NHP]^+^	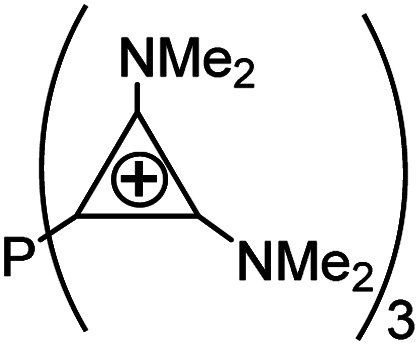 [PR_3_]^3+^	SMe_3_^+^	SPh_3_^+^
								
BDE^a^	−56.76	−53.16	−51.35	−30.55	−57.57	−53.16	+48.60	+30.66
σ-Bonding^a^ (% of σ/Δ*E*_orb_)	−51.83 (51.4%)	−46.89 (49.7%)	−47.79 (55.1%)	−25.30 (40.3%)	−42.93 (44.3%)	−44.43 (42.6%)	−33.74 (57.3%)	−32.84 (56.9%)
π-Backbonding^a^^,^^b^ (% of π/Δ*E*_orb_)	−44.61 (43.3%)	−40.75 (43.2%)	−32.21 (37.1%)	−29.19 (46.4%)	−62.42 (52.3%)	−46.17 (44.2%)	−17.20 (29.2%)	−13.53 (23.5%)

With the cationic [PtMe]^+^ fragment the calculations confirmed that the monodentate sulfonium complexes 16a and 16b (Table 2) are kinetically stable, despite the electrostatic repulsion between the positively charged metal fragment and the sulfonium ligand responsible for the calculated positive BDE values. The obtained density plots of the model Pt complexes 16a and 16b were comparable in shape with those of the Rh complexes 10a and 10b (Fig. S18†), with prominent σ- and π-symmetric interactions. As expected for a positively charged metal center, the contribution of the π back-bonding in these model Pt complexes is significantly weaker than in their RhCl counterparts, yet still not negligible.

The influence of the pincer framework on bonding in both the Rh complexes 17a and 17b and their Pt analogues 18a and 18b is quite pronounced. As evident from Table 3, one can see that in both complexes the geometry deformations imposed by the pincer ligands strengthen the π back-donation within the complexes, so that the overall π/σ ratio significantly increases. Remarkably, in the case of the Pt complexes 18a and 18b π back-bonding even becomes comparable to the σ-donation, in spite of the positive charge on the metal center.

**Table:** EDA-NOCV data for the [LMX]^*n*+^ sulfonium pincer complexes

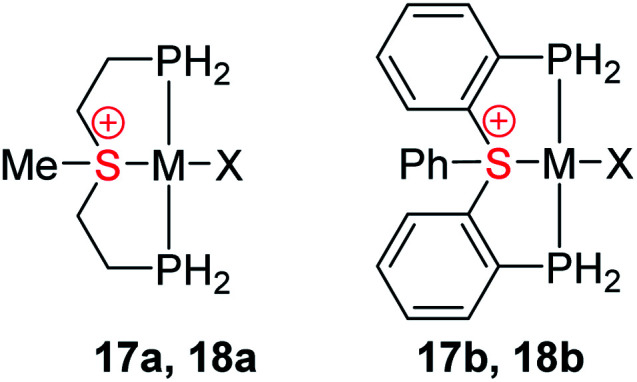				
MX	RhCl		PtMe^+^	
Model complex	17a	17b	18a	18b
σ-Bonding^a^	−19.91	−18.17	−47.16	−45.02
π-Back bonding^a^^,^^b^	−63.38	−59.10	−39.72	−41.57

a All energies are given in kcal mol^−1^.

b Sum of the ⊥ and ‖ π-interactions.

**Fig. 6:**
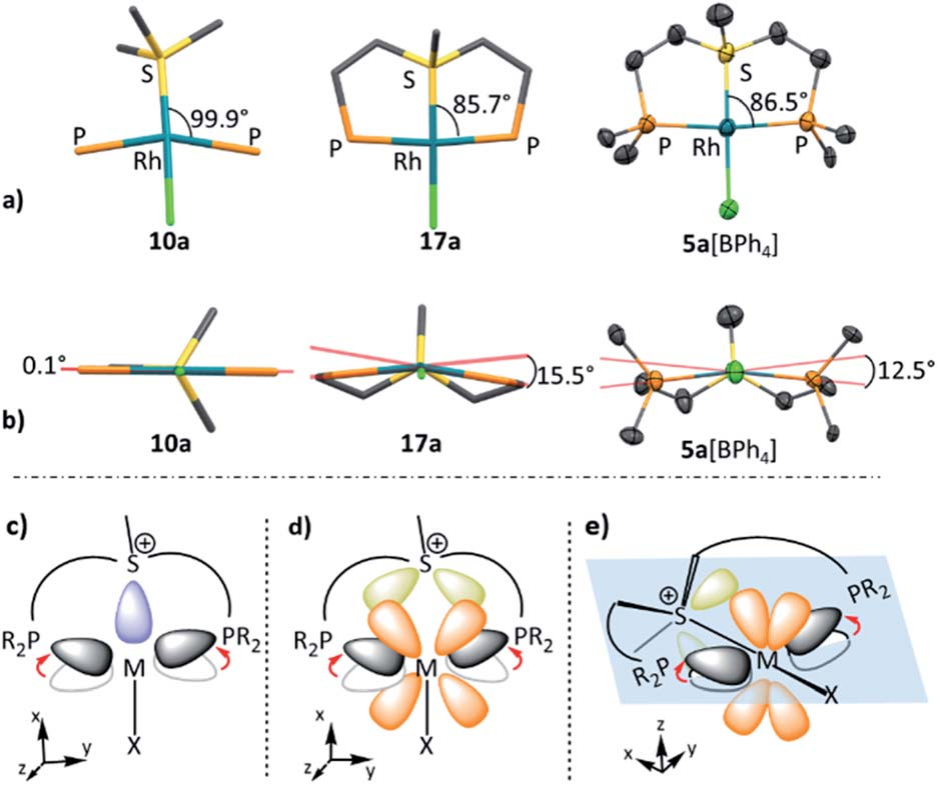
The in-plane (a) and out-of-plane (b) deformations in model complexes 10a, 17a and 5a[BPh_4_] (hydrogen atoms, BPh_4_ counter anion, and phenyl rings are omitted for clarity); schematic representation effect of pincer framework induced the in-plane and out-of-plane deformations on the σ (c) and π-interactions (d and e).

These changes in bonding character can be rationalized by comparing the geometries of the pincer complexes relative to the monodentate ones. The following discussion of the aliphatic and aromatic Rh complexes, as displayed in Fig. 6a, b and S19,† respectively, is also applicable to the Pt systems.

In the aliphatic sulfonium pincer systems (both 5a[BPh_4_] and its model analog 17a), the average P–Rh–S angles are ∼15° smaller than in the optimized monodentate complex 10a (Fig. 6a). Such a decrease essentially pushes the phosphine lone pairs closer to those of sulfonium, increasing repulsive interactions between them. Thus, the sulfonium lone-pair is pushed away from the metal, which results in weakening the σ-donation in pincer complexes (Table 4, column 2). At the same time, this angle reduction also causes a stronger repulsion between the lone pairs of the phosphines and the filled d_*xy*_ orbital of the metal, shifting electron density closer to the adjacent σ*-orbital of the sulfonium (Fig. 6b). An enhanced in-plane π-back-donation is thus induced (Table 4, column 3).

**Table:** Comparison of the σ and π interaction energies in model complexes 10a and 10b, 17a and 17b, 16a and 16b, and 18a and 18b^a^

Model complex		Δ*E*_orb_ of L → M	Δ*E*_orb_ of ‖ M → L	Δ*E*_orb_ of ⊥ M → L
		σ-Donation^b^	π-Backdonation^b^	π-Backdonation^b^
Rh–Cl	10a	−32.26	−21.04	−22.89
	10b	−31.13	−18.10	−21.30
	17a	−19.91	−30.00	−33.38
	17b	−18.17	−30.02	−29.08
				
Pt–Me	16a	−33.74	−8.62	−8.58
	16b	−32.84	−6.42	−7.11
	18a	−47.16	−17.55	−22.17
	18b	−45.02	−22.70	−18.87

a For the corresponding deformation density plots, see Tables S22 and S23.

b All energies are given in kcal mol^−1^.

In addition, the pincer framework also distorts the otherwise nearly planar coordination environment around the metal, pushing the two phosphines out of the coordination plane (Fig. 6b). This in turn results in repulsive interactions with the filled d_*xz*_ orbital, similarly strengthening the interaction with the perpendicular σ*-orbital of the sulfonium (Fig. 6c). Therefore, π-back-donation in the perpendicular plane increases as well (Table 4, column 4).

Overall, the EDA-NOCV data clearly points out that geometric distortion imposed by the pincer framework not only preserves the unique characteristics of sulfonium cations as weak σ-donors and potent π-acceptors, but also enhances them. For comparison, an analogous attempt to incorporate a phosphenium moiety within a pincer framework resulted in a full charge transfer from the metal to the ligand, transforming it into a phosphide.^33^

## Conclusions

To summarize, in this paper we have consolidated the status of sulfonium cations among the family of rare cationic ligands demonstrating for the first time that their coordination chemistry can be extended to the Pt group metals. We also prepared the very first examples of metal-coordinated aromatic sulfonium cations. These unusual compounds might represent stable analogs of possible transient intermediates forming during Pd-catalyzed cross-coupling of sulfonium salts. Our calculations suggested that sulfonium cations are among the best π-acceptors available. Moreover, the pincer frameworks which offer additional robustness also intensify this propensity. These scaffolds might therefore be the key to transform sulfonium complexes from a chemical curiosity into potential π-acid catalysts, the applications of which are currently studied in our lab.

## Data availability

Experimental procedures, NMR spectra and computational details are given in ESI.†

## Author contributions

Y. T. conceived the project, R. L., N. B., and O. C. performed the experiments, V. S. performed the XRD data refinement and DFT calculations, and V. S., Y. T., and F. T. wrote the paper.

## Conflicts of interest

There are no conflicts of interest.

## Supplementary Material

SC-013-D2SC00588C-s001

SC-013-D2SC00588C-s002
