# Supplementary material for: Sulfonium cations as versatile strongly π-acidic ligands
Source: Chem Sci. 2022 Mar 14;13(17):4770–8. doi: 10.1039/d2sc00588c (PMC9067576; doi:10.1039/d2sc00588c)
Supplement: SC-013-D2SC00588C-s001 [file SC-013-D2SC00588C-s001.pdf]

Electronic Supplementary Material (ESI) for Chemical Science.
This journal is © The Royal Society of Chemistry 2022

# Supporting Information

## Sulfonium cations as versatile strongly π-acidic ligands

Ruiping Li, Nitsan Barel, Vasudevan Subramaniyan, Orit Cohen, Francoise Tibika, and Yuri Tulchinsky*

Institute of Chemistry, The Hebrew University of Jerusalem, Jerusalem, 9190401, Israel.

e-mail : yuri.tulchinsky@mail.huji.ac.il

# Table of Contents


Page No.

**1. Synthetic procedures and characterization** **3**

1.1. General information 3

1.2. Synthetic procedures for the preparation of sulfonium ligands 3

1.3. Synthetic procedures for the preparation of Rh(I) and Pt(II) sulfonium complexes 6

1.4. $^{1}$H NMR spectra comparison 10

1.5. Selected 2D NMR spectra of the complexes 11

**2. Crystallography** **20**

2.1. General crystallographic and refinement details 20

2.2. XRD structure of complex **8** 20

2.3. Crystallographic parameter tables 21

**3. Computational details** **23**

3.1. Geometry optimizations 23

3.2. General details of ALMO-EDA calculations 23

3.3. ALMO-EDA analysis of model pincer complexes **10a-16b** 23

3.4. ETS-NOCV analysis results from ALMO-EDA calculations for model complexes **10a-18b** 24

3.5. Selected deformation density plots of model complexes **10a-16b** 34

3.6. Selected deformation density plots of model complexes **17a-18b** 36

3.7. Coordinates of the optimized model complexes **10a-18b** 39

**4. NMR spectra** **50**

**5. References** **90**

# 1. Synthetic procedures and characterization

## 1.1. General information

All air sensitive reactions were carried out under an atmosphere of purified nitrogen in a glovebox equipped with an inert gas purifier. Tetrahydrofuran (THF), acetonitrile (MeCN), diethyl ether ($E_2O$), dichloromethane (DCM), toluene, and MeOH were purified by passing through a column of activated alumina under inert atmosphere. Divinyl sulfide,[1] diphenylphosphine,[2] bis(2-bromophenyl)sulfide,[3] bis(2-fluorophenyl)iodonium trifluoromethanesulfonate,[4] bis(diphenyl)phosphanylethylene sulfoxide (PS(O)P)[5] were prepared according to the literature procedures. Pt(COD)$Me_2$ and $[Rh(COE)_2Cl]_2$ were purchased from STREM Chemicals and used without further purification. Anhydrous hexane, N,N-dimethylformamide (DMF), dichloroethane (DCE) and dimethyl sulfoxide (DMSO) packed under inert gas over molecular sieves were used as purchased. All chromatographic purifications were performed on CombiFlash EZ-Prep and $SiO_2$ columns. In particular, this allowed fast purification of phosphine-containing ligands under air without noticeable oxidation. The microwave reaction was carried out in a Monowave 200 (Anton Paar, Austria) reactor. NMR spectra were recorded on Bruker Avance 400 or Bruker Avance 500 spectrometers at 296K. Residual solvent peaks were used as internal standards for $^{1}H$ and $^{13}C$ respectively ($CDCl_3$: δ 7.26/77.0 ppm; $CD_2Cl_2$: δ 5.31/53.8 ppm, $CD_3OD$: δ 3.31/47.7 ppm, $CD_3NO_2$: δ 4.33/62.8 ppm, and $C_2D_2Cl_4$: δ 5.94/74.4 ppm). $^{31}P$ NMR and $^{31}F$ NMR signals were referenced to internal standards (capillary) of 85% $H_3PO_4$ (δ 0.00 ppm) and neat $C_6F_6$ (δ -164.9 ppm). $^{103}Rh$ and $^{195}Pt$ signals were referenced using Ξ values of 0.03186447 and 0.21496784, respectively, using the IUPAC recommendations.[6] NMR data are reported as follows: chemical shift, multiplicity (s = singlet, d = doublet, t = triplet, q = quartet, m = multiplet, br = broad, v = virtual), coupling constant(s), and integration. High resolution mass spectra (HRMS) were recorded on HR Q-TOF-MS mass instrument, using an electrospray ionization (ESI+) technique (MeCN/$H_2O$ 80%, flow: 0.2 ml/min). All reactions involved in the preparation of sulfonium complexes were performed in degassed dry solvents under the atmosphere of purified nitrogen inside a glovebox, unless specified otherwise.

## 1.2. Synthetic procedures for the preparation of sulfonium ligands

*Bis-diphenyl-phosphanylethylene sulfide bis-borane complex (**2a**)*

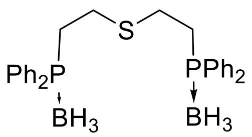


First bis-diphenyl-phosphanylethylene sulfide (**1a**) was prepared by a radical-catalyzed addition of diphenylphosphine to divinyl sulfide by an adapted literature procedure, as follows.1 Diphenylphosphine (35 mmol, 6.1 mL), divinyl sulfide (14 mmol, 1.3 mL) and AIBN (0.18 mmol, 30 mg) were added to a Schlenk tube under nitrogen atmosphere. The tube was sealed, and the reaction mixture was heated at 120°C for 1.5h, then exposed to vacuum at that temperature for another 1.5 h. The obtained crude **1a** was cooled down to room temperature and treated with 1.0 M $BH_3$ solution in THF (35 mmol, 35 ml) which resulted in precipitation of a white solid. This was collected by filtration, washed three times with ether and dried, affording **2a** in a quantitative yield.

$^{1}H$ NMR (400 MHz, $CDCl_3$) δ = 7.43-7.68 (m, 20H, P*Ph₂*) ppm, 2.62-2.67 (m, 4H, P$CH_2$), 2.39-2.45 (m, 4H, $CH_2$S), 0.61-1.35 (br, 6H, 2B$H_3$). $^{13}C$ NMR (101 MHz, $CDCl_3$) δ = 132.3 (d, $^{3}J_{CP}$ = 9.1Hz, meta-C, Ph), 131.7 (d, $^{4}J_{CP}$ = 2.3 Hz, para-C, Ph), 129.3 (d, $^{2}J_{CP}$ = 10.1 Hz, ortho-C, Ph), 128.7 ($^{1}J_{CP}$ = 32.4 Hz, ipso-C, Ph), 26.4 (d, $^{1}J_{CP}$ = 32.4 Hz, *C*H₂P), 25.0 (d, $^{2}J_{CP}$ = 2.1 Hz, *C*H₂S) ppm. $^{31}P$ NMR (162 MHz, $CDCl_3$): δ = 15.5 ppm. HRMS: calcd. for $C_{28}H_{33}B_2P_2S^{+}(M+H)^{+}$: 485.1959; found 485.1969.

*Bis-diphenyl-phosphanylethylene (ethyl) sulfonium trifluoromethanesulfonate bis-borane complex (**3a**[OTf])*

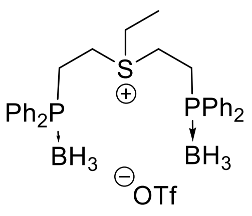


Ethyl triflate (156 µl, 1.2 mmol) and **2a** (389 mg, 0.8 mmol) were dissolved in 6 mL DCM and heated to 50 °C in a sealed Schlenk tube under nitrogen atmosphere for 2 days. All volatiles were removed under reduced pressure, forming **3a**[OTf] as a white foam. The product was purified by a silica gel column chromatography (MeOH/DCM, 3:97) resulting in 240 mg of the product (52% yield).

$^{1}H$ NMR (400 MHz, $CDCl_3$) δ = 7.44-7.81 (m, 20H, P*Ph₂*), 3.35-3.43 (m, S$CH_2$, 4H and S$CH_2$$CH_3$, 2H), 2.83-2.89 (m, 2H, P$CH_2$), 2.64-2.72 (m, 2H, P$CH_2$), 1.33 (t, S$CH_2$$CH_3$, 3H, $^{3}J_{HH}$ = 7.4Hz), 0.53-1.22 (br q, 6H, 2B$H_3$) ppm. $^{13}C$ NMR (101 MHz, $CDCl_3$) δ = 132.2-132.4 (*meta*-C and *para*-C, Ph), 129.4 (dd, $^{2}J_{CP}$ = 5.1 Hz, *ortho*-C, Ph), 126.4

(dd, $^1J_{CP}$ = 51.6 Hz, *ipso*-C, Ph), 34.0-34.2 (S$CH_2CH_3$ and S$CH_2$), 20.8 (d, $^1J_{CP}$ = 34.5 Hz, P$CH_2$), 8.3 ($SCH_2$$CH_3$) ppm. $^{31}$P NMR (162 MHz, $CDCl_3$) δ = 16.9 ppm. $^{19}$F NMR (376 MHz, $CDCl_3$) δ = -78.2 ppm. HRMS: calcd. for $C_{30}H_{39}B_2P_2S^+$ $(M)^+$: 515.2428; found 515.2431.

*Bis-diphenyl-phosphanylethylene(ethyl)sulfonium trifluoromethanesulfonate (**4a**[OTf])*

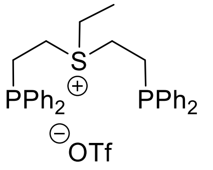


**3a**[OTf] (240 mg, 0.36 mmol) was dissolved in 5 mL of MeOH and heated to 80°C in a sealed Schlenk tube under nitrogen atmosphere for 4h. The solvent was removed, and the residue was washed with ether, forming **4a**[OTf] as a white foam. Quantitative yield.

$^1$H NMR (400 MHz, $CD_2Cl_2$) δ = 7.37-7.40 (m, 20H, P$Ph_2$), 3.28-3.40 (m, S$CH_2$$CH_3$, 2H and S$CH_2$, 4H), 2.39-2.43 (m, $CH_2$P, 4H), 1.28 (t, $SCH_2$$CH_3$, 3H, $^3J_{HH}$ = 7.6 Hz). $^{13}$C NMR (101 MHz, $CD_2Cl_2$) δ = 135.9 (d, $^3J_{CP}$ = 12.5Hz, *ipso*-C, Ph), 133.3 (d, $^4J_{CP}$ = 19.5 Hz, *meta*-C, Ph), 130.4 (d, $^2J_{CP}$ = 2.7 Hz, *para*-C, Ph), 129.6 (d, $^1J_{CP}$ = 7.0 Hz, *ortho*-C, Ph), 37.2 (d, $^2J_{CP}$ = 24.1 Hz, $CH_2$S), 34.9 (S$CH_2$$CH_3$), 23.3 (d, $^1J_{PC}$ = 19.0 Hz, P$CH_2$), 9.4 ($SCH_2$$CH_3$) ppm. $^{31}$P NMR (162 MHz, $CD_2Cl_2$) δ = -18.2 ppm. $^{19}$F NMR (376 MHz, $CD_2Cl_2$) δ = -78.2 ppm. HRMS: calcd. for $C_{30}H_{33}P_2S^+$ $(M)^+$: 487.1773; found 487.1793.

*Bis-diphenyl-phosphanylethylene(ethyl)sulfonium tetraphenylborate (**4a**[$BPh_4$])*

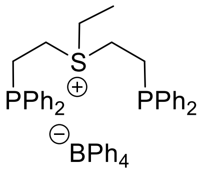


**3a**[OTf] (240 mg, 0.36 mmol) was dissolved in 5 mL of MeOH and heated to 80°C in a sealed Schlenk tube under nitrogen atmosphere for 4h. After the solution cooled down to room temperature, sodium tetraphenylborate (200 mg, 0.58 mmol) was added, forming a white precipitate immediately. The precipitate was collected by filtration through a cotton plug and consecutively washed with MeOH and ether, resulting in 280 mg (0.35 mmol, 96.4%) of **4a**[$BPh_4$]. XRD quality single crystals were grown by slow evaporation of a DCM solution of **4a**[$BPh_4$] at room temperature.

$^1$H NMR (400 MHz, $CDCl_3$) δ = 7.39-6.79 (m, 20H (B$Ph_4$) + 20H (P$Ph_2$)), 2.11-2.16 (m, 4H, $CH_2$S), 1.82-1.85 (m, 4H, $CH_2$P), 1.44-1.49 (q, S$CH_2$$CH_3$, 2H, $^3J_{HH}$ = 7.4 Hz), 0.63 (t, $SCH_2$$CH_3$, 3H, $^3J_{HH}$ = 7.4 Hz) ppm. $^{13}$C NMR (126 MHz, $CDCl_3$) δ = 164.9-163.8 (*ipso*-C, B$Ph_4$), 136.3 (B$Ph_4$), 135.0 (*ipso*-C, $PPh_2$), 132.8 (d, $^4J_{CP}$ = 18.2 Hz, *meta*-C, P*Ph*), 130.3 (d, $^2J_{CP}$ = 2.5 Hz, *para*-C, *Ph*), 129.4 (d, $^1J_{CP}$ = 6.8 Hz, *ortho*-C, Ph), 126.1 (B$Ph_4$), 122.2 (B$Ph_4$), 36.3 (d, $^2J_{PC}$ = 22.9 Hz, $CH_2$S), 33.5 (S$CH_2CH_3$), 23.0 (d, $^1J_{PC}$ = 18.3 Hz, $CH_2$P), 9.1 ($SCH_2$$CH_3$) ppm. $^{31}$P NMR (162 MHz, $CDCl_3$) δ = -18.1 ppm. $^{11}$B NMR (128 MHz, $CDCl_3$) δ = -6.5 ppm. HRMS: calcd. for $C_{30}H_{33}P_2S^+$ $(M)^+$: 487.1773; found 487.1776.

*1,1'-(Thiodi-2,1-phenylene)bis[1,1-diphenylphosphine oxide] (**2b**)*

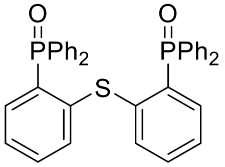


First 1,1'-(Thiodi-2,1-phenylene)bis[1,1-diphenylphosphine] (**1b**) was prepared from bis(2-bromophenyl) sulfide by adapted literature procedures,[7] as follows. In a dry 3-neck Schlenk flask under inert gas 2.5 g (7.26 mmol) of bis(2-bromophenyl) sulfide[3] was added, vacuum was applied, and the flask was purged with inert gas again. 45 ml of dry THF and 7.61 ml (50.7 mmol 7 eq.) of anhydrous TMEDA was added, and the solution was cooled to -78°C. Subsequently, 9.54 ml of n-BuLi (1.6M, 15.26 mmol, 2.1 eq.) were added dropwise which resulting in the solution turning light yellow. After stirring for additional 40 minutes 2.74 ml of $ClPPh_2$ (15.26 mmol, 2.1 eq.) were added dropwise, and the solution was allowed to stir for 30 minutes at -78°C and afterwards for additional 2 hours at RT. Completion of the reaction was established by $^{31}$P-NMR. The reaction solution was then diluted with 150 ml of degassed DCM and washed with 100 ml of degassed $H_2O$ in a 500ml separatory funnel. The organic layer was collected, dried over $Na_2SO_4$, filtered, and all volatiles were removed in vacuo resulting in a yellow-brown crude **1b**. This was then purified by flash-chromatography on silica-gel eluting with $CHCl_3$/Hexane (15:85%) mixture affording 2.12 g of pure **1b** as white foam (53% yield). $^1$H NMR (400MHz, $CDCl_3$) 7.25-7.18 (m, 12H), 7.17-7.12 (m, 8H), 7.11-7.06 (m, 2H), 7.04-6.99 (m, 4H), 6.78-6.74 (m, 2H). $^{31}$P NMR (400MHz, $CDCl_3$) -12.45ppm.

In a round bottom flask, **1b** (3.0 g, 5.41 mmol) was dissolved in 85 ml of MeCN. Benzoyl peroxide (3.28 g, 2.5 eq.) was added gradually to the solution and the reaction was stirred for 30 min. The reaction completion was established by $^{31}$P NMR. The solvent was removed in vacuo resulting in a yellow-brown oil. The crude product was then purified by automatic flash chromatography on silica-gel eluting with MeOH/DCM (12:88%) mixture to afford the product as white foam (3.18 g, quantitative yield).

$^{1}H$ NMR (500 MHz, $CDCl_3$): 7.70-7.64 (m, 2H), 7.63-7.57 (m, 8H), 7.47-7.40 (m, 4H), 7.39-7.31 (m, 8H), 7.25-7.18 (m, 2H), 6.83-6.79 (m, 2H) ppm. $^{31}P$ NMR (162 MHz, $CDCl_3$): 28.9 (s) ppm. $^{13}C$ NMR (125 MHz, $CDCl_3$): 140.93 (d, $J$ = 6.7 Hz), 134.39 (d, $J$ = 9.6 Hz), 133.83 (d, $J$ = 8.0 Hz), 132.66 (s), 132.03 (d, $J$ = 9.5 Hz), 131.6 (s), 131.3 (s), 129.8 (s), 128.2 (d, $J$ = 12 Hz), 126.8 (d, $J$ = 11.0 Hz) ppm. HRMS: calcd. for $C_{36}H_{29}O_2P_2S^+$ $(M+H)^+$: 587.1363; found 587.1510.

*Bis(2-(diphenylphosphoryl)phenyl)(2-fluorophenyl)sulfonium trifluoromethanesulfonate (**3b**[OTf])*

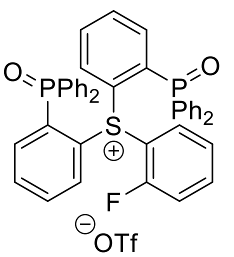


Compound **3b**[OTf] was prepared by an adapted literature procedure.[8] An oven-dried 10 ml microwave tube was loaded under an inert atmosphere with **2b** (800 mg, 1.364 mmol), bis-(2-fluorophenyl)iodonium triflate (1.59 g, 2 eq.), copper(II) benzoate (33.4 mg, 0.08 eq.) and 8 ml of degassed chlorobenzene. The tube was sealed and placed inside a Monowave 200 reactor, where it was heated with stirring to 125 °C for 2.5h. The solvent was then removed in vacuo and the residue was extracted into MeCN. The obtained MeCN solution was washed with hexane, the hexane phase was discarded, while the MeCN phase was evaporated, affording the crude product as a brown-yellow oil. This was then purified by an automatic flash chromatography on silica-gel eluting with an Acetone/$CHCl_3$ (35-55:65-45%) to afford the pure product as a beige-colored foam (0.86 g, 76% yield). Crystals suitable for XRD were grown by layer diffusion of hexane into a concentrated DCM solution.

Due to dynamic equilibrium that resulting a significant peak broadening at r.t., multinuclear NMR measurements of **3b**[OTf] were all performed at 98.5±1.00 °C.

$^{1}H$ NMR (500 MHz, $C_2D_2Cl_4$, 98.5°C): 7.84-7.76 (m, 4H), 7.68-7.60 (m, 8H), 7.59-7.50 (m, 7H), 7.50-7.39 (m, 8H), 7.34-7.29 (m, 2H), 7.29-7.20 (m, 2H), 6.98 (bt, 1H, $J$ = 7.0 Hz) ppm. $^{31}P$ NMR (202.4 MHz, $C_2D_2Cl_4$, 98.5°C): 28.6 (s) ppm. $^{13}C$ NMR (125.8 MHz, $C_2D_2Cl_4$): 160.4 (d, *Ar*F, $J$ = 257.5 Hz), 136.8 (d, $J$ = 8.2 Hz), 136.1 (d, $J$ = 10 Hz), 134.8 (s), 133.8 (d, $J$ = 10.5 Hz), 133.5 (d, $J$ = 2.7 Hz), 133.4 (d, $J$ = 2.7 Hz), 133.2-133.1 (m), 132.7 (d, $J$ = 10.5 Hz), 132.4 (d, $J$ = 10.0 Hz), 132.3 (d, $J$ = 8.0 Hz), 132.1 (d, $J$ = 10.3 Hz), 131.1 (s), 130.2 (s), 129.4 (d, $J$ = 4.4 Hz), 129.3 (d, $J$ = 4.4 Hz), 126.9 (d, $J$ = 3.3 Hz), 121.5 (q, *OTf*, $J$ = 322.5 Hz), 118.2 (d, $J$ = 19.5 Hz), 116.7 (d, $J$ = 15.0 Hz) ppm. $^{19}F$ NMR (470.6 MHz, $C_2D_2Cl_4$, 98.5°C): -76.9 (s, *OTf*, 3F), -104.4 (q, *ArF*, 1F, $J$ = 6.7 Hz) ppm. HRMS: calcd. For $C_{42}H_{32}FO_2P_2S_2^+$ $(M)^+$: 681.1571; found 681.1551.

*Bis(2-(diphenylphosphaneyl)phenyl)(2-fluorophenyl)sulfonium trifluoromethanesulfonate (**4b**[OTf])*

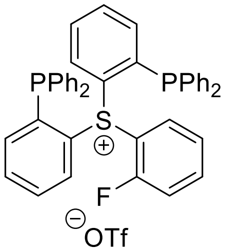


Compound **4b**[OTf] was prepared by an adapted literature procedure.[9] Compound **3b**[OTf] (1.675 g, 2.016 mmol) was charged into an oven dried Schlenk tube and suspended in 60 ml of 1:2 DCE/Toluene mixture under nitrogen atmosphere. Then 54 µL (0.3 eq.) of triflic acid were added and the reaction mixture was stirred for 10 minutes. Next, 4.00 ml (16 eq.) of $PhSiH_3$ were added, the tube was sealed and placed in a pre-heated oil bath at 85 °C. During the reaction, the suspension turned into a clear pale-yellow solution. The reaction progress was monitored by $^{31}P$ NMR which showed a complete conversion after 40 h. The solvents were evaporated, affording the crude product as a yellow oil. This was then purified by automatic flash chromatography on silica-gel eluting with Acetone/$CHCl_3$ (35-55:65-45%) affording the product as a white foam (0.86 g, 88% yield). Crystals suitable for XRD were grown by layer diffusion of hexane into a concentrated DCM solution.

$^{1}H$ NMR (500 MHz, $CDCl_3$) 7.68 (dt, 2H, $J$ = 7.7, 1.4 Hz), 7.59 (dt, 2H, $J$ = 7.5, 1.0 Hz), 7.58-7.53 (m, 1H), 7.48 (dd, 2H, $J$ = 8.3, 2.7 Hz), 7.37-7.32 (m, 3H), 7.32-7.28 (m, 6H), 7.28-7.21 (m, 8H), 7.15-7.07 (m, 8H) ppm. $^{31}P$ NMR (202MHz, $CDCl_3$): -13.0 (s) ppm. $^{13}C$ NMR (125 MHz, $CDCl_3$): 160.4 (d, ArF, J = 257.0 Hz), 141.9 (d, J = 17.5 Hz), 137.8 (d, J = 8.5 Hz), 137.3 (s), 135.0 (s), 134.1 (d, $J$ = 14 Hz), 133.9 (d, $J$ = 14 Hz), 133.0 (s), 132.9 (s), 132.8 (d, $J$ = 5.5 Hz), 132.4 (d, $J$ = 5.5 Hz) 132.1 (t, $J$ = 3.2 Hz), 130.4 (d, $J$ = 3.2 Hz), 129.4 (d, $J$ = 3.3 Hz), 129.3 (d, $J$ = 3.3 Hz), 127.6 (d, $J$ = 3.2 Hz), 121.4 (q, OTf, $J$ = 321.0 Hz), 118.3 (d, $J$ = 19.5 Hz), 111.1 (dt, $J$ = 7.6 Hz) ppm.$^{19}F$ NMR (376 MHz, $CDCl_3$): -76.5 (s, OTf), -105.3 (br s, ArF) ppm. HRMS: calcd. For $C_{42}H_{32}FP_2S^+$ $(M)^+$: 649.1678; found 649.1684.

*Bis(2-(diphenylphosphaneyl)phenyl)(2-fluorophenyl)sulfonium tetraphenylborate (**4b**[BPh$_4$])*

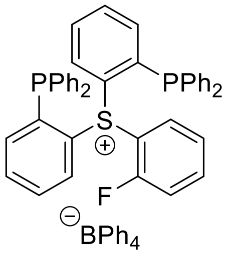


Compound **4b**[OTf] (150 mg, 0.19 mmol) was dissolved in 2.0 ml of MeOH. Separately, sodium tetraphenylborate (64.3 mg, 1.0 eq.) was dissolved in additional 2.0 ml of MeOH and added to the solution of **4b**[OTf]. Immediately a white precipitate formed, and this suspension was then stirred for additional 15-20 minutes. The precipitate was then filtered out, and washed several times with MeOH, affording 160 mg of the product, as a white powder (88% yield).

$^{1}$H NMR (400 MHz, $CD_2Cl_2$) 7.61 (t, 2H, *J* = 7.5 Hz), 7.58-7.53 (m, 1H), 7.42-7.36 (m, 6H), 7.35-7.32 (m, 5H), 7.31-7.25 (m, 14H), 7.20 (t, 1H, *J* = 9.0 Hz), 7.18-7.09 (m, 8H), 7.03 (t, 1H, *J* = 7.7 Hz), 6.98 (t, 10H, *J* = 7.3 Hz), 6.84 (tt, $BPh_4$, 4H, *J* = 7.2, 1.3 Hz), 6.72 (t, 1H, *J* = 7.3 Hz) ppm. $^{31}$P NMR (162 MHz, $CDCl_3$) -13.1 (s) ppm. $^{13}$C NMR (100 MHz, $CDCl_3$) 164.2 (q, $BPh_4$, $J_{CB}$ = 49.7), 159.7 (d, ArF, *J* = 257.0 Hz), 141.3 (d, *J* = 18.0 Hz), 137.3 (d, *J* = 8.5 Hz), 136.8 (s), 136.3 (d, $BPh_4$, *J* = 1.3 Hz), 134.6 (s), 133.7 (d, *J* = 14.8 Hz), 133.5 (d, *J* = 14.8 Hz), 132.9 (s), 132.3-132.2 (m), 132.0 (d, *J* = 6.5 Hz), 131.4 (t, *J* = 3 Hz), 130.1 (d, *J* = 1.5 Hz), 129.1 (q, *J* = 3.7 Hz), 128.8 (d, *J* = 5.6 Hz), 127.5 (d, *J* = 3.0 Hz), 125.4 (q, $BPh_4$, *J* = 2.7 Hz), 121.6 (s, $BPh_4$), 117.7 (d, *J* = 19.3 Hz), 110.6 (m, *J* = 7.2 Hz) ppm. $^{19}$F NMR (376 MHz, $CDCl_3$) -104.3 (q, ArF, *J* = 6.7 Hz) ppm. $^{19}$F{H} NMR (376 MHz, $CDCl_3$) -104.3 (s, ArF) ppm.

*Bis(2-(diphenylphosphaneyl)phenyl)(2-fluorophenyl)sulfonium hexafluorophosphate (**4b**[PF$_6$])*

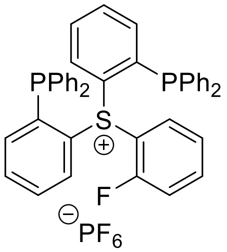


Compound **4b**[$BPh_4$] (40 mg, 41.3 μmol) was dissolved in 2.3 ml of MeCN. In another vial, $TlPF_6$ (14.4 mg, 1.0 eq.) was dissolved in additional 2.3 ml of MeCN and added to the vial containing the solution of **4b**[$BPh_4$]. Immediately the solution turned into a white emulsion that was stirred for 6 hours. The $TlBPh_4$ that formed was collected by filtration, washed with MeCN several times, and discarded. The combined filtrate was evaporated in vacuo, affording 31.4 mg of the product as colorless solid (96% yield).

$^{1}$H NMR (500 MHz, $CD_2Cl_2$) 7.66 (td, 3H, *J* = 7.5, 1.1 Hz), 7.53 (td, 2H, *J* = 7.8, 1.4 Hz), 7.40-7.35 (m, 4H), 7.35-7.30 (m, 5H), 7.30-7.24 (m, 5H), 7.24-7.18 (m, 2H), 7.18-7.10 (m, 10H), 6.89 (t, 1H, *J* = 7.5 Hz) ppm. $^{31}$P NMR (162 MHz, $CDCl_3$) -13.11 ppm (s), -143.9 (sept, $PF_6$, *J* = 710.0 Hz) ppm. $^{13}$C NMR (100 MHz, $CD_2Cl_2$) 160.5 (d, ArF, *J* = 257.0 Hz), 142.0 (d, *J* = 18.0 Hz), 137.8 (d, *J* = 8.5 Hz), 137.4 (s), 135.0 (s), 134.2 (d, *J* = 13.5 Hz), 133.9 (d, *J* = 13.5 Hz), 132.9 (s), 132.8 (d, *J* = 5.0 Hz), 132.4 (d, *J* = 5.5 Hz), 131.9 (t, *J* = 6.0 Hz), 131.1 (d, *J* = 13.5 Hz), 130.4 (d, *J* = 3.0 Hz), 129.4 (q, *J* = 3.5 Hz), 127.6 (d, *J* = 3.0 Hz), 118.4 (d, *J* = 19.3 Hz), 111.2 (dt, *J* = 7.3 Hz) ppm. $^{19}$F NMR (376 MHz, $CD_2Cl_2$) -71.7 (d, $PF_6$, *J* = 710.0 Hz), -104.7 (q, ArF, *J* = 7.0 Hz) ppm. $^{19}$F{H} NMR (376 MHz, $CD_2Cl_2$) -71.7 (d, $PF_6$, *J* = 710.0 Hz), -104.7 (s, ArF) ppm.

## 1.3 Synthetic procedures for the preparation of Rh(I) and Pt(II) sulfonium complexes

*Bis-diphenyl-phosphanylethylene(ethyl)sulfonium rhodium(I) chloride trifluoromethanesulfonate (**5a**[OTf])*

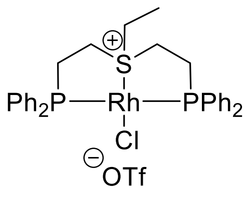


The solution of $[RhCl(COE)_2]_2$ (36 mg, 0.05 mmol) in 2 ml of dry DCM was added dropwise to the solution of sulfonium ligand **4a**[OTf] (64 mg, 0.1 mmol) in 2 ml of DCM. Upon addition, the solution first became dark red then quickly turned yellow. The solution was then stirred for 15 min, then concentrated to less than a half of its initial volume and the product was precipitated by addition of diethyl ether. The solid was then filtered off, washed with ether, and dried under vacuum, affording 70 mg of **5a**[OTf] as a yellow solid (90% yield). XRD quality single crystals were obtained by a slow diffusion of hexane into a difluorbenzene solution of **5a**[$BPh_4$] at room temperature. Under inert atmosphere, the Complex **5a**[OTf] is stable as solid and as solution in non-chlorinated solvents (more than a week). Under air, as solid the complex **5a**[OTf] is stable for more than a day, but in solution, it decomposes within a few hours.

$^{1}$H NMR (400 MHz, $CD_2Cl_2$) 7.37-7.68 (m, 20H, $PPh_2$), 4.73-4.88 (m, 2H, C$H_2$S), 3.65 (qd, SC$H_2$CH$_3$, 2H, $^{3}J_{RhH}$ = 1.3 Hz; $^{3}J_{HH}$ = 7.6 Hz), 3.20-3.35 (m, C$H_2$S, 2H and C$H_2$P, 2H), 2.50-2.61 (m, 2H, C$H_2$P), 1.34 (t, 3H, $^{3}J_{HH}$ = 7.4 Hz, SCH$_2$C$H_3$) ppm. $^{13}$C NMR (101 MHz, $CD_2Cl_2$) 133.6 (*ortho*-C, Ph), 133.3 (*ortho*-C, Ph), 132.0 (*para*-C, Ph), 131.5 (*para*-C, Ph), 131.0-132.1 (m, *ipso*-C, Ph), 129.6 (*meta*-C, Ph), 129.3 (*meta*-C, Ph), 51.3 (*C*H$_2$S), 46.9 (S*C*H$_2$CH$_3$), 30.1 (t, *C*H$_2$P, $^{2}J_{CP}$ = 13.1 Hz), 11.9

($SCH_2CH_3$). $^{31}$P NMR (162 MHz, $CD_2Cl_2$) 46.6 (d, $^1J_{RhP}$ = 127.8 Hz) ppm. $^{19}$F NMR (376 MHz, $CDCl_3$) -78.2 ppm. $^{103}$Rh NMR (16 MHz, $CD_2Cl_2$) -8577.2 (t, $^1J_{RhP}$ = 129.0 Hz) ppm. HRMS: calcd. for $C_{30}H_{33}ClP_2RhS^+$: 625.0516; found 625.0519.

*Bis-diphenyl-phosphanylethylene(ethyl)sulfonium platinum(II) dimethyl trifluoromethanesulfonate (**6a**[OTf])*

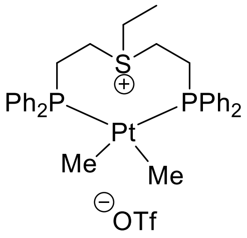

The solution of $Pt(COD)Me_2$ (34 mg, 0.1 mmol) in 2 ml of dry DCM was added dropwise to the solution of sulfonium ligand **4a**[OTf] (64 mg, 0.1 mmol) in 2 ml of DCM. The solution was stirred for 15 min, then concentrated to less than a half of the original volume and the product was precipitated by addition of diethyl ether. The solid was then filtered off, washed with ether and dried under vacuum, affording 73 mg of **6a**[OTf], as a white solid (89% yield). XRD quality single crystals were obtained by a slow diffusion of ether into a DCM solution of **6a**[$BPh_4$] at room temperature. Even under air, the Complex **7a**[OTf] is stable for days (more than a week) as solid and as solution.

$^1$H NMR (400 MHz, $CD_2Cl_2$) 7.34-7.68 (m, 20H, P*Ph₂*), 4.20-4.29 (m, 2H, $CH_2$P), 3.12-3.17 (q, $^3J_{HH}$ = 7.5 Hz, $SCH_2CH_3$, 2H and $SCH_2$, 2H), 2.98-3.06 (m, 2H, $SCH_2$), 2.87-2.92 (m, 2H, $CH_2$P), 1.09 (t, 3H, $^3J_{HH}$ = 7.4 Hz, $SCH_2CH_3$), 0.42 (AB q with Pt satellites, 6H, Pt-$CH_3$, $^2J_{PtH}$ = 69.1 Hz) ppm. $^{13}$C NMR (101 MHz, $CD_2Cl_2$) 134.4 (*ortho*-C, Ph), 132.6 (*ortho*-C, Ph), 132.2 (*ipso*-C, Ph), 131.5 (*para*-C, Ph), 131.1 (*para*-C, Ph)), 129.4 (*meta*-C, Ph), 39.6 ($SCH_2CH_3$), 39.0 ($SCH_2$), 26.6 (d, $PCH_2$), 9.7 ($SCH_2CH_3$), 7.5 (dd, 6H, Pt-$CH_3$, $^2J_{CtransP}$ = 104.0 Hz, $^2J_{CcisP}$ = 87.9 Hz) ppm. $^{31}$P NMR (162 MHz, $CD_2Cl_2$) 11.3 (s with Pt satellites, $^1J_{PtP}$ = 1813.0 Hz) ppm. $^{19}$F NMR (376 MHz, $CD_2Cl_2$) δ = -78.9 ppm. $^{195}$Pt NMR (108 MHz, $CD_2Cl_2$) δ = -4558.2 (t, $^1J_{PtP}$ = 1813.0 Hz) ppm. HRMS: calcd. for $C_{32}H_{39}P_2PtS^+$: 712.1890; found 712.1895.

*Bis-diphenyl-phosphanylethylene(ethyl)sulfonium methylplatinum(II) bis-tetrafluoroborate (**7a**[$BF_4$]$_2$)*

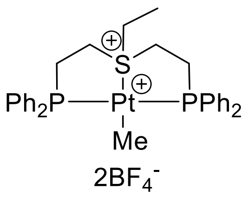

The solution of $Pt(COD)Me_2$ (66.67 mg, 0.2 mmol) in 2 mL of dry DCM was added dropwise to the solution of sulfonium ligand **4a**[$BPh_4$] (161.36 mg, 0.2 mmol) in 3 mL of DCM. The solution was stirred for 30 min, then treated with 3 eq. of $HBF_4$*$Et_2O$ (35µL) added dropwise, which resulted in vigorous gas evolution. The reaction mixture was stirred for another 30 min, during which a white precipitate formed slowly. The solution was concentrated to less than a half of its original volume and diethyl ether was added. The precipitate was then filtered off, washed with ether and dried under vacuum, affording 51 mg of **7a**[$BF_4$]$_2$, as a white solid (67%). XRD quality single crystals were obtained by a slow diffusion of ether into a DCM of **7a**[$BF_4$]$_2$ at room temperature. Even under air, the Complex **7a**[OTf] is stable for days (more than a week) as solid and as solution in non-coordinating solvents.

$^1$H NMR (400 MHz, $CD_2Cl_2$) 7.44-7.70 (m, 20H), 4.44-4.54 (m, 4H), 3.90-3.95 (m, 2H), 3.72-3.82 (m, 2H), 3.21-3.30 (m, 2H), 1.20 (3H, Pt-$CH_3$, $^2J_{PtH}$ = 39.9 Hz), 1.06 (t, 3H, $^3J_{HH}$ = 7.4 Hz, $SCH_2CH_3$) ppm. $^1$H NMR (500 MHz, $CD_3NO_2$) 7.58-7.95 (m, 20H, $PPh_2$), 4.70-4.76 (m, 2H, $SCH_2$), 4.28-4.30 (m, 2H, $SCH_2$), 4.08-4.16 (q, 2H, $SCH_2CH_3$ and m, 2H, $PCH_2$), 3.37-3.44 (m, 2H, $PCH_2$), 1.45 (t, 3H, $^3J_{HH}$ = 7.3 Hz, $SCH_2CH_3$), 1.41 (t with Pt satellites, 3H, Pt-$CH_3$, $^2J_{PtH}$ = 39.9 Hz, $^3J_{PH}$ = 7.5 Hz) ppm. $^{13}$C NMR (126 MHz, $CD_3NO_2$) 135.5 (*ortho*-C, Ph), 135.1 (*para*-C, Ph), 134.2 (*para*-C, Ph), 134.0 (*ortho*-C, Ph), 131.5 (*meta*-C, Ph), 130.9 (*meta*-C, Ph), 126.6-127.5 (*ipso*-C, Ph), 47.1 (vt, $^2J_{P\text{-}C}$ = 2.7 Hz, $SCH_2$), 43.3 ($SCH_2CH_3$), 35.7 (vt, $^1J_{P\text{-}C}$ = 18.1 Hz, $CPH_2$), 11.0 ($SCH_2CH_3$), 2.3 (t, Pt-$CH_3$, $^2J_{PC}$ = 9.8 Hz) ppm. $^{31}$P NMR (162 MHz, $CD_2Cl_2$) 42.4 (s with Pt satellites, $^1J_{PtP}$ = 2736.0 Hz) ppm. $^{195}$Pt NMR (108 MHz, $CD_3NO_2$) -4813.8 (t, $^1J_{PtP}$ = 2736.0 Hz) ppm. HRMS: calcd. for $C_{31}H_{36}BF_4P_2PtS^+$: 784.1684; found 784.1691.

*Bis-diphenyl-phosphanylethylene sulfoxide chlororhodium(I) (**8**)*

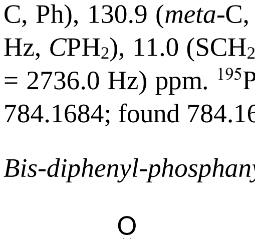

[$RhCl(COE)_2$] (7 mg, 0.01 mmol), dissolved in 1 mL of DCM was added at once to a solution of PS(O)P[5] (10 mg, 0.02 mmol) in an additional 1 mL of dry DCM. The mixture was stirred at room temperature for 30 mins, after which all volatiles were evaporated under vacuum. The residue was dissolved in a minimum amount of DCM and ether was added to precipitate out the complex **8**. The precipitate was collected by filteration and dried under vacuum, affording complex **8** (12 mg, 0.019 mmol, 95%). Crystals suitable for single crystal X-ray diffraction were grown by vapor diffusion of ether into a DCM solution of complex **8** at r.t.

$^1$H NMR ($CDCl_3$, 500 MHz) 7.95-7.96 (8H, m, P*Ph₂*), 7.35-7.42 (12H, m, P*Ph₂*), 3.68-3.78 (2H, m, $PCH_2CH_2S(O)$), 2.82-2.90 (6H, m, $PCH_2CH_2S(O)$). $^{13}$C NMR ($CDCl_3$, 126 MHz) 134.0 (t, *o*-P*Ph*, $^2J_{PC}$ = 7.1 Hz), 133.2 (t, *o-Ph*, $^2J_{PC}$ = 7.1 Hz), 133.4 (m,

*ipso*-P*Ph*), 130.6 (s, *p*-P*Ph*), 130.5 (s, *p*-P*Ph*), 128.9 (t, *m*-P*Ph*, $^3J_{P-C}$ = 4.6 Hz), 128.8 (t, *m*-P*Ph*, $^3J_{PC}$ = 4.6 Hz), 60.7 (dt, $PCH_2CH_2S(O)$, $^2J_{PC}$ = 8.3 Hz, $^2J_{RhC}$ = 5.5 Hz), 27.0 (td, $PCH_2CH_2S(O)$, $^1J_{PC}$ = 13.0 Hz, $^2J_{RhC}$ = 2.6 Hz). $^{31}P\{^1H\}$ NMR ($CDCl_3$, 202 MHz) 40.5 (d, $^1J_{RhP}$ = 147.5). HRMS (ESI +ve mode): m/z calcd for $[M-Cl]^+$ $C_{28}H_{28}OP_2RhS^+$ 577.0386, found 577.0383, m/z calcd for $[M-Cl+MeCN]^+$ $C_{30}H_{31}NOP_2RhS^+$ 618.0651, found 618.0652.

*Bis-diphenyl-phosphanylethylene sulfide methylplatinum(II) bis-tetrafluoroborate (**9**[BF$_4$]$_2$)*

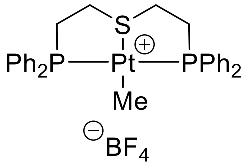


Pt(COD)MeCl (17.5 mg, 0.05 mmol), dissolved in 1 mL of dry DCM was added dropwise to a solution of **1a** (22.9 mg, 0.05 mmol) dissolved in 1.5 mL of DCM. The solution was stirred for 10 min, then treated with 1.5 equiv. of $AgBF_4$ (14.6 mg) and stirred for additional 30 min, while protected from light by aluminum paper. After 30 min, the precipitate was filtered off and washed with ether, the filtrate was dried under reduced vacuum, affording **9**[BF$_4$] as a white solid (29 mg, 0.0384 mmol, 76.8 %). XRD quality single crystals were obtained by a slow evaporation of a DCM solution of **9**[BF$_4$] at room temperature. Even under air, the Complex **7a**[OTf] is stable for days (more than a week) as solid and as solution.

$^1$H NMR (400 MHz, $CDCl_3$) 7.45-7.69 (m, 20H, $PPh_2$), 3.86-4.04 (m, 2H, $SCH_2$), 3.56-3.66 (m, 2H, $PCH_2$), 3.05-3.13 (m, 2H, $SCH_2$), 2.59-2.66 (m, 2H, $PCH_2$), 0.79-0.89 (triplet with Pt satellite, 3H, $CH_3$-Pt, $^2J_{PtH}$ = 74.2 Hz, $^3J_{PH}$ = 6.4Hz). $^{13}$C NMR (101 MHz, $CDCl_3$) 133.3 (*ortho*-C, Ph), 132.6 (*ortho*-C, Ph), 132.2 (*para*-C, Ph), 131.8 (*para*-C, Ph), 129.3-129.5 (*meta*-C, Ph), 128.0-128.9 (*ipso*-C, Ph), 36.9 (t, P*C*H$_2$) ppm, 35.3 (t, S*C*H$_2$), -9.7 (t, $^2J_{PC}$ = 5.6 Hz, $^1J_{PtC}$ = 605.7 Hz, *C*H$_3$-Pt) ppm. $^{31}$P NMR (162 MHz, $CD_2Cl_2$) $\delta$ = 37.8 (s with Pt satellites, $^1J_{PtP}$ = 2923.0 Hz) ppm. HRMS: calcd. for $C_{29}H_{31}P_2PtS^+$: 668.1264; found 668.1270.

*Bis(2-(diphenylphosphaneyl)phenyl)(2-fluorophenyl)sulfonium chlororordhodium(I) trifluoromethanesulfonate (5**b**[OTf])*

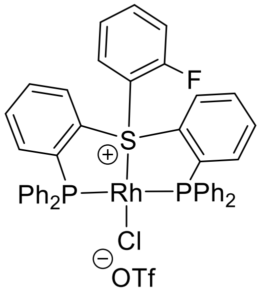


Ligand **4b**[OTf] (100 mg, 0.125 mmol) was dissolved in 2.0 ml DCM in a vial. In another vial, $[RhCl(COE)_2]_2$ (44.9 mg, 0.5 eq.) was dissolved in additional 2.0 ml of DCM as well and added to the solution of **4b**[OTf]. The solution that immediately turned dark red orange was left stirring overnight. The solvent was then removed in vacuo and the residue was washed several times with hexane to afford the product, as a brown-yellowish powder. (0.12 g, 97% yield). As all attempts to grow single crystals of **5b**[OTf] suitable for XRD analysis failed, instead we prepared compound **5b**[PF$_6$] from ligand **4b**[PF$_6$] following the same procedure. This time XRD quality crystals were obtained by a layer diffusion of toluene into a concentrated DCM solution of **5b**[PF$_6$]. Under inert atmosphere, the Complex **5b**[OTf] is stable as solid and as solution in non-chlorinated solvents (more than a week). Under air, as solid the complex **5a**[OTf] is stable for more than a day, but in solution, it decomposes within a few hours.

$^1$H NMR (500 MHz, $CDCl_3$) 8.88 (d, 2H, *J* = 8.3 Hz), 8.15 (t, 2H, *J* = 7.7 Hz), 7.88 (t, 2H, *J* = 7.5 Hz), 7.80-7.76 (m, 2H), 7.66 (q, 4H, *J* = 6.0 Hz), 7.52 (q, 4H, *J* = 6.5 Hz), 7.50-7.42 (m, 5H), 7.42-7.36 (m, 8H), 6.91 (t, 1H, *J* = 7.9 Hz), 6.85 (t, 1H, *J* = 9.5 Hz), 6.69 (t, 1H, *J* = 7.6 Hz) ppm. $^{31}$P NMR (162 MHz, $CDCl_3$) 48.7 (dd, *J* = 126.0, 6.0 Hz) ppm. $^{13}$C NMR (125MHz, $CDCl_3$) 157.4 (d, ArF, *J* = 257.0 Hz), 138.9 (vt, *J* = 15.6 Hz), 138.5 (vtd, *J* = 19.3, 1.7 Hz), 136.2 (d, *J* = 8.5 Hz), 135.8 (s), 135.4 (s), 135.0 (s), 133.8 (t, *J* = 6.7 Hz), 133.6 (t, *J* = 7.6 Hz), 131.5 (d, *J* = 9.5 Hz), 130.2 (t, *J* = 5.0 Hz), 129.2 (s), 129.1 (t, *J* = 5.3 Hz), 128.8 (t, *J* = 5.5 Hz), 128.2 (vt, *J* = 21.9 Hz), 125.6 (d, *J* = 3.2 Hz), 120.8 (q, OTf, *J* = 320.0 Hz), 120.0 (d, *J* = 11.7 Hz), 118.1 (d, *J* = 20.1 Hz) ppm. $^{19}$F NMR (376 MHz, $CDCl_3$) -76.5 (s, OTf), -104.1 (m, ArF) ppm. $^{19}F\{H\}$ NMR (376 MHz, $CDCl_3$) -76.5 (s, OTf), -104.1 (t, ArF, *J* = 6.0 Hz) ppm. HRMS: calcd. For $C_{42}H_{32}ClFP_2RhS^+$: 787.0422; found 787.0345.

*Bis(2-(diphenylphosphaneyl)phenyl)(2-fluorophenyl)sulfonium dimethylplatinum(II) trifluoromethanesulfonate (6**b**[OTf])*

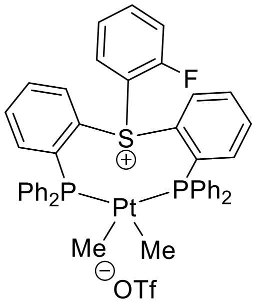


Ligand **4b**[OTf] (50 mg, 62.6 μmol) was placed in a glass pressure tube and dissolved in 1.6 ml of THF. In a separate vial, Pt(COD)$Me_2$ (20.9 mg, 1 eq.) was dissolved in additional 1.6 ml of THF and added to the pressure tube containing the solution of **4b**[OTf]. The tube was sealed off, placed in an oil bath at 65°C and left stirring overnight gradually forming a white precipitate. After allowing to cool to r.t., the pressure tube was opened, the precipitate was filtered and washed several times with hexane affording 34 mg the product, as a white powder (53% yield). Even under air, the Complex **7a**[OTf] is stable for days (more than a week) as solid and as solution, however, decomposes under prolonged vacuum. Thus, clean NMR without residual solvent could not be obtained for complex **7b**[OTf].

$^{1}$H NMR (500 MHz, $CD_2Cl_2$) 7.76 (dddd, 1H, $J$ = 8.0, 5.5, 1.5, 0.6 Hz), 7.59 (t, 2H, $J$ = 7.7 Hz), 7.48 (br t, 4H, $J$ = 8.2 Hz), 7.43-7.34 (m, 7H), 7.34-7.26 (m, 9H), 7.22 (td, 4H, $J$ = 7.5, 1.9 Hz), 7.14 (br t, 2H, $J$ = 7.0 Hz), 7.04 (td, 1H, $J$ = 7.6, 1.5 Hz), 6.91 (br s, 2H), 0.65 (AB q with Pt satellites, Pt*Me*$_2$, 6H, $J$ = 71.2 Hz) ppm. $^{31}$P NMR (162 MHz, $CD_2Cl_2$) 16.6 (br s with Pt satellites, $J$ = 1781.0 Hz) ppm. $^{13}$C NMR (100 MHz, $CD_2Cl_2$) 160.7 (d, ArF, $J$ = 255.0 Hz), 137.6 (d, $J$ = 8.5 Hz), 137.5-136.1 (m), 135.6 (d, $J$ = 8.0 Hz), 134.2 (d, $J$ = 12.0 Hz), 133.8 (s), 133.6 (d, $J$ = 3.6 Hz), 132.6 (s), 131.5 (s), 131.1 (s, $J$ = 13.2), 130.6 (s), 128.9 (d, $J$ = 10 Hz), 128.6 (d, $J$ = 10 Hz), 127.1 (d, $J$ = 2.5 Hz), 121.4 (q, OTf, $J$ = 321.5 Hz), 118.0 (d, $J$ = 19 Hz), 105.4 (d, $J$ = 15.2 Hz), 9.6 (dd, Pt*Me*$_2$) ppm. $^{19}$F{H} NMR (376 MHz, $CD_2Cl_2$) -77.2 (s, OTf), -102.3 (s, ArF) ppm. $^{195}$Pt NMR (108 MHz, $CD_2Cl_2$) -4390.2 (t, $J$ = 1781.0 Hz) ppm. HRMS: calcd. For $C_{44}H_{38}FP_2PtS^+$: 874.1796; found 874.1885.

*Bis(2-(diphenylphosphaneyl)phenyl)(2-fluorophenyl)sulfonium methylplatinum(II) bis-trifluoromethanesulfonate (**7b**[OTf]$_2$)*

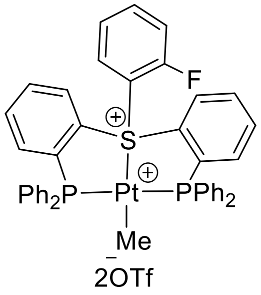


**6b**[OTf]) (15 mg, 14.6 μmol) was dissolved in 1.0 ml DCM in a vial forming a light-yellow transparent solution. To this solution 1.3 μL (1 eq.) of triflic acid were added dropwise. The solution that turned colorless was left closed to stir for 40 minutes. The solvent was removed in vacuo to afford the product as a light-yellow powder. (14.8 mg, quantitative yield). As all attempts to grow single crystals of **6b**[OTf]$_2$ suitable for XRD analysis failed, instead we prepared compound **6b**[$NTf_2$]$_2$ by treating complex **6b**[OTf] with an excess of bistriflimide. XRD quality crystals were then obtained by a layer diffusion of toluene into its concentrated DCM solution. Even under air, the Complex **7a**[OTf] is stable for days (more than a week) as solid and as solution in non-coordinating solvents.

$^{1}$H NMR (500 MHz, $CD_2Cl_2$) 9.11 (d, 2H, $J$ = 8.5 Hz), 8.29 (t, 2H, $J$ = 8.0 Hz), 8.13 (td, 2H, $J$ = 7.6, 0.7 Hz), 7.91 (qd, 2H $J$ = 4.79, 1.33 Hz), 7.72-7.67 (m, 2H), 7.64-7.56 (m, 11H), 7.56-7.51 (m, 4H), 7.47-7.40 (m, 4H), 7.15 (dt, 1H, $J$ = 7.9, 1.2 Hz), 7.05-6.99 (m, 2H,), 1.56 (t with Pt satellites, Pt*Me*, 3H, $J$ = 41.2, 7.0 Hz) ppm. $^{31}$P NMR (162 MHz, $CD_2Cl_2$) 44.34 (d with Pt satellites, $J$ = 2768.0, 3.3 Hz) ppm. $^{13}$C NMR (125 MHz, $CD_2Cl_2$) 158.2 (d, ArF, $J$ = 259.5 Hz), 139.8 (d, $J$ = 9.1 Hz), 138.8 (vt, $J$ = 3.5 Hz), 138.1 (s), 137.9 (vt, $J$ = 10 Hz), 134.5 (vt, $J$ = 7.8 Hz), 134.4 (vt, $J$ = 3.3 Hz), 134.3 (s), 134.1 (vt, $J$ = 6.7 Hz), 134.1 (s), 130.7 (vt, $J$ = 6 Hz), 127.9 (d, $J$ = 3 Hz), 123.7 (vt, $J$ = 30 Hz), 122.2 (vt, $J$ = 32.2 Hz), 121.2 (q, OTf, $J$ = 320.5 Hz), 119.0 (d, $J$ = 19.0 Hz), 112.8 (d, $J$ = 12.8 Hz), 4.74 (t with Pt satellites, Pt*Me*, $J$ = 318.0, 4.5 Hz) ppm. $^{19}$F NMR (376 MHz, $CD_2Cl_2$) -77.26 (s, OTf, 6F), -102.45 (bs, ArF, 1F) ppm. $^{19}$F{H} NMR (470 MHz, $CD_2Cl_2$) -77.26 (s, OTf, 6F), -102.45 (t, ArF, 1F, $J$ = 3.3 Hz) ppm. $^{195}$Pt NMR (108 MHz, $CD_2Cl_2$) -4811.0 (t, $^{1}J_{PtP}$ = 2768.0 Hz) ppm. HRMS: calcd. For $C_{43}H_{35}FP_2PtS^+$: 859.1561; found 859.1521.

### 1.4 $^1$H NMR spectra comparison

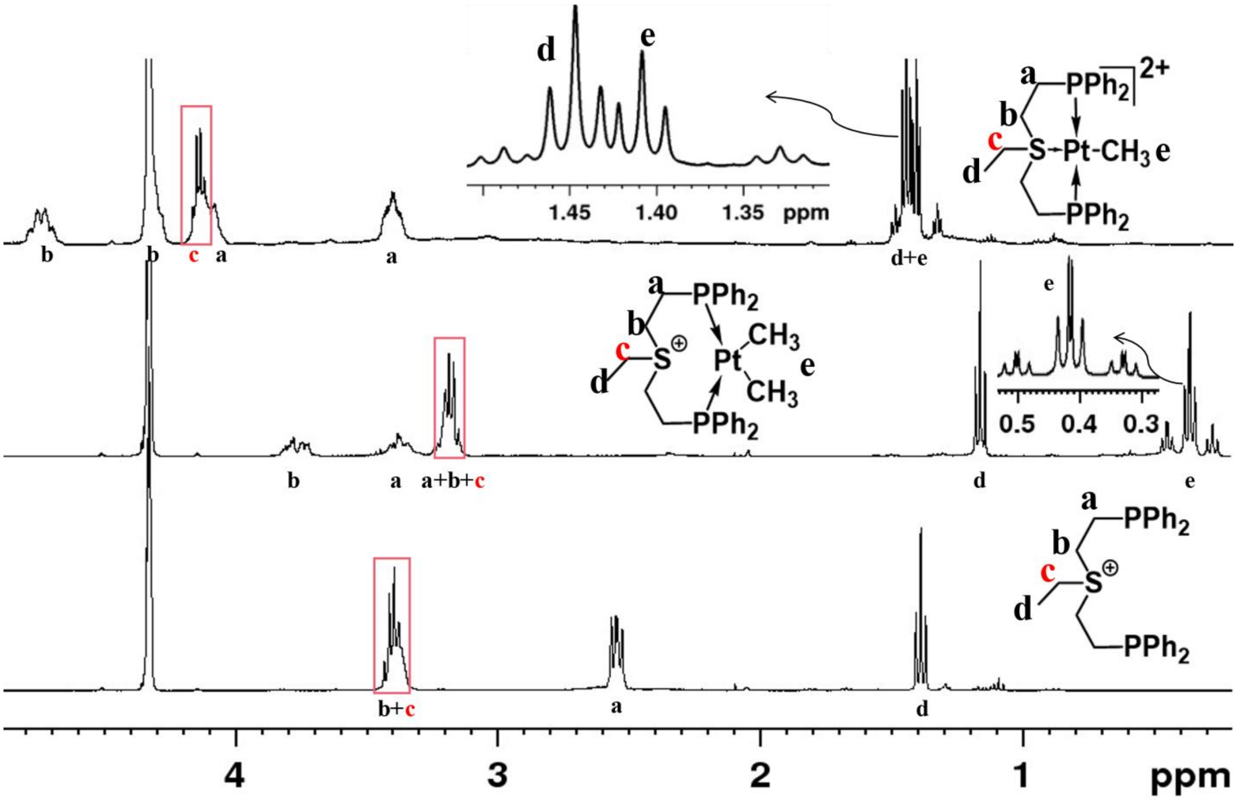


**Figure S1**: Part of $^1$H NMR (400 MHz) of **4a**[OTf], **6a**[OTf] and **7a**[$BF_4$]$_2$ in $CD_3NO_2$, measured at 298 K.

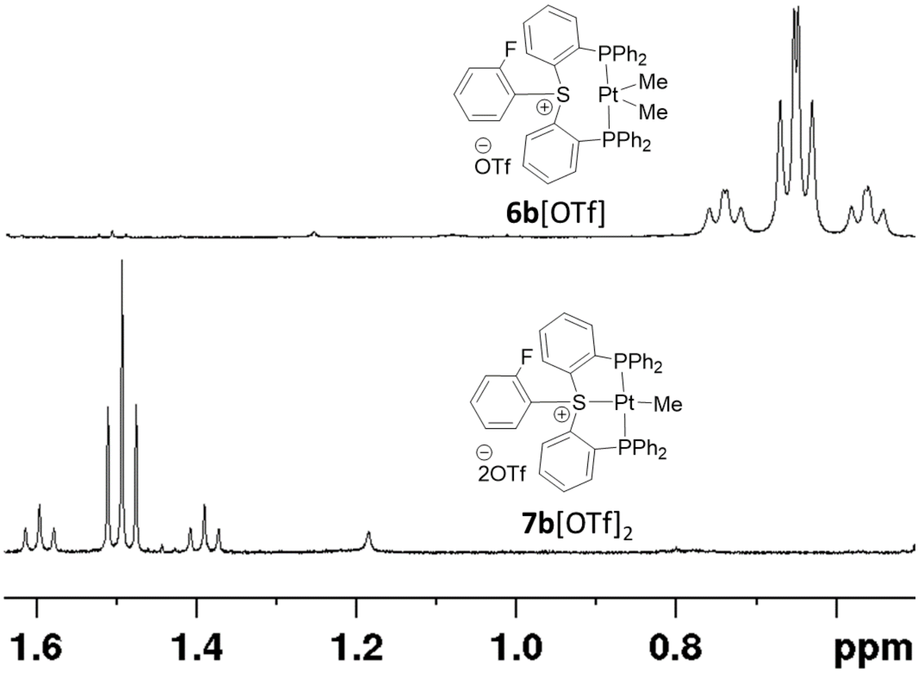


**Figure S2**: Part of $^1$H NMR (400 MHz) of **6b**[OTf], and **7b**[OTf]$_2$ in $CD_2Cl_2$, measured at 298 K.

**1.5. Selected 2D NMR spectra of the complexes.**

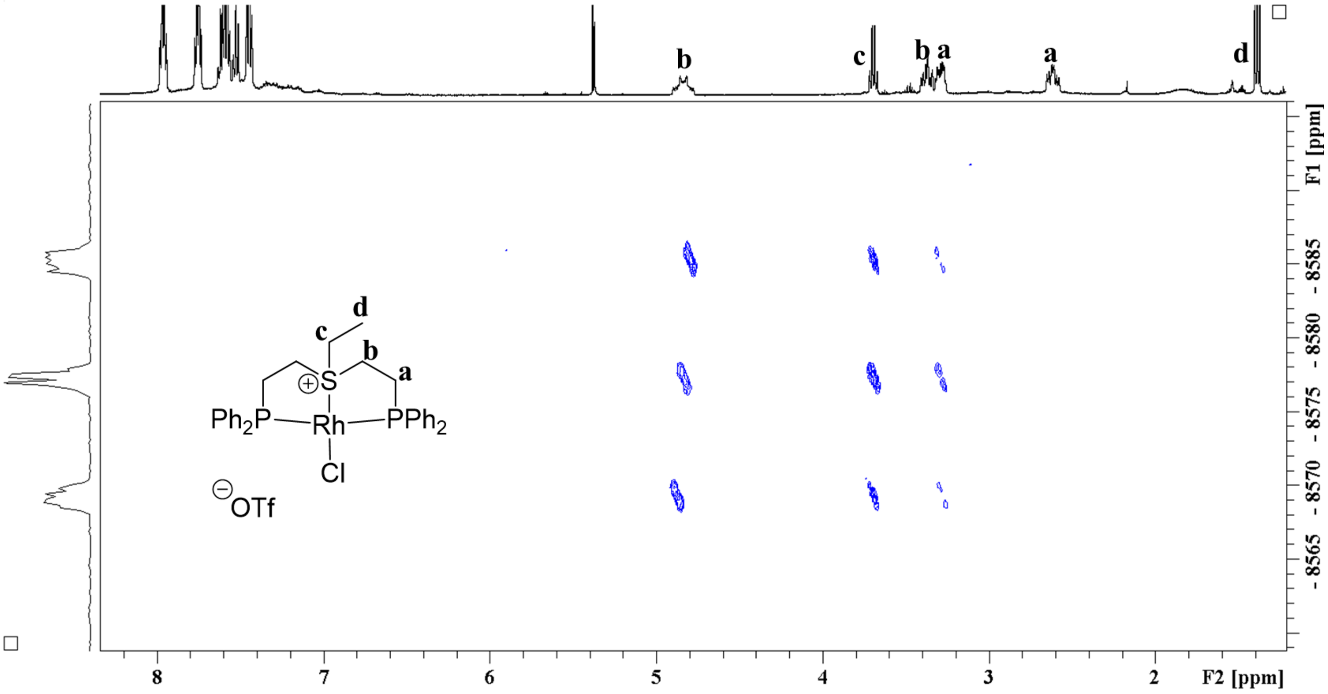


**Figure S3**: $^{103}$Rh-$^{1}$H HMBC NMR (500 MHz) of **5a**[OTf] in $CD_2Cl_2$, measured at 298 K.

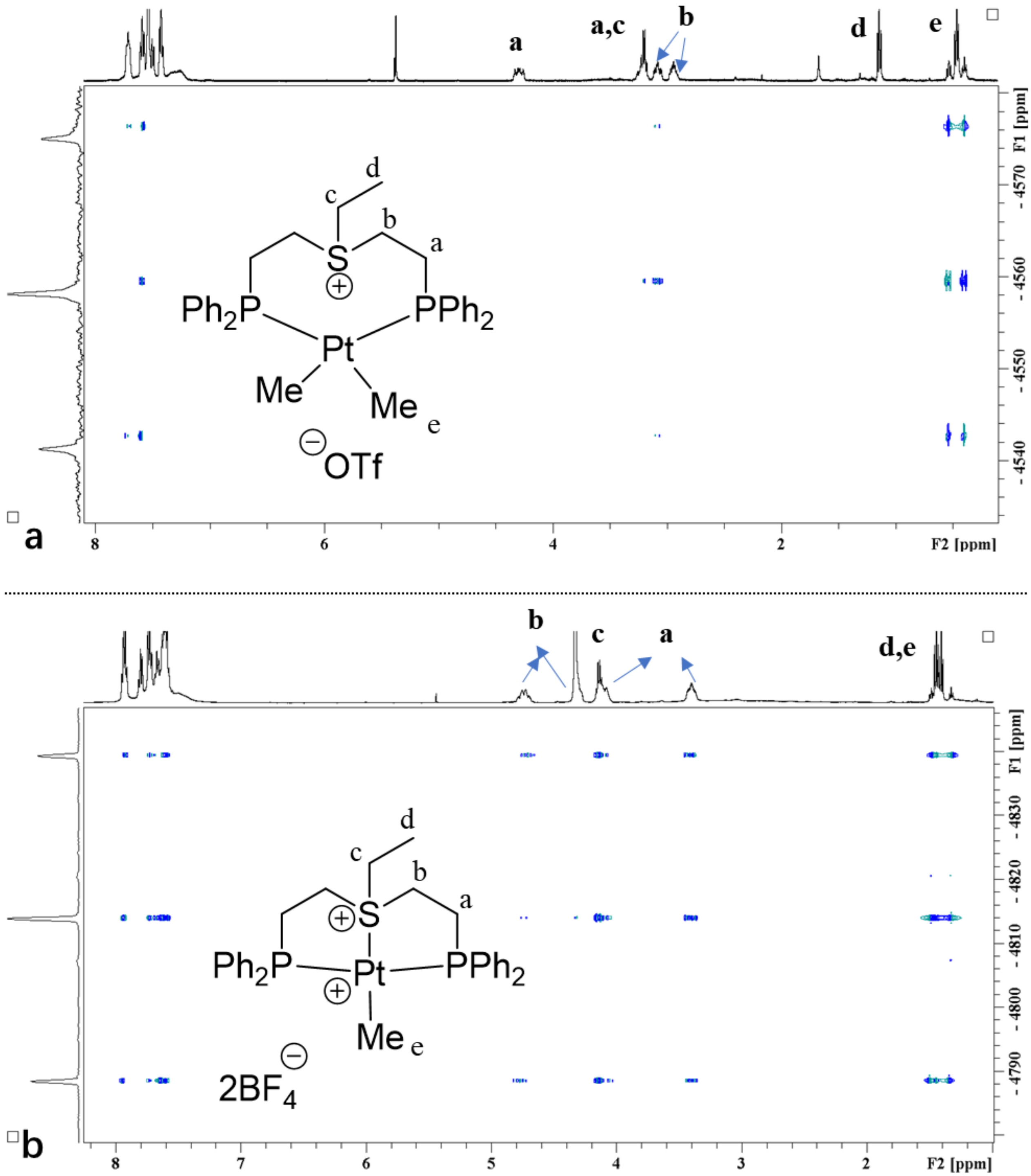


**Figure S4**: $^{195}$Pt-$^{1}$H HMBC NMR (500 MHz) of **6a**[OTf] in $CD_2Cl_2$ (**a**) and **7a**$[BF_4]_2$ in $CD_3NO_2$, (**b**) measured at 298 K.

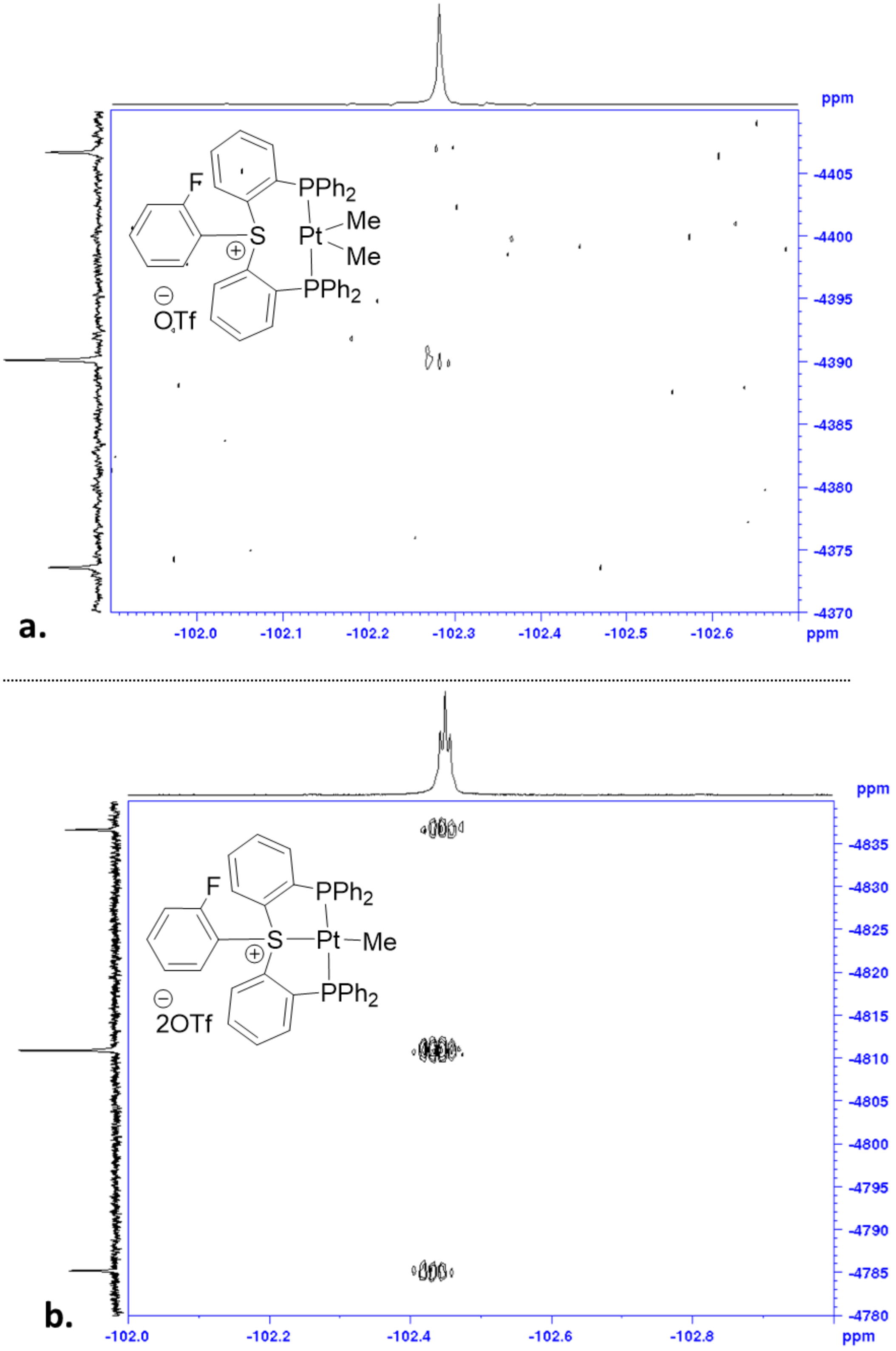


**Figure S5**: Comparison of $^{195}$Pt-$^{19}$F HMBC NMR (500 MHz) of **6b**[OTf] and **7b**$[OTf]_2$ in $CD_2Cl_2$, measured at 298 K.

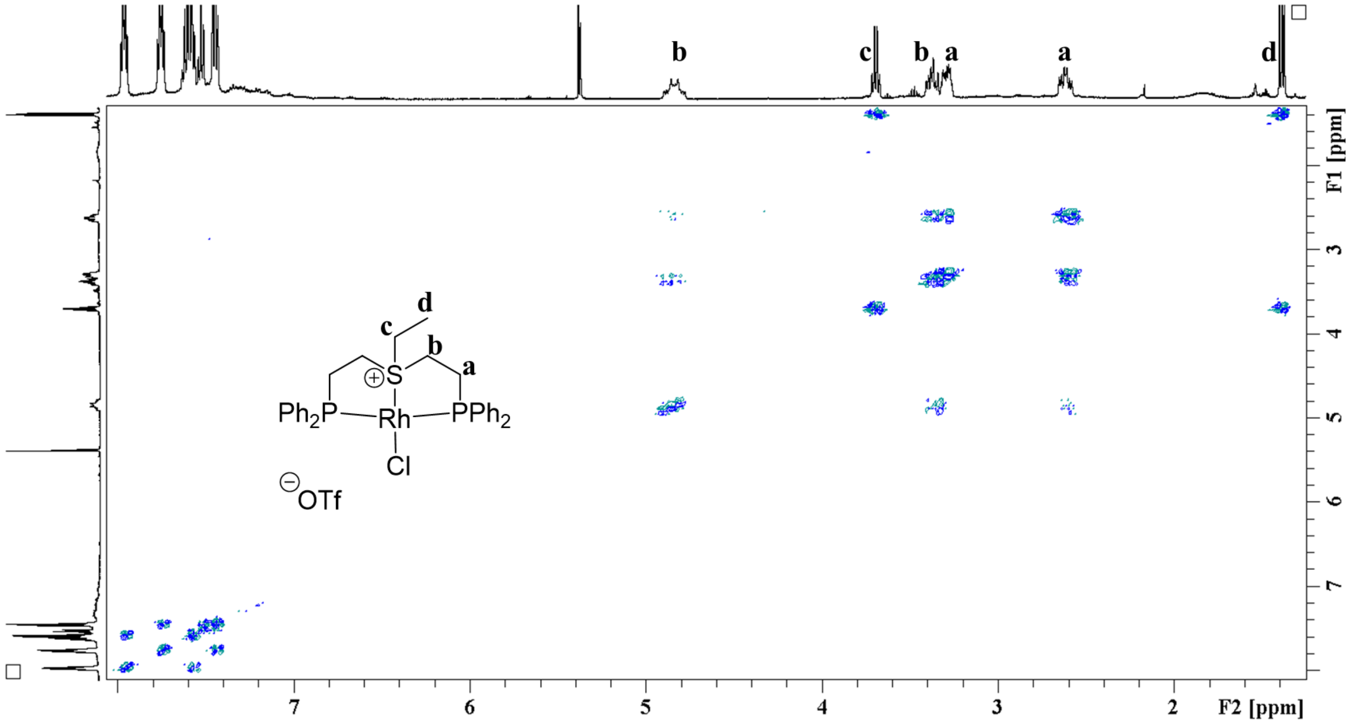


**Figure S6**: COSY NMR (400 MHz) of **5a**[OTf] in $CD_2Cl_2$, measured at 298 K.

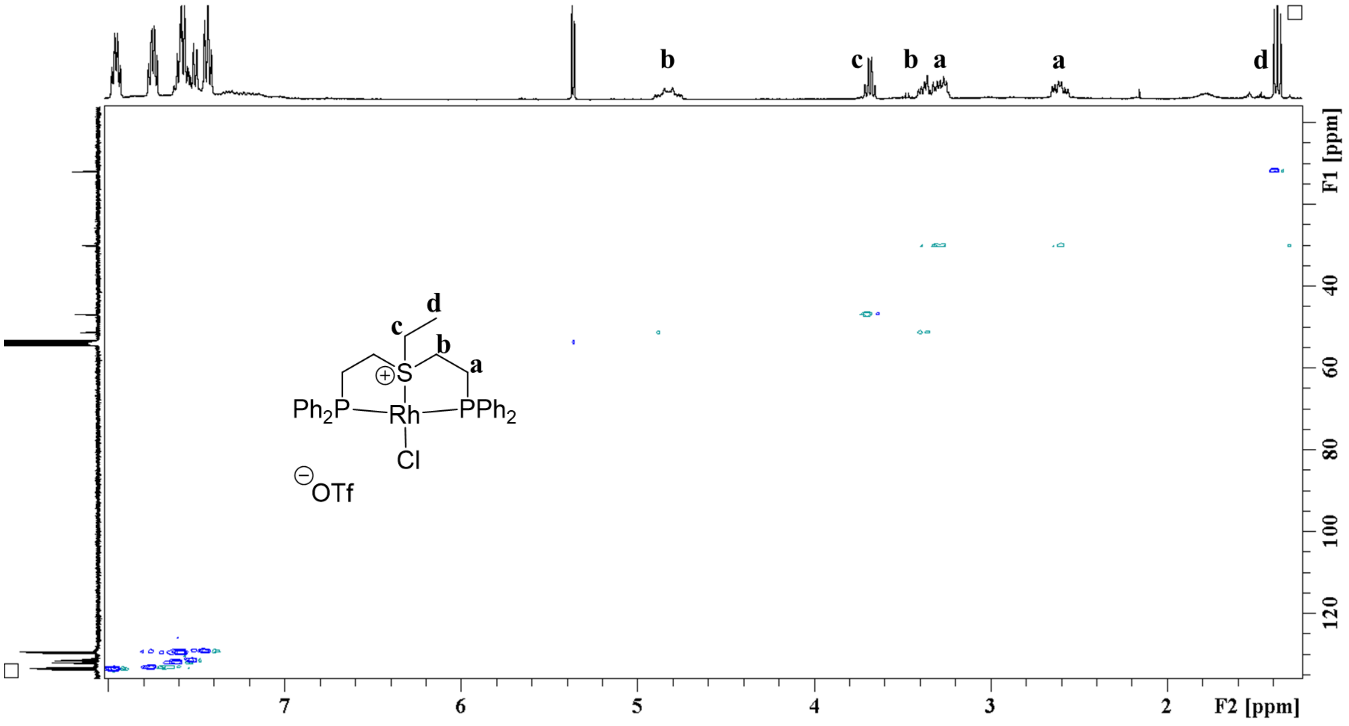


**Figure S7**: HSQC NMR (400 MHz) of **5a**[OTf] in $CD_2Cl_2$, measured at 298 K.

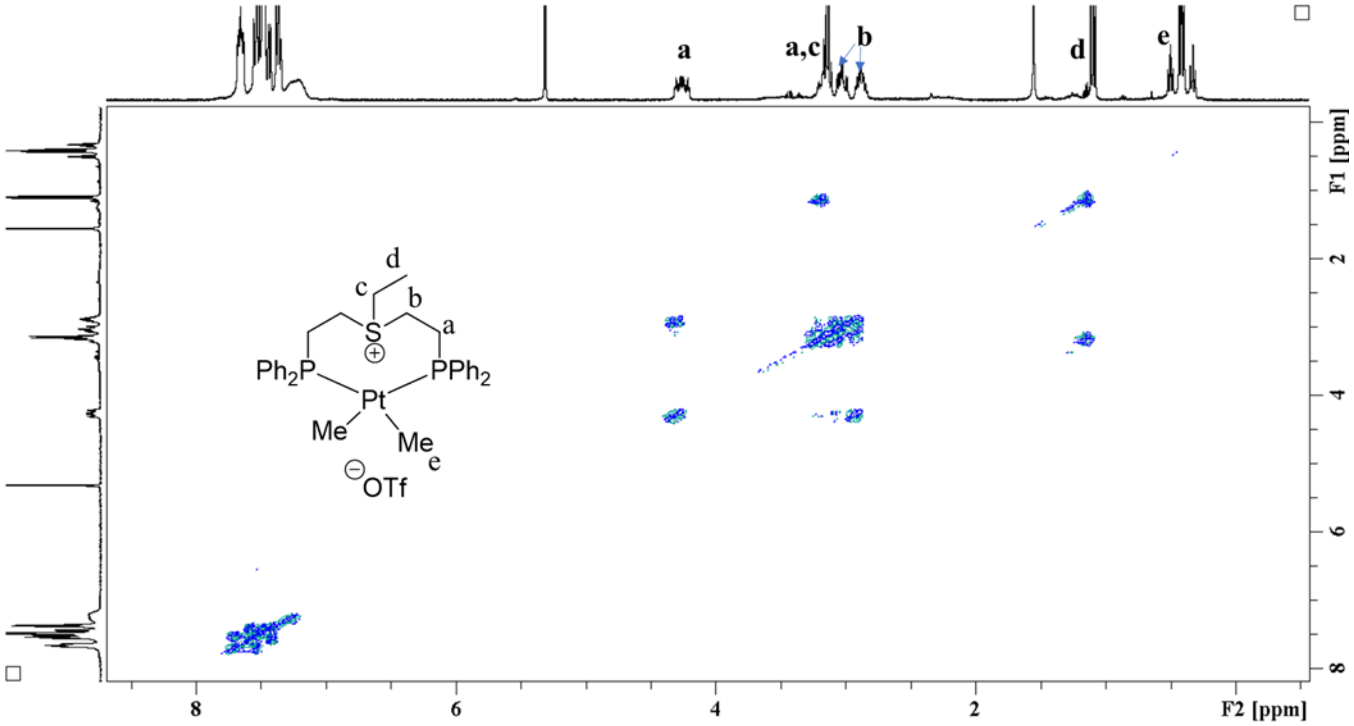


**Figure S8**: COSY NMR (400 MHz) of **6a**[OTf] in $CD_2Cl_2$, measured at 298 K.

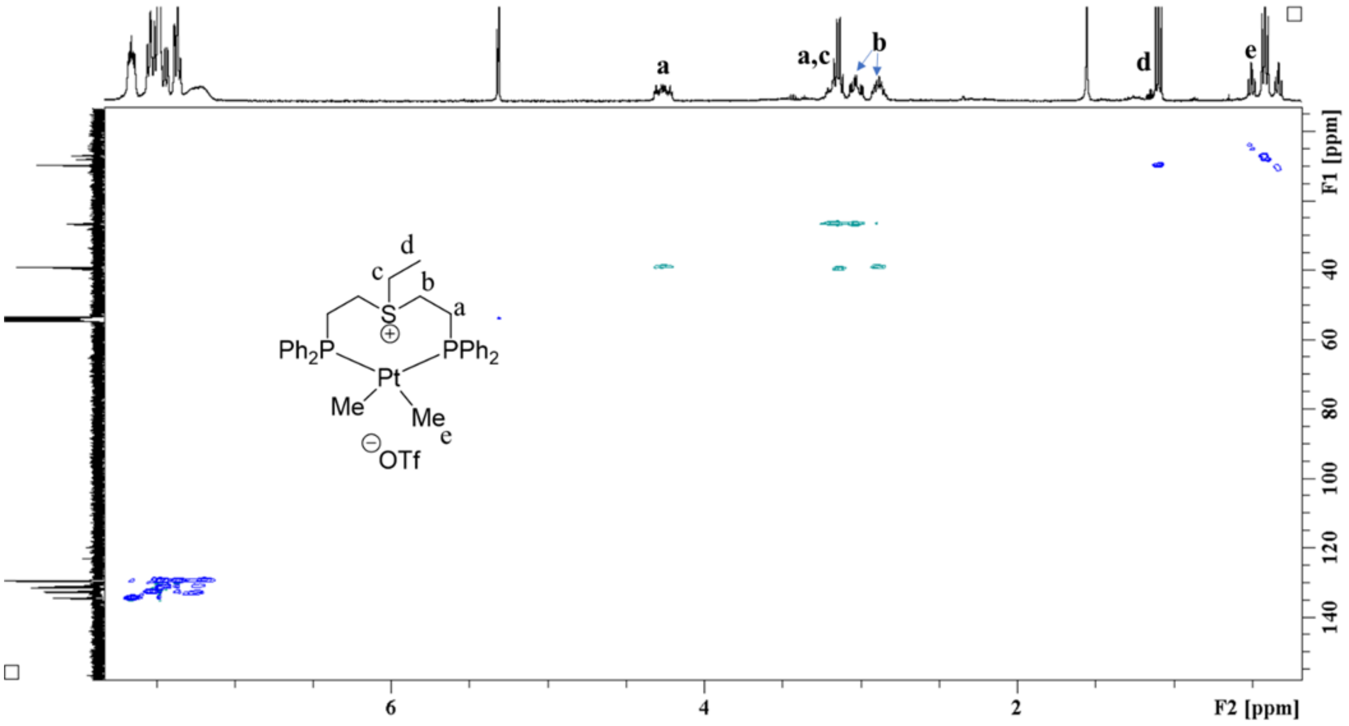


**Figure S9**: HSQC NMR (400 MHz) of **6a**[OTf] in $CD_2Cl_2$, measured at 298 K.

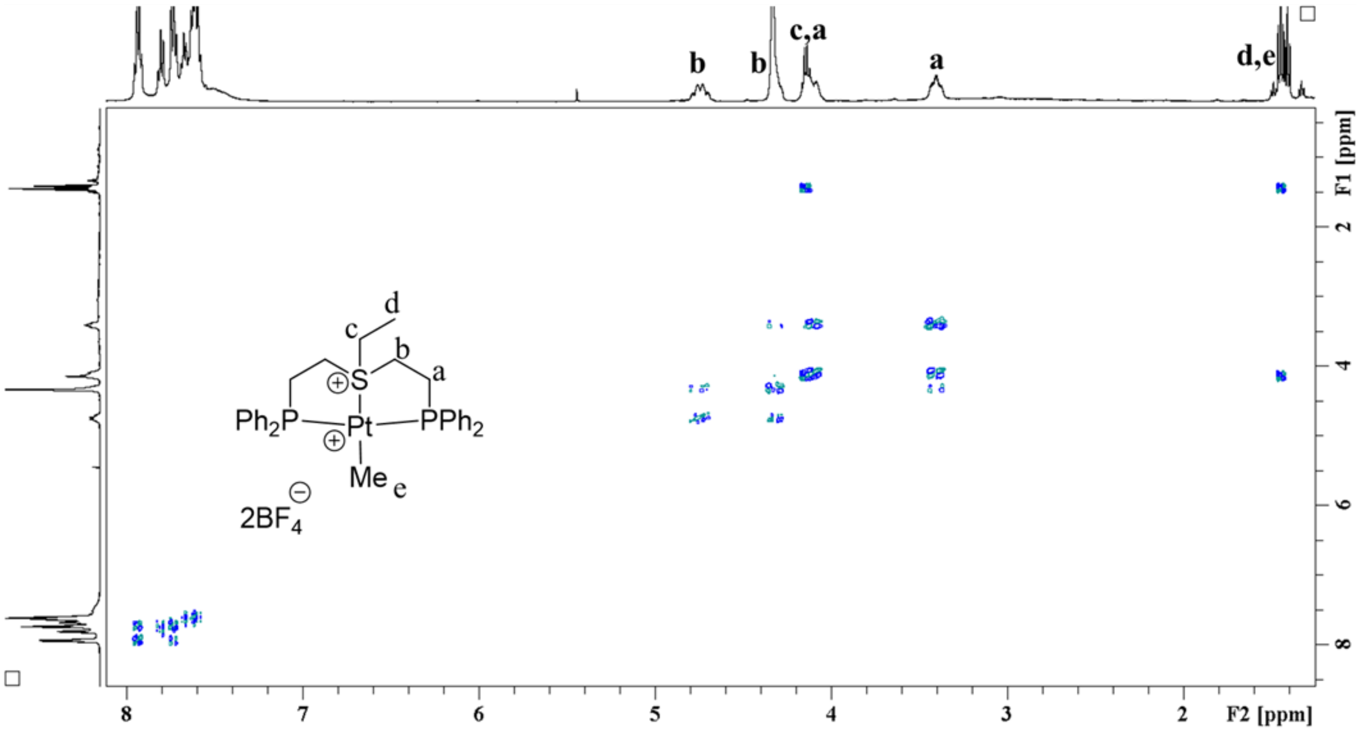


**Figure S10**: COSY NMR (400 MHz) of **7a**$[BF_4]_2$ in $CD_3NO_2$, measured at 298 K.

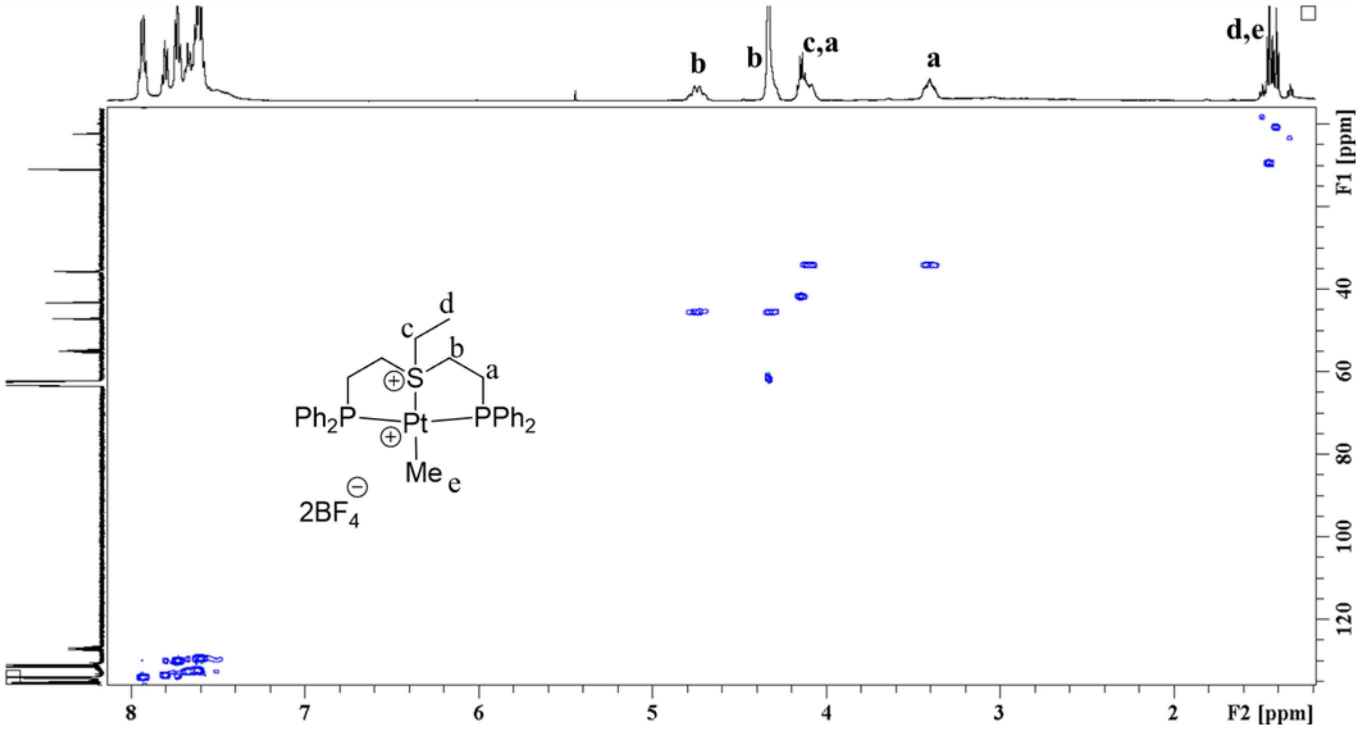


**Figure S11**: HSQC NMR (400 MHz) of **7a**$[BF_4]_2$ in $CD_3NO_2$, measured at 298 K.

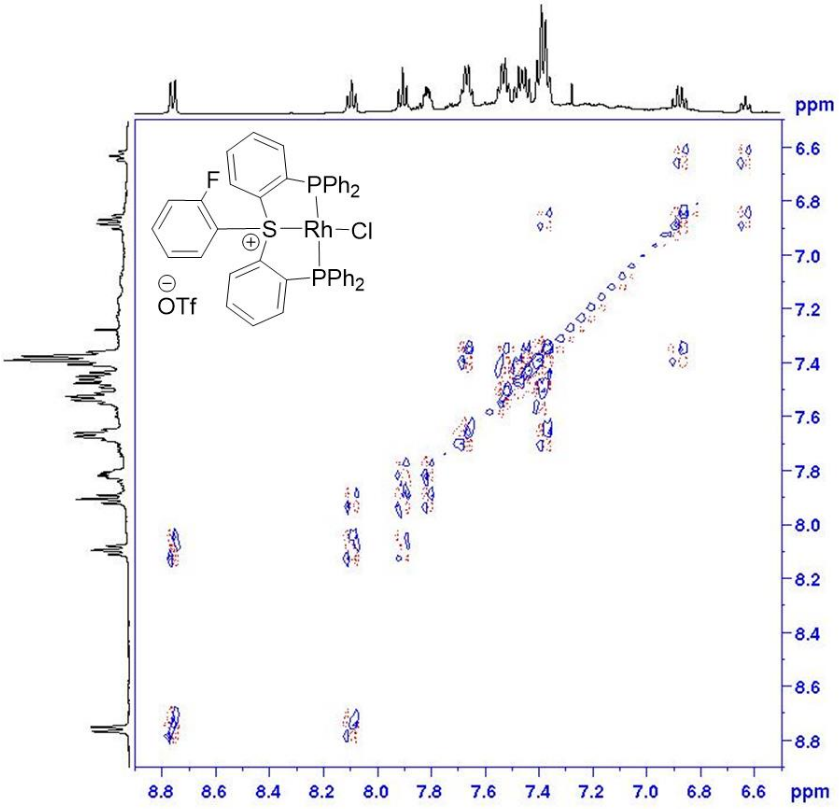


**Figure S12**: COSY NMR (400 MHz) of **5b**[OTf] in $CDCl_3$, measured at 298 K.

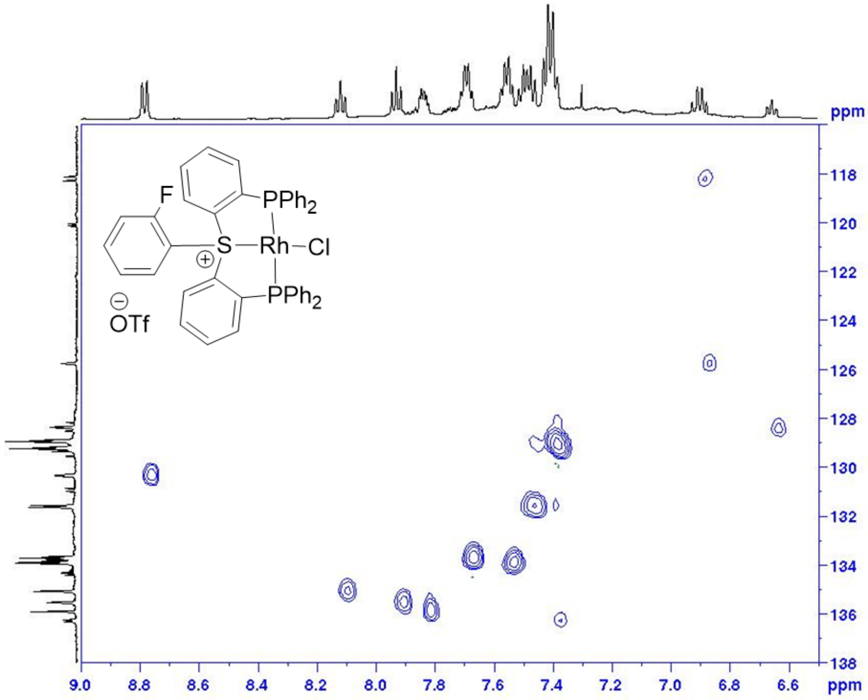


**Figure S13**: HSQC NMR (500 MHz) of **5b**[OTf] in $CDCl_3$, measured at 298 K.

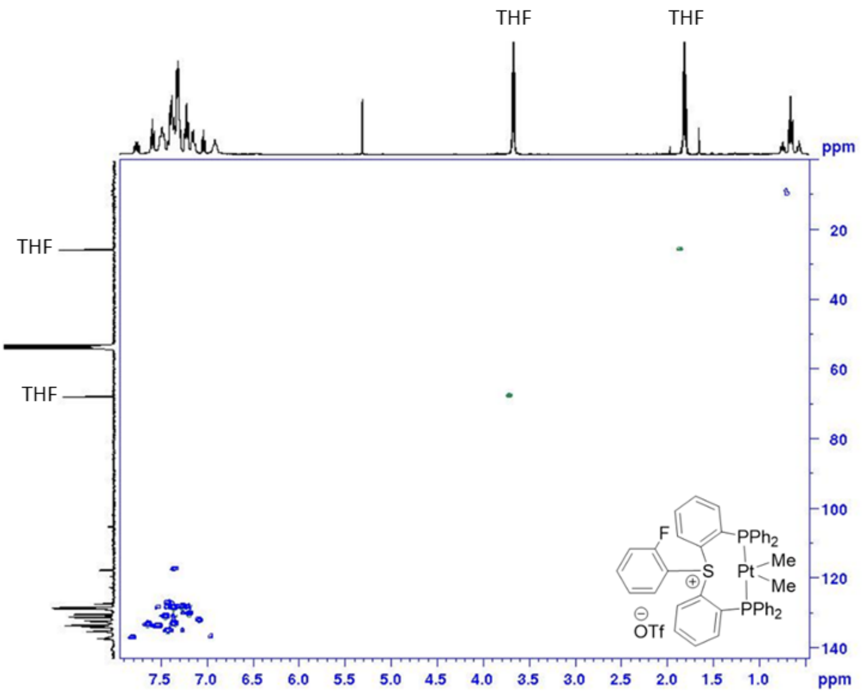


**Figure S14**: HSQC NMR (400 MHz) of **6b**[OTf] in $CD_2Cl_2$, measured at 298 K.

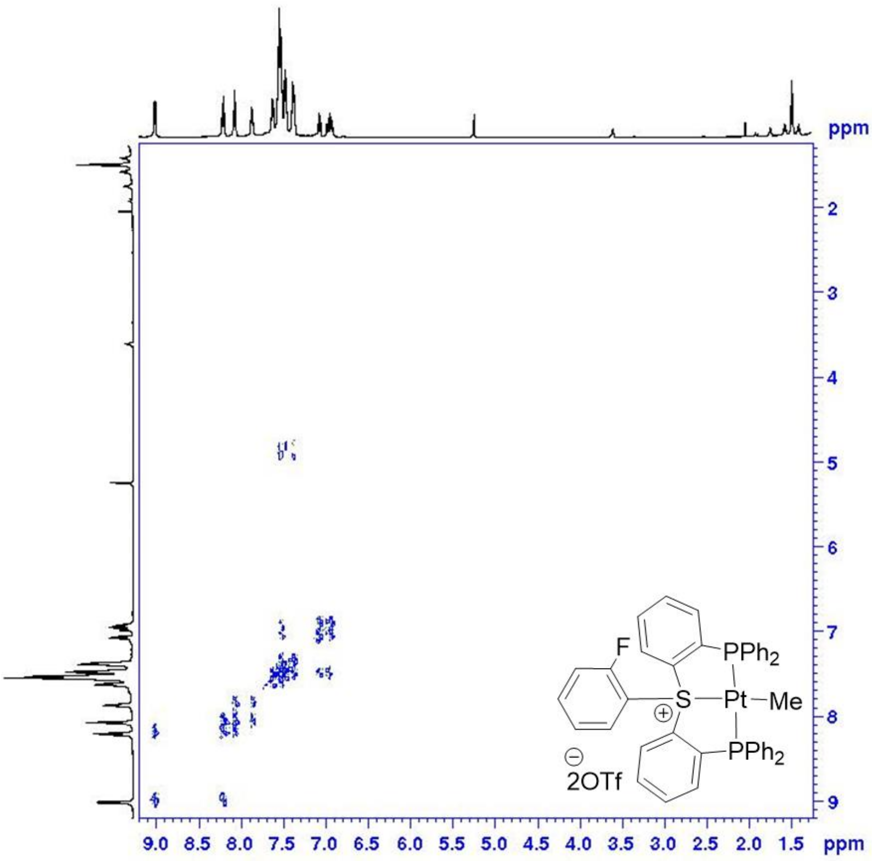


**Figure S15**: COSY NMR (500 MHz) of **7b**$[OTf]_2$ in $CD_2Cl_2$, measured at 298 K.

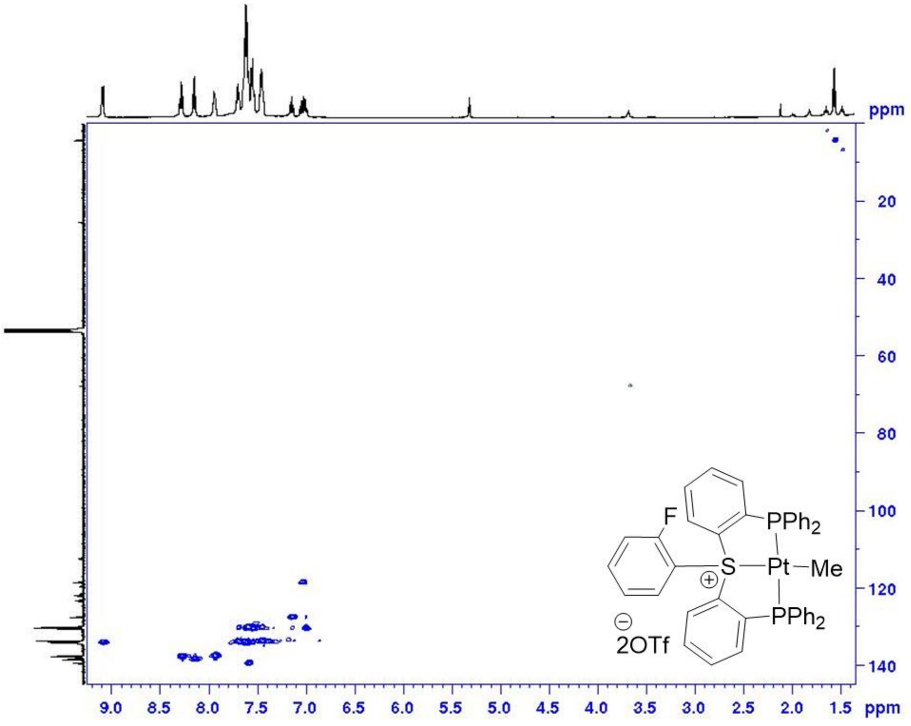


**Figure S16**: HSQC NMR (500 MHz) of **7b**[OTf]$_2$ in $CD_2Cl_2$, measured at 298 K.

## 2. Crystallography

### 2.1. General crystallographic and refinement details:

Suitable single crystals of **4a**[$BPh_4$], **4b**[OTf], **5a**[$BPh_4$], **5b**[$PF_6$], **6a**[$BPh_4$], **7a**[$BF_4$]$_2$, **7b**[$NTf_2$]$_2$, **8** and **9**[$BF_4$] were grown from the solvents mentioned in their respective synthetic procedures. Single-crystal X-ray diffraction data collections were performed using Rigaku goniometer diffractometer with graphite monochromated Mo Kα radiation (λ = 0.71073 Å). The diffraction intensity details were extracted from the diffraction frames using CrysAlisPro XtalLAB Synergy-S system (version 41.112a) program. The structures were then solved by SHELXT-9740 available in Olex2 crystallographic suite, which located most of the non-hydrogen atoms. Subsequently, least-squares refinements were carried out on $F^2$ using SHELXL-Version 2018/3 to locate the remaining non-hydrogen atoms. Non-hydrogen atoms were refined with anisotropic displacement parameters. Hydrogen atoms attached to carbon atoms were fixed in calculated positions. All disorders were handled with SADI, SIMU, EADP, SAME and RIGU constraints and restraints. The crystallographic parameters of each structure are given bellow. All structures are deposited to CCDC and their corresponding deposition numbers are listed below:

**4a**[$BPh_4$] = 2119886; **4b**[OTf] = 2119887; **5a**[$BPh_4$] = 2119888; **5b**[$PF_6$] = 2119889; **6a**[$BPh_4$] = 2119890; **7a**[$BF_4$]$_2$ = 2119891; **7b**[$NTf_2$]$_2$ = 2119892; **8** = 2119893; and **9**[$BF_4$] = 2119894.

### 2.2 XRD structure of complex 8

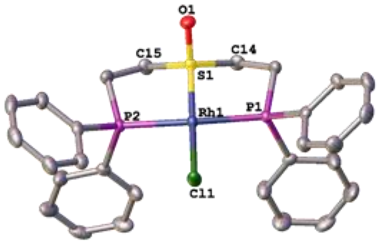


**Figure S17**: XRD structure of complex **8**. Hydrogen atoms are omitted for clarity. Selected bond lengths (Å): Rh1−S1 2.135(1), Rh1−Cl1 2.369(1), Rh1−P2 2.314(1), Rh1−P1 2.312(1), S1−O1 1.474(2), S1−C14 1.800(3), S1−C15 1.799(3). Selected bond angles (°): S1−Rh1−Cl1 172.4(1), S1−Rh1−P2 84.4(1), S1−Rh1−P1 84.2(1), P2−Rh1−Cl1 96.8(1), P1−Rh1−Cl1 96.4(1), P1−Rh1−P2 161.2(1), O1−S1−Rh1 118.2(1), O1−S1−C14 107.2(2), O1−S1−C15 106.7(2), C14−S1−Rh1 109.5(1), C15−S1−Rh1 110.1(1), C15−S1−C14 104.3(2).

## 2.3 Crystallographic parameter tables:

| | **4a**[$BPh_4$] | **4b**[OTf] | **5a**[$BPh_4$] | **5b**[$PF_6$] | **6a**[$BPh_4$] |
|---|---|---|---|---|---|
| Empirical formula | $C_{54.5}H_{53}BClP_2S$ | $C_{43}H_{32}F_4O_3P_2S_2$ | $C_{54}H_{53}BClP_2RhS$ | $C_{45.5}H_{36}ClF_7P_3RhS$ | $C_{56}H_{59}BP_2PtS$ |
| Formula weight | 848.23 | 798.74 | 945.13 | 979.07 | 1031.93 |
| Wavelength (Å) | 0.71073 | 0.71073 | 0.71073 | 0.71073 | 0.71073 |
| Temperature (K) | 293(2) | 293(2) | 150 (2) | 150 (2) | 293(2) |
| Crystal system | triclinic | monoclinic | monoclinic | Triclinic | monoclinic |
| Space group | *P-1* | *P21/n* | *P21/n* | *P-1* | *P21/c* |
| a/Å | 8.8424(5) | 12.4956(2) | 10.9915(4) | 9.1404(2) | 16.6775(2) |
| b/Å | 11.4067(6) | 28.0310(4) | 34.1432(10) | 12.3384(3) | 15.3273(2) |
| c/Å | 24.8662(10) | 12.6611(2) | 12.5533(4) | 19.4965(5) | 19.3126(3) |
| α/degree | 81.957(4) | 90.00 | 90 | 102.063(2) | 90.00 |
| β/degree | 80.897(4) | 119.324(2) | 99.760(3) | 92.077(2) | 102.4960(10) |
| γ/degree | 69.046(5) | 90.00 | 90 | 103.656(2) | 90.00 |
| Volume ($Å^3$) | 2303.2(2) | 3866.48(12) | 4642.9(3) | 2080.94(9) | 4819.76(12) |
| Z | 2 | 4 | 4 | 2 | 4 |
| $D_{calcd}$, g $cm^{-3}$ | 1.223 | 1.372 | 1.352 | 1.563 | 1.422 |
| $\mu/mm^{-1}$ | 0.234 | 0.280 | 0.576 | 0.705 | 3.057 |
| F(000) | 896.0 | 1648.0 | 1960.0 | 990.0 | 2096.0 |
| 2θ range (degree) | 3.332 to 50 | 4.012 to 49.998 | 4.066 to 50 | 4.29 to 50 | 3.65 to 50 |
| | $-10 \leq h \leq 10$ | $-14 \leq h \leq 14$ | $-12 \leq h \leq 13$ | $-10 \leq h \leq 10$ | $-19 \leq h \leq 19$ |
| Limiting Indices | $-13 \leq k \leq 13$ | $-33 \leq k \leq 33$ | $-38 \leq k \leq 40$ | $-14 \leq k \leq 14$ | $-18 \leq k \leq 18$ |
| | $-29 \leq l \leq 29$ | $-14 \leq l \leq 15$ | $-14 \leq l \leq 14$ | $-23 \leq l \leq 23$ | $-22 \leq l \leq 22$ |
| Total/ unique no. of reflns | 20848 / 8109 | 84590 / 6794 | 36105/ 8148 | 31769/ 7313 | 83399/ 8484 |
| $R_{int}$ | 0.0986 | 0.0628 | 0.0842 | 0.0347 | 0.0328 |
| Data / restr./params. | 8109/0/467 | 6794/186/458 | 8148/0/518 | 7313/154/580 | 8484/1/552 |
| GOF ($F^2$) | 1.062 | 1.987 | 1.305 | 1.025 | 1.045 |
| $R_1$, $wR_2$ | 0.1202, 0.2962 | 0.1103, 0.3948 | 0.1003, 0.2126 | 0.0308, 0.0739 | 0.0177, 0.0401 |
| R indices (all data) $R_1$, $wR_2$ | 0.1790, 0.3438 | 0.1156, 0.4059 | 0.1215, 0.2204 | 0.0367, 0.0764 | 0.0216, 0.0412 |
| Largest different peak and hole (e $Å^{-3}$) | 0.73, –0.48 | 1.07, –1.34 | 1.01, –1.67 | 1.12, –0.50 | 0.65/-0.39 |

| | **7a**[$BF_4$]$_2$ | **7b**[$NTf_2$]$_2$ | **8** | **7b**[$NTf_2$]$_2$ |
|---|---|---|---|---|
| Empirical formula | $C_{32}H_{39}B_2F_8NO_2P_2PtS$ | $C_{47}H_{35}F_{13}N_2O_8P_2PtS_5$ | $C_{28}H_{28}ClOP_2RhS$ | $C_{29.25}H_{31.5}BCl_{0.5}F_4P_2PtS$ |
| Formula weight | 932.35 | 1420.10 | 612.86 | 776.67 |
| Wavelength (Å) | 0.71073 | 0.71073 | 0.71073 | 0.71073 |
| Temperature (K) | 293(2) | 150 (2) | 150 (2) | 293(2) |
| Crystal system | triclinic | monoclinic | orthorhombic | orthorhombic |
| Space group | *P-1* | *P21/c* | *P212121* | *Pbam* |
| a/Å | 10.7270(2) | 23.6369(3) | 10.4581(2) | 20.9374(6) |
| b/Å | 13.1882(2) | 12.7303(2) | 14.2792(3) | 10.9959(3) |
| c/Å | 15.1430(2) | 18.9449(2) | 17.4031(4) | 14.9555(5) |
| α/degree | 65.687(2) | 90 | 90 | 90 |
| β/degree | 75.1110(10) | 98.2970(10) | 90 | 90 |
| γ/degree | 75.5460(10) | 90 | 90 | 90 |
| Volume (Å$^3$) | 1861.41(6) | 5640.95(13) | 2598.86(9) | 3443.14(18) |
| Z | 2 | 4 | 4 | 4 |
| $D_{calcd}$, g cm$^{-3}$ | 1.663 | 1.672 | 1.566 | 1.498 |
| μ/mm$^{-1}$ | 3.981 | 2.823 | 0.984 | 4.305 |
| F(000) | 920.0 | 2800.0 | 1248.0 | 1522.0 |
| 2θ range (degree) | 3.982 to 50 | 3.868 to 49.996 | 4.544 to 49.994 | 4.992 to 64.214 |
| | $-12 \le h \le 12$ | $-28 \le h \le 28$ | $-11 \le h \le 12$ | $-24 \le h \le 30$ |
| Limiting Indices | $-15 \le k \le 15$ | $-15 \le k \le 15$ | $-16 \le k \le 16$ | $-13 \le k \le 14$ |
| | $-17 \le l \le 17$ | $-22 \le l \le 21$ | $-20 \le l \le 17$ | $-15 \le l \le 19$ |
| Total/ unique no. of reflns | 25834/ 6546 | 92765/9935 | 21884/4575 | 17185/4698 |
| Rint | 0.0281 | 0.0500 | 0.0291 | 0.0391 |
| Data / restr./params. | 6546/17/393 | 9935/9/793 | 4575/0/307 | 4698/2/225 |
| GOF (F$^2$) | 1.049 | 1.033 | 1.044 | 1.083 |
| $R_1$, $wR_2$ | 0.0286, 0.0722 | 0.0346, 0.0897 | 0.0191, 0.0449 | 0.0454, 0.1063 |
| R indices (all data) $R_1$, $wR_2$ | 0.0323, 0.0738 | 0.0385, 0.0923 | 0.0207, 0.0455 | 0.0652, 0.1137 |
| Largest different peak and hole (e Å$^{-3}$) | 1.51, -0.83 | 1.17, -0.68 | 0.35, -0.28 | 1.52, -0.96 |

## 3. Computational details:

### 3.1. Geometry optimizations:

All molecular models were created using GaussView 6.0[10] and all optimization and frequency calculations were performed using Gaussian 16 program[11] with ωB97XD[12] functional and def2TZVP[13] basis set, def2-ECP was used for Rh and Pt metals. Frequency calculations were performed on the optimized geometries to confirm that they are local minimum in the potential energy surface. The coordinates of the optimized geometries are given in **section 3.9**. Bond Dissociation Energies (BDE) for the simplified monodentate complexes were calculated thermal free energies of the model complexes and the optimized ligand and metal fragments. The BDE values are given in **table S1**.

### 3.2. General details of ALMO-EDA calculations:

All ALMO-EDA[14] calculations and deformation density plots were calculated using Q-chem 5.4[15] program with ωB97X[16] functional and def2TZVP[13] basis set, def2-ECP was used for Rh and Pt metals. Deformation density plots were visualized using IQmol 2.15.3 program[17]. The results of the ALMO-EDA analysis are given below.

### 3.3. ALMO-EDA analysis of model pincer complexes 10a-16b:

The results EDA results from ALMO-EDA calculations for the model complexes **10a-16b** are given in the following table.

**Table S1**: EDA results from ALMO-EDA calculations for the model complexes **10a-16b** (all energies are given in kcal/mol).

| Entry | Model complex | BDE | $\Delta E_{int}$ | $\Delta E_{orb}$ | $\Delta E_{elstat}$ | $\Delta E_{Pauli}$ | $\Delta E_{disp}$ |
|---|---|---|---|---|---|---|---|
| 1 | **10a** | -38.57 | -38.88 | -86.04 | -73.75 | 135.11 | -14.20 |
| 2 | **10b** | -36.96 | -36.38 | -79.21 | -80.56 | 141.81 | -18.43 |
| 3 | **11a** | -36.21 | -37.96 | -54.94 | -85.13 | 116.01 | -13.90 |
| 4 | **11b** | -38.19 | -38.00 | -56.18 | -87.45 | 121.81 | -16.17 |
| 5 | **12a** | -39.82 | -41.23 | -69.41 | -95.82 | 138.71 | -14.71 |
| 6 | **12b** | -44.55 | -43.28 | -75.80 | -105.34 | 155.19 | -17.34 |
| 7 | **13a** | -70.06 | -58.01 | -83.95 | -150.52 | 193.26 | -16.80 |
| 8 | **13b** | -57.77 | -56.80 | -83.83 | -144.20 | 191.32 | -20.10 |
| 9 | **14a** | -56.76 | -57.99 | -100.38 | -125.23 | 181.39 | -13.77 |
| 10 | **14b** | -53.16 | -53.26 | -94.00 | -116.92 | 174.61 | -16.96 |
| 11 | **14c** | -51.35 | -49.12 | -86.58 | -124.54 | 181.48 | -19.47 |
| 12 | **15a** | -30.55 | -30.11 | -62.61 | -64.66 | 108.55 | -11.40 |
| 13 | **15b** | -57.57 | -58.76 | -118.80 | -80.65 | 154.40 | -13.72 |
| 14 | **15c** | -53.16 | -55.73 | -103.95 | -115.24 | 184.08 | -20.61 |
| 15 | **16a** | +48.60 | 45.47 | -58.63 | 25.85 | 89.26 | -11.01 |
| 16 | **16b** | +30.66 | 31.00 | -57.45 | 10.92 | 92.73 | -15.20 |

## 3.4. ETS-NOCV analysis results from ALMO-EDA calculations for model complexes 10a-18b.

The ETS-NOCV results from ALMO-EDA calculations for the model complexes **10a-18b** are given below (**Table S2-S21**). Each row in the tables corresponds to the relevant NOCV pair, eigenvalue of the deformation density and the orbital interaction energy ($\Delta E_{orb}$) of the corresponding interaction. Deformation densities with the highest orbital interaction energies ($\Delta E_{orb}$ > 2.5 kcal/mol) in **Tables S2-S21** are highlighted in bold. The plots of selected deformation densities were visualized (complexes with monodentate ligands in **Table S22**, complexes with pincer ligands in **Table S23**). In model complexes **10a**–**16b**, making two fragments involves breaking only one bond between the metal atom and the donor atom of the ligand. However, in model pincer complexes **17a-18b** (shown in **Table S22**), three bonds are broken during such fragmentation. Therefore, all highest energy interactions in the complexes **10a-16b** of the monodentate ligands (reported in **Table 1** of the main text) are the interactions between the donor atom and the metal atom. On the other hand, in the model pincer complexes **17a-18b**, the interaction energies between the phosphine arms and the metal center are also significant. Hence, deformation density plots of all highest energy interactions of the pincer complexes (shown in **Table S23**) were carefully inspected, in order to discriminate the σ- and π-interaction of the metal with the central donor atom from those with the flanking phosphine arms. Only the energies corresponding to the first category (the highlighted deformation density plots in **Table S23**) are those reported in **Table 2** of the main text.

All energies in the following tables are in kcal/mol.

…………………………………………………………………………………………………………………………………..

**Table S2**: $[(SMe_3)(PH_3)_2RhCl]^+$, **10a**

| NOCV pairs | Eigenvalue ($|\upsilon_n|$) | $\Delta E_{orb(n)}$ |
|---|---|---|
| **1 -> 371** | **0.483** | **-22.888** |
| **2 -> 370** | **0.442** | **-32.2558** |
| **3 -> 369** | **0.413** | **-21.0389** |
| **4 -> 368** | **0.145** | **-4.54829** |
| 5 -> 367 | 0.085 | -1.11228 |
| 6 -> 366 | 0.071 | -1.3517 |
| 7 -> 365 | 0.062 | -0.9377 |
| 8 -> 364 | 0.051 | -0.60074 |
| 9 -> 363 | 0.046 | -0.51722 |
| 10 -> 362 | 0.045 | -0.3617 |
| 11 -> 361 | 0.041 | -0.44695 |
| 12 -> 360 | 0.034 | -0.1825 |
| 13 -> 359 | 0.03 | -0.14417 |
| 14 -> 358 | 0.029 | -0.26093 |
| 15 -> 357 | 0.022 | -0.15586 |
| 16 -> 356 | 0.022 | -0.138 |
| 17 -> 355 | 0.02 | -0.04464 |
| 18 -> 354 | 0.019 | -0.12286 |
| 19 -> 353 | 0.018 | -0.05047 |
| 20 -> 352 | 0.015 | -0.03079 |
| 21 -> 351 | 0.012 | -0.05374 |
| 22 -> 350 | 0.012 | -0.02275 |
| 23 -> 349 | 0.011 | -0.03451 |
| 24 -> 348 | 0.011 | -0.01258 |
| 25 -> 347 | 0.01 | -0.03101 |

**Table S3**: $[(SPh_3)(PH_3)_2RhCl]^+$, **10b**

| NOCV pairs | Eigenvalue ($|\upsilon_n|$) | $\Delta E_{orb(n)}$ |
|---|---|---|
| **1 -> 872** | **0.47** | **-21.2994** |
| **2 -> 871** | **0.441** | **-31.1321** |
| **3 -> 870** | **0.389** | **-18.1002** |
| **4 -> 869** | **0.138** | **-4.06699** |
| 5 -> 868 | 0.088 | -1.03879 |
| 6 -> 867 | 0.067 | -0.97078 |
| 7 -> 866 | 0.057 | -0.59784 |
| 8 -> 865 | 0.051 | -0.64094 |

| | | |
|---|---|---|
| 9 -> 864 | 0.047 | -0.54994 |
| 10 -> 863 | 0.043 | -0.40006 |
| 11 -> 862 | 0.037 | -0.22865 |
| 12 -> 861 | 0.035 | -0.18857 |
| 13 -> 860 | 0.031 | -0.25474 |
| 14 -> 859 | 0.028 | -0.13421 |
| 15 -> 858 | 0.027 | -0.15002 |
| 16 -> 857 | 0.025 | -0.13596 |
| 17 -> 856 | 0.021 | -0.13354 |
| 18 -> 855 | 0.021 | -0.08458 |
| 19 -> 854 | 0.019 | -0.08117 |
| 20 -> 853 | 0.018 | -0.05546 |
| 21 -> 852 | 0.016 | -0.05767 |
| 22 -> 851 | 0.015 | -0.04757 |
| 23 -> 850 | 0.013 | -0.03067 |
| 24 -> 849 | 0.013 | -0.05426 |
| 25 -> 848 | 0.011 | -0.03511 |
| 26 -> 847 | 0.011 | -0.04457 |
| 27 -> 846 | 0.01 | -0.02359 |

**Table S4**: [($SMe_2$)($PH_3$)$_2$RhCl], **11a**

| NOCV pairs | Eigenvalue ($\|\upsilon_n\|$) | $\Delta E_{orb(n)}$ |
|---|---|---|
| **1 -> 322** | **0.515** | **-35.0343** |
| **2 -> 321** | **0.278** | **-8.79744** |
| **3 -> 320** | **0.204** | **-6.09343** |
| **4 -> 319** | **0.123** | **-3.40781** |
| 5 -> 318 | 0.058 | -0.62537 |
| 6 -> 317 | 0.05 | -0.46682 |
| 7 -> 316 | 0.041 | -0.31994 |
| 8 -> 315 | 0.034 | -0.23309 |
| 9 -> 314 | 0.033 | -0.24929 |
| 10 -> 313 | 0.028 | -0.12065 |
| 11 -> 312 | 0.027 | -0.12907 |
| 12 -> 311 | 0.019 | -0.0925 |
| 13 -> 310 | 0.019 | -0.07404 |
| 14 -> 309 | 0.017 | -0.08474 |
| 15 -> 308 | 0.017 | -0.05222 |
| 16 -> 307 | 0.014 | -0.03331 |
| 17 -> 306 | 0.012 | -0.02938 |
| 18 -> 305 | 0.012 | -0.01889 |
| 19 -> 304 | 0.011 | -0.0251 |

**Table S5**: [($SPh_2$)($PH_3$)$_2$RhCl], **11b**

| NOCV pairs | Eigenvalue ($\|\upsilon_n\|$) | $\Delta E_{orb(n)}$ |
|---|---|---|
| **1 -> 656** | **0.502** | **-34.3907** |
| **2 -> 655** | **0.282** | **-9.2015** |
| **3 -> 654** | **0.227** | **-6.90653** |
| **4 -> 653** | **0.13** | **-3.4416** |
| 5 -> 652 | 0.061 | -0.62894 |
| 6 -> 651 | 0.059 | -0.51667 |
| 7 -> 650 | 0.055 | -0.40061 |
| 8 -> 649 | 0.04 | -0.2735 |
| 9 -> 648 | 0.033 | -0.2861 |
| 10 -> 647 | 0.03 | -0.13598 |
| 11 -> 646 | 0.03 | -0.1801 |
| 12 -> 645 | 0.028 | -0.17693 |
| 13 -> 644 | 0.025 | -0.11597 |
| 14 -> 643 | 0.024 | -0.12672 |
| 15 -> 642 | 0.02 | -0.07123 |
| 16 -> 641 | 0.018 | -0.07169 |
| 17 -> 640 | 0.016 | -0.05282 |
| 18 -> 639 | 0.014 | -0.04512 |
| 19 -> 638 | 0.013 | -0.03108 |
| 20 -> 637 | 0.012 | -0.02827 |

| | | |
|---|---|---|
| 21 -> 636 | 0.011 | -0.02582 |
| 22 -> 635 | 0.01 | -0.04097 |

**Table S6**: [(DMSO)($PH_3$)$_2$RhCl], **12a**

| NOCV pairs | Eigenvalue ($\|\upsilon_n\|$) | $\Delta E_{orb(n)}$ |
|---|---|---|
| **1 -> 353** | **0.556** | **-39.8206** |
| **2 -> 352** | **0.344** | **-13.005** |
| **3 -> 351** | **0.303** | **-11.4101** |
| **4 -> 350** | **0.112** | **-3.5472** |
| 5 -> 349 | 0.054 | -0.57684 |
| 6 -> 348 | 0.041 | -0.38652 |
| 7 -> 347 | 0.039 | -0.39053 |
| 8 -> 346 | 0.035 | -0.29374 |
| 9 -> 345 | 0.032 | -0.26928 |
| 10 -> 344 | 0.028 | -0.14378 |
| 11 -> 343 | 0.025 | -0.16414 |
| 12 -> 342 | 0.023 | -0.17302 |
| 13 -> 341 | 0.022 | -0.10788 |
| 14 -> 340 | 0.02 | -0.19195 |
| 15 -> 339 | 0.019 | -0.06775 |
| 16 -> 338 | 0.018 | -0.06876 |
| 17 -> 337 | 0.015 | -0.06492 |
| 18 -> 336 | 0.015 | -0.0463 |
| 19 -> 335 | 0.014 | -0.03703 |
| 20 -> 334 | 0.011 | -0.02352 |

**Table S7**: [($Ph_2SO$)($PH_3$)$_2$RhCl], **12b**

| NOCV pairs | Eigenvalue ($\|\upsilon_n\|$) | $\Delta E_{orb(n)}$ |
|---|---|---|
| **1 -> 687** | **0.547** | **-40.7343** |
| **2 -> 686** | **0.389** | **-15.9253** |
| **3 -> 685** | **0.318** | **-12.9919** |
| **4 -> 684** | **0.119** | **-3.57595** |
| 5 -> 683 | 0.062 | -0.61553 |
| 6 -> 682 | 0.058 | -0.51965 |
| 7 -> 681 | 0.05 | -0.43872 |
| 8 -> 680 | 0.041 | -0.34486 |
| 9 -> 679 | 0.037 | -0.30842 |
| 10 -> 678 | 0.031 | -0.24833 |
| 11 -> 677 | 0.03 | -0.24041 |
| 12 -> 676 | 0.028 | -0.13524 |
| 13 -> 675 | 0.027 | -0.32239 |
| 14 -> 674 | 0.024 | -0.11978 |
| 15 -> 673 | 0.021 | -0.11618 |
| 16 -> 672 | 0.021 | -0.12355 |
| 17 -> 671 | 0.018 | -0.09854 |
| 18 -> 670 | 0.017 | -0.0426 |
| 19 -> 669 | 0.016 | -0.06586 |
| 20 -> 668 | 0.015 | -0.04783 |
| 21 -> 667 | 0.013 | -0.04934 |
| 22 -> 666 | 0.013 | -0.03722 |
| 23 -> 665 | 0.012 | -0.03991 |
| 24 -> 664 | 0.011 | -0.03648 |

**Table S8**: [($PMe_3$)($PH_3$)$_2$RhCl], **13a**

| NOCV pairs | Eigenvalue ($\|\upsilon_n\|$) | $\Delta E_{orb(n)}$ |
|---|---|---|
| **1 -> 371** | **0.633** | **-56.6936** |
| **2 -> 370** | **0.323** | **-11.6232** |
| **3 -> 369** | **0.278** | **-10.4428** |
| **4 -> 368** | **0.13** | **-3.93** |
| 5 -> 367 | 0.052 | -0.5357 |
| 6 -> 366 | 0.047 | -0.44414 |
| 7 -> 365 | 0.036 | -0.26834 |
| 8 -> 364 | 0.032 | -0.22834 |
| 9 -> 363 | 0.03 | -0.08863 |

| | | |
|---|---|---|
| 10 -> 362 | 0.028 | -0.17419 |
| 11 -> 361 | 0.025 | -0.11486 |
| 12 -> 360 | 0.023 | -0.0973 |
| 13 -> 359 | 0.021 | -0.0959 |
| 14 -> 358 | 0.019 | -0.12449 |
| 15 -> 357 | 0.018 | -0.08825 |
| 16 -> 356 | 0.016 | -0.04632 |
| 17 -> 355 | 0.014 | -0.0379 |
| 18 -> 354 | 0.013 | -0.03677 |
| 19 -> 353 | 0.013 | -0.0312 |
| 20 -> 352 | 0.013 | -0.04399 |
| 21 -> 351 | 0.011 | -0.04769 |
| 22 -> 350 | 0.011 | -0.01363 |

**Table S9**: [$(PPh_3)(PH_3)_2RhCl$], **13b**

| NOCV pairs | Eigenvalue ($|\upsilon_n|$) | $\Delta E_{orb(n)}$ |
|---|---|---|
| **1 -> 872** | **0.62** | **-53.3144** |
| **2 -> 871** | **0.348** | **-13.0266** |
| **3 -> 870** | **0.294** | **-11.1584** |
| **4 -> 869** | **0.13** | **-3.76034** |
| 5 -> 868 | 0.065 | -0.74686 |
| 6 -> 867 | 0.061 | -0.57502 |
| 7 -> 866 | 0.049 | -0.38122 |
| 8 -> 865 | 0.041 | -0.32729 |
| 9 -> 864 | 0.038 | -0.17563 |
| 10 -> 863 | 0.032 | -0.2579 |
| 11 -> 862 | 0.029 | -0.24631 |
| 12 -> 861 | 0.029 | -0.17256 |
| 13 -> 860 | 0.027 | -0.12845 |
| 14 -> 859 | 0.026 | -0.1247 |
| 15 -> 858 | 0.022 | -0.09566 |
| 16 -> 857 | 0.02 | -0.10673 |
| 17 -> 856 | 0.019 | -0.09626 |
| 18 -> 855 | 0.019 | -0.0829 |
| 19 -> 854 | 0.017 | -0.06175 |
| 20 -> 853 | 0.016 | -0.03914 |
| 21 -> 852 | 0.015 | -0.05381 |
| 22 -> 851 | 0.013 | -0.04728 |
| 23 -> 850 | 0.012 | -0.03912 |
| 24 -> 849 | 0.012 | -0.03619 |
| 25 -> 848 | 0.011 | -0.05906 |

**Table S10**: [$(PF_3)(PH_3)_2RhCl$], **14a**

| NOCV pairs | Eigenvalue ($|\upsilon_n|$) | $\Delta E_{orb(n)}$ |
|---|---|---|
| **1 -> 317** | **0.582** | **-51.8275** |
| **2 -> 316** | **0.466** | **-23.5887** |
| **3 -> 315** | **0.385** | **-20.0155** |
| **4 -> 314** | **0.117** | **-3.8987** |
| 5 -> 313 | 0.058 | -0.57497 |
| 6 -> 312 | 0.048 | -0.4679 |
| 7 -> 311 | 0.032 | -0.10034 |
| 8 -> 310 | 0.031 | -0.57374 |
| 9 -> 309 | 0.026 | -0.16001 |
| 10 -> 308 | 0.024 | -0.13181 |
| 11 -> 307 | 0.023 | -0.16577 |
| 12 -> 306 | 0.02 | -0.057 |
| 13 -> 305 | 0.018 | -0.07956 |
| 14 -> 304 | 0.017 | -0.0576 |
| 15 -> 303 | 0.015 | -0.0829 |
| 16 -> 302 | 0.014 | -0.05419 |
| 17 -> 301 | 0.013 | -0.0474 |
| 18 -> 300 | 0.013 | -0.03953 |
| 19 -> 299 | 0.012 | -0.04922 |
| 20 -> 298 | 0.011 | -0.03379 |

**Table S11**: [(P($CF_3$)$_3$)($PH_3$)$_2$RhCl], **14b**

| NOCV pairs | Eigenvalue ($|\upsilon_n|$) | $\Delta E_{orb(n)}$ |
|---|---|---|
| **1 -> 596** | **0.557** | **-46.8873** |
| **2 -> 595** | **0.465** | **-21.7612** |
| **3 -> 594** | **0.398** | **-18.9917** |
| **4 -> 593** | **0.13** | **-4.00109** |
| 5 -> 592 | 0.072 | -0.76841 |
| 6 -> 591 | 0.053 | -0.56777 |
| 7 -> 590 | 0.037 | -0.36535 |
| 8 -> 589 | 0.032 | -0.26484 |
| 9 -> 588 | 0.031 | -0.22202 |
| 10 -> 587 | 0.031 | -0.18706 |
| 11 -> 586 | 0.028 | -0.15391 |
| 12 -> 585 | 0.028 | -0.17438 |
| 13 -> 584 | 0.025 | -0.16387 |
| 14 -> 583 | 0.025 | -0.18494 |
| 15 -> 582 | 0.022 | -0.18852 |
| 16 -> 581 | 0.02 | -0.11386 |
| 17 -> 580 | 0.018 | -0.09744 |
| 18 -> 579 | 0.016 | -0.08124 |
| 19 -> 578 | 0.015 | -0.05035 |
| 20 -> 577 | 0.013 | -0.06187 |
| 21 -> 576 | 0.013 | -0.0383 |
| 22 -> 575 | 0.012 | -0.04176 |
| 23 -> 574 | 0.011 | -0.02959 |
| 24 -> 573 | 0.011 | -0.03667 |

**Table S12**: [(P($C_6F_5$)$_3$)($PH_3$)$_2$RhCl], **14c**

| NOCV pairs | Eigenvalue ($|\upsilon_n|$) | $\Delta E_{orb(n)}$ |
|---|---|---|
| **1 -> 1247** | **0.577** | **-47.7922** |
| **2 -> 1246** | **0.392** | **-16.6418** |
| **3 -> 1245** | **0.365** | **-15.5711** |
| **4 -> 1244** | **0.147** | **-3.92066** |
| 5 -> 1243 | 0.065 | -0.65746 |
| 6 -> 1242 | 0.058 | -0.63401 |
| 7 -> 1241 | 0.047 | -0.45833 |
| 8 -> 1240 | 0.04 | -0.35695 |
| 9 -> 1239 | 0.036 | -0.27682 |
| 10 -> 1238 | 0.032 | -0.20623 |
| 11 -> 1237 | 0.029 | -0.17244 |
| 12 -> 1236 | 0.028 | -0.17942 |
| 13 -> 1235 | 0.027 | -0.18466 |
| 14 -> 1234 | 0.025 | -0.21926 |
| 15 -> 1233 | 0.023 | -0.16944 |
| 16 -> 1232 | 0.02 | -0.10277 |
| 17 -> 1231 | 0.018 | -0.0833 |
| 18 -> 1230 | 0.017 | -0.07706 |
| 19 -> 1229 | 0.017 | -0.07718 |
| 20 -> 1228 | 0.016 | -0.06574 |
| 21 -> 1227 | 0.015 | -0.06494 |
| 22 -> 1226 | 0.015 | -0.0613 |
| 23 -> 1225 | 0.012 | -0.04246 |
| 24 -> 1224 | 0.012 | -0.05503 |
| 25 -> 1223 | 0.011 | -0.04056 |
| 26 -> 1222 | 0.01 | -0.04327 |

**Table S13**: [(NHN)($PH_3$)$_2$RhCl]$^+$, **15a**

| NOCV pairs | Eigenvalue ($|\upsilon_n|$) | $\Delta E_{orb(n)}$ |
|---|---|---|
| **1 -> 366** | **0.673** | **-21.3717** |
| **2 -> 365** | **0.382** | **-25.3039** |
| **3 -> 364** | **0.215** | **-7.82381** |
| **4 -> 363** | **0.127** | **-3.96461** |
| 5 -> 362 | 0.071 | -0.67709 |
| 6 -> 361 | 0.067 | -0.85778 |

| | | |
|---|---|---|
| 7 -> 360 | 0.06 | -0.92374 |
| 8 -> 359 | 0.05 | -0.51031 |
| 9 -> 358 | 0.046 | -0.35122 |
| 10 -> 357 | 0.043 | -0.37939 |
| 11 -> 356 | 0.036 | -0.36331 |
| 12 -> 355 | 0.03 | -0.22442 |
| 13 -> 354 | 0.028 | -0.18314 |
| 14 -> 353 | 0.023 | -0.14244 |
| 15 -> 352 | 0.021 | -0.08729 |
| 16 -> 351 | 0.018 | -0.08244 |
| 17 -> 350 | 0.014 | -0.0804 |
| 18 -> 349 | 0.013 | -0.03341 |
| 19 -> 348 | 0.013 | -0.03281 |
| 20 -> 347 | 0.012 | -0.01574 |
| 21 -> 346 | 0.01 | -0.01639 |
| 22 -> 345 | 0.01 | -0.01997 |

**Table S14**: $[(NHP)(PH_3)_2RhCl]^+$, **15b**

| NOCV pairs | Eigenvalue ($|\upsilon_n|$) | $\Delta E_{orb(n)}$ |
|---|---|---|
| **1 -> 372** | **0.676** | **-34.8235** |
| **2 -> 371** | **0.535** | **-42.9273** |
| **3 -> 370** | **0.437** | **-27.5978** |
| **4 -> 369** | **0.154** | **-5.99222** |
| 5 -> 368 | 0.1 | -1.46431 |
| 6 -> 367 | 0.087 | -1.93637 |
| 7 -> 366 | 0.077 | -0.72036 |
| 8 -> 365 | 0.069 | -1.16698 |
| 9 -> 364 | 0.062 | -0.98693 |
| 10 -> 363 | 0.043 | -0.46493 |
| 11 -> 362 | 0.043 | -0.46949 |
| 12 -> 361 | 0.039 | -0.41004 |
| 13 -> 360 | 0.035 | -0.22385 |
| 14 -> 359 | 0.03 | -0.10188 |
| 15 -> 358 | 0.03 | -0.20417 |
| 16 -> 357 | 0.021 | -0.03684 |
| 17 -> 356 | 0.02 | -0.04188 |
| 18 -> 355 | 0.019 | -0.02707 |
| 19 -> 354 | 0.018 | -0.02964 |
| 20 -> 353 | 0.017 | -0.03833 |
| 21 -> 352 | 0.015 | -0.02518 |
| 22 -> 351 | 0.013 | -0.02993 |
| 23 -> 350 | 0.013 | -0.09566 |
| 24 -> 349 | 0.013 | -0.04572 |
| 25 -> 348 | 0.011 | -0.03269 |
| 26 -> 347 | 0.011 | -0.03744 |

**Table S15**: $[(PR_3)(PH_3)_2RhCl]^{3+}$, **15c**

| NOCV pairs | Eigenvalue ($|\upsilon_n|$) | $\Delta E_{orb(n)}$ |
|---|---|---|
| **1 -> 1277** | **0.54** | **-44.4321** |
| **2 -> 1276** | **0.497** | **-25.301** |
| **3 -> 1275** | **0.431** | **-20.8725** |
| **4 -> 1274** | **0.171** | **-5.63568** |
| 5 -> 1273 | 0.103 | -1.41199 |
| 6 -> 1272 | 0.084 | -1.60639 |
| 7 -> 1271 | 0.07 | -1.0896 |
| 8 -> 1270 | 0.066 | -0.75653 |
| 9 -> 1269 | 0.06 | -0.84893 |
| 10 -> 1268 | 0.056 | -0.84312 |
| 11 -> 1267 | 0.049 | -0.6354 |
| 12 -> 1266 | 0.046 | -0.4927 |
| 13 -> 1265 | 0.043 | -0.40807 |
| 14 -> 1264 | 0.034 | -0.18554 |
| 15 -> 1263 | 0.032 | -0.18566 |
| 16 -> 1262 | 0.027 | -0.14657 |

| | | |
|---|---|---|
| 17 -> 1261 | 0.023 | -0.09362 |
| 18 -> 1260 | 0.022 | -0.07822 |
| 19 -> 1259 | 0.019 | -0.05921 |
| 20 -> 1258 | 0.018 | -0.06552 |
| 21 -> 1257 | 0.017 | -0.08004 |
| 22 -> 1256 | 0.016 | -0.07747 |
| 23 -> 1255 | 0.015 | -0.04092 |
| 24 -> 1254 | 0.014 | -0.03667 |
| 25 -> 1253 | 0.014 | -0.02479 |
| 26 -> 1252 | 0.013 | -0.03048 |
| 27 -> 1251 | 0.013 | -0.0445 |
| 28 -> 1250 | 0.012 | -0.04188 |
| 29 -> 1249 | 0.011 | -0.02815 |
| 30 -> 1248 | 0.011 | -0.02837 |
| 31 -> 1247 | 0.011 | -0.03086 |

**Table S16:** [(SMe$_3$)PtMe]$^{2+}$, **16a**

| NOCV pairs | Eigenvalue (\|$\upsilon_n$\|) | $\Delta E_{orb(n)}$ |
|---|---|---|
| **1 -> 383** | **0.401** | **-33.7422** |
| **2 -> 382** | **0.269** | **-8.57897** |
| **3 -> 381** | **0.255** | **-8.61754** |
| **4 -> 380** | **0.117** | **-2.98668** |
| 5 -> 379 | 0.058 | -0.86551 |
| 6 -> 378 | 0.055 | -0.79817 |
| 7 -> 377 | 0.047 | -0.50734 |
| 8 -> 376 | 0.045 | -0.48626 |
| 9 -> 375 | 0.043 | -0.49462 |
| 10 -> 374 | 0.039 | -0.38107 |
| 11 -> 373 | 0.038 | -0.36336 |
| 12 -> 372 | 0.033 | -0.31855 |
| 13 -> 371 | 0.026 | -0.28586 |
| 14 -> 370 | 0.024 | -0.1745 |
| 15 -> 369 | 0.022 | -0.12158 |
| 16 -> 368 | 0.021 | -0.0648 |
| 17 -> 367 | 0.019 | -0.0965 |
| 18 -> 366 | 0.018 | -0.09283 |
| 19 -> 365 | 0.017 | -0.07634 |
| 20 -> 364 | 0.015 | -0.06713 |
| 21 -> 363 | 0.014 | -0.07255 |
| 22 -> 362 | 0.014 | -0.05678 |
| 23 -> 361 | 0.013 | -0.05333 |
| 24 -> 360 | 0.011 | -0.03648 |

**Table S17**: [(SPh$_3$)PtMe]$^{2+}$, **16b**

| NOCV pairs | Eigenvalue (\|$\upsilon_n$\|) | $\Delta E_{orb(n)}$ |
|---|---|---|
| **1 -> 884** | **0.422** | **-32.8406** |
| **2 -> 883** | **0.239** | **-7.10914** |
| **3 -> 882** | **0.209** | **-6.42324** |
| **4 -> 881** | **0.115** | **-2.63633** |
| 5 -> 880 | 0.08 | -1.2755 |
| 6 -> 879 | 0.076 | -1.0993 |
| 7 -> 878 | 0.07 | -0.9593 |
| 8 -> 877 | 0.058 | -0.69593 |
| 9 -> 876 | 0.051 | -0.64102 |
| 10 -> 875 | 0.049 | -0.45998 |
| 11 -> 874 | 0.042 | -0.61147 |
| 12 -> 873 | 0.039 | -0.38542 |
| 13 -> 872 | 0.035 | -0.30014 |
| 14 -> 871 | 0.032 | -0.24353 |
| 15 -> 870 | 0.028 | -0.21758 |
| 16 -> 869 | 0.027 | -0.19831 |
| 17 -> 868 | 0.024 | -0.18473 |
| 18 -> 867 | 0.023 | -0.15245 |
| 19 -> 866 | 0.022 | -0.11772 |

| | | |
|---|---|---|
| 20 -> 865 | 0.021 | -0.12382 |
| 21 -> 864 | 0.02 | -0.12914 |
| 22 -> 863 | 0.02 | -0.0881 |
| 23 -> 862 | 0.019 | -0.09682 |
| 24 -> 861 | 0.019 | -0.0941 |
| 25 -> 860 | 0.019 | -0.07836 |
| 26 -> 859 | 0.018 | -0.08292 |
| 27 -> 858 | 0.017 | -0.07783 |
| 28 -> 857 | 0.017 | -0.06199 |
| 29 -> 856 | 0.017 | -0.05942 |
| 30 -> 855 | 0.016 | -0.0701 |
| 31 -> 854 | 0.015 | -0.05282 |
| 32 -> 853 | 0.014 | -0.05544 |
| 33 -> 852 | 0.014 | -0.04637 |
| 34 -> 851 | 0.013 | -0.04687 |
| 35 -> 850 | 0.012 | -0.04973 |
| 36 -> 849 | 0.012 | -0.04498 |
| 37 -> 848 | 0.011 | -0.03466 |
| 38 -> 847 | 0.011 | -0.04279 |
| 39 -> 846 | 0.011 | -0.05184 |
| 40 -> 845 | 0.01 | -0.04327 |

**Table S18**: $[(\text{Me-PSP})\text{RhCl}]^+$, **17a**

| NOCV pairs | Eigenvalue ($|\upsilon_n|$) | $\Delta E_{orb(n)}$ |
|---|---|---|
| **1 -> 433** | **1.167** | **-102.502** |
| **2 -> 432** | **0.588** | **-33.3825** |
| **3 -> 431** | **0.465** | **-29.9982** |
| **4 -> 430** | **0.332** | **-16.7944** |
| **5 -> 429** | **0.265** | **-19.911** |
| **6 -> 428** | **0.217** | **-25.7976** |
| **7 -> 427** | **0.207** | **-7.80713** |
| **8 -> 426** | **0.119** | **-5.5175** |

| | | |
|---|---|---|
| 9 -> 425 | 0.091 | -1.24735 |
| 10 -> 424 | 0.071 | -0.87367 |
| 11 -> 423 | 0.038 | -0.32443 |
| 12 -> 422 | 0.036 | -0.38011 |
| 13 -> 421 | 0.035 | -0.25358 |
| 14 -> 420 | 0.03 | -0.13826 |
| 15 -> 419 | 0.03 | -0.28195 |
| 16 -> 418 | 0.027 | -0.13421 |
| 17 -> 417 | 0.025 | -0.08412 |
| 18 -> 416 | 0.022 | -0.03912 |
| 19 -> 415 | 0.018 | -0.03264 |
| 20 -> 414 | 0.017 | -0.12274 |
| 21 -> 413 | 0.016 | -0.06353 |
| 22 -> 412 | 0.015 | -0.01788 |
| 23 -> 411 | 0.015 | 0.001872 |
| 24 -> 410 | 0.013 | -0.0059 |
| 25 -> 409 | 0.013 | -0.02734 |
| 26 -> 408 | 0.011 | -0.01692 |
| 27 -> 407 | 0.011 | -0.0151 |
| 28 -> 406 | 0.01 | -0.0251 |

**Table S19**: $[(\text{aromaticPh-PSP})\text{RhCl}]^+$, **17b**

| NOCV pairs | Eigenvalue ($|\upsilon_n|$) | $\Delta E_{orb(n)}$ |
|---|---|---|
| **1 -> 848** | **1.156** | **-99.5774** |
| **2 -> 847** | **0.542** | **-29.0778** |
| **3 -> 846** | **0.468** | **-30.0241** |
| **4 -> 845** | **0.338** | **-17.5962** |
| **5 -> 844** | **0.262** | **-18.1708** |
| **6 -> 843** | **0.221** | **-26.4948** |
| **7 -> 842** | **0.205** | **-8.00894** |
| **8 -> 841** | **0.118** | **-5.59308** |
| 9 -> 840 | 0.08 | -1.10971 |

| | | |
|---|---|---|
| 10 -> 839 | 0.072 | -0.88824 |
| 11 -> 838 | 0.039 | -0.3558 |
| 12 -> 837 | 0.037 | -0.32621 |
| 13 -> 836 | 0.035 | -0.31332 |
| 14 -> 835 | 0.031 | -0.20827 |
| 15 -> 834 | 0.028 | -0.11995 |
| 16 -> 833 | 0.028 | -0.18907 |
| 17 -> 832 | 0.025 | -0.08532 |
| 18 -> 831 | 0.022 | -0.07699 |
| 19 -> 830 | 0.021 | -0.02825 |
| 20 -> 829 | 0.02 | -0.04608 |
| 21 -> 828 | 0.018 | -0.08162 |
| 22 -> 827 | 0.017 | -0.04668 |
| 23 -> 826 | 0.017 | -0.09103 |
| 24 -> 825 | 0.015 | -0.06166 |
| 25 -> 824 | 0.014 | -0.0187 |
| 26 -> 823 | 0.013 | -0.04584 |
| 27 -> 822 | 0.011 | -0.03641 |

**Table S20**: [(Me-PSP)PtMe]$^{2+}$, **18a**

| NOCV pairs | Eigenvalue ($|\upsilon_n|$) | $\Delta E_{orb(n)}$ |
|---|---|---|
| **1 -> 445** | **1.072** | **-142.996** |
| **2 -> 444** | **0.399** | **-22.17** |
| **3 -> 443** | **0.369** | **-47.1645** |
| **4 -> 442** | **0.35** | **-17.5457** |
| **5 -> 441** | **0.312** | **-17.8781** |
| **6 -> 440** | **0.287** | **-15.3321** |
| **7 -> 439** | **0.235** | **-21.7597** |
| **8 -> 438** | **0.166** | **-10.3801** |
| 9 -> 437 | 0.089 | -2.23205 |
| 10 -> 436 | 0.088 | -1.76839 |
| 11 -> 435 | 0.072 | -1.43573 |
| 12 -> 434 | 0.063 | -0.70258 |
| 13 -> 433 | 0.053 | -0.35237 |
| 14 -> 432 | 0.053 | -0.54012 |
| 15 -> 431 | 0.042 | -0.60214 |
| 16 -> 430 | 0.04 | -0.62148 |
| 17 -> 429 | 0.04 | -0.41076 |
| 18 -> 428 | 0.038 | -0.29592 |
| 19 -> 427 | 0.037 | -0.41438 |
| 20 -> 426 | 0.034 | -0.34438 |
| 21 -> 425 | 0.033 | -0.3089 |
| 22 -> 424 | 0.03 | -0.31162 |
| 23 -> 423 | 0.029 | -0.25843 |
| 24 -> 422 | 0.028 | -0.18734 |
| 25 -> 421 | 0.028 | -0.1841 |
| 26 -> 420 | 0.027 | -0.18194 |
| 27 -> 419 | 0.024 | -0.14599 |
| 28 -> 418 | 0.023 | -0.17234 |
| 29 -> 417 | 0.015 | -0.04411 |
| 30 -> 416 | 0.015 | -0.06374 |
| 31 -> 415 | 0.015 | -0.06372 |
| 32 -> 414 | 0.011 | 0.004104 |

**Table S21**: [(aromaticPh-PSP)PtMe]$^{2+}$, **18b**

| NOCV pairs | Eigenvalue ($|\upsilon_n|$) | $\Delta E_{orb(n)}$ |
|---|---|---|
| **1 -> 860** | **1.077** | **-141.346** |
| **2 -> 859** | **0.387** | **-45.023** |
| **3 -> 858** | **0.354** | **-22.7034** |
| **4 -> 857** | **0.342** | **-18.8704** |
| **5 -> 856** | **0.306** | **-16.9536** |
| **6 -> 855** | **0.285** | **-15.5201** |
| **7 -> 854** | **0.247** | **-21.9136** |
| **8 -> 853** | **0.16** | **-9.86916** |

| | | |
|---|---|---|
| 9 -> 852 | 0.09 | -1.90375 |
| 10 -> 851 | 0.087 | -1.33541 |
| 11 -> 850 | 0.083 | -1.4735 |
| 12 -> 849 | 0.077 | -1.37455 |
| 13 -> 848 | 0.075 | -1.37494 |
| 14 -> 847 | 0.065 | -1.18411 |
| 15 -> 846 | 0.061 | -0.8267 |
| 16 -> 845 | 0.057 | -0.69043 |
| 17 -> 844 | 0.055 | -0.63746 |
| 18 -> 843 | 0.049 | -0.75062 |
| 19 -> 842 | 0.047 | -0.50297 |
| 20 -> 841 | 0.043 | -0.33451 |
| 21 -> 840 | 0.04 | -0.51814 |
| 22 -> 839 | 0.037 | -0.34997 |
| 23 -> 838 | 0.036 | -0.27924 |
| 24 -> 837 | 0.033 | -0.33835 |
| 25 -> 836 | 0.032 | -0.34255 |
| 26 -> 835 | 0.03 | -0.19577 |
| 27 -> 834 | 0.028 | -0.19056 |
| 28 -> 833 | 0.027 | -0.17563 |

| | | |
|---|---|---|
| 29 -> 832 | 0.024 | -0.1457 |
| 30 -> 831 | 0.024 | -0.13435 |
| 31 -> 830 | 0.023 | -0.1265 |
| 32 -> 829 | 0.023 | -0.12002 |
| 33 -> 828 | 0.02 | -0.12631 |
| 34 -> 827 | 0.019 | -0.14436 |
| 35 -> 826 | 0.019 | -0.09864 |
| 36 -> 825 | 0.018 | -0.09103 |
| 37 -> 824 | 0.017 | -0.09295 |
| 38 -> 823 | 0.016 | -0.05986 |
| 39 -> 822 | 0.016 | -0.05402 |
| 40 -> 821 | 0.014 | -0.07579 |
| 41 -> 820 | 0.014 | -0.05426 |
| 42 -> 819 | 0.013 | -0.0511 |
| 43 -> 818 | 0.013 | -0.05422 |
| 44 -> 817 | 0.012 | -0.05011 |
| 45 -> 816 | 0.012 | -0.03372 |
| 46 -> 815 | 0.011 | -0.03898 |
| 47 -> 814 | 0.01 | -0.02546 |
| 48 -> 813 | 0.01 | -0.03871 |

## 3.5. Selected deformation density plots of model complexes 10a-16b

**Table S22**: Selected deformation density plots of model complexes **10a-16b** (all energies are in kcal/mol).

| Model complex | L→M σ-donation | M→L π-backdonation | | M→L σ-backdonation |
|---|---|---|---|---|
| **10a** | 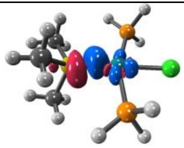<br>$|\upsilon_2| = 0.442$<br>ΔE = -32.26 | 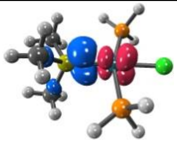<br>$|\upsilon_1| = 0.483$<br>ΔE = -22.89 | 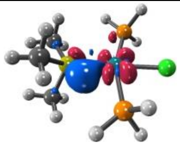<br>$|\upsilon_3| = 0.413$<br>ΔE = -21.04 | 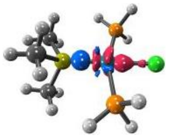<br>$|\upsilon_4| = 0.145$<br>ΔE = -4.55 |
| **10b** | 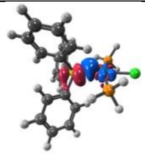<br>$|\upsilon_2| = 0.441$<br>ΔE = -31.13 | 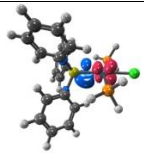<br>$|\upsilon_1| = 0.470$<br>ΔE = -21.30 | 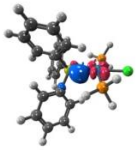<br>$|\upsilon_3| = 0.389$<br>ΔE = -18.10 | 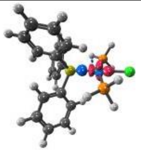<br>$|\upsilon_4| = 0.138$<br>ΔE = -4.07 |
| **11a** | 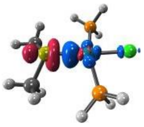<br>$|\upsilon_1| = 0.515$<br>ΔE = -35.03 | 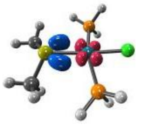<br>$|\upsilon_2| = 0.278$<br>ΔE = -8.80 | 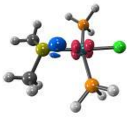<br>$|\upsilon_3| = 0.204$<br>ΔE = -6.09 | 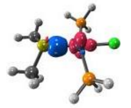<br>$|\upsilon_4| = 0.123$<br>ΔE = -3.41 |
| **11b** | 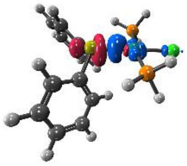<br>$|\upsilon_1| = 0.502$ ΔE = -34.39 | 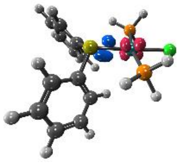<br>$|\upsilon_3| = 0.227$ ΔE = -6.90 | 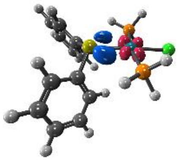<br>$|\upsilon_2| = 0.282$ ΔE = -9.20 | 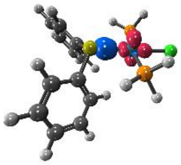<br>$|\upsilon_4| = 0.130$<br>ΔE = -3.44 |
| **12a** | 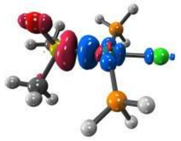<br>$|\upsilon_1| = 0.556$<br>ΔE = -39.82 | 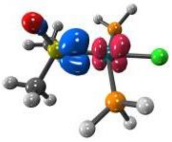<br>$|\upsilon_2| = 0.344$<br>ΔE = -13.01 | 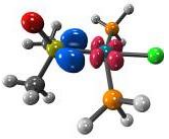<br>$|\upsilon_3| = 0.303$<br>ΔE = -11.41 | 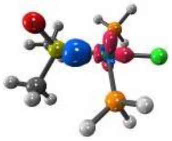<br>$|\upsilon_4| = 0.112$<br>ΔE = -3.55 |
| **12b** | 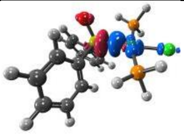<br>$|\upsilon_1| = 0.547$<br>ΔE = -40.73 | 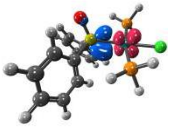<br>$|\upsilon_2| = 0.389$<br>ΔE =-15.93 | 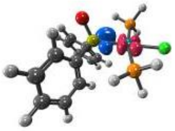<br>$|\upsilon_3| = 0.318$<br>ΔE =-12.99 | 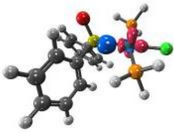<br>$|\upsilon_4| = 0.119$<br>ΔE =-3.58 |
| **13a** | 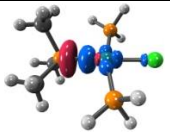<br>$|\upsilon_1| = 0.633$<br>ΔE = -56.69 | 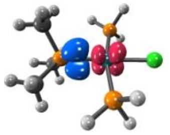<br>$|\upsilon_2| = 0.323$<br>ΔE = -11.62 | 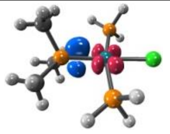<br>$|\upsilon_3| = 0.278$<br>ΔE = -10.44 | 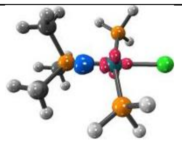<br>$|\upsilon_4| = 0.130$<br>ΔE = -3.93 |

| | | | | |
|---|---|---|---|---|
| **13b** | 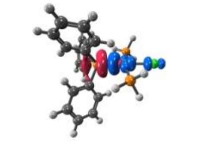<br>$\lvert\upsilon_1\rvert$ = 0.620<br>ΔE = -53.31 | 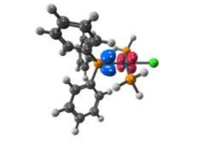<br>$\lvert\upsilon_2\rvert$ = 0.348<br>ΔE = -13.03 | 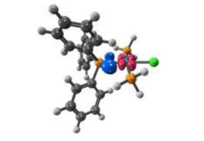<br>$\lvert\upsilon_3\rvert$ = 0.294<br>ΔE = -11.16 | 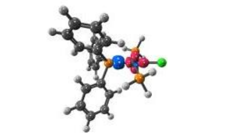<br>$\lvert\upsilon_4\rvert$ = 0.130<br>ΔE = -3.76 |
| **14a** | 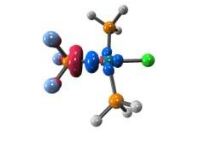<br>$\lvert\upsilon_1\rvert$ = 0.582<br>ΔE = -51.83 | 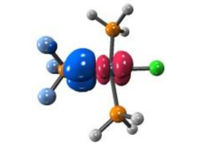<br>$\lvert\upsilon_2\rvert$ = 0.466<br>ΔE = -23.59 | 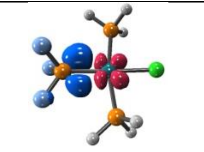<br>$\lvert\upsilon_3\rvert$ = 385<br>ΔE = -21.02 | 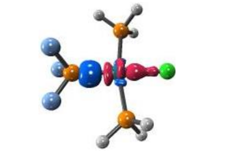<br>$\lvert\upsilon_4\rvert$ = 0.117<br>ΔE = -3.90 |
| **14b** | 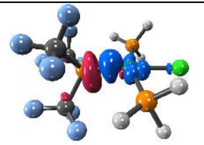<br>$\lvert\upsilon_1\rvert$ = 0.557<br>ΔE = -46.89 | 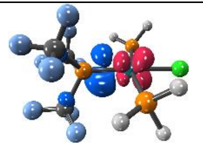<br>$\lvert\upsilon_2\rvert$ = 0.465<br>ΔE = -21.76 | 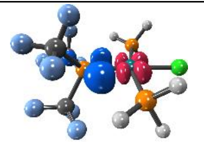<br>$\lvert\upsilon_3\rvert$ = 0.398<br>ΔE = -18.99 | 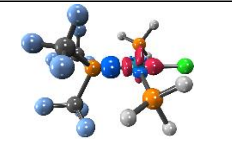<br>$\lvert\upsilon_4\rvert$ = 0.130<br>ΔE = -4.00 |
| **14c** | 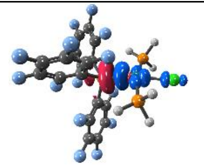<br>$\lvert\upsilon_1\rvert$ = 0.577<br>ΔE = -47.79 | 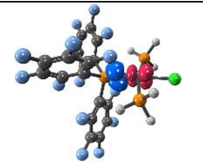<br>$\lvert\upsilon_2\rvert$ = 0.392<br>ΔE = -16.64 | 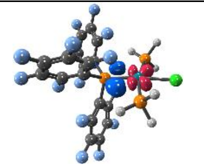<br>$\lvert\upsilon_3\rvert$ = 0.365<br>ΔE = -15.57 | 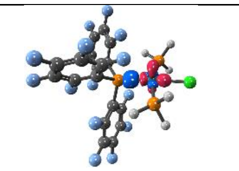<br>$\lvert\upsilon_4\rvert$ = 0.147<br>ΔE = -3.92 |
| **15a** | 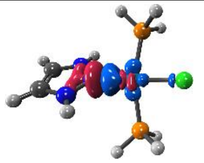<br>$\lvert\upsilon_2\rvert$ = 0.382<br>ΔE = -25.30 | 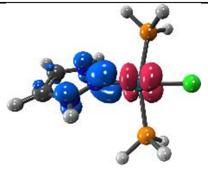<br>$\lvert\upsilon_1\rvert$ = 0.673<br>ΔE = -21.37 | 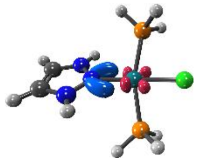<br>$\lvert\upsilon_3\rvert$ = 0.215<br>ΔE = -7.82 | 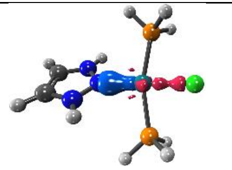<br>$\lvert\upsilon_4\rvert$ = 0.127<br>ΔE = -3.96 |
| **15b** | 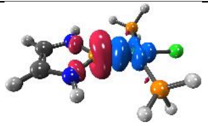<br>$\lvert\upsilon_2\rvert$ = 0.535<br>ΔE = -42.93 | 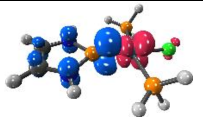<br>$\lvert\upsilon_1\rvert$ = 0.676<br>ΔE = -34.82 | 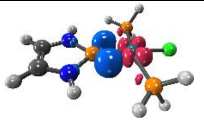<br>$\lvert\upsilon_3\rvert$ = 0.437<br>ΔE = -27.60 | 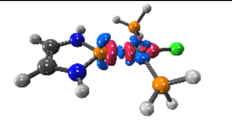<br>$\lvert\upsilon_4\rvert$ = 0.154<br>ΔE = -5.99 |
| **15c** | 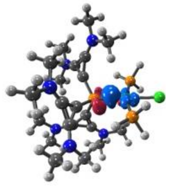<br>$\lvert\upsilon_1\rvert$ = 0.540<br>ΔE = -44.43 | 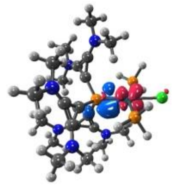<br>$\lvert\upsilon_2\rvert$ = 0.497<br>ΔE = -25.30 | 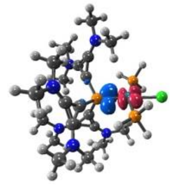<br>$\lvert\upsilon_3\rvert$ = 0.431<br>ΔE = -20.87 | 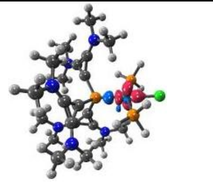<br>$\lvert\upsilon_4\rvert$ = 0.171<br>ΔE = -5.64 |

| | | | | |
|---|---|---|---|---|
| **16a** | 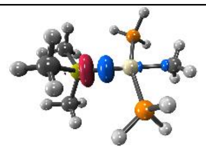<br>$|\upsilon_1| = 0.401$ ΔE = -33.74 | 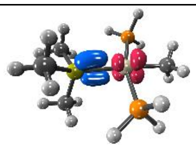<br>$|\upsilon_3| = 0.269$<br>ΔE = -8.58 | 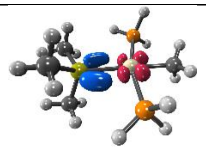<br>$|\upsilon_2| = 0.255$<br>ΔE = -8.62 | 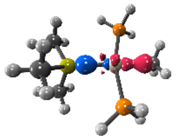<br>$|\upsilon_4| = 0.117$<br>ΔE = -2.99 |
| **16b** | 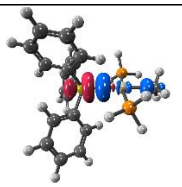<br>$|\upsilon_1| = 0.422$<br>ΔE = -32.84 | 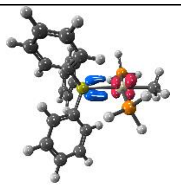<br>$|\upsilon_3| = 0.239$<br>ΔE = -7.11 | 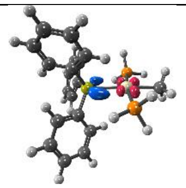<br>$|\upsilon_2| = 0.209$<br>ΔE = -6.42 | 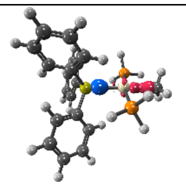<br>$|\upsilon_4| = 0.115$<br>ΔE = -2.64 |

## 3.6 Selected deformation density plots of model complexes 17a-18b

**Table S23**: Selected deformation density plots of model pincer complexes **17a-18b** (all energies are given in kcal/mol).

| Model complex | | | | |
|---|---|---|---|---|
| **17a** | 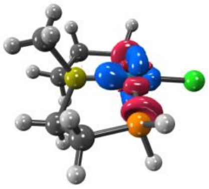<br>$|\upsilon1| = 1.167$<br>ΔE = -102.50 | 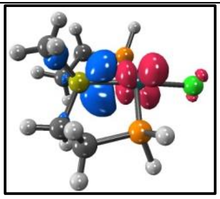<br>$|\upsilon_2| = 0.588$<br>ΔE = -33.38 | 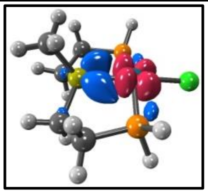<br>$|\upsilon_3| = 0.465$<br>ΔE = -30.00 | 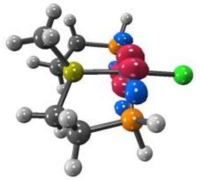<br>$|\upsilon_4| = 0.332$<br>ΔE = -16.79 |
| | 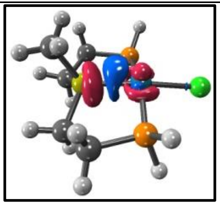<br>$|\upsilon_5| = 0.265$<br>ΔE = -19.91 | 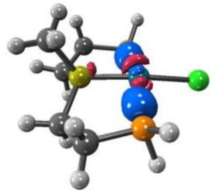<br>$|\upsilon_6| = 0.217$<br>ΔE = -25.80 | 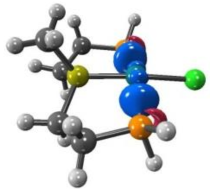<br>$|\upsilon_7| = 0.207$<br>ΔE = -7.81 | 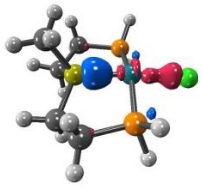<br>$|\upsilon_8| = 0.119$<br>ΔE = -5.52 |
| **17b** | 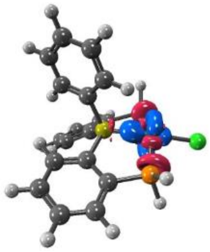<br>$|\upsilon_1| = 1.156$<br>ΔE = -99.58 | 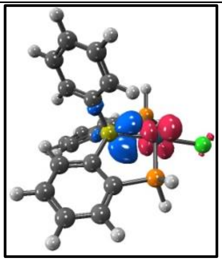<br>$|\upsilon_2| = 0.542$<br>ΔE = -29.08 | 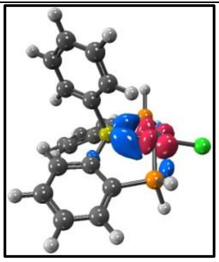<br>$|\upsilon_3| = 0.468$<br>ΔE = -30.02 | 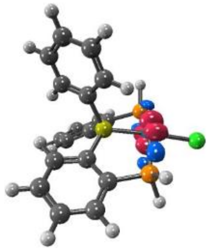<br>$|\upsilon_4| = 0.338$<br>ΔE = -17.60 |

| | | | | |
|---|---|---|---|---|
| | 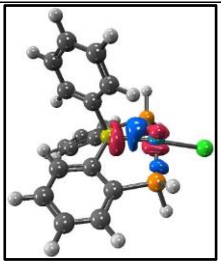<br>$\lvert\upsilon_5\rvert = 0.262$<br>ΔE = -18.17 | 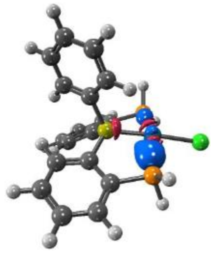<br>$\lvert\upsilon_6\rvert = 0.221$<br>ΔE = -26.50 | 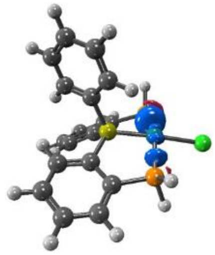<br>$\lvert\upsilon_7\rvert = 0.205$<br>ΔE = -8.01 | 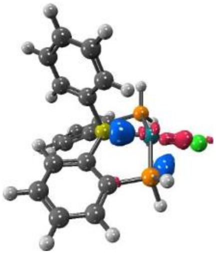<br>$\lvert\upsilon_8\rvert = 0.118$<br>ΔE = -5.59 |
| **18a** | 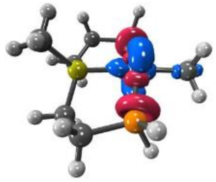<br>$\lvert\upsilon_1\rvert = 1.072$<br>ΔE = -143.00 | 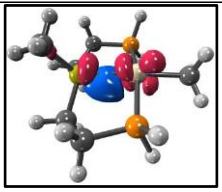<br>$\lvert\upsilon_2\rvert = 0.399$<br>ΔE = -22.17 | 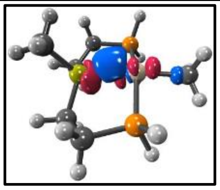<br>$\lvert\upsilon_3\rvert = 0.369$<br>ΔE = -47.16 | 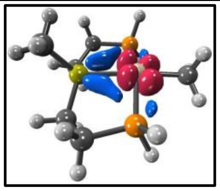<br>$\lvert\upsilon_4\rvert = 0.350$<br>ΔE = -17.55 |
| | 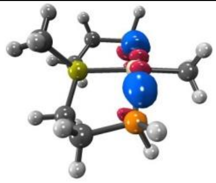<br>$\lvert\upsilon_5\rvert = 0.312$<br>ΔE = -17.88 | 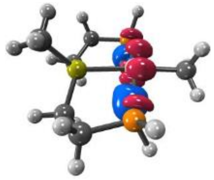<br>$\lvert\upsilon_6\rvert = 0.287$<br>ΔE = -15.33 | 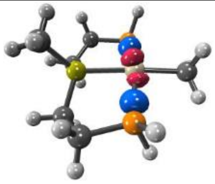<br>$\lvert\upsilon_7\rvert = 0.235$<br>ΔE = -21.76 | 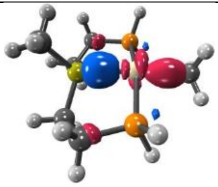<br>$\lvert\upsilon_8\rvert = 0.166$<br>ΔE = -10.38 |
| **18b** | 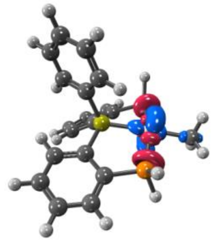<br>$\lvert\upsilon_1\rvert = 1.077$<br>ΔE = -141.35 | 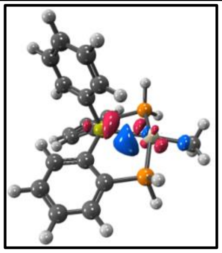<br>$\lvert\upsilon_2\rvert = 0.387$<br>ΔE = -45.02 | 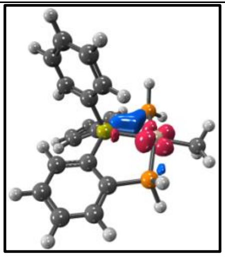<br>$\lvert\upsilon_3\rvert = 0.354$<br>ΔE = -22.70 | 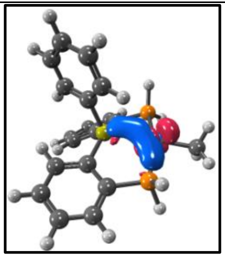<br>$\lvert\upsilon_4\rvert = 0.342$<br>ΔE = -18.87 |
| | 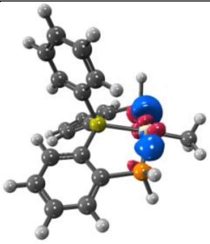<br>$\lvert\upsilon_5\rvert = 0.306$<br>ΔE = -16.95 | 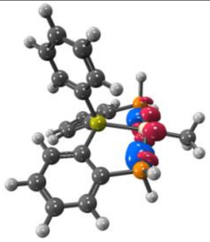<br>$\lvert\upsilon_6\rvert = 0.285$<br>ΔE = -16.95 | 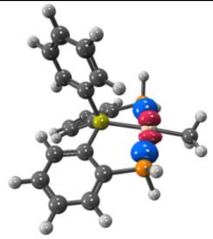<br>$\lvert\upsilon_7\rvert = 0.247$<br>ΔE = -15.52 | 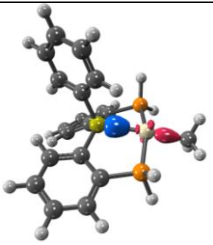<br>$\lvert\upsilon_8\rvert = 0.160$<br>ΔE = -9.87 |

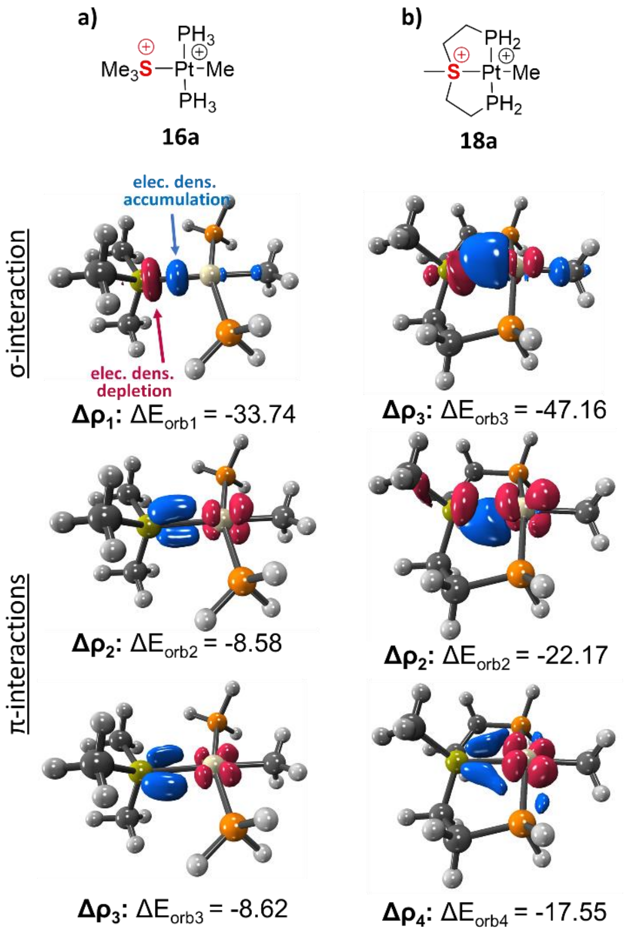


**Figure S18**: Selected deformation density plots of model complexes **16a** and **18a** (All energies are given in kcal/mol).

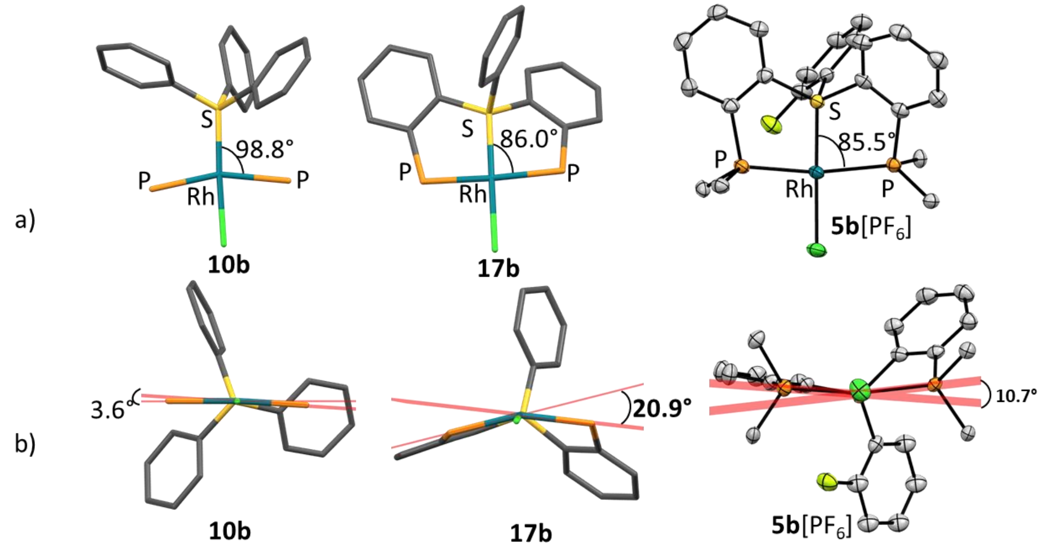


**Figure S19**: The in-plane (**a**) and out-of-plane (**b**) deformations in model complexes **10b**, **17b** and XRD structure of **5b**[$PF_6$].

### 3.7 Coordinates of the optimized model complexes 10a-18b.

$[(SMe_3)(PH_3)_2RhCl]^+$, **10a**

| | | | |
|---|---|---|---|
| Rh | -0.30350100 | -0.00024000 | -0.12111400 |
| Cl | -2.66847000 | -0.00100300 | -0.01323000 |
| P | -0.63394600 | -2.23652100 | 0.17720600 |
| P | -0.63610000 | 2.23615000 | 0.17512000 |
| H | -1.27362800 | -2.54307700 | 1.38942300 |
| H | -1.51667800 | -2.89442700 | -0.69685100 |
| H | -1.51829400 | 2.89289700 | -0.70033200 |
| H | -1.27725200 | 2.54292000 | 1.38647500 |
| H | 0.35590800 | -3.24450000 | 0.23397600 |
| H | 0.35312700 | 3.24478300 | 0.23223100 |
| C | 2.82423000 | -1.37272300 | 0.28952600 |
| C | 2.82166000 | 1.37366500 | 0.29705300 |
| H | 3.89157200 | -1.26010800 | 0.10140800 |
| H | 2.63131000 | -1.42634000 | 1.36025700 |
| H | 2.62867100 | 1.42089400 | 1.36806300 |
| H | 3.88922100 | 1.26434900 | 0.10821500 |
| S | 1.94303300 | 0.00172200 | -0.47138000 |
| H | 2.46988900 | 2.28567700 | -0.17873200 |
| H | 2.47450300 | -2.28283500 | -0.19136500 |

$[(SPh_3)(PH_3)_2RhCl]^+$, **10b**

| | | | |
|---|---|---|---|
| Rh | 1.20076900 | 0.75134000 | 1.64556500 |
| P | 1.21305800 | -1.14986600 | 2.95930500 |
| H | 2.49371300 | -1.58095600 | 3.33264700 |
| H | 0.61000700 | -1.03026200 | 4.21983200 |
| H | 0.66370400 | -2.38165200 | 2.54808700 |
| P | 1.62733100 | 2.88152700 | 0.85632400 |
| H | 1.00337400 | 3.92191200 | 1.56074000 |
| H | 2.96755000 | 3.27381800 | 0.97333400 |
| H | 1.36931300 | 3.30035300 | -0.46315100 |
| Cl | 2.23928500 | 1.62822600 | 3.52884700 |
| C | 1.01520100 | -1.73272500 | -0.51231900 |
| C | 0.30061200 | -2.85428500 | -0.89968100 |
| C | 2.40210900 | -1.75727500 | -0.41228100 |
| C | 0.99427500 | -4.01695900 | -1.20206200 |
| H | -0.77758400 | -2.84747100 | -0.95046200 |
| C | 3.08053900 | -2.92036400 | -0.73059400 |
| H | 2.93917200 | -0.87718800 | -0.07974800 |
| C | 2.37720100 | -4.05004900 | -1.12475000 |
| H | 0.44385600 | -4.90104700 | -1.49460300 |
| H | 4.15967400 | -2.94491600 | -0.65971700 |
| H | 2.90930400 | -4.96159500 | -1.36271500 |
| C | -1.50545300 | -0.55658200 | 0.09054000 |
| C | -2.27056700 | -0.89087200 | -1.02199000 |
| C | -2.06636100 | -0.47817800 | 1.35472900 |
| C | -3.61121400 | -1.18098400 | -0.84898300 |
| H | -1.83338000 | -0.91223000 | -2.01145200 |
| C | -3.41245900 | -0.77098300 | 1.51381300 |
| H | -1.46628100 | -0.16119100 | 2.19755100 |
| C | -4.18021600 | -1.12642500 | 0.41735600 |
| H | -4.21593300 | -1.44207200 | -1.70708300 |
| H | -3.85979800 | -0.70658800 | 2.49647300 |
| H | -5.23148600 | -1.34896900 | 0.54433800 |
| C | 0.22034200 | 0.73478100 | -1.62994600 |
| C | 1.03757100 | 0.38010800 | -2.68753000 |
| C | -0.60988500 | 1.84695800 | -1.69166800 |
| C | 1.01715000 | 1.16037700 | -3.83648100 |
| H | 1.67800000 | -0.48870400 | -2.63650800 |
| C | -0.62488400 | 2.60905800 | -2.84496300 |
| H | -1.24338000 | 2.11139800 | -0.85388100 |
| C | 0.19012700 | 2.26735700 | -3.91695300 |

| | | | |
|---|---|---|---|
| H | 1.65025300 | 0.89092700 | -4.67115700 |
| H | -1.27405400 | 3.47223500 | -2.90630700 |
| H | 0.17672400 | 2.86736500 | -4.81709900 |
| S | 0.23901400 | -0.16881300 | -0.07469000 |

[$(SMe_2)(PH_3)_2RhCl$], **11a**

| | | | |
|---|---|---|---|
| Rh | -0.30350100 | -0.00024000 | -0.12111400 |
| Cl | -2.66847000 | -0.00100300 | -0.01323000 |
| P | -0.63394600 | -2.23652100 | 0.17720600 |
| P | -0.63610000 | 2.23615000 | 0.17512000 |
| H | -1.27362800 | -2.54307700 | 1.38942300 |
| H | -1.51667800 | -2.89442700 | -0.69685100 |
| H | -1.51829400 | 2.89289700 | -0.70033200 |
| H | -1.27725200 | 2.54292000 | 1.38647500 |
| H | 0.35590800 | -3.24450000 | 0.23397600 |
| H | 0.35312700 | 3.24478300 | 0.23223100 |
| C | 2.82423000 | -1.37272300 | 0.28952600 |
| C | 2.82166000 | 1.37366500 | 0.29705300 |
| H | 3.89157200 | -1.26010800 | 0.10140800 |
| H | 2.63131000 | -1.42634000 | 1.36025700 |
| H | 2.62867100 | 1.42089400 | 1.36806300 |
| H | 3.88922100 | 1.26434900 | 0.10821500 |
| S | 1.94303300 | 0.00172200 | -0.47138000 |
| H | 2.46988900 | 2.28567700 | -0.17873200 |
| H | 2.47450300 | -2.28283500 | -0.19136500 |

[$(SPh_2)(PH_3)_2RhCl$], **11b**

| | | | |
|---|---|---|---|
| C | 2.29600400 | -0.24789400 | 1.19598200 |
| C | 2.04642400 | 0.07414100 | -0.13378200 |
| C | 3.60463000 | -0.42150900 | 1.61796200 |
| C | 3.09483200 | 0.20548200 | -1.03365200 |
| C | 4.65594400 | -0.28058600 | 0.72308000 |
| H | 3.81157300 | -0.67645900 | 2.64814300 |
| C | 4.40101700 | 0.02949500 | -0.60262100 |
| H | 2.88976900 | 0.43314300 | -2.07198400 |
| H | 5.67467800 | -0.42240300 | 1.06052100 |
| H | 5.21723600 | 0.12906400 | -1.30655800 |
| C | -0.00623500 | 2.93300000 | -1.35825000 |
| C | -0.22762300 | 4.27355700 | -1.07032900 |
| C | 0.14198200 | 2.02243800 | -0.32422600 |
| C | -0.29103700 | 4.69944600 | 0.24548300 |
| H | -0.34823300 | 4.98289800 | -1.87899700 |
| C | 0.06859600 | 2.44507200 | 0.99744700 |
| C | -0.14073300 | 3.78324900 | 1.27907100 |
| H | -0.46172600 | 5.74458400 | 0.46958800 |
| H | 0.15998500 | 1.72330800 | 1.79924100 |
| H | -0.19546600 | 4.11229900 | 2.30889700 |
| S | 0.37706500 | 0.29807300 | -0.74976900 |
| H | 1.46638000 | -0.38328300 | 1.87849700 |
| H | 0.04750600 | 2.59319400 | -2.38463600 |
| H | -2.78271500 | 1.72429800 | 0.22200900 |
| H | -3.11709400 | 0.63191000 | 2.01371100 |
| H | -0.35972800 | -4.02680800 | -0.64397600 |
| P | 0.23428500 | -2.94433700 | 0.02565700 |
| P | -2.77400300 | 0.38622100 | 0.67146000 |
| H | 0.63500000 | -3.61534700 | 1.19485100 |
| H | -4.03980600 | -0.00333400 | 0.20390700 |
| Cl | -2.57110400 | -2.63202300 | 1.28860000 |
| Rh | -1.07903600 | -1.09596500 | 0.29235600 |
| H | 1.48168900 | -2.94694400 | -0.63613300 |

[$(DMSO)(PH_3)_2RhCl$], **12a**

| | | | |
|---|---|---|---|
| C | -2.47814300 | -1.34308000 | -0.77461300 |
| C | -2.47784700 | 1.34367600 | -0.77456900 |

| | | | |
|---|---|---|---|
| H | -3.55759100 | -1.20036300 | -0.80226100 |
| H | -2.04974500 | -1.36200400 | -1.77592500 |
| H | -2.04945000 | 1.36253900 | -1.77588300 |
| H | -3.55732600 | 1.20119500 | -0.80221700 |
| O | -2.44032200 | 0.00025800 | 1.46661300 |
| S | -1.75461000 | 0.00020300 | 0.17101200 |
| H | -2.24496200 | 2.26385700 | -0.24477500 |
| H | -2.24546000 | -2.26332900 | -0.24484700 |
| Rh | 0.45352800 | -0.00005600 | 0.16242700 |
| Cl | 2.81716100 | -0.00034400 | 0.26655100 |
| P | 0.82500700 | -2.24453200 | -0.10692400 |
| P | 0.82554600 | 2.24432300 | -0.10695700 |
| H | 1.57377900 | -2.54047400 | -1.25682800 |
| H | 1.63448500 | -2.87864700 | 0.84973100 |
| H | 1.63517300 | 2.87825900 | 0.84969200 |
| H | 1.57439900 | 2.54006600 | -1.25686000 |
| H | -0.14581700 | -3.26077900 | -0.24383700 |
| H | -0.14502200 | 3.26081200 | -0.24389500 |

[$(Ph_2SO)(PH_3)_2RhCl$], **12b**

| | | | |
|---|---|---|---|
| C | 2.29600400 | -0.24789400 | 1.19598200 |
| C | 2.04642400 | 0.07414100 | -0.13378200 |
| C | 3.60463000 | -0.42150900 | 1.61796200 |
| C | 3.09483200 | 0.20548200 | -1.03365200 |
| C | 4.65594400 | -0.28058600 | 0.72308000 |
| H | 3.81157300 | -0.67645900 | 2.64814300 |
| C | 4.40101700 | 0.02949500 | -0.60262100 |
| H | 2.88976900 | 0.43314300 | -2.07198400 |
| H | 5.67467800 | -0.42240300 | 1.06052100 |
| H | 5.21723600 | 0.12906400 | -1.30655800 |
| C | -0.00623500 | 2.93300000 | -1.35825000 |
| C | -0.22762300 | 4.27355700 | -1.07032900 |
| C | 0.14198200 | 2.02243800 | -0.32422600 |
| C | -0.29103700 | 4.69944600 | 0.24548300 |
| H | -0.34823300 | 4.98289800 | -1.87899700 |
| C | 0.06859600 | 2.44507200 | 0.99744700 |
| C | -0.14073300 | 3.78324900 | 1.27907100 |
| H | -0.46172600 | 5.74458400 | 0.46958800 |
| H | 0.15998500 | 1.72330800 | 1.79924100 |
| H | -0.19546600 | 4.11229900 | 2.30889700 |
| S | 0.37706500 | 0.29807300 | -0.74976900 |
| H | 1.46638000 | -0.38328300 | 1.87849700 |
| H | 0.04750600 | 2.59319400 | -2.38463600 |
| H | -2.78271500 | 1.72429800 | 0.22200900 |
| H | -3.11709400 | 0.63191000 | 2.01371100 |
| H | -0.35972800 | -4.02680800 | -0.64397600 |
| P | 0.23428500 | -2.94433700 | 0.02565700 |
| P | -2.77400300 | 0.38622100 | 0.67146000 |
| H | 0.63500000 | -3.61534700 | 1.19485100 |
| H | -4.03980600 | -0.00333400 | 0.20390700 |
| Cl | -2.57110400 | -2.63202300 | 1.28860000 |
| Rh | -1.07903600 | -1.09596500 | 0.29235600 |
| H | 1.48168900 | -2.94694400 | -0.63613300 |

[$(PMe_3)(PH_3)_2RhCl$], **13a**

| | | | |
|---|---|---|---|
| Rh | 1.18803600 | 0.96524100 | 1.81435200 |
| P | 0.79173200 | -0.74928500 | 3.24986400 |
| H | 1.91371600 | -1.27270200 | 3.91540800 |
| H | 0.00278900 | -0.44689700 | 4.37314100 |
| H | 0.18027300 | -1.98065700 | 2.91694600 |
| P | 1.79004000 | 2.92069400 | 0.81816700 |
| H | 1.23735500 | 4.08698800 | 1.37492300 |
| H | 3.14860800 | 3.26680900 | 0.91886300 |
| H | 1.60834700 | 3.25357900 | -0.54206600 |

| | | | |
|---|---|---|---|
| Cl | 2.09944200 | 2.01555800 | 3.75912400 |
| C | -1.42830800 | -0.56440700 | 0.19227800 |
| H | -2.03669000 | 0.32449600 | 0.35835100 |
| H | -1.77579600 | -1.08002800 | -0.70524000 |
| H | -1.55315500 | -1.22337900 | 1.05173700 |
| C | 0.30782000 | 0.77377200 | -1.57514600 |
| H | -0.14308100 | 0.14042500 | -2.34095700 |
| H | -0.26149400 | 1.70064000 | -1.50053800 |
| H | 1.32718500 | 1.02033400 | -1.87329800 |
| C | 1.11762600 | -1.65657800 | -0.40655100 |
| H | 1.06484200 | -2.34599600 | 0.43631500 |
| H | 0.63290300 | -2.11380300 | -1.27148800 |
| H | 2.16927000 | -1.47973800 | -0.63153900 |
| P | 0.32676800 | -0.07053800 | 0.04586200 |

[($PPh_3$)($PH_3$)$_2$RhCl], **13b**

| | | | |
|---|---|---|---|
| Rh | 1.24701300 | 0.79324700 | 1.70986300 |
| P | 1.20433100 | -1.12438600 | 2.93972100 |
| H | 2.46160100 | -1.62423500 | 3.32018600 |
| H | 0.61051200 | -1.05593700 | 4.21259800 |
| H | 0.62842500 | -2.33781100 | 2.50643300 |
| P | 1.62357500 | 2.86239500 | 0.82780100 |
| H | 0.97753800 | 3.94308000 | 1.45553700 |
| H | 2.94582700 | 3.32490700 | 0.93647400 |
| H | 1.37896400 | 3.22820200 | -0.51154800 |
| Cl | 2.30831100 | 1.70604000 | 3.64096400 |
| P | 0.25423900 | -0.15249300 | -0.04374200 |
| C | 1.02848800 | -1.75311400 | -0.50715200 |
| C | 0.32480300 | -2.88590500 | -0.90187100 |
| C | 2.42030600 | -1.82260300 | -0.43010300 |
| C | 0.99690300 | -4.05659700 | -1.22344900 |
| H | -0.75546000 | -2.87051200 | -0.94633100 |
| C | 3.09197800 | -2.98652400 | -0.76429600 |
| H | 2.97402600 | -0.95491500 | -0.08871400 |
| C | 2.37985200 | -4.10859700 | -1.16211200 |
| H | 0.43315300 | -4.93223800 | -1.51967200 |
| H | 4.17228300 | -3.02013400 | -0.70183700 |
| H | 2.90157000 | -5.02337500 | -1.41341500 |
| C | -1.52732900 | -0.54625800 | 0.13822600 |
| C | -2.31895900 | -0.90395400 | -0.95398600 |
| C | -2.11784600 | -0.47105600 | 1.39505900 |
| C | -3.65926500 | -1.20499200 | -0.78583500 |
| H | -1.88665100 | -0.93536400 | -1.94674600 |
| C | -3.46154200 | -0.77249900 | 1.56560800 |
| H | -1.52330900 | -0.14633300 | 2.24040000 |
| C | -4.23251700 | -1.14453300 | 0.47717300 |
| H | -4.26029100 | -1.48076000 | -1.64312100 |
| H | -3.90599700 | -0.70481700 | 2.55037700 |
| H | -5.28213500 | -1.37591000 | 0.60791800 |
| C | 0.23587600 | 0.76094100 | -1.63417800 |
| C | 1.12406500 | 0.49036400 | -2.66772900 |
| C | -0.65002200 | 1.82925500 | -1.77221000 |
| C | 1.12592400 | 1.26986400 | -3.81697200 |
| H | 1.81795600 | -0.33619900 | -2.58675300 |
| C | -0.65271700 | 2.60238800 | -2.91970300 |
| H | -1.34709100 | 2.05644900 | -0.97353100 |
| C | 0.23877600 | 2.32484900 | -3.94709000 |
| H | 1.82289800 | 1.04451500 | -4.61427700 |
| H | -1.35094800 | 3.42465600 | -3.01217200 |
| H | 0.23917800 | 2.92944000 | -4.84508400 |

[($PF_3$)($PH_3$)$_2$RhCl], **14a**

| | | | |
|---|---|---|---|
| Rh | 1.18950200 | 0.96239600 | 1.82339000 |
| P | 0.77951000 | -0.78129600 | 3.25325600 |

| | | | |
|---|---|---|---|
| H | 1.91013100 | -1.29448600 | 3.90694200 |
| H | -0.01745700 | -0.46558800 | 4.36416900 |
| H | 0.16947700 | -1.99246200 | 2.87613900 |
| P | 1.78404600 | 2.92028400 | 0.78935400 |
| H | 1.21735600 | 4.08292800 | 1.33406000 |
| H | 3.14306100 | 3.25848100 | 0.87977000 |
| H | 1.57337600 | 3.18101200 | -0.57663100 |
| Cl | 2.09084700 | 2.00232300 | 3.74503500 |
| P | 0.36900200 | -0.01456000 | 0.12776800 |
| F | -1.12621700 | -0.48003000 | 0.15689500 |
| F | 0.33408100 | 0.67870800 | -1.27581700 |
| F | 0.97453800 | -1.38109200 | -0.34042600 |

[(P($CF_3$)$_3$)($PH_3$)$_2$RhCl], **14b**

| | | | |
|---|---|---|---|
| P | 0.37020000 | -0.04170000 | 0.07270000 |
| C | 0.42240000 | 0.77940000 | -1.65070000 |
| C | -1.48640000 | -0.49720000 | 0.10720000 |
| C | 1.08660000 | -1.74000000 | -0.44290000 |
| F | -2.17730000 | 0.54240000 | 0.57020000 |
| F | -2.01390000 | -0.84680000 | -1.06160000 |
| F | -1.65990000 | -1.51960000 | 0.95670000 |
| F | 0.32290000 | -2.43970000 | -1.27700000 |
| F | 1.28780000 | -2.49520000 | 0.64130000 |
| F | 2.26970000 | -1.54370000 | -1.02740000 |
| F | -0.41380000 | 1.82420000 | -1.65250000 |
| F | 0.09250000 | -0.01120000 | -2.66490000 |
| F | 1.65430000 | 1.23970000 | -1.88580000 |
| Rh | 1.18170000 | 0.93730000 | 1.80990000 |
| P | 0.79920000 | -0.77780000 | 3.29020000 |
| H | 1.95710000 | -1.31890000 | 3.86940000 |
| H | 0.11190000 | -0.37840000 | 4.44520000 |
| H | 0.10740000 | -1.96830000 | 3.01080000 |
| P | 1.74240000 | 2.97370000 | 0.90150000 |
| H | 1.22390000 | 4.06680000 | 1.61030000 |
| H | 3.11000000 | 3.28470000 | 0.94620000 |
| H | 1.45860000 | 3.39770000 | -0.40840000 |
| Cl | 2.09490000 | 1.94380000 | 3.74110000 |

[(P($C_6F_5$)$_3$)($PH_3$)$_2$RhCl], **14c**

| | | | |
|---|---|---|---|
| P | 0.23957700 | -0.18038100 | -0.02679800 |
| C | 1.13824800 | -1.70920800 | -0.52864000 |
| C | 0.72593900 | -3.00432500 | -0.25131500 |
| C | 2.44000600 | -1.56931300 | -0.99970500 |
| C | 1.53882700 | -4.10479900 | -0.46985700 |
| C | 3.27177700 | -2.64530900 | -1.23188600 |
| C | 2.81609600 | -3.92678700 | -0.96710000 |
| C | -1.54806500 | -0.64949800 | 0.00396200 |
| C | -2.14783800 | -1.33207600 | -1.05125800 |
| C | -2.39172400 | -0.25149000 | 1.03559900 |
| C | -3.49391900 | -1.63294200 | -1.08233700 |
| C | -3.74900800 | -0.53968400 | 1.03023900 |
| C | -4.30162600 | -1.23381800 | -0.02906200 |
| C | 0.11708600 | 0.85096300 | -1.56430900 |
| C | 0.49289200 | 0.52548600 | -2.86282100 |
| C | -0.44308000 | 2.11516100 | -1.39155700 |
| C | 0.36101700 | 1.41794100 | -3.91565300 |
| C | -0.59079400 | 3.02263400 | -2.42318700 |
| C | -0.17900000 | 2.67188500 | -3.69742600 |
| F | -1.94744100 | 0.43660700 | 2.07644500 |
| F | -4.51713800 | -0.14586700 | 2.03269700 |
| F | -5.59011000 | -1.51125700 | -0.04207700 |
| F | -4.01524800 | -2.29559700 | -2.10178800 |
| F | -1.41548800 | -1.73411500 | -2.08524600 |
| F | -0.47710300 | -3.25150200 | 0.26776000 |

| | | | |
|---|---|---|---|
| F | 1.09785600 | -5.32292100 | -0.19740300 |
| F | 3.59830000 | -4.96749600 | -1.18123600 |
| F | 4.49524200 | -2.46171500 | -1.69915200 |
| F | 2.92851400 | -0.35687600 | -1.25114800 |
| F | 0.98558200 | -0.66597800 | -3.17316900 |
| F | 0.73824000 | 1.06753700 | -5.13428500 |
| F | -0.31222800 | 3.52224900 | -4.69646300 |
| F | -1.12422300 | 4.21283600 | -2.20177600 |
| F | -0.87693300 | 2.49060300 | -0.19145300 |
| Rh | 1.28669600 | 0.55590400 | 1.74889600 |
| P | 0.55126700 | -1.09626700 | 3.15445100 |
| H | 1.54324700 | -1.94218100 | 3.67591600 |
| H | -0.02329100 | -0.64645900 | 4.35295300 |
| H | -0.40321000 | -2.07973100 | 2.81902600 |
| P | 2.36793400 | 2.33160300 | 0.80722400 |
| H | 2.18462000 | 3.57122600 | 1.44072100 |
| H | 3.76984900 | 2.26859400 | 0.82867000 |
| H | 2.19700200 | 2.74942900 | -0.52971600 |
| Cl | 2.55760900 | 1.18313900 | 3.64870700 |

$[(NHN)(PH_3)_2RhCl]^+$, **15a**

| | | | |
|---|---|---|---|
| N | 0.79950900 | -1.19469500 | -0.31209500 |
| C | -0.75251700 | -0.49759600 | -1.63677100 |
| C | 0.08654000 | -1.53667900 | -1.41839300 |
| H | 1.49397200 | -1.72824500 | 0.18937800 |
| H | -1.49641300 | -0.32098200 | -2.39316900 |
| H | 0.23185700 | -2.46543000 | -1.94089000 |
| N | -0.48380300 | 0.39387800 | -0.64559200 |
| H | -0.87179200 | 1.31267200 | -0.49163300 |
| N | 0.46460400 | -0.01492000 | 0.17676400 |
| Rh | 1.19684100 | 0.90324100 | 1.74328400 |
| P | 3.44654000 | 0.50130500 | 1.37128000 |
| H | 4.21473900 | 1.65976400 | 1.19199200 |
| H | 4.13675500 | -0.10640400 | 2.42868600 |
| H | 3.95507300 | -0.26475300 | 0.29793600 |
| P | -0.78668600 | 1.64187000 | 2.68188900 |
| H | -1.04811700 | 1.12434900 | 3.95785700 |
| H | -0.85728500 | 3.01732800 | 2.94040600 |
| H | -2.06338400 | 1.45317800 | 2.10571700 |
| Cl | 2.05775200 | 1.98009700 | 3.58671500 |

$[(NHP)(PH_3)_2RhCl]^+$, **15b**

| | | | |
|---|---|---|---|
| Rh | 1.28380800 | 1.01254200 | 1.92573000 |
| P | 3.49248100 | 0.34662800 | 1.73551200 |
| H | 4.40095600 | 1.40778700 | 1.63633200 |
| H | 3.97794800 | -0.34200800 | 2.85413900 |
| H | 3.98176600 | -0.48556500 | 0.71000200 |
| P | -0.65092100 | 2.01939600 | 2.70133500 |
| H | -0.99014900 | 1.66549900 | 4.01312700 |
| H | -0.56969600 | 3.41446200 | 2.79342200 |
| H | -1.91059000 | 1.89191400 | 2.08453200 |
| Cl | 2.15622500 | 2.09825300 | 3.78640000 |
| N | 1.10833700 | -0.94114700 | -0.92693400 |
| C | -1.03337900 | -0.88395500 | -1.57977800 |
| C | 0.18293800 | -1.37381500 | -1.86139200 |
| H | 2.07659800 | -1.21240000 | -0.98218600 |
| H | -1.95587100 | -1.03615400 | -2.11336800 |
| H | 0.47381200 | -2.01465500 | -2.67599300 |
| N | -0.98780800 | -0.09695800 | -0.44164100 |
| H | -1.81308400 | 0.35403100 | -0.08148700 |
| P | 0.50081700 | 0.03412300 | 0.25561000 |

$[(PR_3)(PH_3)_2RhCl]^{3+}$, **15c**

| | | | |
|---|---|---|---|
| Rh | 0.65163800 | 0.17458000 | 1.67670200 |

| | | | |
|---|---|---|---|
| P | 1.96586200 | -1.55376100 | 2.45920400 |
| H | 3.20532800 | -1.17575400 | 2.99472300 |
| H | 1.41481800 | -2.25499200 | 3.54009200 |
| H | 2.38163300 | -2.64771100 | 1.66836100 |
| P | -0.67255700 | 2.06574800 | 1.68047100 |
| H | -1.74144700 | 2.01529700 | 2.58646800 |
| H | -0.04409100 | 3.24848400 | 2.09376900 |
| H | -1.36012500 | 2.55645300 | 0.54759300 |
| Cl | 0.88136200 | 0.82554600 | 3.90154300 |
| P | 0.44770100 | -0.32131500 | -0.40662900 |
| C | -1.23043800 | -0.15439200 | -1.07983400 |
| C | -2.21505800 | 0.41620900 | -1.86089800 |
| C | -2.54762100 | -0.28269000 | -0.68498900 |
| N | -2.57354700 | 1.14161600 | -2.87507800 |
| N | -3.41350200 | -0.71777800 | 0.17479300 |
| C | -2.97715600 | -1.34416400 | 1.42440600 |
| H | -3.37908500 | -0.78327800 | 2.26861200 |
| H | -3.35460500 | -2.36578600 | 1.47306500 |
| H | -1.88956500 | -1.33403200 | 1.48783900 |
| C | -4.85804300 | -0.55743300 | -0.00421900 |
| H | -5.35299100 | -1.47014100 | 0.32396500 |
| H | -5.22431900 | 0.27676500 | 0.59488500 |
| H | -5.09204500 | -0.39843000 | -1.05275400 |
| C | -3.96790500 | 1.49855100 | -3.14271900 |
| H | -4.03194400 | 2.57222600 | -3.31651300 |
| H | -4.32604000 | 0.97177700 | -4.02755200 |
| H | -4.58842200 | 1.24954600 | -2.28816200 |
| C | -1.58662800 | 1.64966200 | -3.81982600 |
| H | -0.59951500 | 1.28787800 | -3.54422900 |
| H | -1.83420800 | 1.30896600 | -4.82592600 |
| H | -1.59007400 | 2.74012900 | -3.80712700 |
| C | 0.88797400 | -1.97846500 | -1.00117400 |

| | | | |
|---|---|---|---|
| C | 0.59999600 | -3.30975300 | -1.21987500 |
| C | 1.93015800 | -2.86664400 | -1.15495000 |
| N | -0.26788800 | -4.26709400 | -1.33839600 |
| N | 3.21012400 | -3.08638300 | -1.17777000 |
| C | 0.11486500 | -5.66631900 | -1.53132400 |
| C | -1.69886900 | -3.99196200 | -1.27804900 |
| C | 4.14998600 | -2.02288900 | -0.83502600 |
| C | 3.78374000 | -4.40727800 | -1.44036900 |
| H | -0.40957700 | -6.28221200 | -0.80132600 |
| H | -0.15698100 | -5.99366100 | -2.53510800 |
| H | 1.18282200 | -5.78636200 | -1.38099000 |
| H | -1.86121000 | -2.92237100 | -1.17087200 |
| H | -2.17815000 | -4.34116900 | -2.19322100 |
| H | -2.13820600 | -4.51616300 | -0.42861100 |
| H | 4.76626200 | -2.33901700 | 0.00768700 |
| H | 4.80181400 | -1.81374600 | -1.68389900 |
| H | 3.60441300 | -1.12334700 | -0.55537800 |
| H | 4.67388800 | -4.28637400 | -2.05591400 |
| H | 4.06354900 | -4.89459900 | -0.50555900 |
| H | 3.07353400 | -5.02247300 | -1.98490100 |
| C | 1.43438200 | 0.64823400 | -1.58264300 |
| C | 2.01913300 | 0.86971600 | -2.81039700 |
| C | 2.34702500 | 1.68027500 | -1.69887200 |
| N | 2.16553100 | 0.53099400 | -4.05462000 |
| N | 3.02569400 | 2.64385300 | -1.16932600 |
| C | 3.08346100 | 1.21185200 | -4.96915800 |
| C | 1.40418000 | -0.58329900 | -4.61142300 |
| C | 2.95500800 | 2.91187400 | 0.26912400 |
| C | 3.91315900 | 3.51221700 | -1.94500600 |
| H | 3.69229800 | 0.46755600 | -5.48147000 |
| H | 2.52120800 | 1.78092500 | -5.70983800 |
| H | 3.73929300 | 1.87561400 | -4.41541300 |

| | | | |
|---|---|---|---|
| H | 0.71110900 | -0.96570400 | -3.86441000 |
| H | 0.84732800 | -0.24645200 | -5.48645200 |
| H | 2.08365000 | -1.37911200 | -4.91869500 |
| H | 3.96683600 | 2.94744600 | 0.67231300 |
| H | 2.47544700 | 3.87635000 | 0.43904900 |
| H | 2.39687000 | 2.12085900 | 0.76954500 |
| H | 3.74100000 | 4.54451800 | -1.64340800 |
| H | 4.95458900 | 3.25578800 | -1.74965100 |
| H | 3.69702100 | 3.42336700 | -3.00518800 |

[(SMe$_3$)PtMe]$^{2+}$, **16a**

| | | | |
|---|---|---|---|
| P | 1.86964500 | -2.45779900 | -0.54686800 |
| H | 2.94950500 | -3.35653200 | -0.56020200 |
| H | 0.97180000 | -3.11489500 | 0.30453300 |
| H | 1.31065000 | -2.68349500 | -1.81221800 |
| P | 1.74959600 | 2.04517600 | 0.44148200 |
| H | 2.78482800 | 2.91482700 | 0.81816200 |
| H | 1.16562300 | 2.74910200 | -0.62043800 |
| H | 0.82713900 | 2.23677500 | 1.47819500 |
| Pt | 2.15623200 | -0.19932900 | -0.03375700 |
| C | 0.06835200 | -0.24809600 | -0.05074200 |
| H | -0.25462600 | -0.49051900 | 0.96300100 |
| H | -0.37084600 | 0.70342100 | -0.34878100 |
| H | -0.32207700 | -0.99529700 | -0.74119100 |
| S | 4.55679700 | -0.20891700 | 0.01858900 |
| C | 5.38825200 | 1.34089100 | 0.34932500 |
| H | 6.46538000 | 1.18027100 | 0.33223300 |
| H | 5.09068400 | 2.04724300 | -0.42440900 |
| H | 5.06561500 | 1.68579500 | 1.33057000 |
| C | 5.24979500 | -1.30324400 | 1.25456600 |
| H | 4.89515800 | -2.31011600 | 1.04218100 |
| H | 6.33711400 | -1.25602500 | 1.21122900 |
| H | 4.87786500 | -0.97421700 | 2.22403900 |
| C | 5.31295500 | -0.74655500 | -1.51262200 |
| H | 4.97701300 | -0.06826700 | -2.29592700 |
| H | 6.39694600 | -0.72475200 | -1.40851700 |
| H | 4.96302300 | -1.75650700 | -1.71871100 |

[(SPh$_3$)PtMe]$^{2+}$, **16b**

| | | | |
|---|---|---|---|
| Rh | 1.20076900 | 0.75134000 | 1.64556500 |
| P | 1.21305800 | -1.14986600 | 2.95930500 |
| H | 2.49371300 | -1.58095600 | 3.33264700 |
| H | 0.61000700 | -1.03026200 | 4.21983200 |
| H | 0.66370400 | -2.38165200 | 2.54808700 |
| P | 1.62733100 | 2.88152700 | 0.85632400 |
| H | 1.00337400 | 3.92191200 | 1.56074000 |
| H | 2.96755000 | 3.27381800 | 0.97333400 |
| H | 1.36931300 | 3.30035300 | -0.46315100 |
| Cl | 2.23928500 | 1.62822600 | 3.52884700 |
| C | 1.01520100 | -1.73272500 | -0.51231900 |
| C | 0.30061200 | -2.85428500 | -0.89968100 |
| C | 2.40210900 | -1.75727500 | -0.41228100 |
| C | 0.99427500 | -4.01695900 | -1.20206200 |
| H | -0.77758400 | -2.84747100 | -0.95046200 |
| C | 3.08053900 | -2.92036400 | -0.73059400 |
| H | 2.93917200 | -0.87718800 | -0.07974800 |
| C | 2.37720100 | -4.05004900 | -1.12475000 |
| H | 0.44385600 | -4.90104700 | -1.49460300 |
| H | 4.15967400 | -2.94491600 | -0.65971700 |
| H | 2.90930400 | -4.96159500 | -1.36271500 |
| C | -1.50545300 | -0.55658200 | 0.09054000 |
| C | -2.27056700 | -0.89087200 | -1.02199000 |
| C | -2.06636100 | -0.47817800 | 1.35472900 |
| C | -3.61121400 | -1.18098400 | -0.84898300 |

| | | | |
|---|---|---|---|
| H | -1.83338000 | -0.91223000 | -2.01145200 |
| C | -3.41245900 | -0.77098300 | 1.51381300 |
| H | -1.46628100 | -0.16119100 | 2.19755100 |
| C | -4.18021600 | -1.12642500 | 0.41735600 |
| H | -4.21593300 | -1.44207200 | -1.70708300 |
| H | -3.85979800 | -0.70658800 | 2.49647300 |
| H | -5.23148600 | -1.34896900 | 0.54433800 |
| C | 0.22034200 | 0.73478100 | -1.62994600 |
| C | 1.03757100 | 0.38010800 | -2.68753000 |
| C | -0.60988500 | 1.84695800 | -1.69166800 |
| C | 1.01715000 | 1.16037700 | -3.83648100 |
| H | 1.67800000 | -0.48870400 | -2.63650800 |
| C | -0.62488400 | 2.60905800 | -2.84496300 |
| H | -1.24338000 | 2.11139800 | -0.85388100 |
| C | 0.19012700 | 2.26735700 | -3.91695300 |
| H | 1.65025300 | 0.89092700 | -4.67115700 |
| H | -1.27405400 | 3.47223500 | -2.90630700 |
| H | 0.17672400 | 2.86736500 | -4.81709900 |
| S | 0.23901400 | -0.16881300 | -0.07469000 |

[(Me-PSP)RhCl]$^{+}$, **17a**

| | | | |
|---|---|---|---|
| S | 1.54610500 | 0.00209600 | 0.09054900 |
| P | -0.44564300 | 2.26532400 | -0.13846800 |
| P | -0.43928700 | -2.26679300 | -0.13855700 |
| C | 1.36252100 | 2.67834800 | -0.30766100 |
| H | 1.51931000 | 3.51384900 | -0.98906800 |
| H | 1.71834400 | 2.99420400 | 0.67447700 |
| C | 1.37006400 | -2.67465900 | -0.30773800 |
| H | 1.72675200 | -2.98956200 | 0.67439300 |
| H | 1.52921800 | -3.50969000 | -0.98917200 |
| C | 2.16043400 | -1.46384900 | -0.79935300 |
| H | 1.95737000 | -1.25214800 | -1.84964600 |
| H | 3.23530600 | -1.56219600 | -0.64878800 |
| C | 2.15629300 | 1.46979100 | -0.79933400 |
| H | 3.23088300 | 1.57116200 | -0.64878400 |
| H | 1.95381400 | 1.25757700 | -1.84963900 |
| C | 2.57485600 | 0.00352400 | 1.56828800 |
| H | 2.31318900 | -0.88211700 | 2.14395300 |
| H | 3.63089100 | 0.00495900 | 1.30274900 |
| H | -0.88886200 | 3.25327000 | 0.75529300 |
| H | -1.00865800 | 2.74917000 | -1.32937800 |
| H | -0.87967600 | -3.25612700 | 0.75507200 |
| H | -1.00090100 | -2.75211400 | -1.32953100 |
| H | 2.31076700 | 0.88848200 | 2.14389500 |
| Rh | -0.56834900 | -0.00089000 | 0.17574000 |
| Cl | -2.86361600 | -0.00394000 | 0.06890500 |

[(aromaticPh-PSP)RhCl]$^{+}$, **17b**

| | | | |
|---|---|---|---|
| Rh | -1.65594000 | 0.26229500 | 0.48951100 |
| Cl | -3.85669300 | 0.72963900 | 0.97280200 |
| S | 0.34239800 | -0.19534200 | -0.11122400 |
| P | -1.31345900 | 2.33947100 | -0.33446900 |
| P | -1.96077500 | -2.01007900 | 0.45344700 |
| H | -1.21601200 | 3.52459400 | 0.41769900 |
| H | -2.16466800 | 2.78817500 | -1.35602800 |
| H | -1.75279200 | -2.92505800 | 1.50363000 |
| H | -3.15671400 | -2.52472500 | -0.06881400 |
| C | 1.57784500 | -0.59099600 | 1.12567900 |
| C | 1.46894000 | 0.10886200 | 2.31928100 |
| C | 2.58104100 | -1.52471300 | 0.91444700 |
| C | 2.40288400 | -0.11975300 | 3.31480100 |
| C | 3.50507500 | -1.74561500 | 1.92331900 |
| H | 2.63977200 | -2.08995100 | -0.00534200 |
| C | 3.41947200 | -1.04280200 | 3.11586800 |

| | | | |
|---|---|---|---|
| H | 2.32773500 | 0.41640000 | 4.25116500 |
| H | 4.28952300 | -2.47594400 | 1.77687000 |
| H | 4.14290400 | -1.22406400 | 3.89974100 |
| C | 0.32635800 | 2.29824300 | -1.15059300 |
| C | 1.10731500 | 1.15353300 | -1.01506900 |
| C | 0.87070800 | 3.38456000 | -1.82694000 |
| C | 2.41797000 | 1.09861600 | -1.46541100 |
| C | 2.15951000 | 3.32464100 | -2.33070200 |
| H | 0.28799500 | 4.28933100 | -1.94704800 |
| C | 2.93693200 | 2.19092100 | -2.13764100 |
| H | 3.04118700 | 0.23637900 | -1.27118300 |
| H | 2.57056900 | 4.17719500 | -2.85481900 |
| H | 3.95572600 | 2.16179000 | -2.49988000 |
| C | -0.73214600 | -2.55570300 | -0.79424300 |
| C | -0.82070200 | -3.75601500 | -1.48527400 |
| C | 0.29226600 | -1.67172900 | -1.14087900 |
| C | 0.06721600 | -4.04588200 | -2.51095600 |
| H | -1.60588400 | -4.46004900 | -1.23958400 |
| C | 1.17605100 | -1.93997400 | -2.16997800 |
| C | 1.05125500 | -3.13603100 | -2.86139600 |
| H | -0.02430100 | -4.97819500 | -3.05202500 |
| H | 1.93879700 | -1.23174300 | -2.45722000 |
| H | 1.72537000 | -3.35096500 | -3.67958000 |
| H | 0.65558300 | 0.80799500 | 2.47135800 |

[(Me-PSP)PtMe]$^{2+}$, **18a**

| | | | |
|---|---|---|---|
| Pt | 0.00025100 | -0.72801700 | 0.13200000 |
| C | 0.00094700 | -2.80950900 | -0.00939300 |
| H | 0.89296400 | -3.18828100 | -0.52037600 |
| H | -0.88964300 | -3.18854400 | -0.52267900 |
| H | -0.00033800 | -3.14653500 | 1.04018400 |
| S | -0.00051500 | 1.58432600 | 0.08301000 |
| P | -2.29183600 | -0.58661700 | -0.15702500 |
| P | 2.29216800 | -0.58517300 | -0.15703900 |
| C | -2.69070800 | 1.23036700 | -0.32720500 |
| H | -3.54863600 | 1.36660800 | -1.00316200 |
| H | -3.00818800 | 1.59034600 | 0.66283200 |
| C | 2.68990800 | 1.23206200 | -0.32716700 |
| H | 3.00711000 | 1.59222200 | 0.66289500 |
| H | 3.54777400 | 1.36886200 | -1.00309000 |
| C | 1.50826300 | 2.04875400 | -0.86296000 |
| H | 1.26713400 | 1.80271400 | -1.90779500 |
| H | 1.66288800 | 3.13323200 | -0.77004200 |
| C | -1.50957100 | 2.04780500 | -0.86298500 |
| H | -1.66487200 | 3.13218700 | -0.77008000 |
| H | -1.26828100 | 1.80189500 | -1.90781400 |
| C | -0.00091600 | 2.68463000 | 1.52135700 |
| H | 0.89377100 | 2.45098600 | 2.11284500 |
| H | -0.00120400 | 3.73113600 | 1.18948800 |
| H | -3.22172100 | -1.05543700 | 0.80466900 |
| H | -2.77456600 | -1.19092700 | -1.34407900 |
| H | 3.22235500 | -1.05349200 | 0.80460600 |
| H | 2.77518500 | -1.18915600 | -1.34414100 |
| H | -0.89557400 | 2.45046100 | 2.11268800 |

[(aromaticPh-PSP)PtMe]$^{2+}$, **18b**

| | | | |
|---|---|---|---|
| S | 0.48364100 | -0.22172100 | -0.12055200 |
| P | -1.29405300 | 2.38861200 | -0.31272400 |
| P | -1.96078200 | -2.03060100 | 0.45950400 |
| H | -1.18196400 | 3.48217100 | 0.56049500 |
| H | -2.15152000 | 2.90436100 | -1.29472800 |
| H | -1.67507600 | -2.80191700 | 1.59800100 |
| H | -3.15289500 | -2.61506000 | 0.01192200 |
| C | 1.66141900 | -0.61949300 | 1.14379500 |

| | | | |
|---|---|---|---|
| C | 1.56411500 | 0.12013700 | 2.31700700 |
| C | 2.62889800 | -1.59650900 | 0.95469400 |
| C | 2.47975400 | -0.12334600 | 3.32481500 |
| C | 3.53261500 | -1.82650100 | 1.97904000 |
| H | 2.67640500 | -2.18159700 | 0.04651500 |
| C | 3.46053200 | -1.09166900 | 3.15423200 |
| H | 2.42167400 | 0.43789800 | 4.24754100 |
| H | 4.29256100 | -2.58661200 | 1.85804900 |
| H | 4.17025400 | -1.28106500 | 3.94872400 |
| C | 0.33933800 | 2.30763900 | -1.12106500 |
| C | 1.14671300 | 1.16944700 | -1.01429700 |
| C | 0.85410400 | 3.40875600 | -1.79534800 |
| C | 2.44739100 | 1.14384700 | -1.49442400 |
| C | 2.13508200 | 3.37553700 | -2.32591800 |
| H | 0.25888400 | 4.30793500 | -1.89731000 |
| C | 2.93471000 | 2.25375700 | -2.16372200 |
| H | 3.08829900 | 0.28806500 | -1.32808000 |
| H | 2.51925200 | 4.24188400 | -2.84799100 |
| H | 3.94556100 | 2.24523300 | -2.54876200 |
| C | -0.73491300 | -2.53613100 | -0.79495600 |
| C | -0.86317700 | -3.72443600 | -1.49979000 |
| C | 0.31804200 | -1.67642200 | -1.14386600 |
| C | 0.01464700 | -4.03237500 | -2.53117300 |
| H | -1.66691300 | -4.41026100 | -1.26223700 |
| C | 1.19020700 | -1.96555900 | -2.17656200 |
| C | 1.02630000 | -3.15255800 | -2.87712400 |
| H | -0.10752800 | -4.95824800 | -3.07742700 |
| H | 1.97770700 | -1.28128800 | -2.45718200 |
| H | 1.69347100 | -3.38383000 | -3.69659600 |
| H | 0.78834700 | 0.86446000 | 2.44885500 |
| C | -3.73545600 | 0.65214700 | 0.79097000 |
| H | -3.97682600 | 1.71240300 | 0.79078300 |
| H | -4.33809900 | 0.15551400 | 0.02982600 |
| H | -3.97963600 | 0.23749100 | 1.77028100 |
| Pt | -1.73063300 | 0.27504800 | 0.43704400 |

## 4. NMR spectra

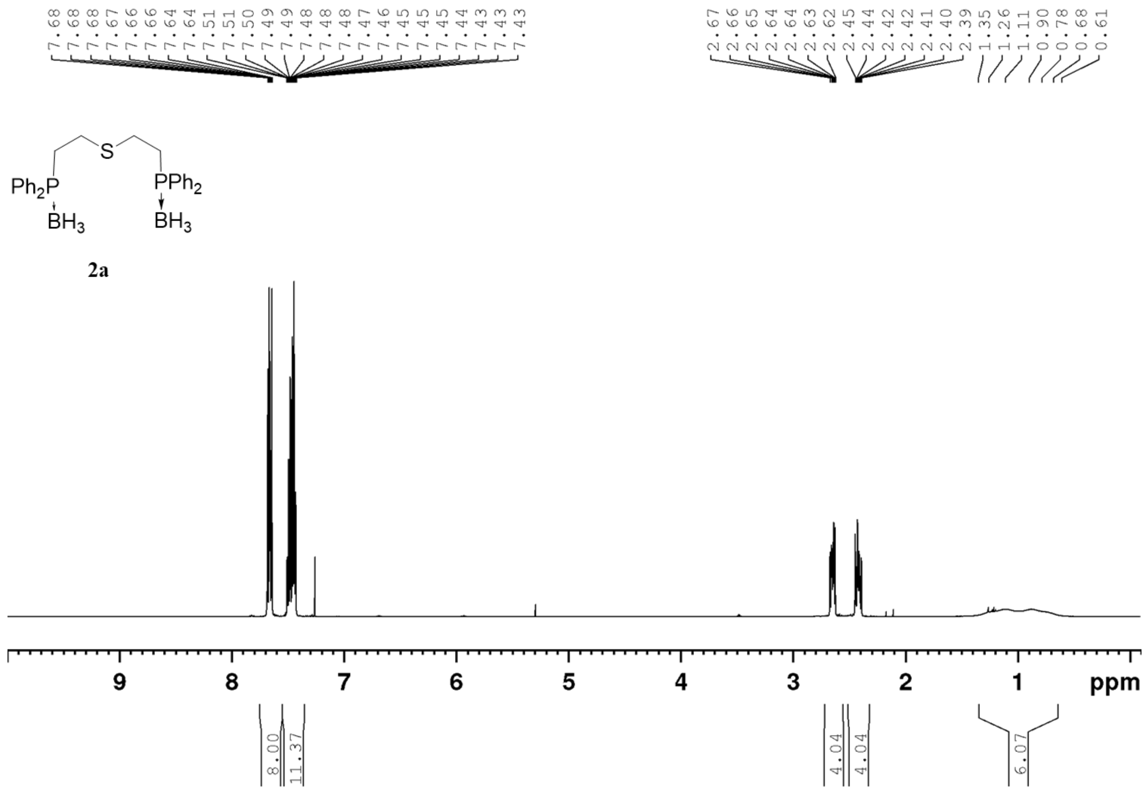


**Figure S20**: $^{1}$H NMR (400 MHz) of **2a** in $CDCl_3$.

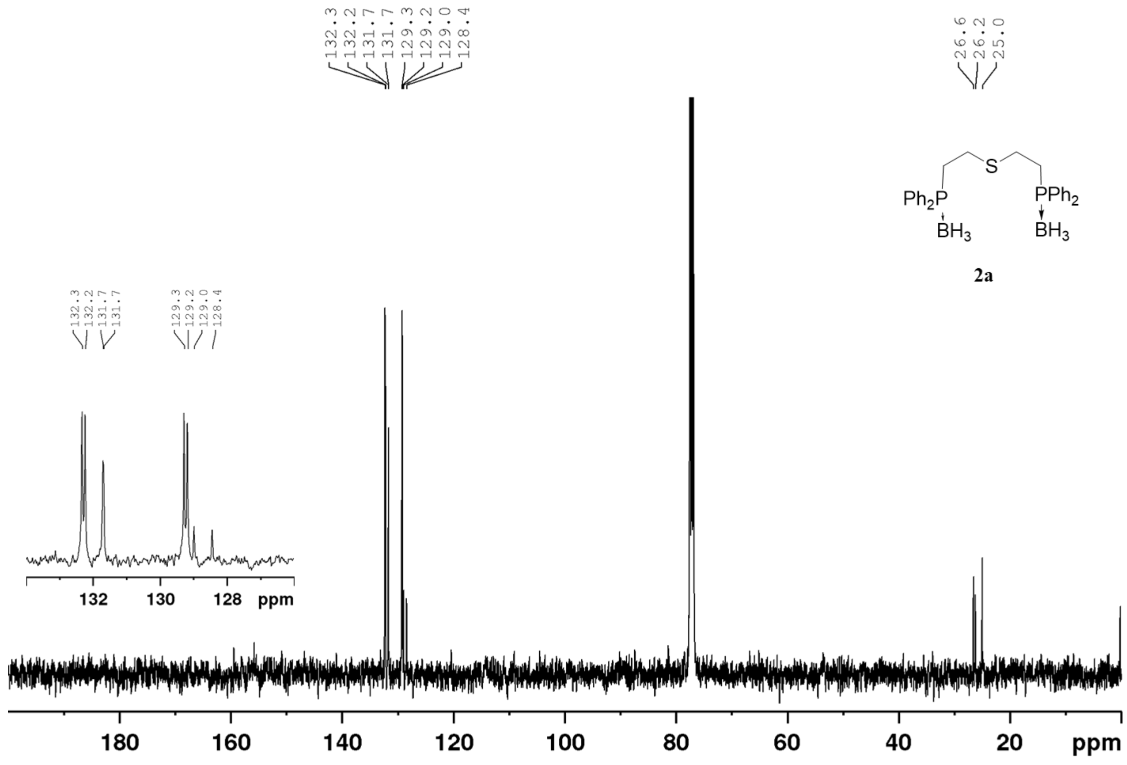


**Figure S21**: $^{13}$C NMR (101 MHz) of **2a** in $CDCl_3$.

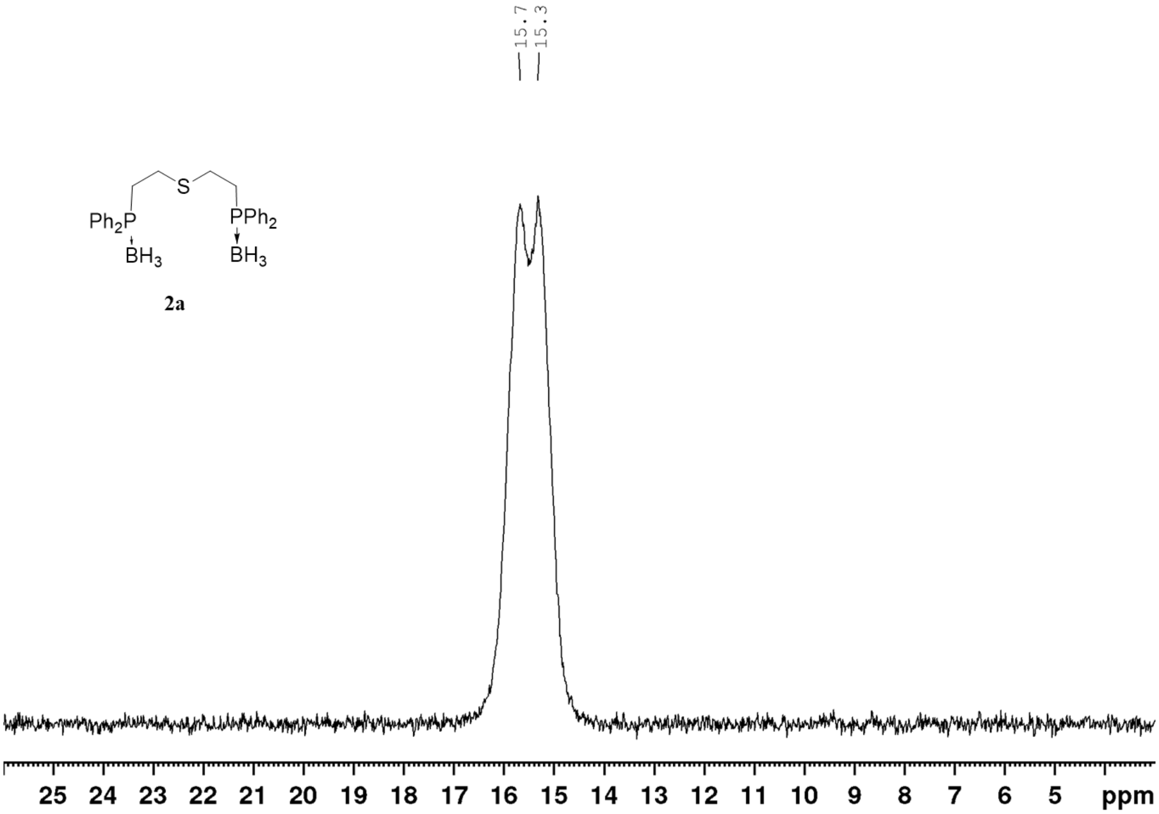


**Figure S22**: $^{31}P\{H\}$ NMR (162 MHz) of **2a** in $CDCl_3$.

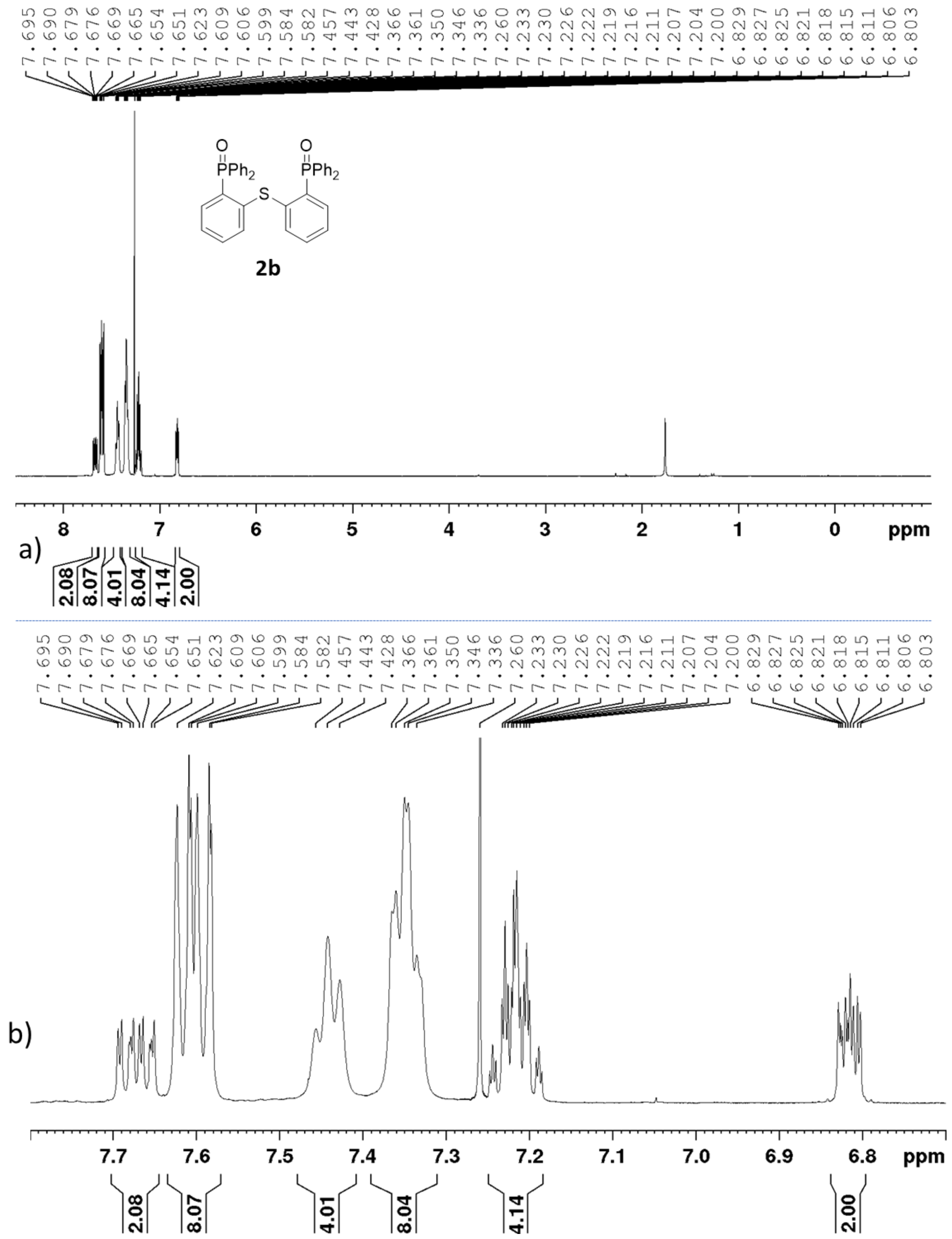


**Figure S23**: $^{1}H$ NMR (500 MHz) of **2b** in $CDCl_3$. a) full spectrum, b) aromatic region expanded.

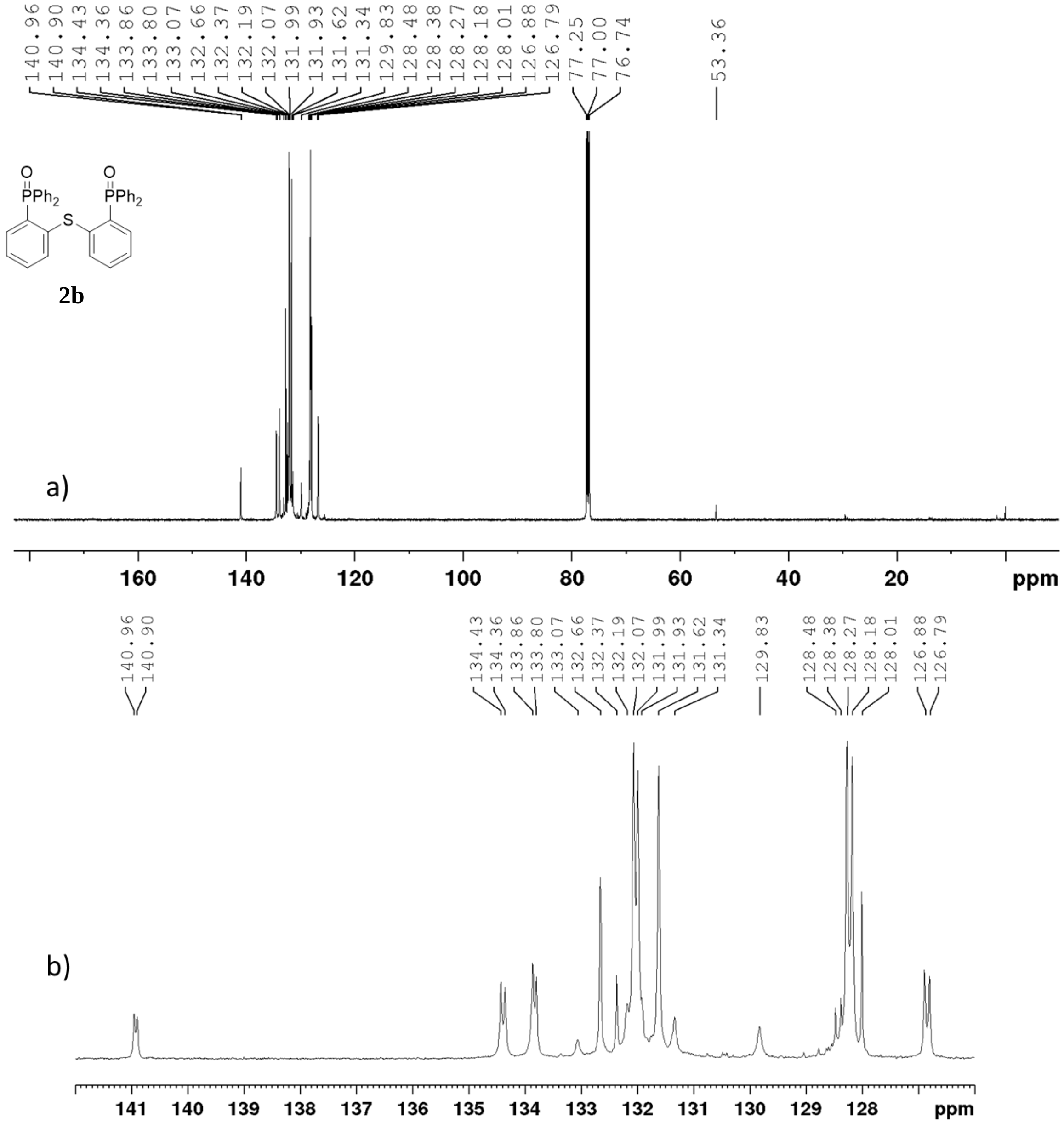


**Figure S24**: $^{13}C$ NMR (126 MHz) of **2b** in $CDCl_3$. a) Full spectrum, b) aromatic region expanded.

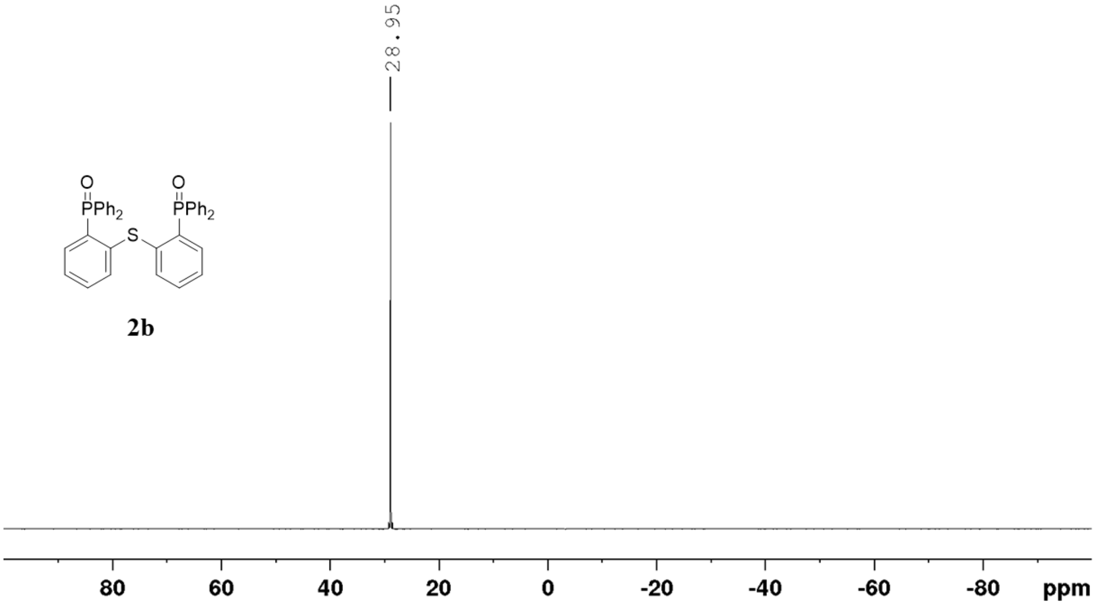


**Figure S25**: $^{31}P\{H\}$ NMR (162 MHz) of **2b** in $CDCl_3$

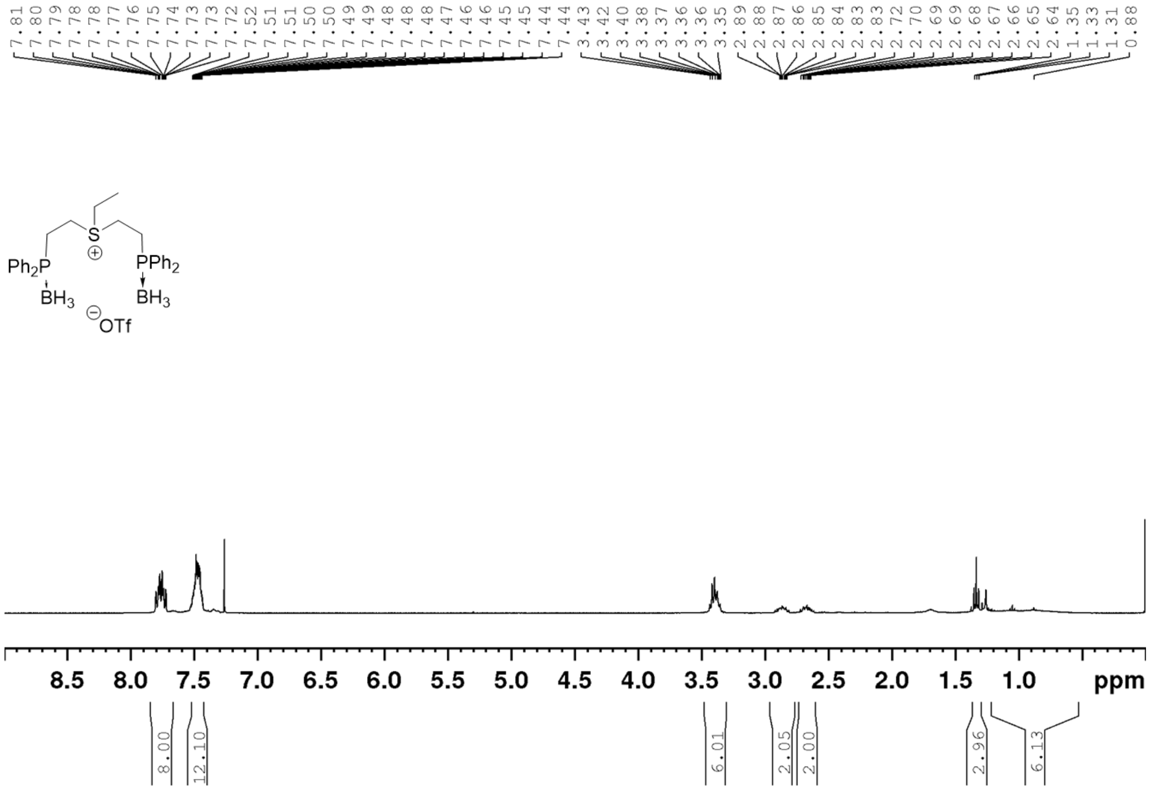


**Figure S26**: $^{1}H$ NMR (400 MHz) of **3a**[OTf] in $CDCl_3$, measured at 298 K.

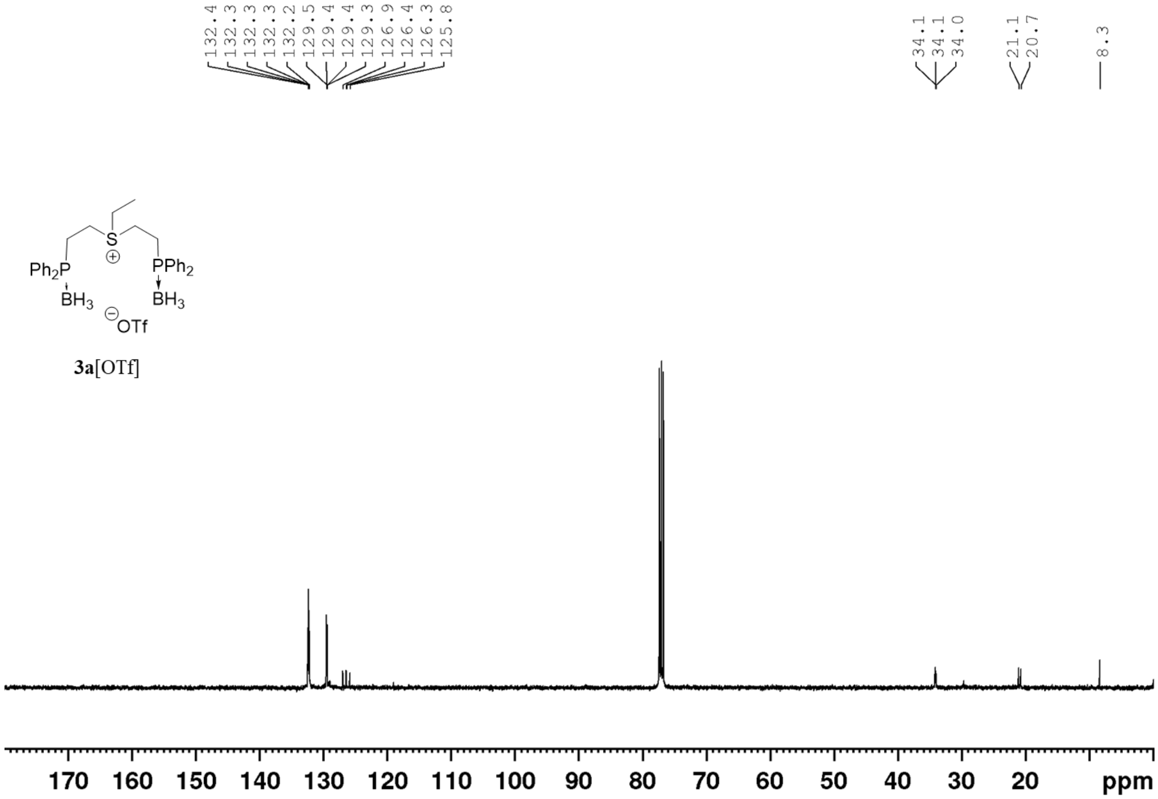


**Figure S27**: $^{13}$C NMR (101 MHz) of **3a**[OTf] in $CDCl_3$.

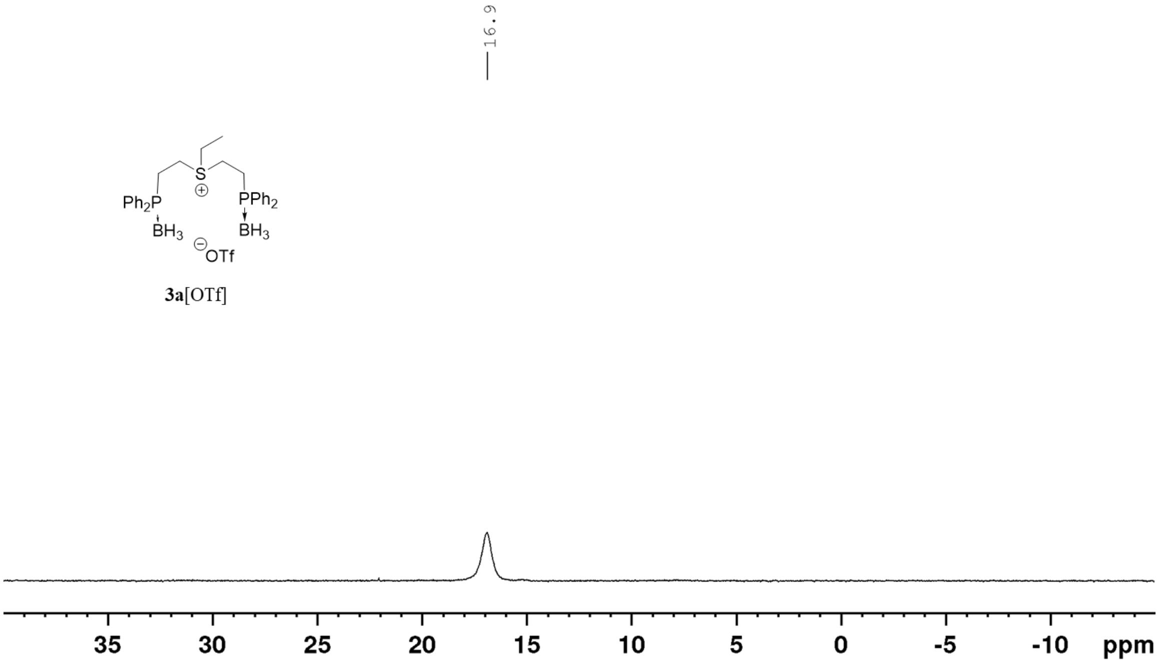


**Figure S28**: $^{31}$P{H} NMR (162 MHz) of **3a**[OTf] in $CDCl_3$.

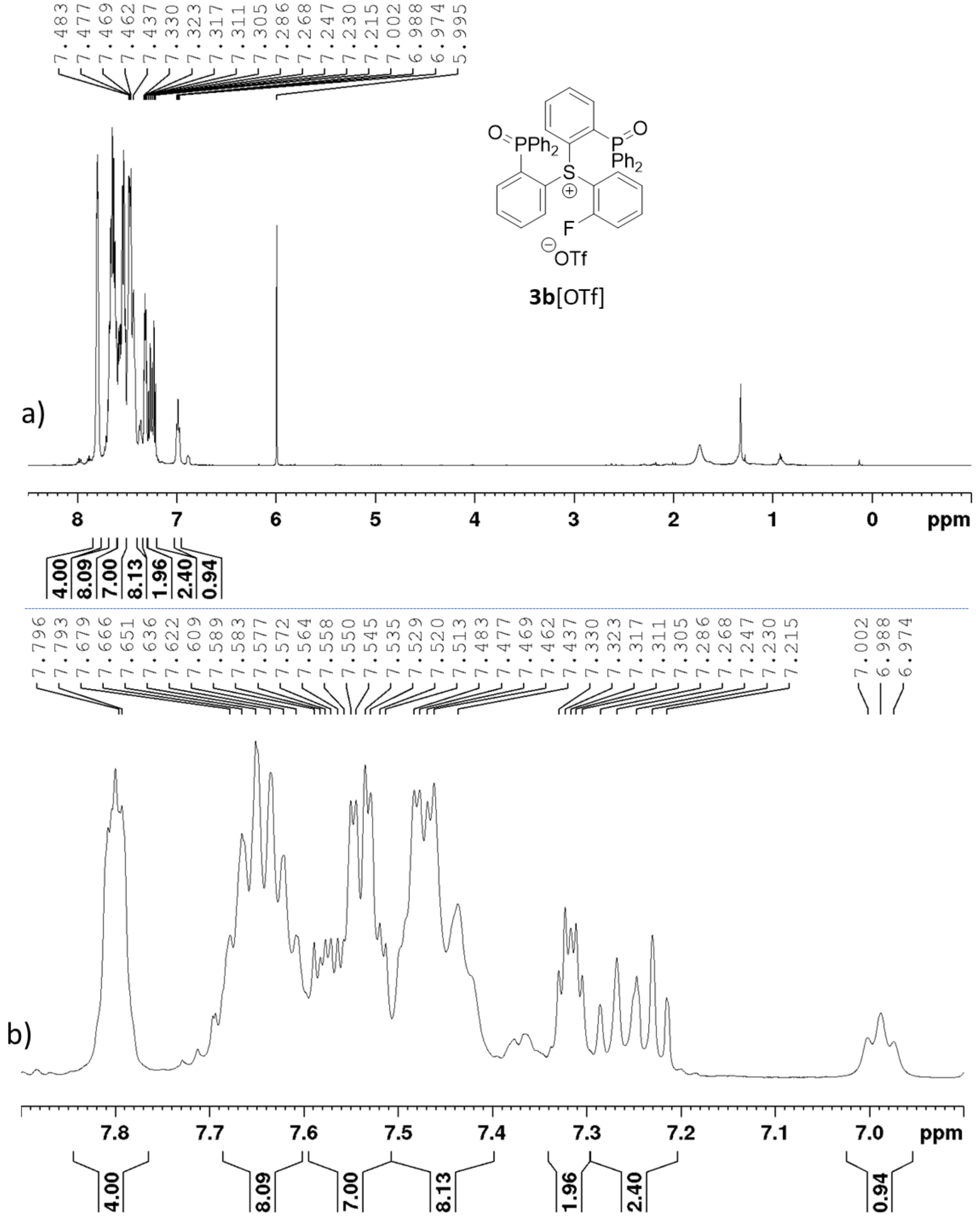


**Figure S29**: $^{1}$H NMR (500 MHz) of **3b**[OTf] in $C_2D_2Cl_4$ at 98.5±1.00 °C. a) Full spectrum, b) aromatic region expanded.

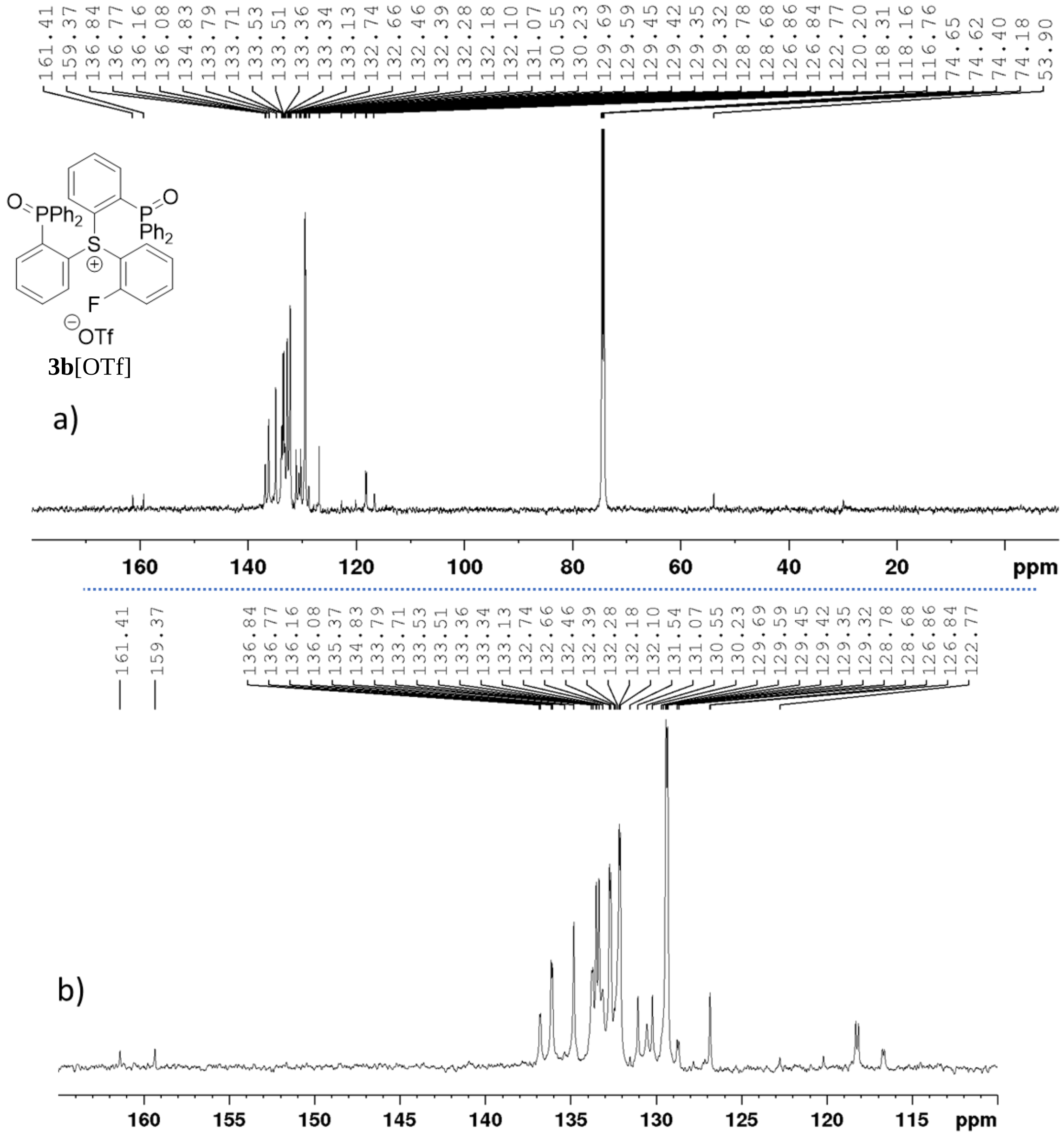


**Figure S30**: $^{13}C$ NMR (126 MHz) of **3b**[OTf] in $C_2D_2Cl_4$ at 98.5±1.00 °C. a) full spectrum, b) aromatic region expanded.

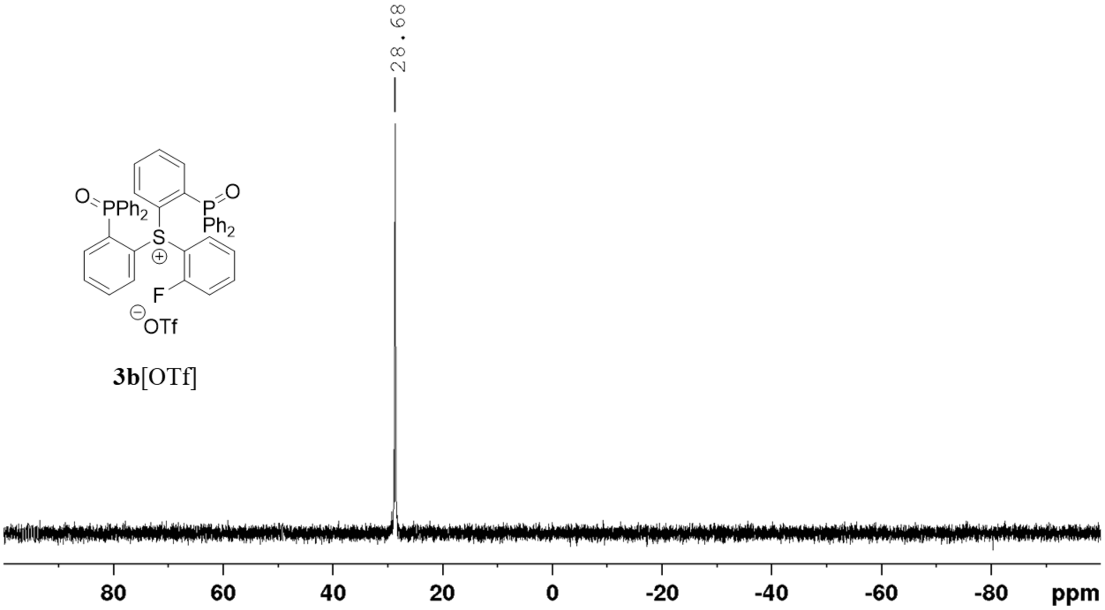


**Figure S31**: $^{31}P\{H\}$ NMR (202 MHz) of **3b**[OTf] in $C_2D_2Cl_4$ at 98.5±1.00 °C.

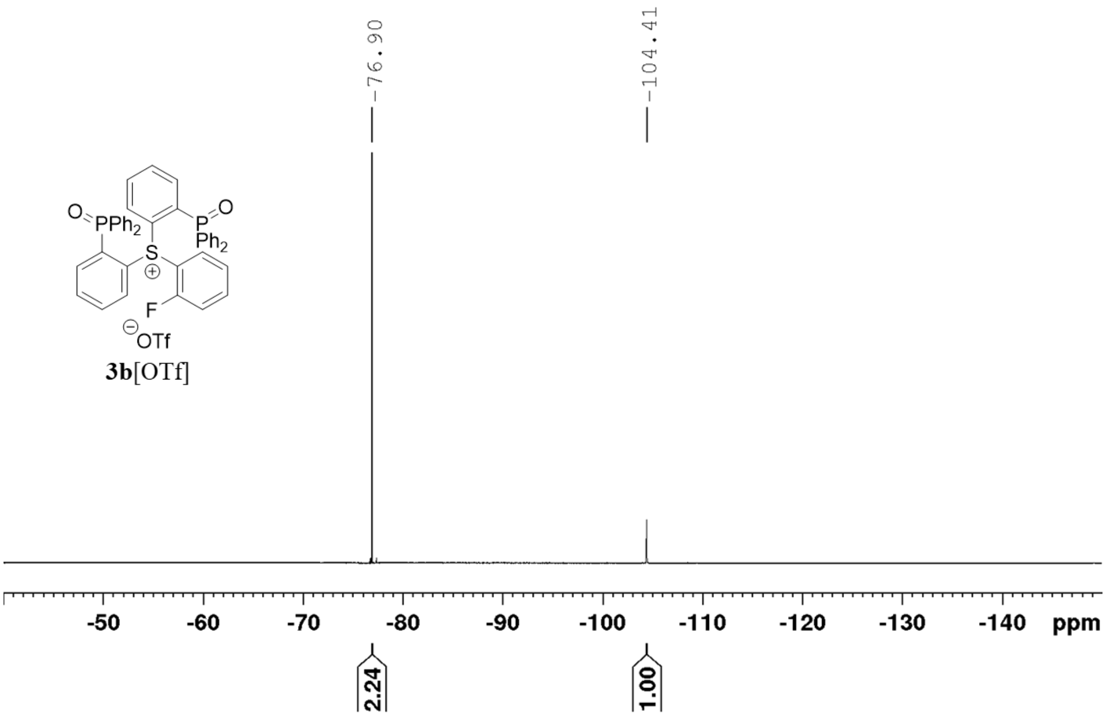


**Figure S32**: $^{19}F\{H\}$ NMR (471 MHz) of **3b**[OTf] in $C_2D_2Cl_4$ at 98.5±1.00 °C.

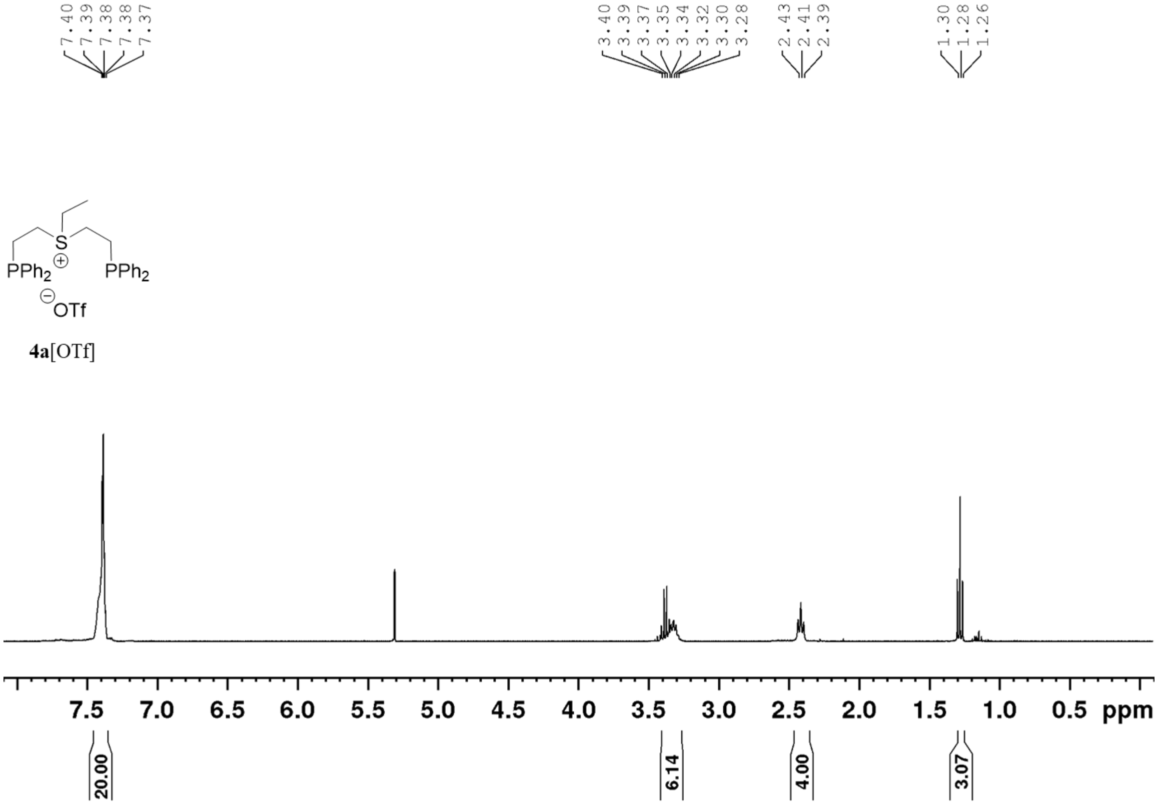


**Figure S33**: $^{1}$H NMR (400 MHz) of **4a**[OTf] in $CD_2Cl_2$.

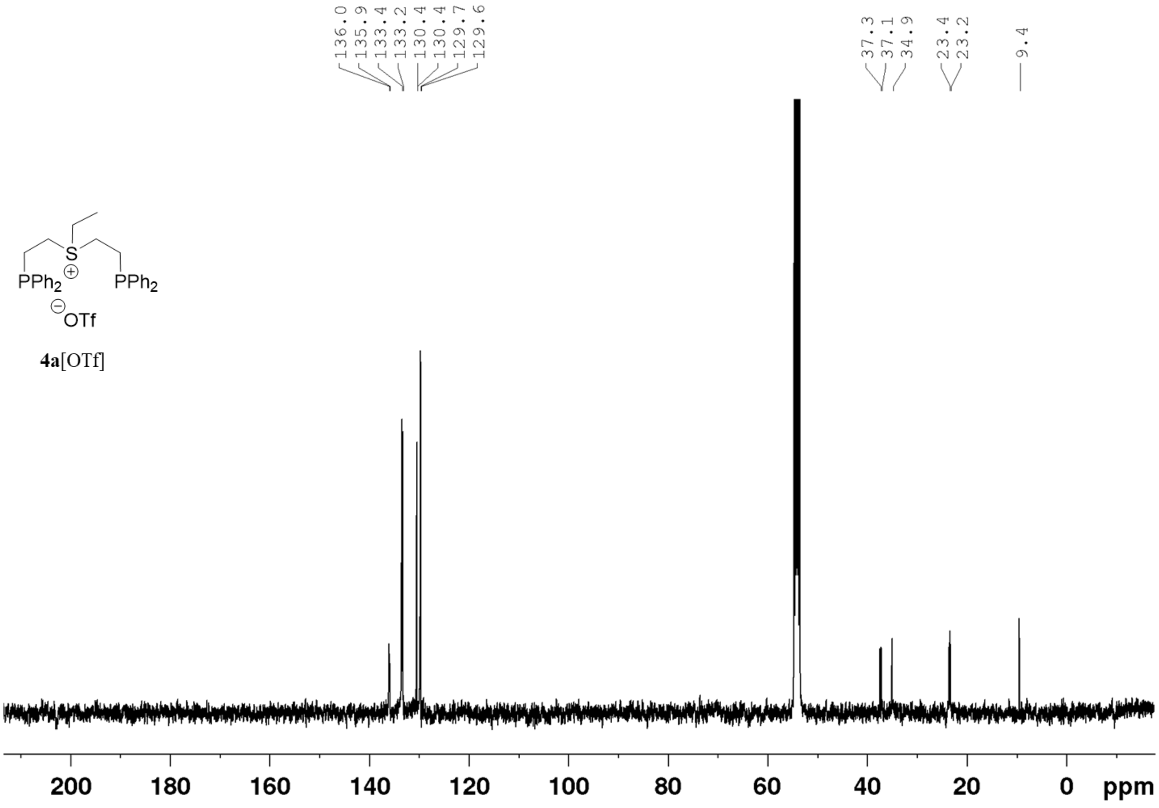


**Figure S34**: $^{13}$C NMR (101 MHz) of **4a**[OTf] in $CD_2Cl_2$.

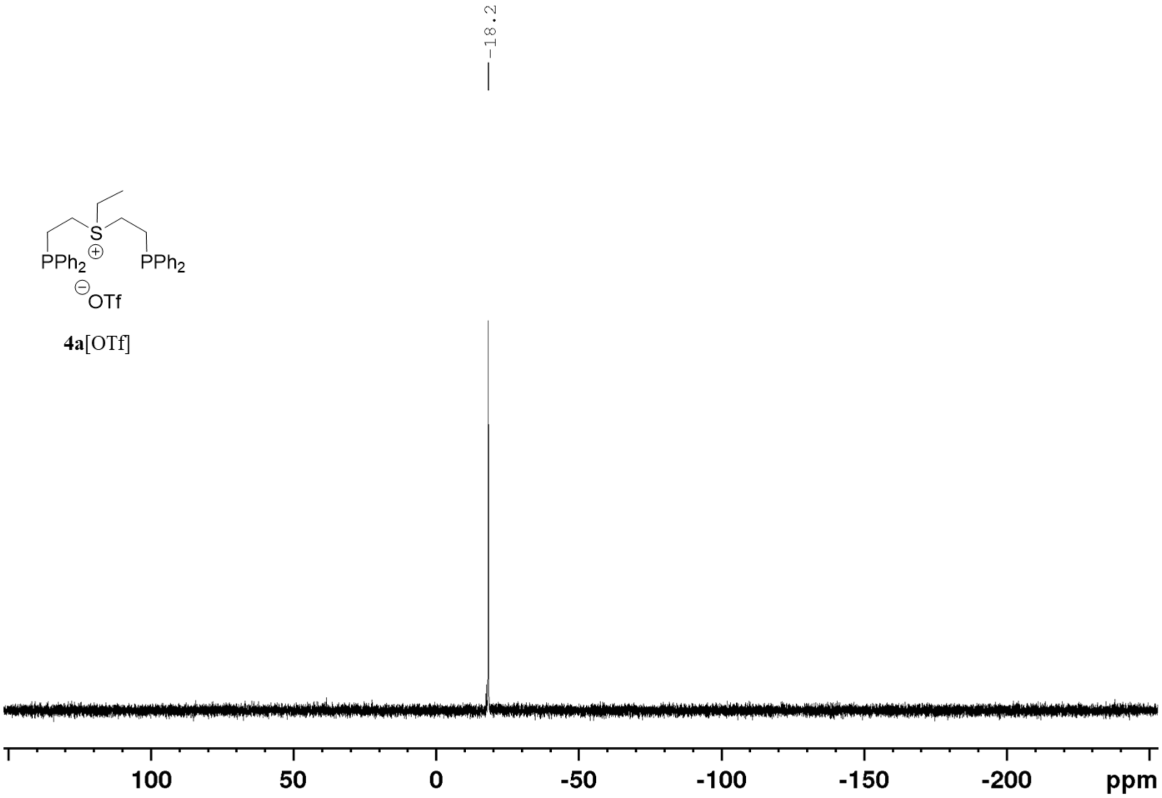


**Figure S35**: $^{31}P\{H\}$ NMR (162 MHz) of **4a**[OTf] in $CD_2Cl_2$.

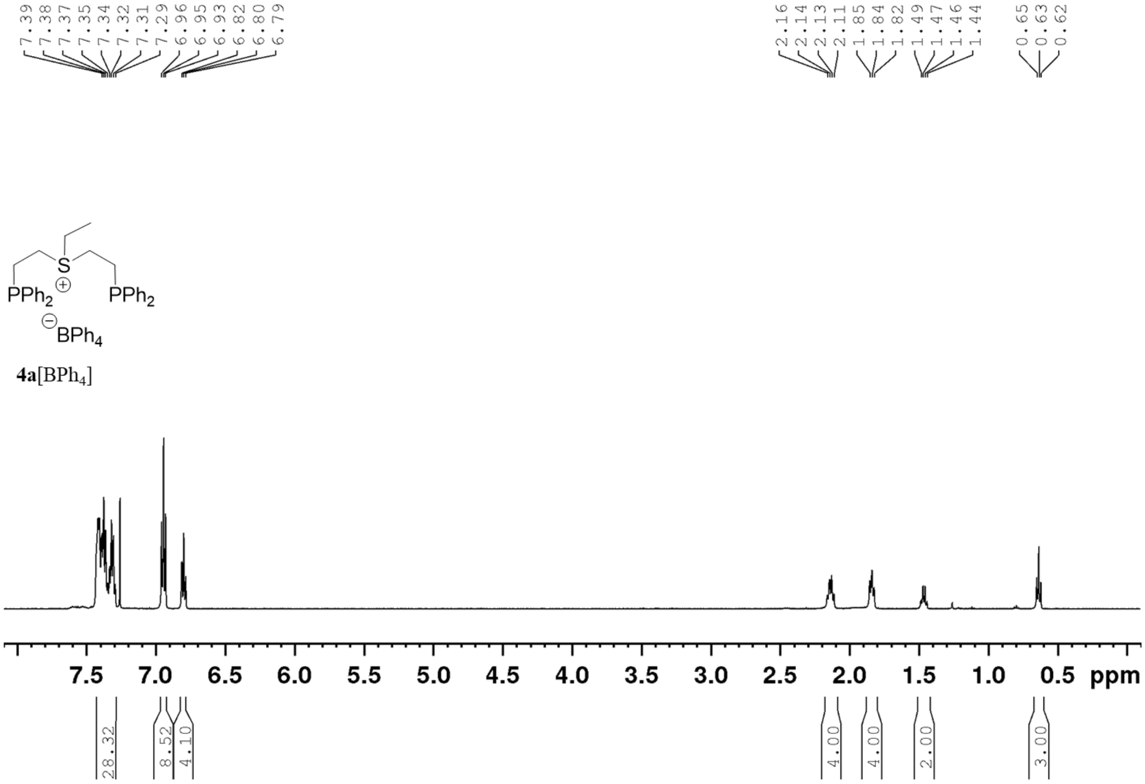


**Figure S36**: $^{1}H$ NMR (400 MHz) of **4a**[$BPh_4$] in $CDCl_3$.

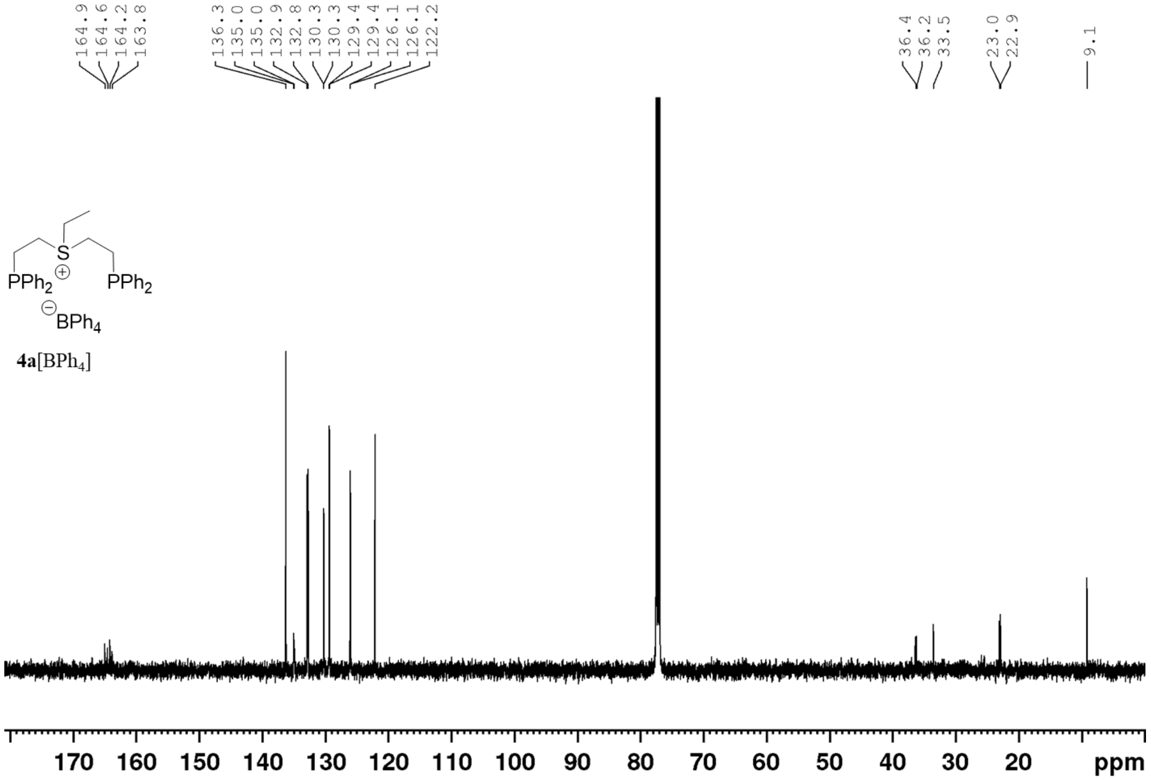


**Figure S37**: $^{13}$C NMR (126 MHz) of **4a**[$BPh_4$] in $CDCl_3$.

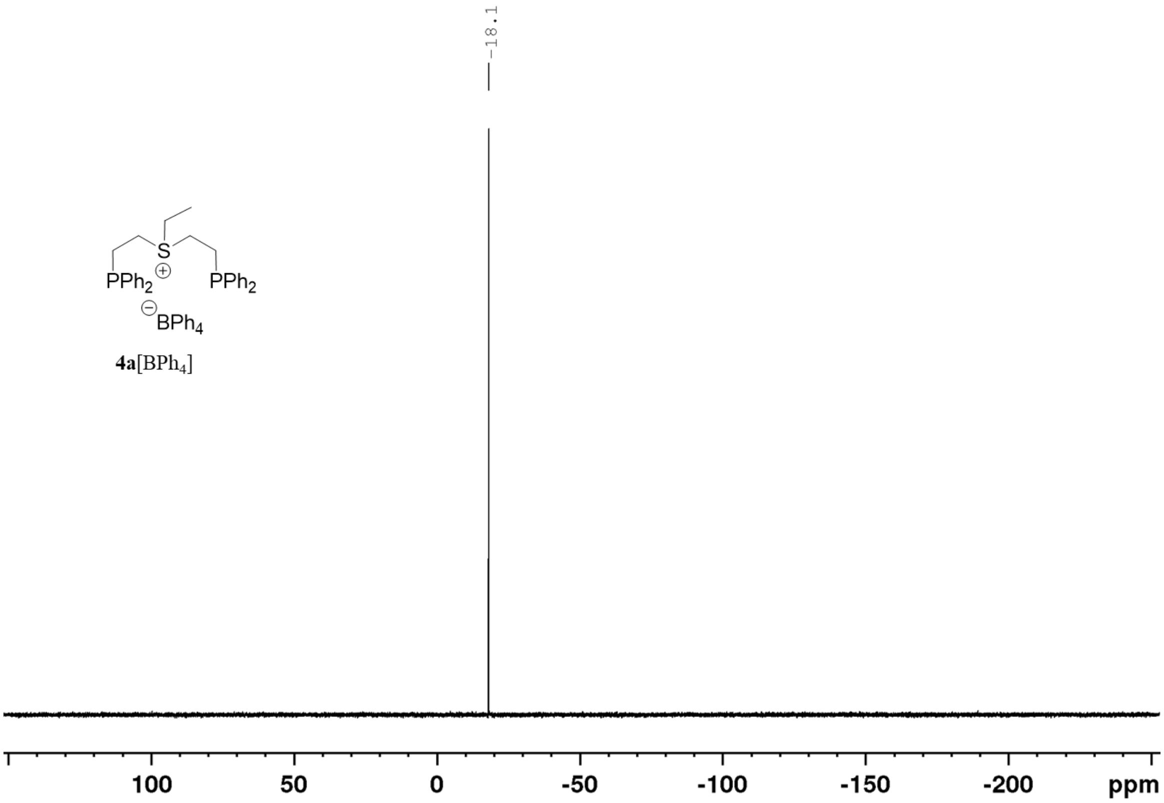


**Figure S38**: $^{31}$P{H} NMR (162 MHz) of **4a**[$BPh_4$] in $CDCl_3$.

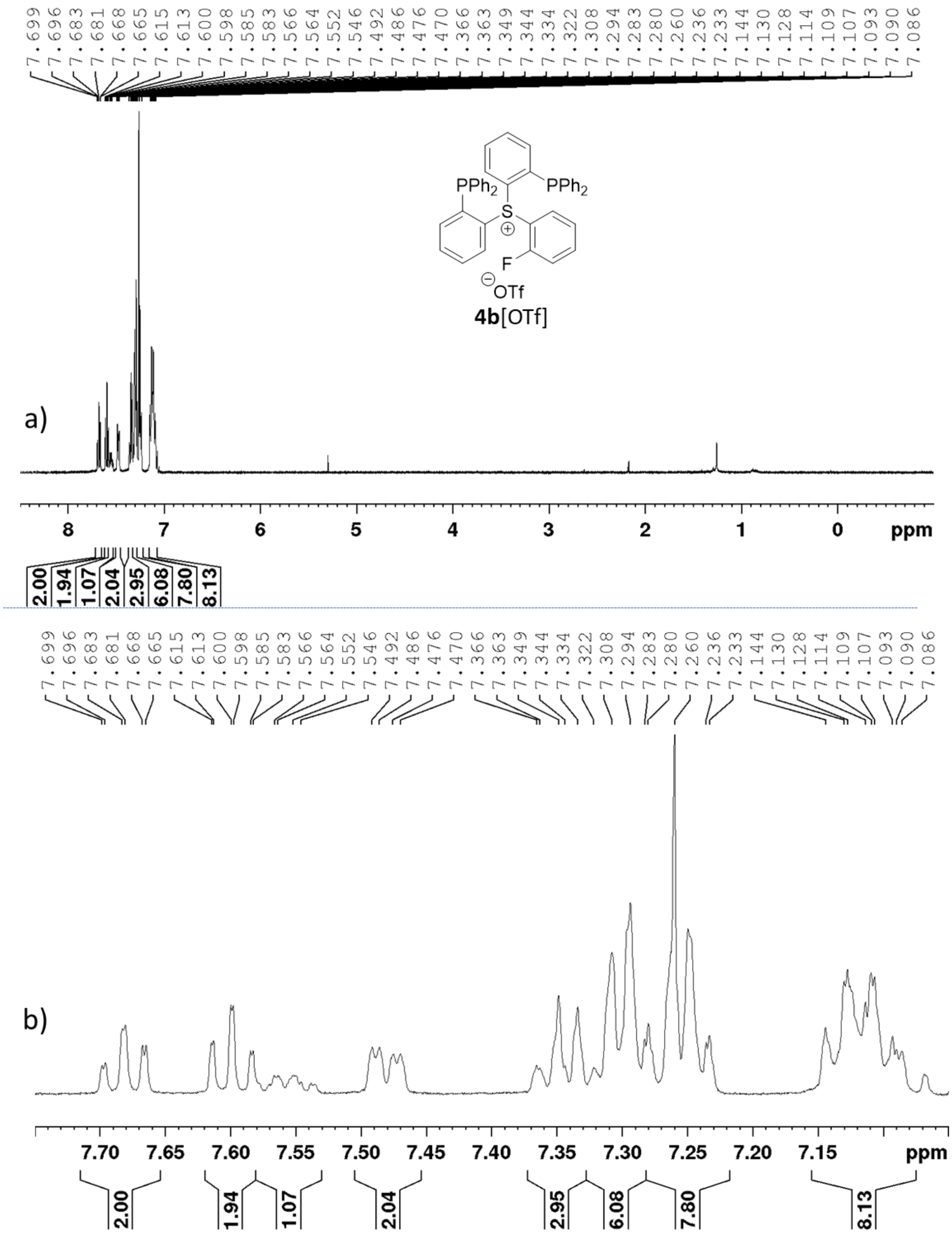


**Figure S39**: $^{1}H$ NMR (500 MHz) of **4b**[OTf] in $CDCl_3$. a) full spectrum, b) aromatic region expanded.

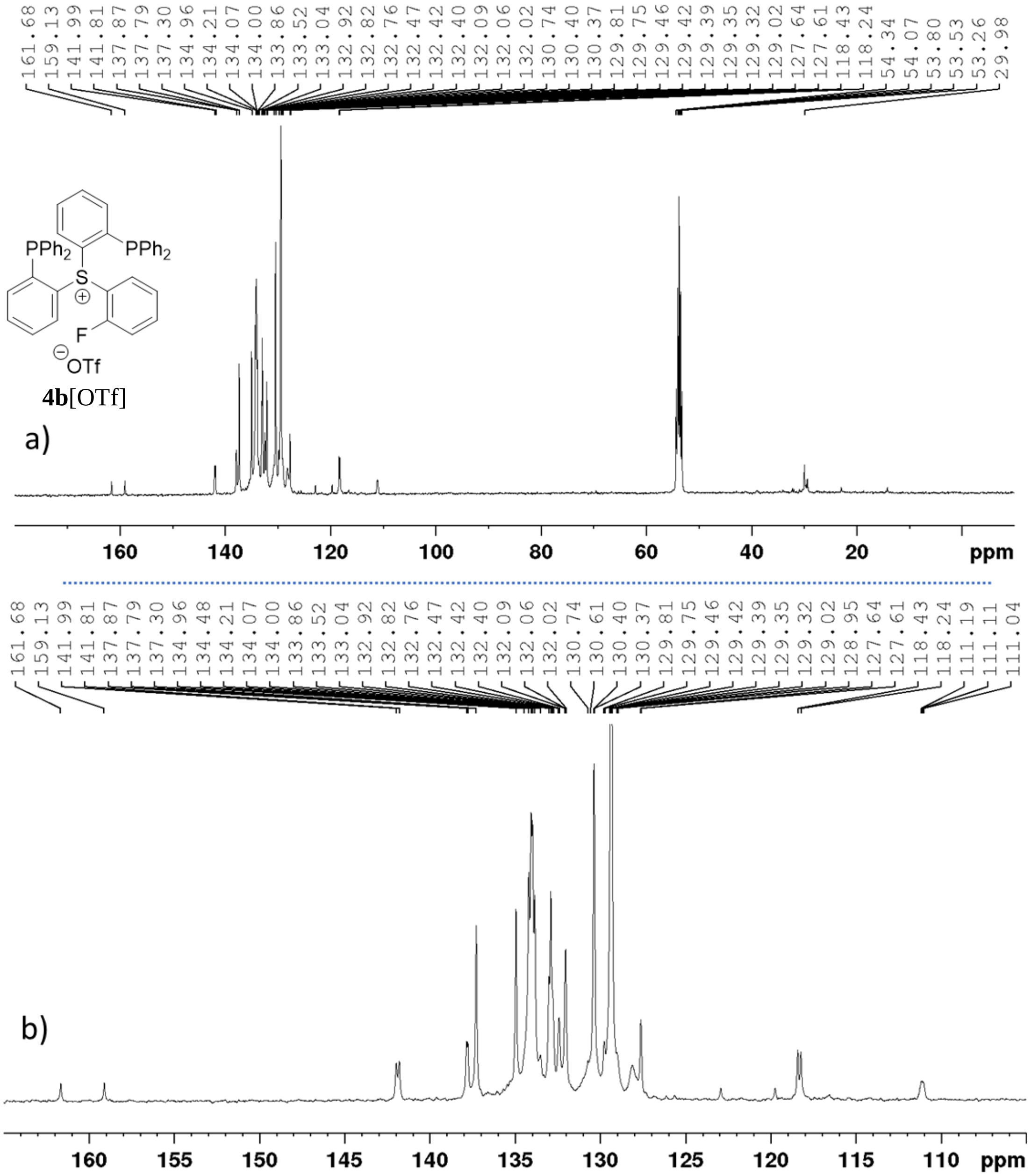


**Figure S40**: $^{31}$C NMR (126 MHz) of **4b**[OTf] in $CDCl_3$. a) full spectrum, b) aromatic region expanded.

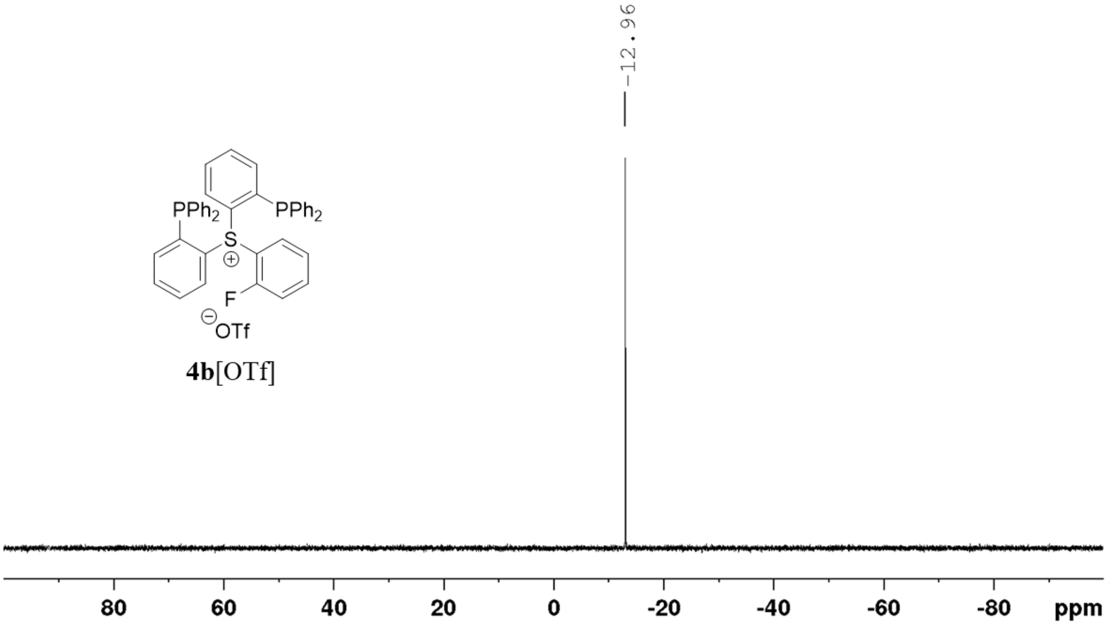


**Figure S41**: $^{31}P\{H\}$ NMR (202 MHz) of **4b**[OTf] in $CDCl_3$.

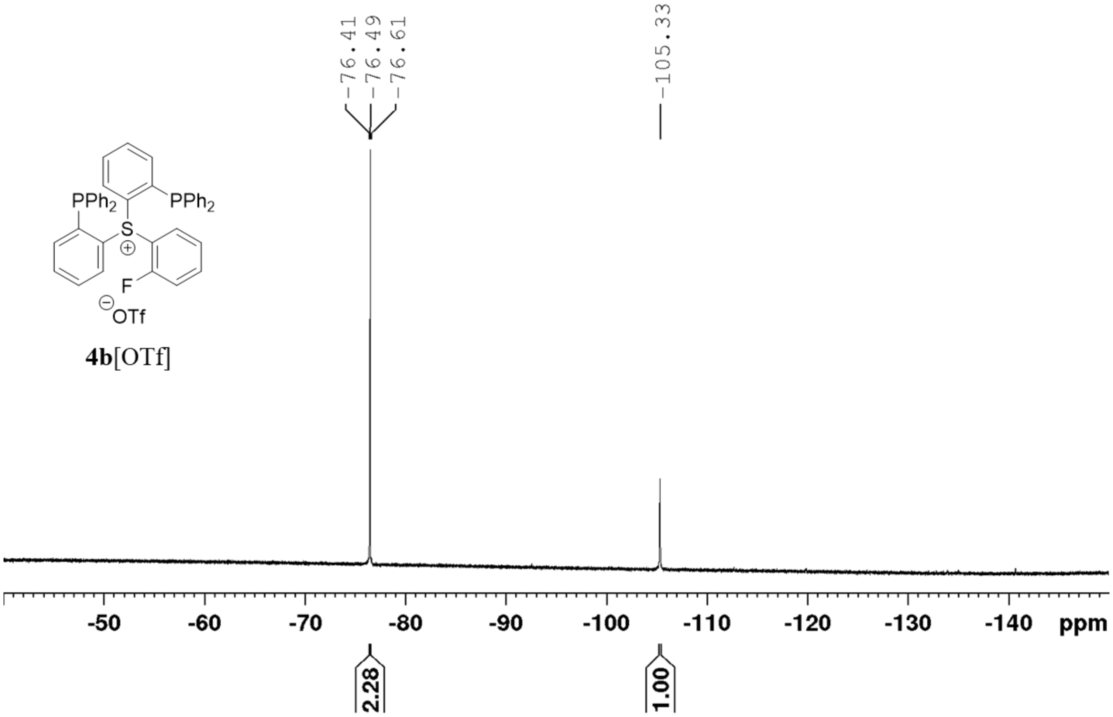


**Figure S42**: $^{19}F\{H\}$ NMR (376 MHz) of **4b**[OTf] in $CDCl_3$.

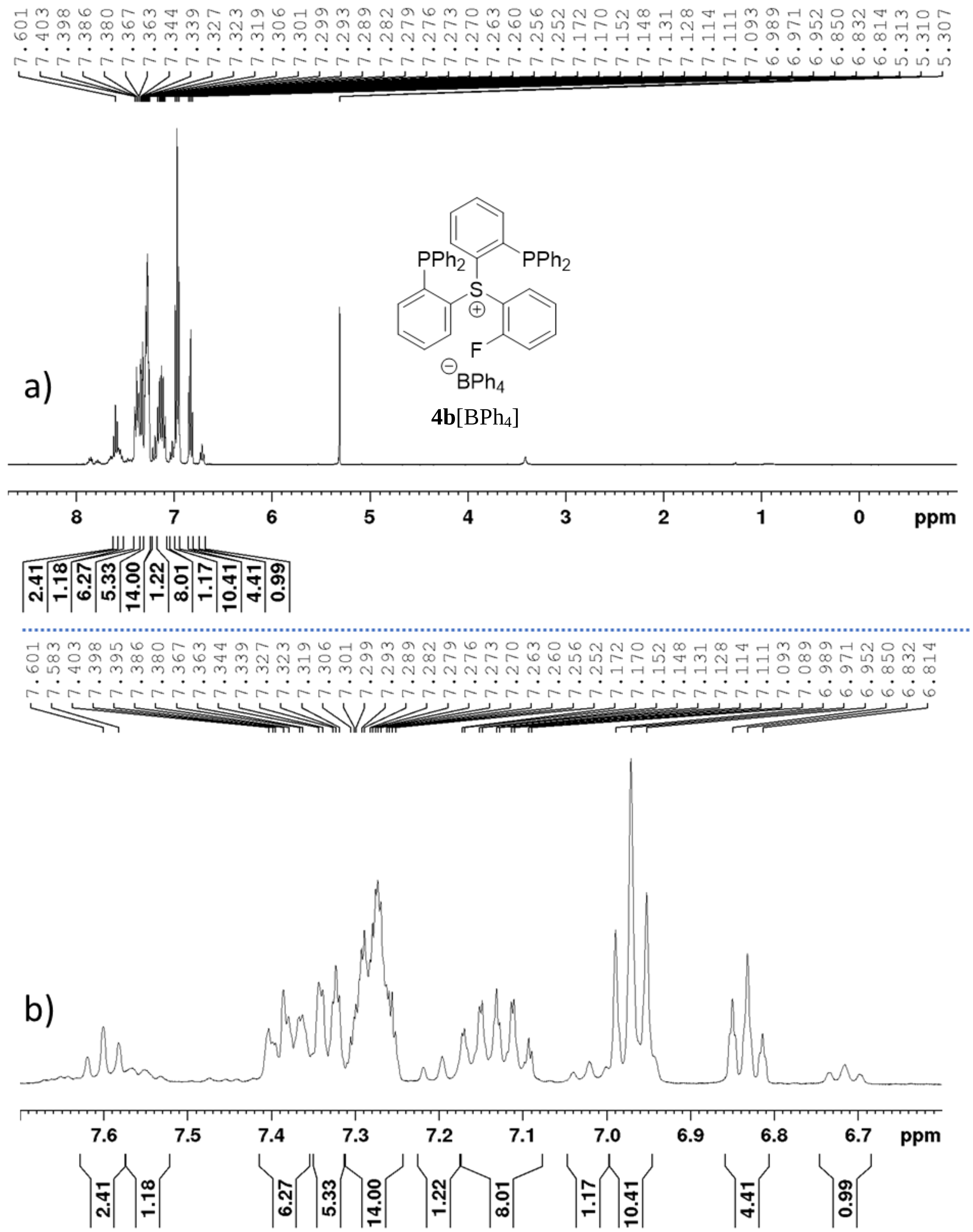

**Figure S43**: $^{1}H$ NMR (500 MHz) of **4b**[$BPh_4$] in $CD_2Cl_2$. a) full spectrum, b) aromatic region expanded.

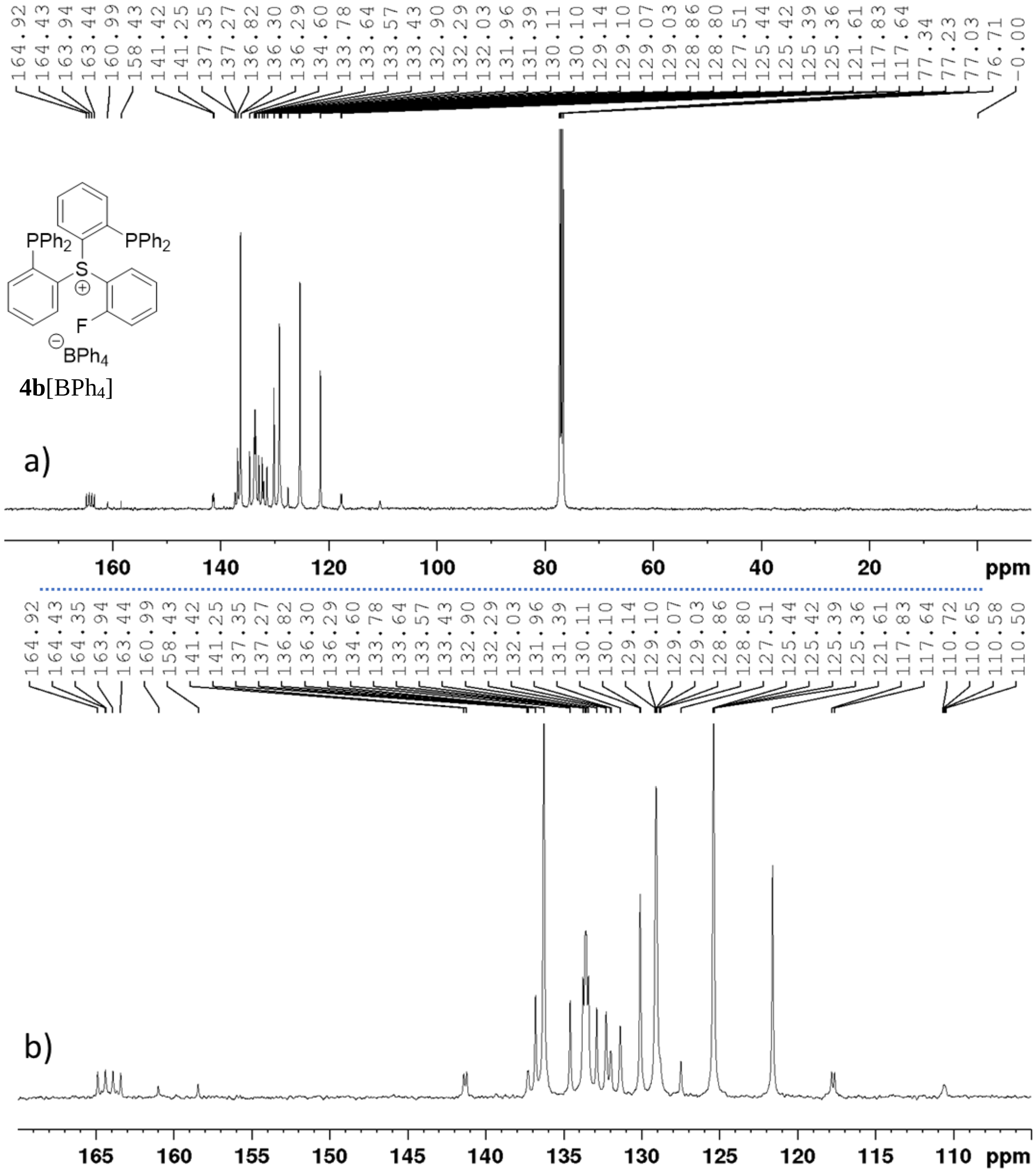


**Figure S44**: $^{13}C$ NMR (101 MHz) of **4b**[$BPh_4$] in $CD_2Cl_2$. a) full spectrum, b) aromatic region expanded.

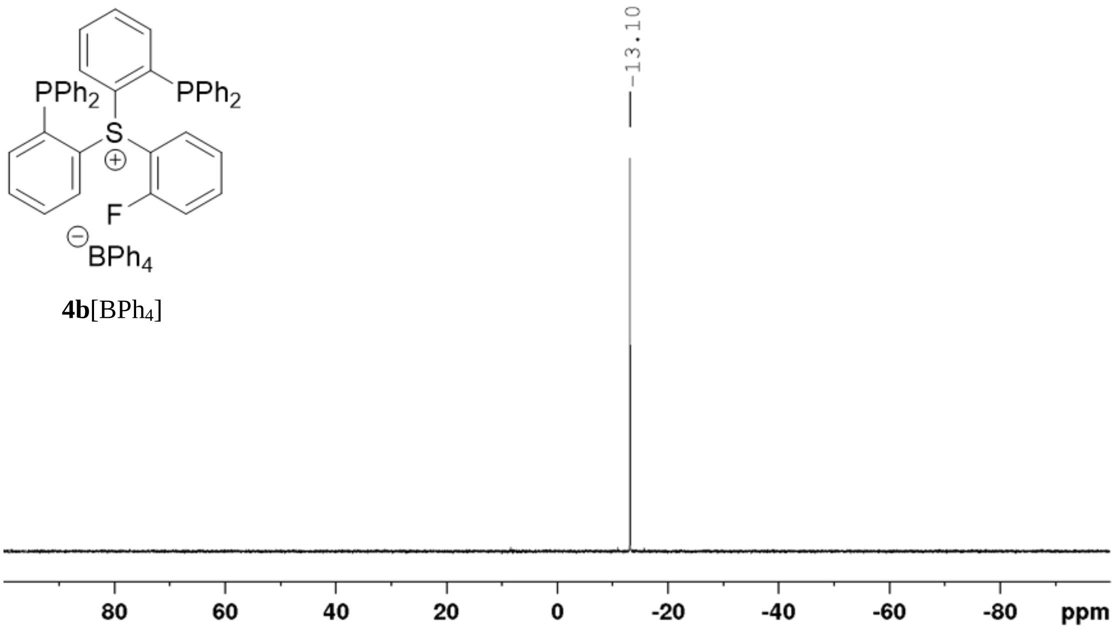


**Figure S45**: $^{31}P\{H\}$ NMR (162 MHz) of **4b**[BPh4] in $CD_2Cl_2$.

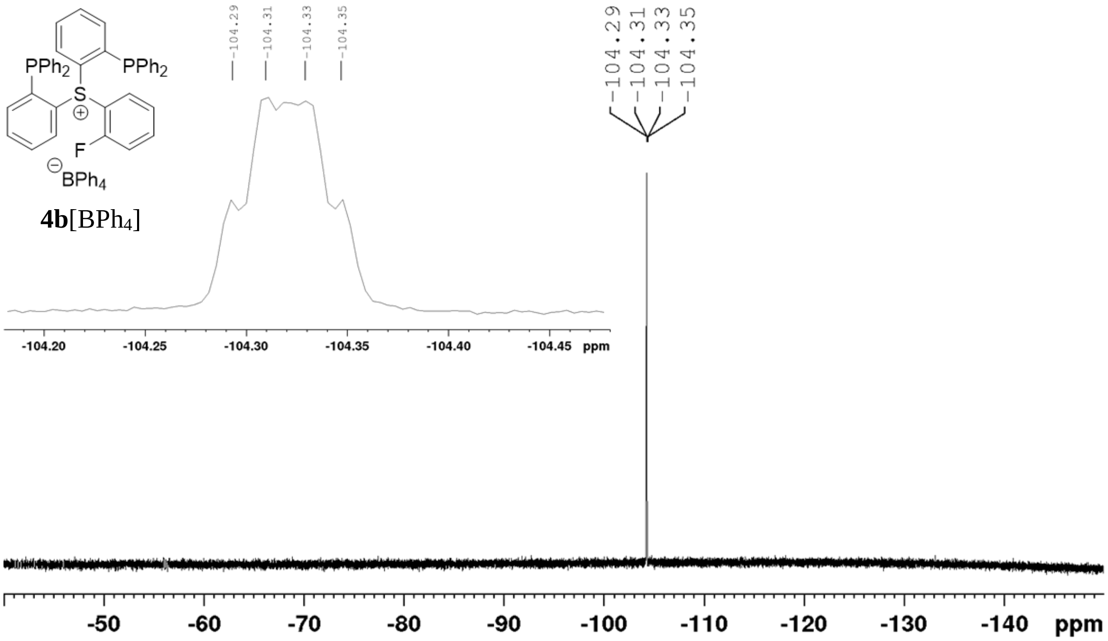


**Figure S46**: $^{19}F$ NMR (376 MHz) of **4b**[BPh4] in $CD_2Cl_2$.

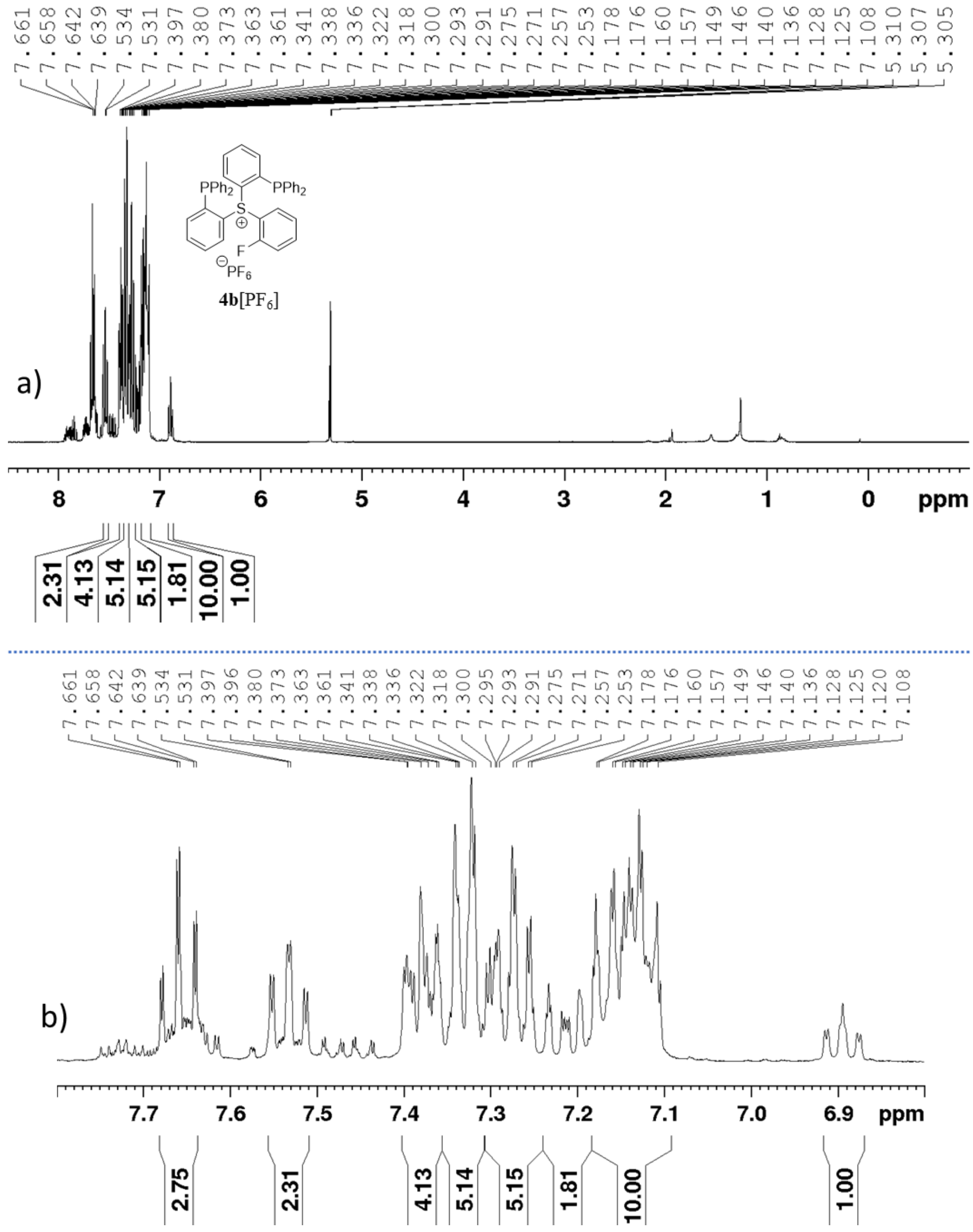


**Figure S47**: $^{1}$H NMR (500 MHz) of **4b**[$PF_6$] in $CD_2Cl_2$. a) full spectrum, b) aromatic region expanded.

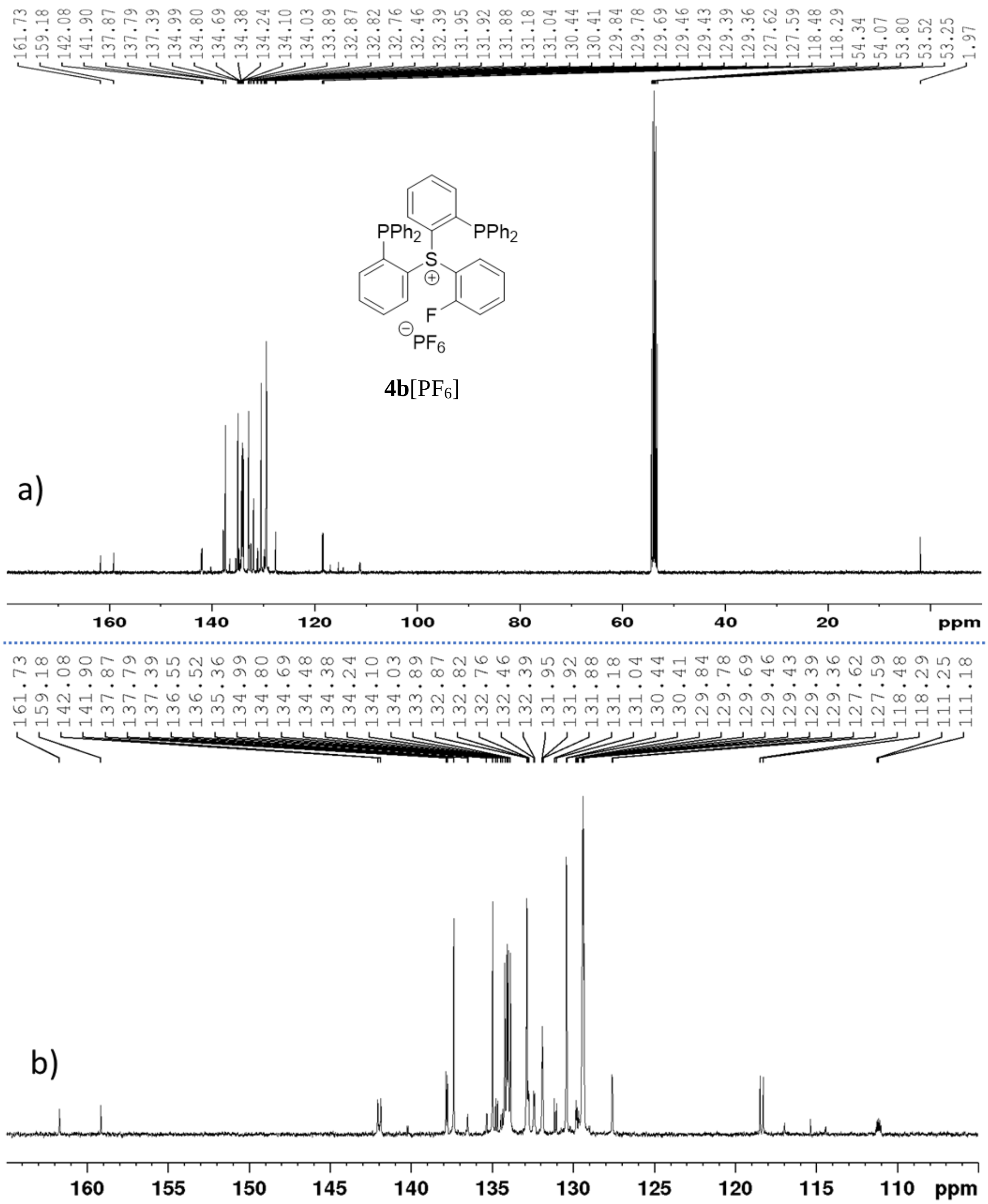

**Figure S48**: $^{13}C$ NMR (101 MHz) of **4b**[$PF_6$] in $CD_2Cl_2$. a) full spectrum, b) aromatic region expanded.

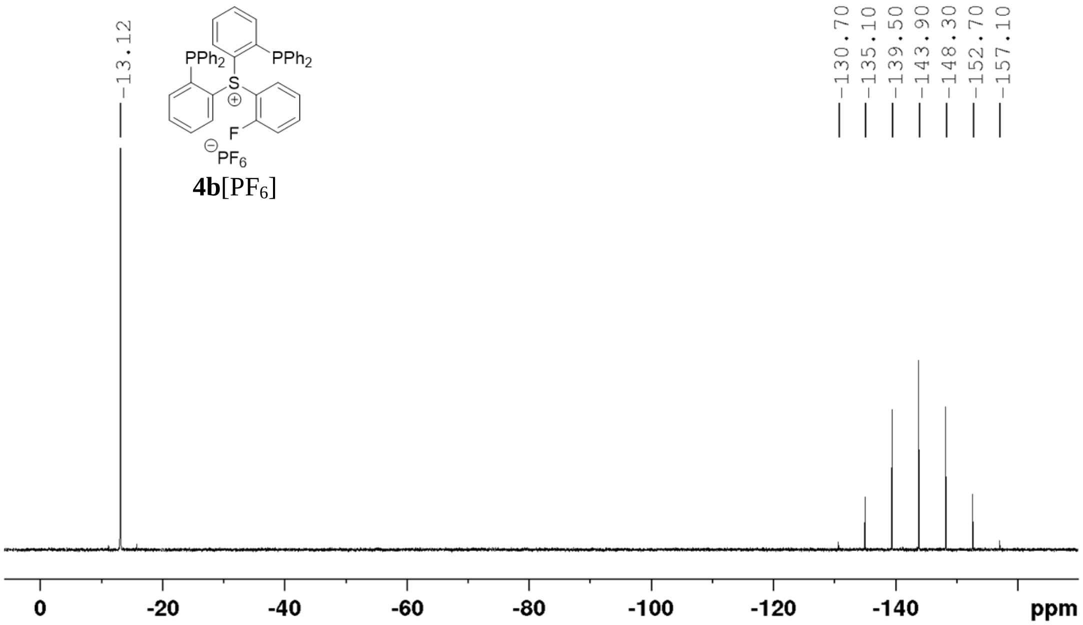


**Figure S49**: $^{31}P\{H\}$ NMR (162 MHz) of **4b**[$PF_6$] in $CDCl_3$.

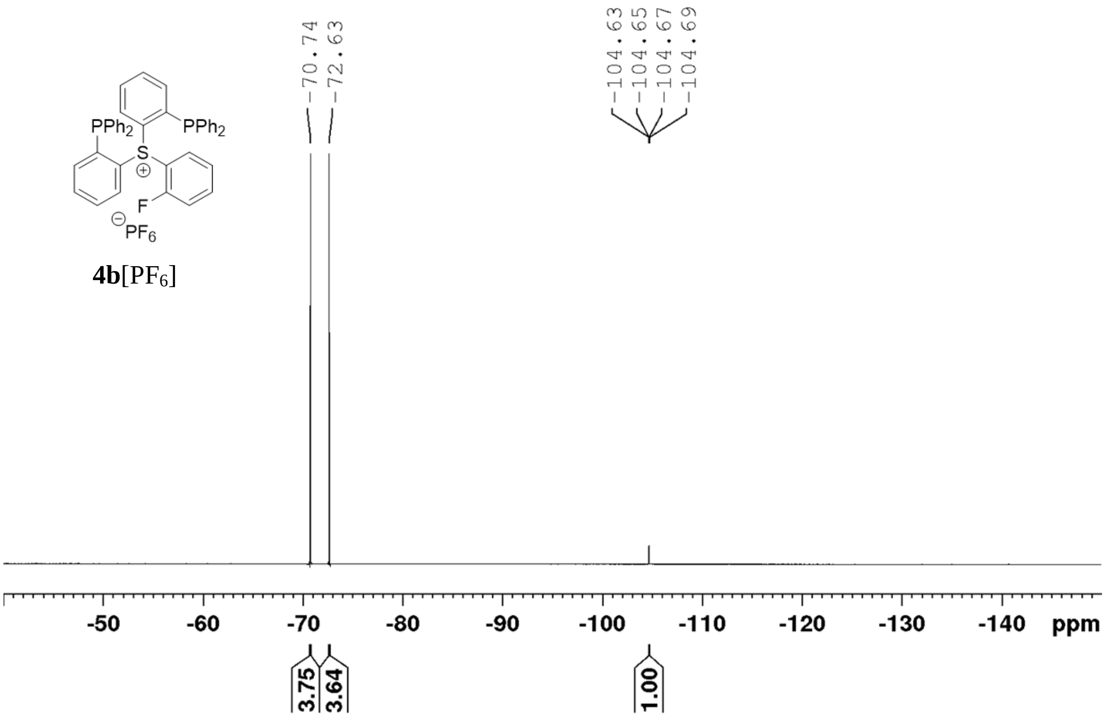


**Figure S50**: $^{19}F$ NMR (376 MHz) of **4b**[$PF_6$] in $CDCl_3$.

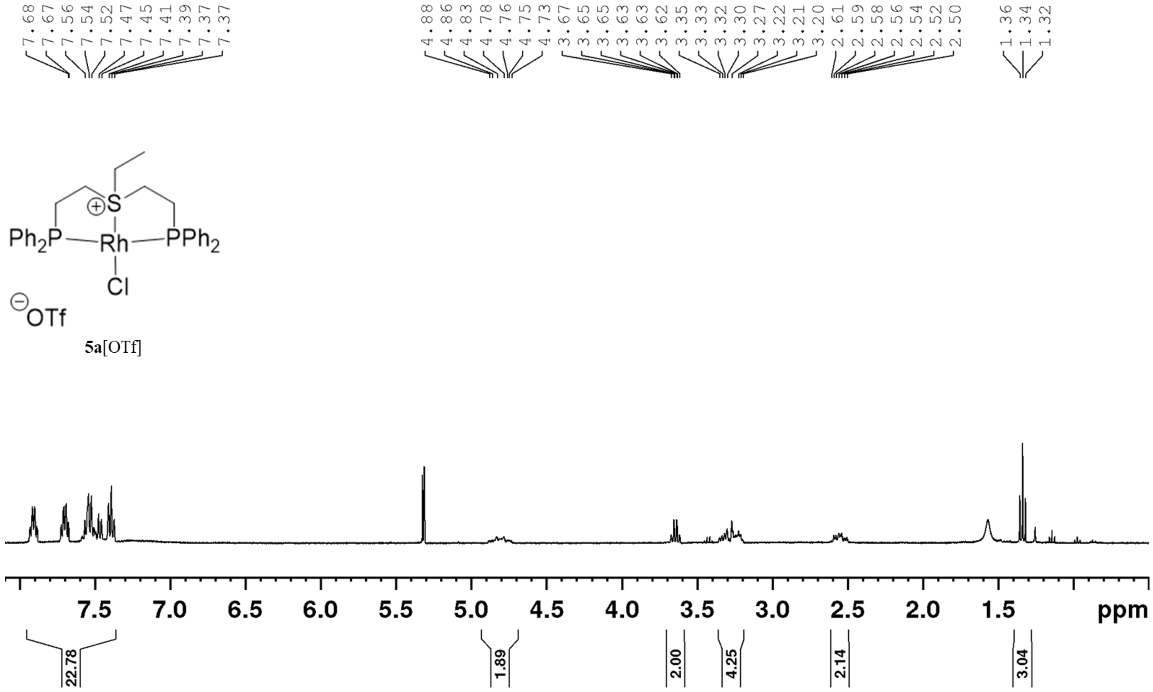


**Figure S51**: $^{1}$H NMR (400 MHz) of **5a**[OTf] in $CD_2Cl_2$.

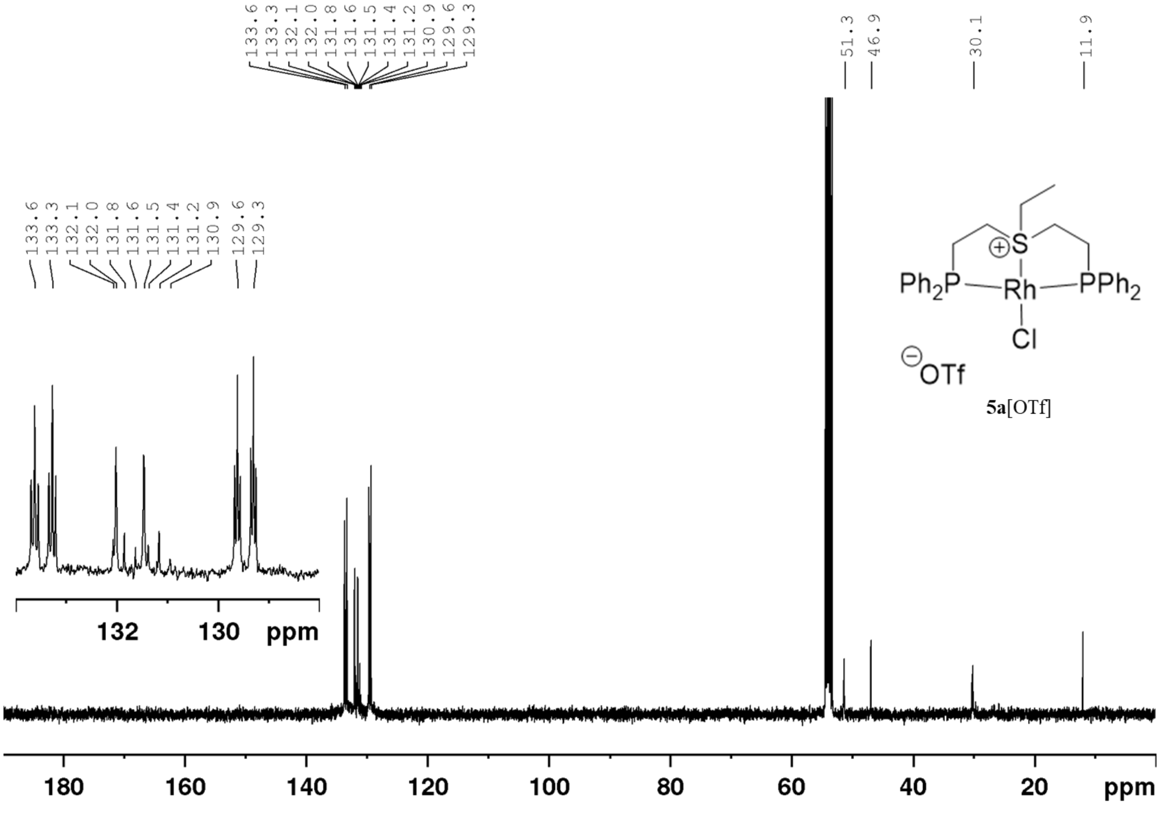


**Figure S52**: $^{13}$C NMR (101 MHz) of **5a**[OTf] in $CD_2Cl_2$.

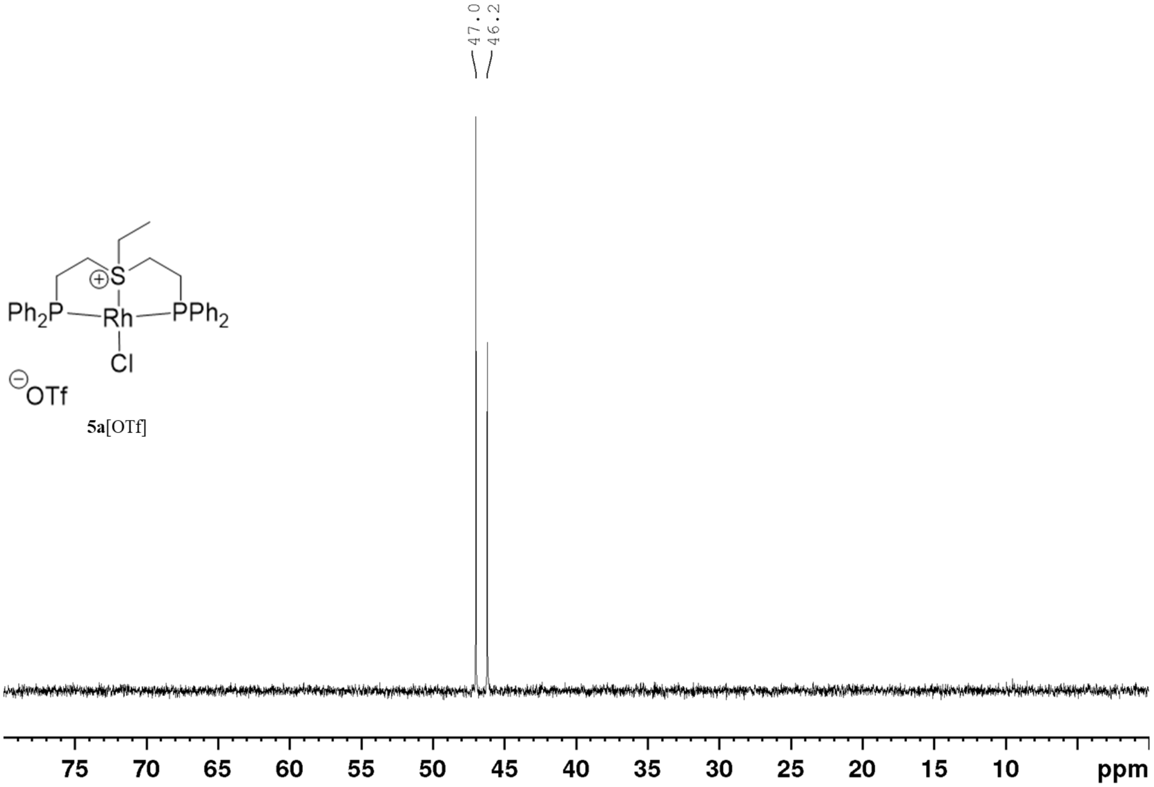


**Figure S53**: $^{31}P\{H\}$ NMR (162 MHz) of **5a**[OTf] in $CD_2Cl_2$.

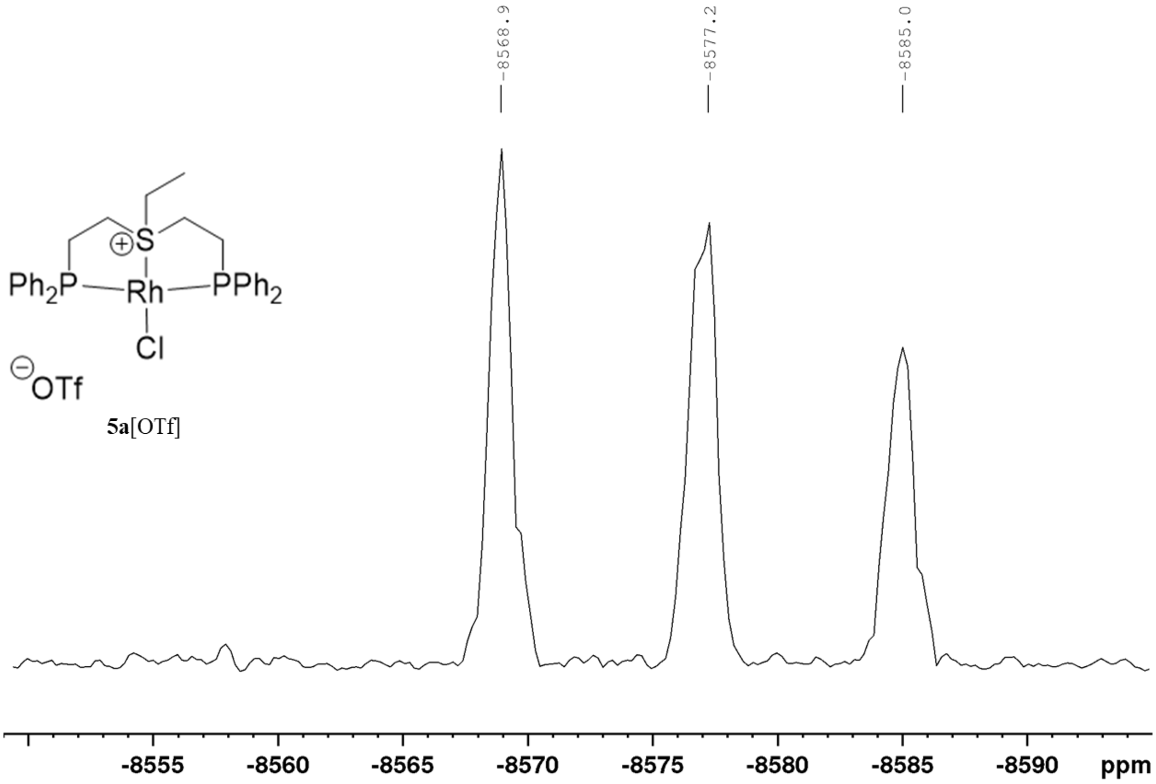


**Figure S54**: $^{103}Rh\{H\}$ NMR (16 MHz) of **5a**[OTf] in $CD_2Cl_2$.

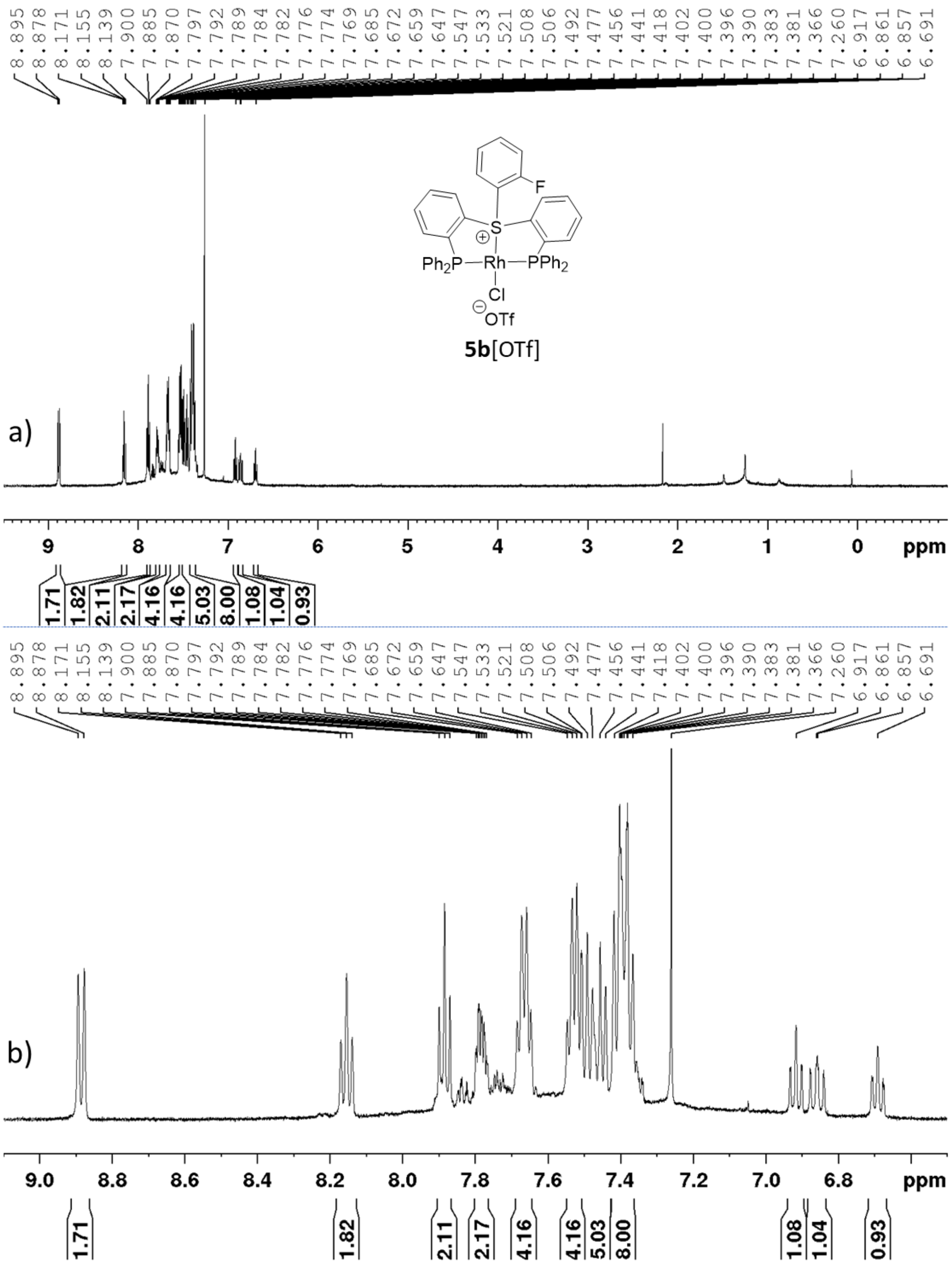


**Figure S55**: $^{1}$H NMR (500 MHz) of **5b**[OTf] in $CDCl_3$. a) full spectrum, b) aromatic region expanded.

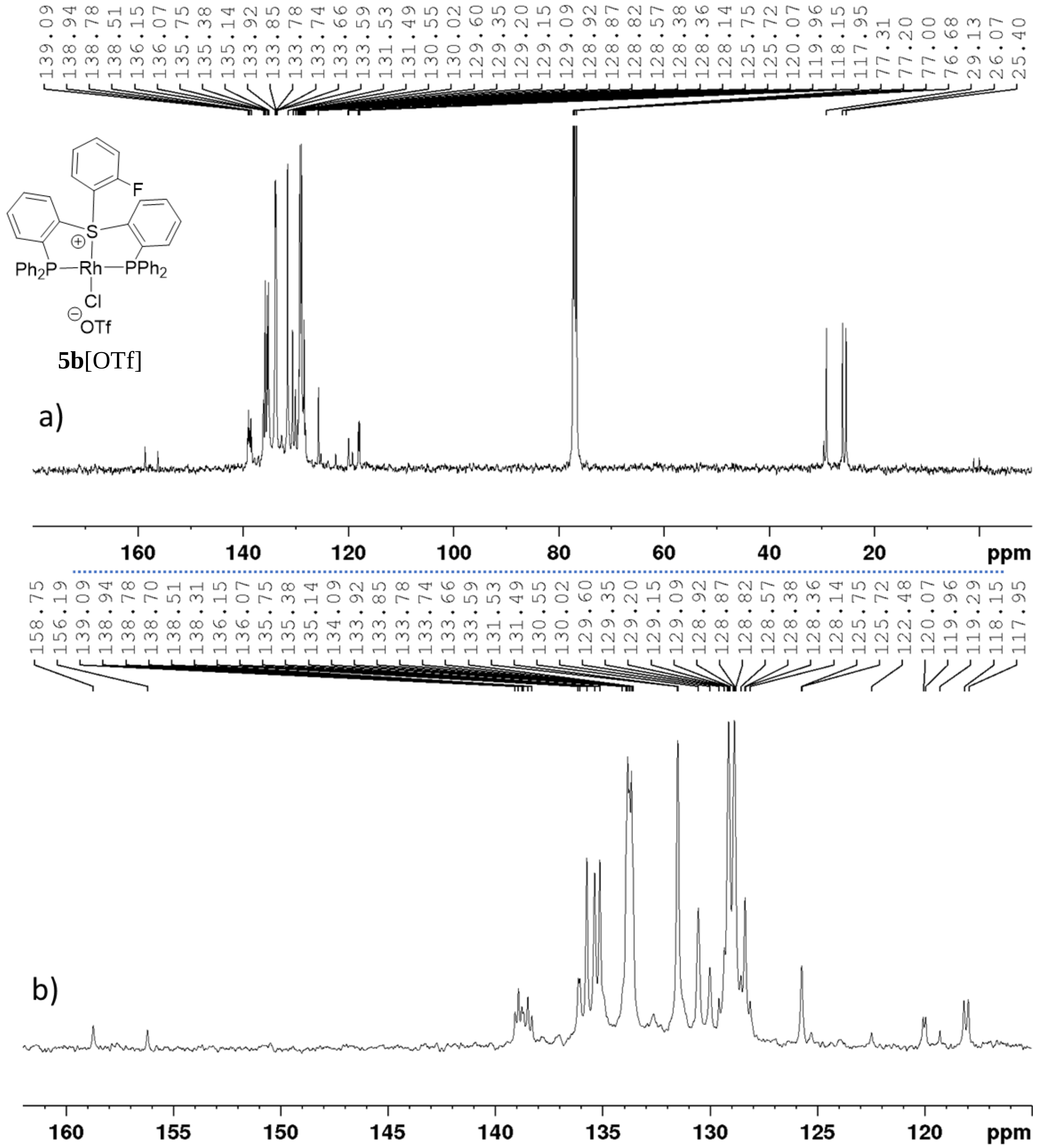


**Figure S56**: $^{13}$C NMR (126 MHz) of **5b**[OTf] in $CDCl_3$. a) full spectrum, b) aromatic region expanded.

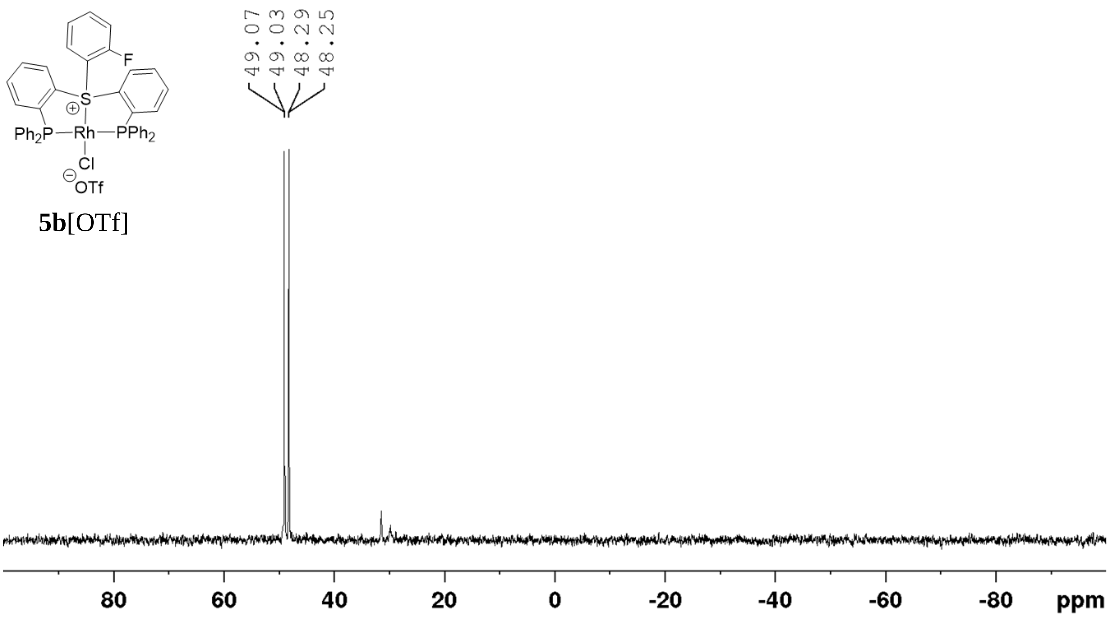


**Figure S57**: $^{31}P\{H\}$ NMR (162 MHz) of **5b**[OTf] in $CDCl_3$.

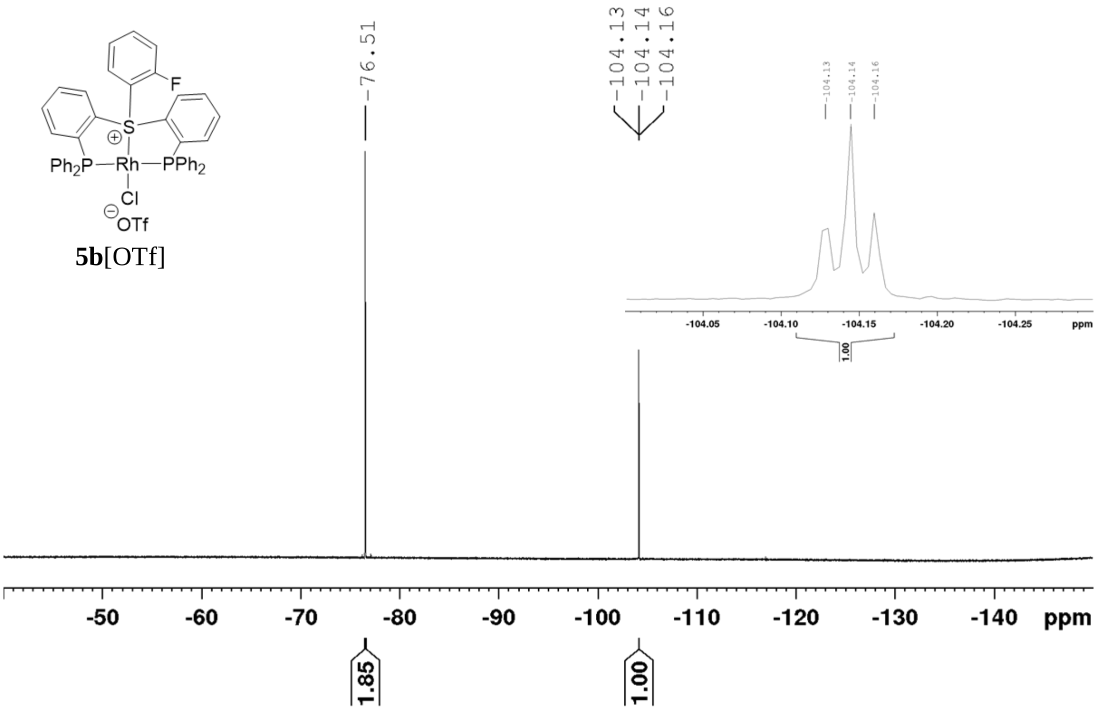


**Figure S58**: $^{19}F\{H\}$ NMR (376 MHz) of **5b**[OTf] in $CDCl_3$.

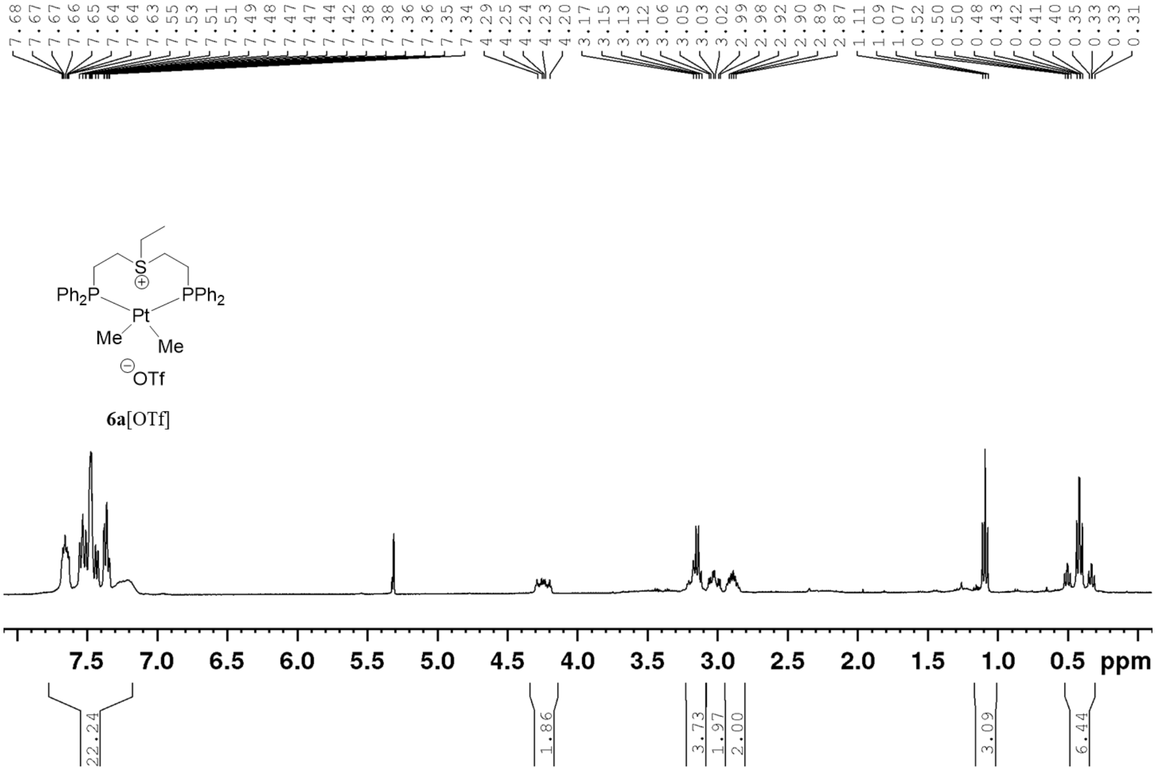


**Figure S59**: $^{1}H$ NMR (400 MHz) of **6a**[OTf] in $CD_2Cl_2$.

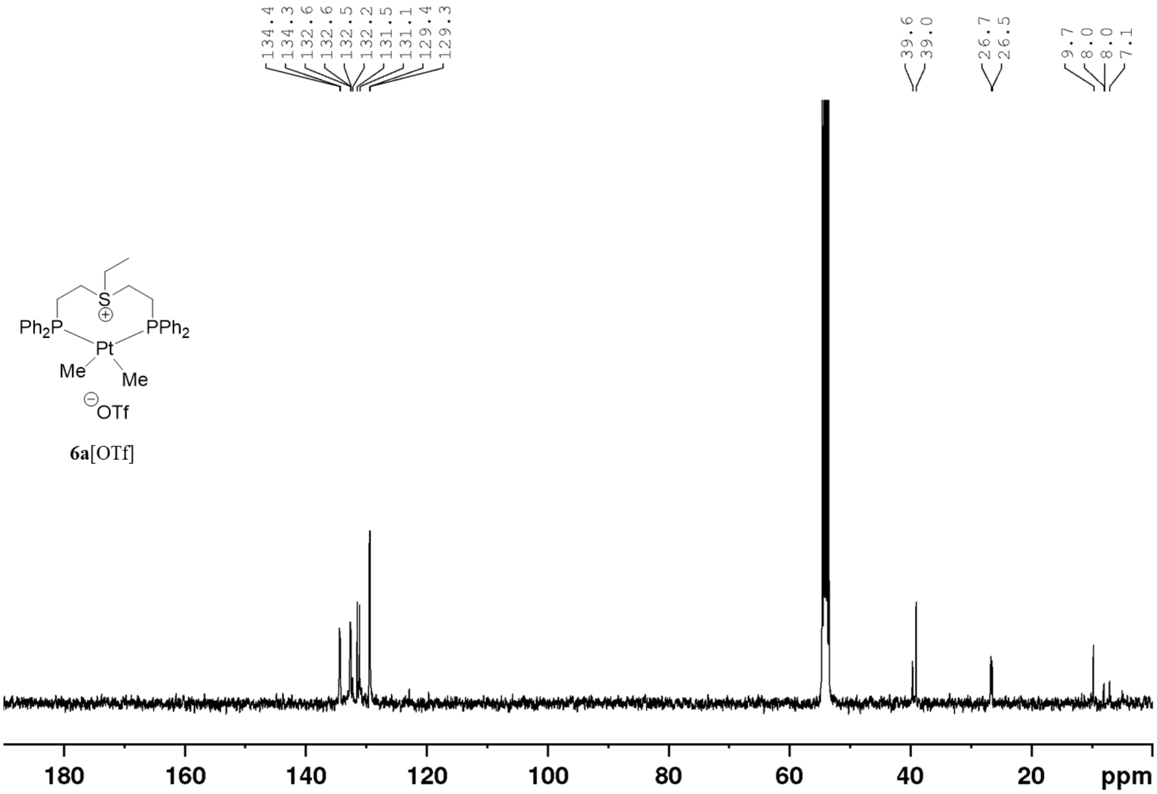


**Figure S60**: $^{13}C$ NMR (101 MHz) of **6a**[OTf] in $CD_2Cl_2$.

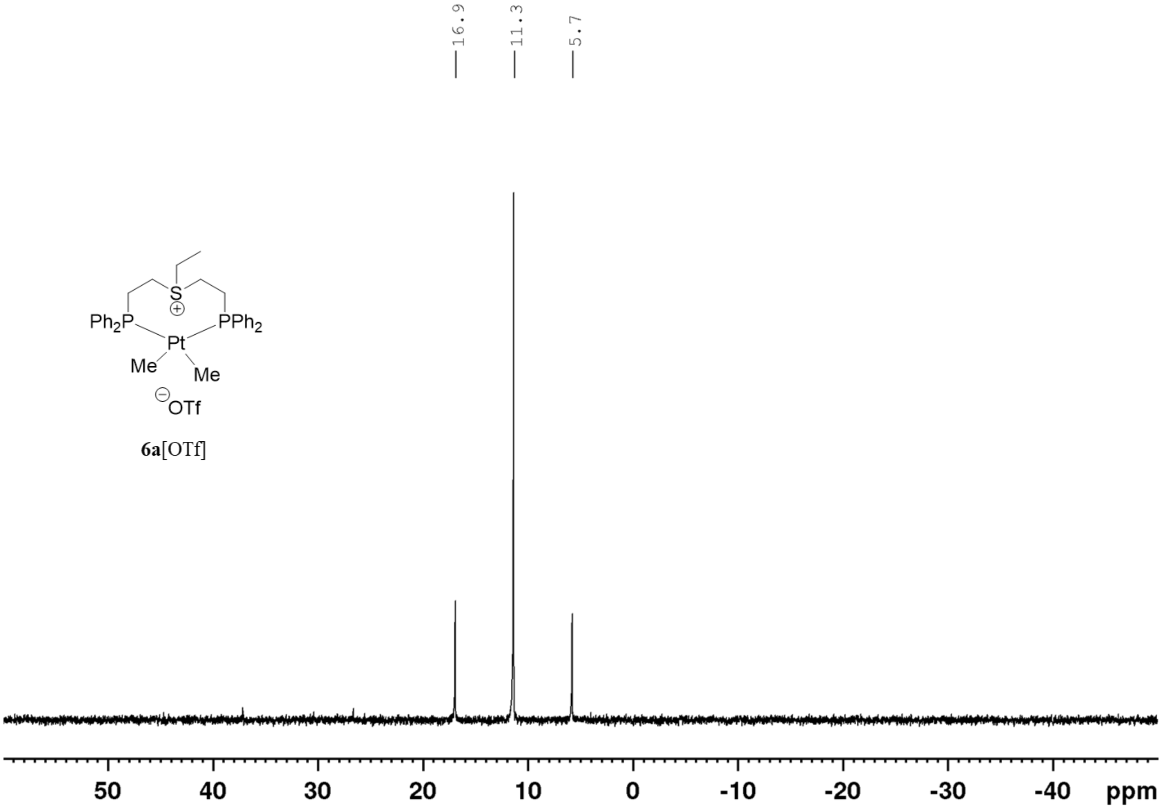


**Figure S61**: $^{31}$P{H} NMR (162 MHz) of **6a**[OTf] in $CD_2Cl_2$.

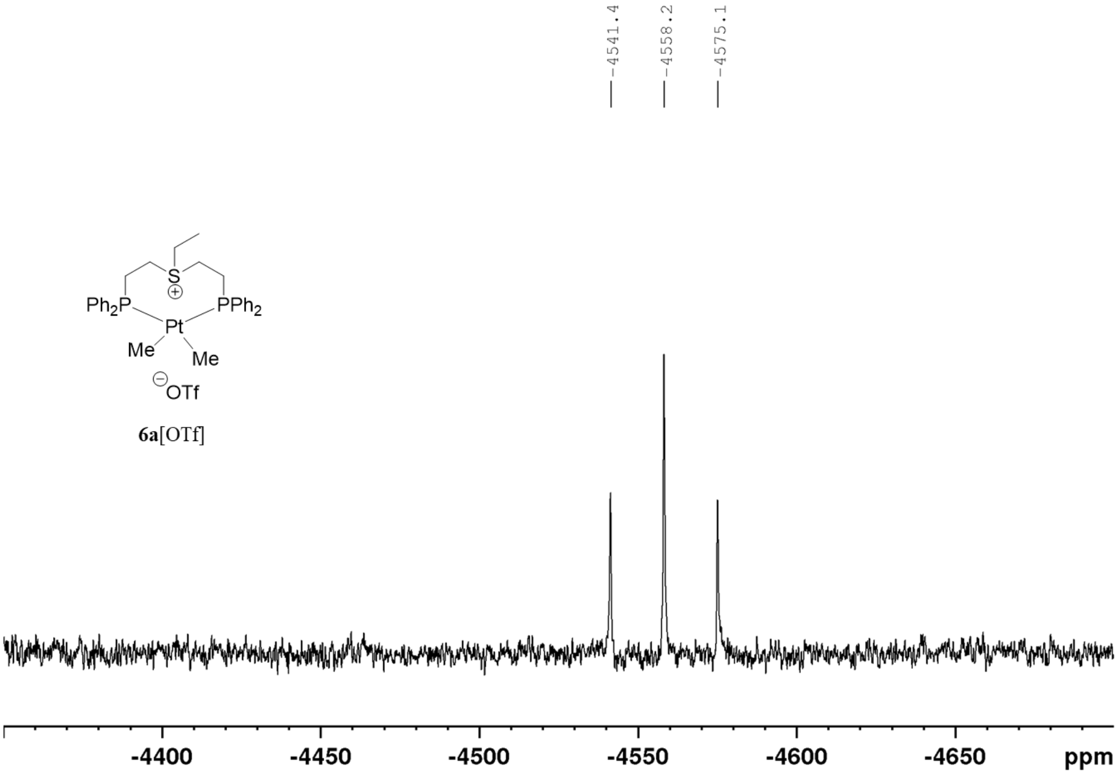


**Figure S62**: $^{195}$Pt{H} NMR (108 MHz) of **6a**[OTf] in $CD_2Cl_2$.

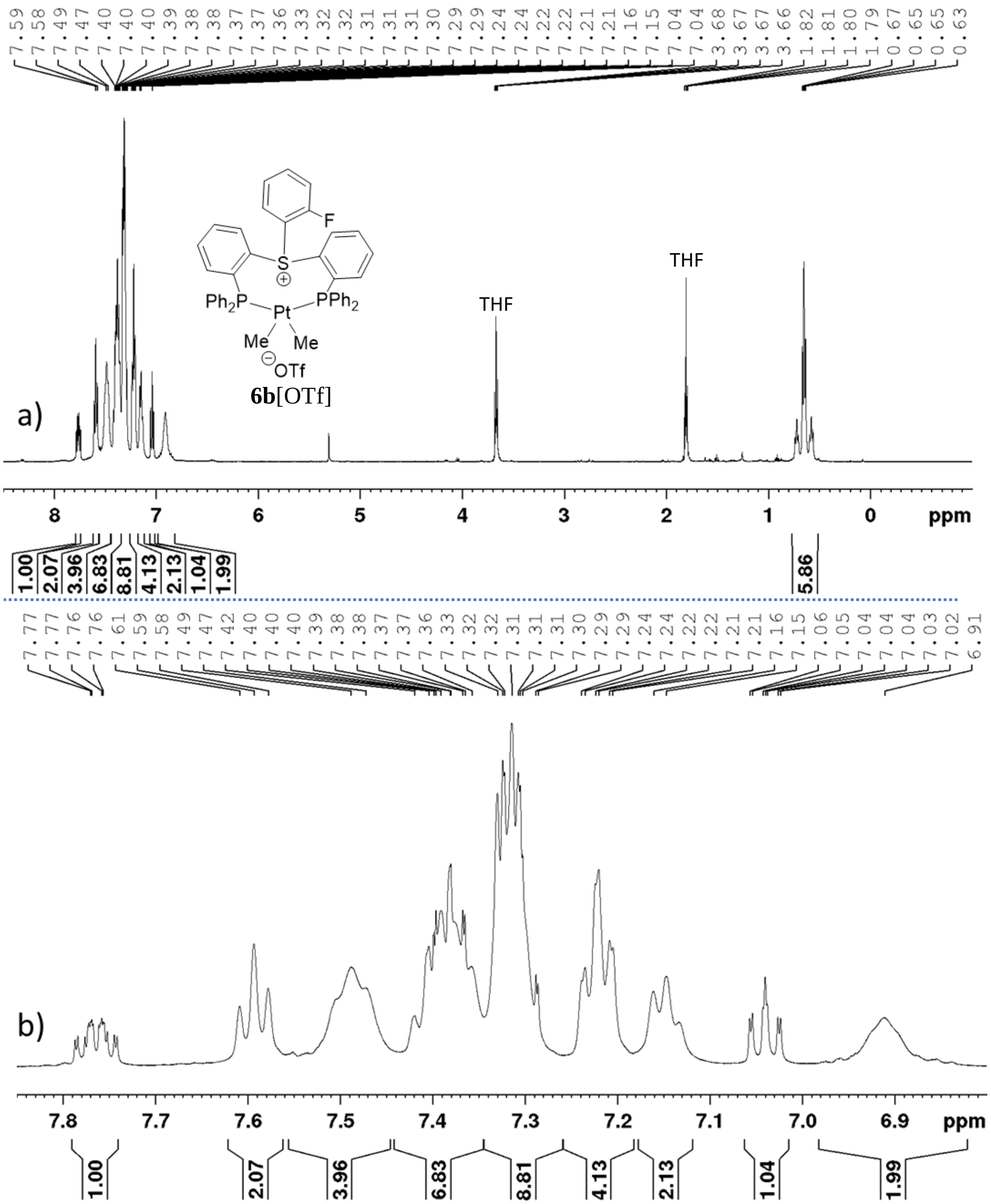


**Figure S63**: $^{1}H$ NMR (500 MHz) of **6b**[OTf] in $CD_2Cl_2$. a) full spectrum, b) aromatic region expanded.

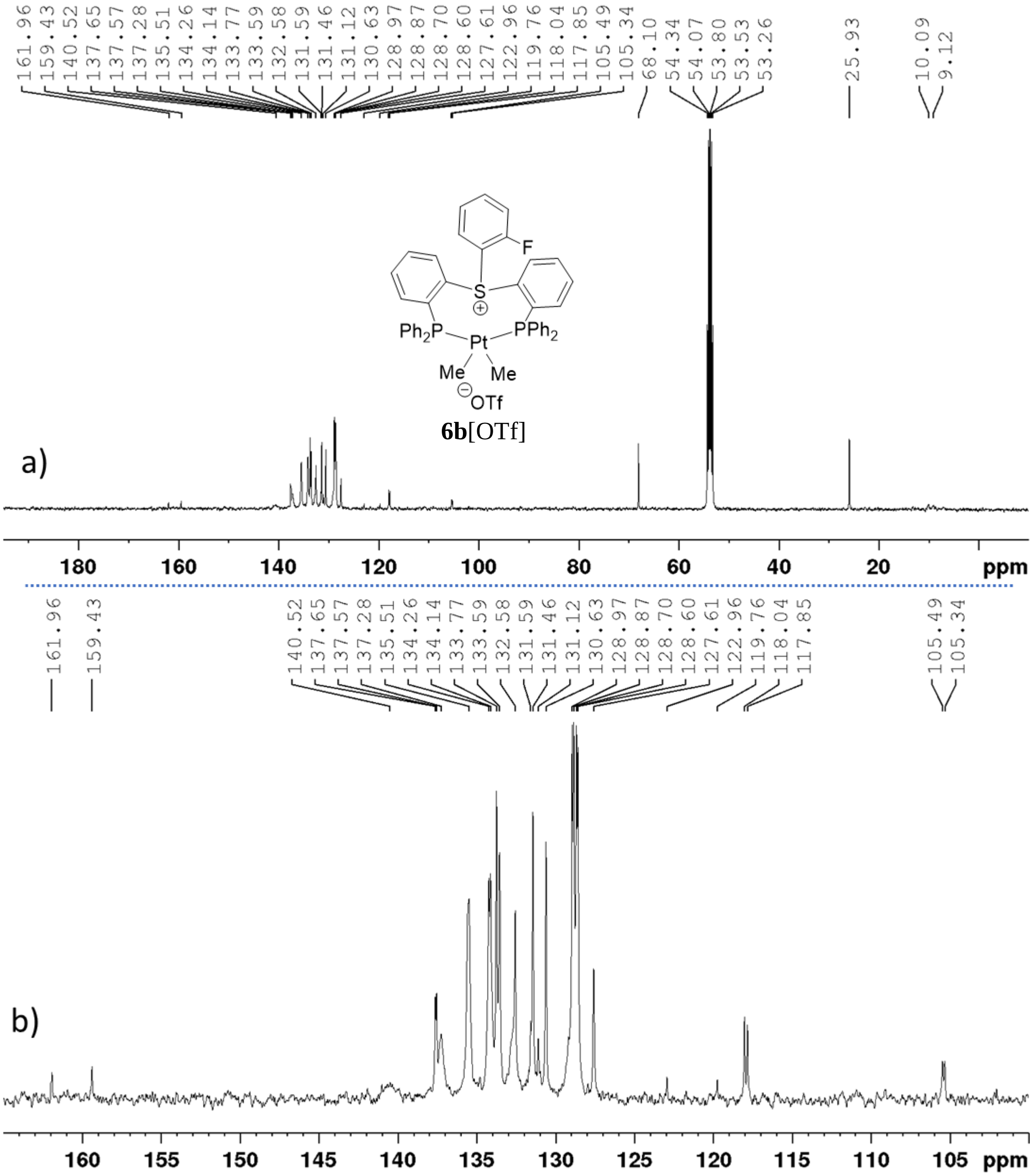


**Figure S64**: $^{13}C$ NMR (101 MHz) of **6b**[OTf] in $CD_2Cl_2$. a) full spectrum, b) aromatic region expanded.

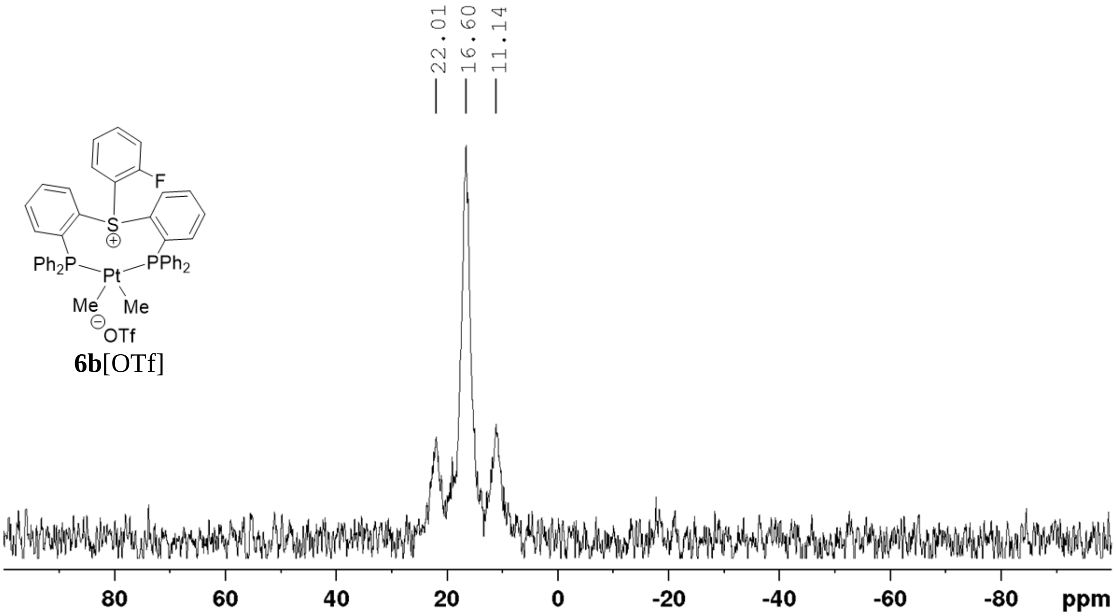


**Figure S65**: $^{31}P\{H\}$ NMR (162 MHz) of **6b**[OTf] in $CD_2Cl_2$.

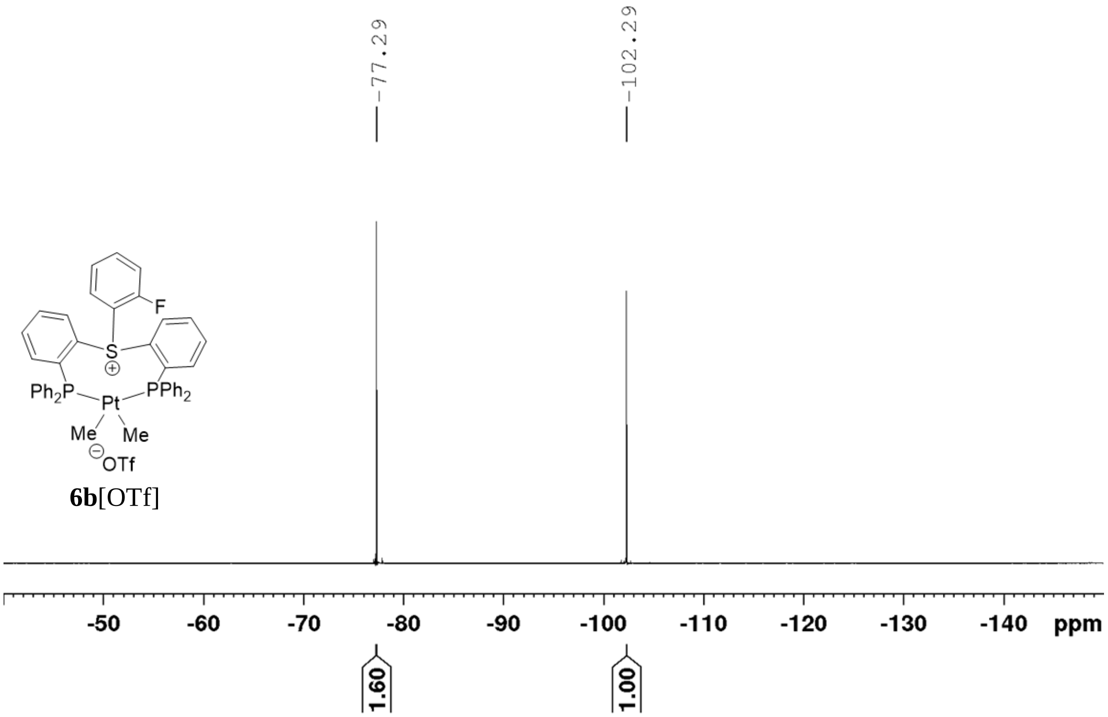


**Figure S66**: $^{19}F\{H\}$ NMR (376 MHz) of **6b**[OTf] in $CD_2Cl_2$.

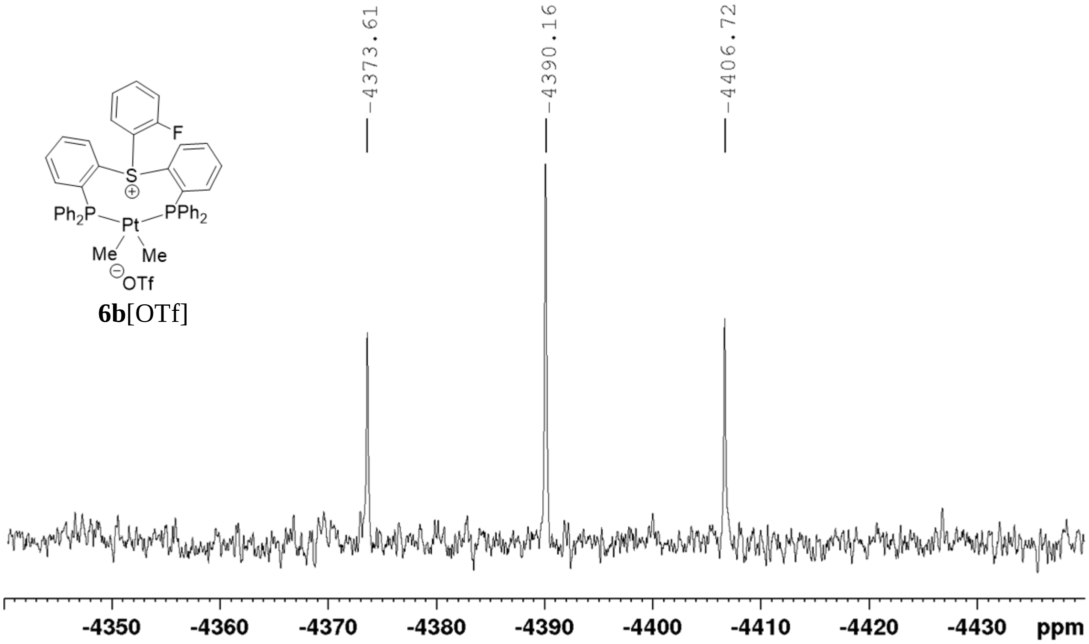


**Figure S67**: $^{195}$Pt{H} NMR (108 MHz) of **6b**[OTf] in $CD_2Cl_2$.

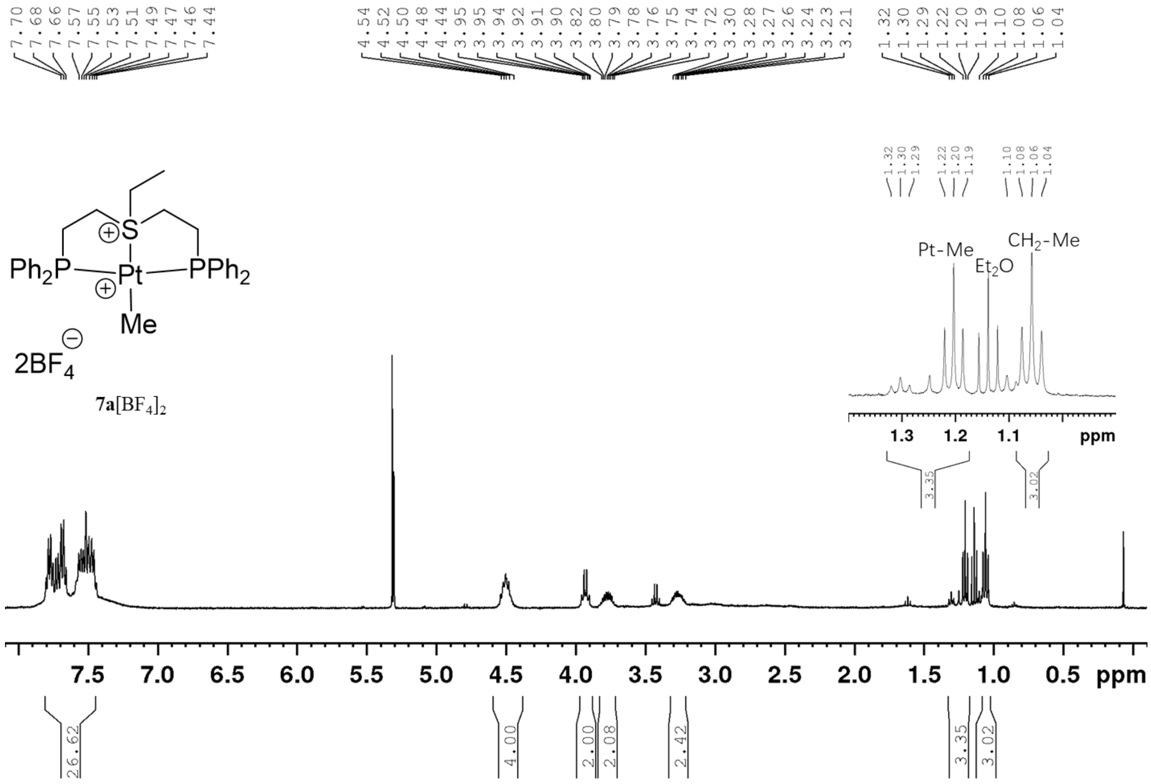


**Figure S68**: $^{1}$H NMR (400 MHz) of **7a**$[BF_4]_2$ in $CD_2Cl_2$.

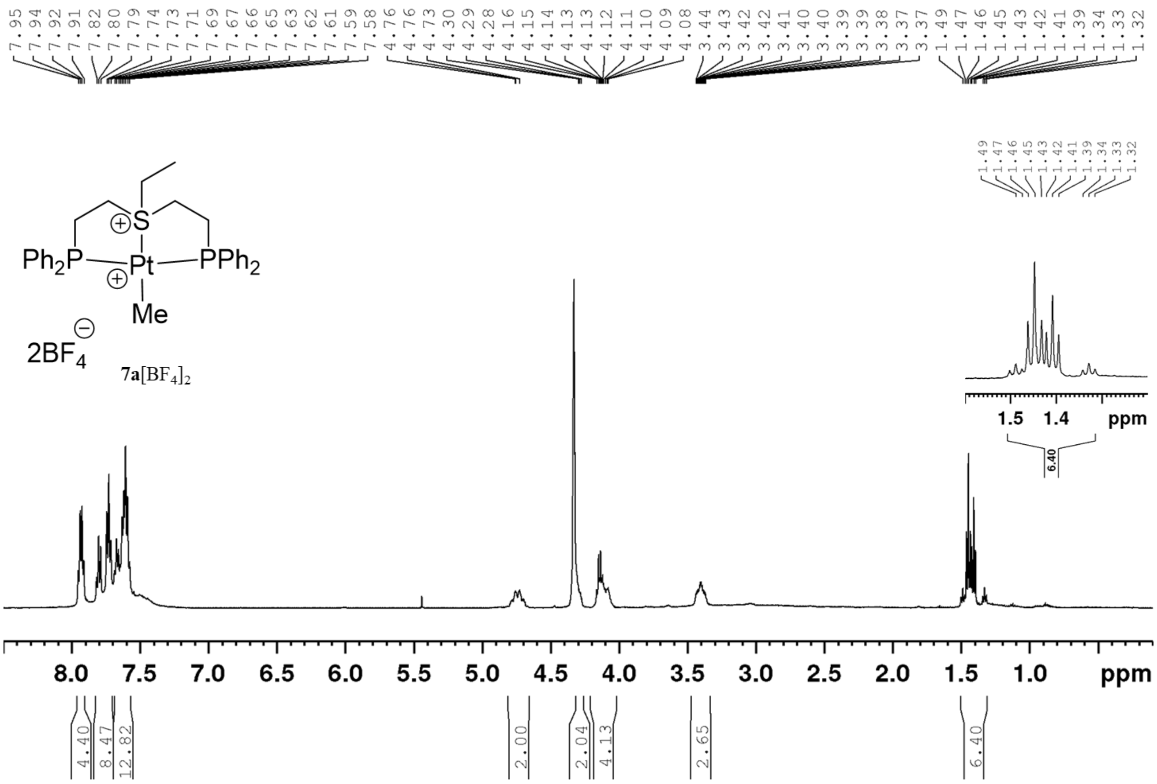


**Figure S69**: $^{1}$H NMR (500 MHz) of **7a**[BF$_4$]$_2$ in $CD_3NO_2$.

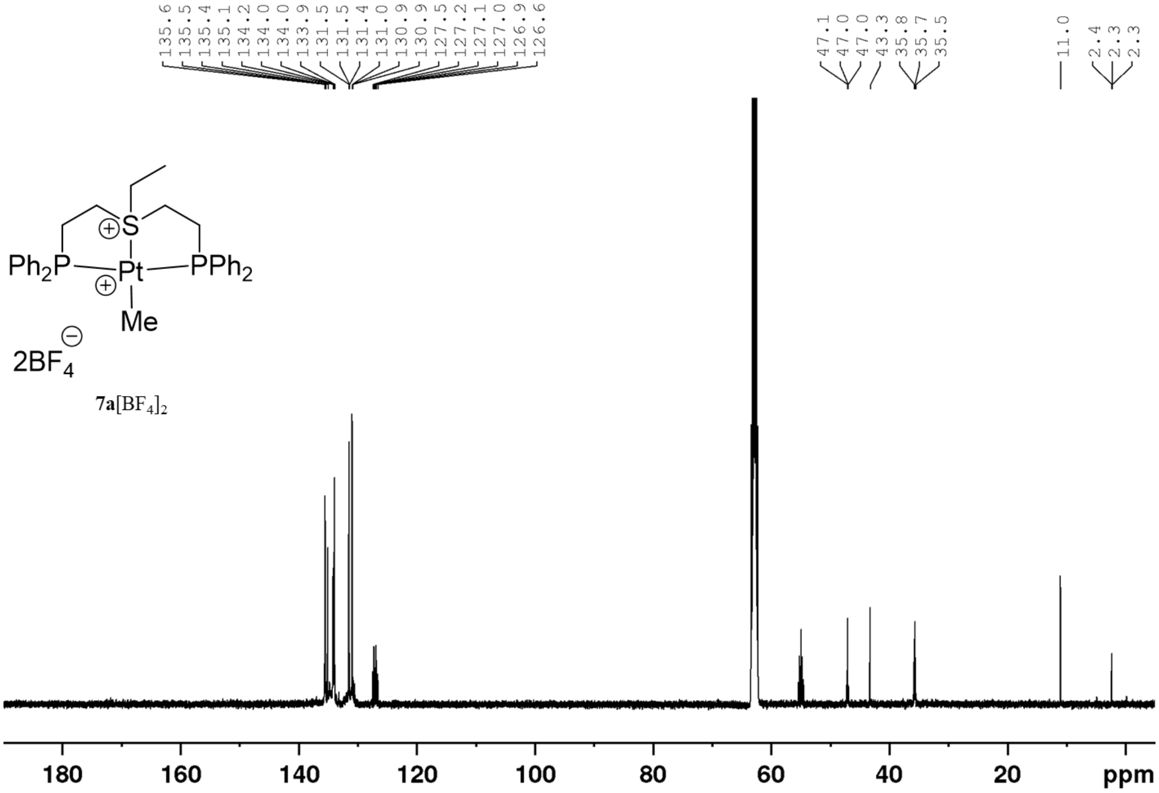


**Figure S70**: $^{13}$C NMR (126 MHz) of **7a**[BF$_4$]$_2$ in $CD_3NO_2$.

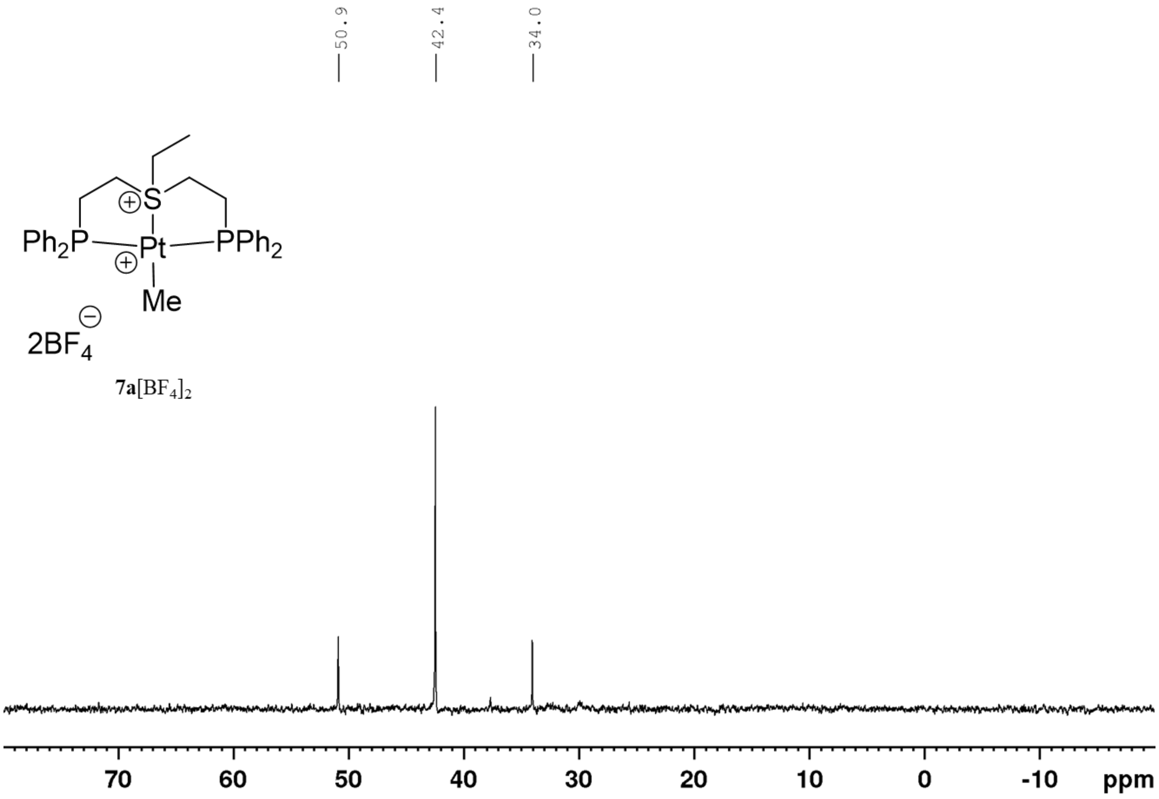


**Figure S71**: $^{31}$P{H} NMR (162 MHz) of **7a**[BF$_4$]$_2$ in $CD_2Cl_2$.

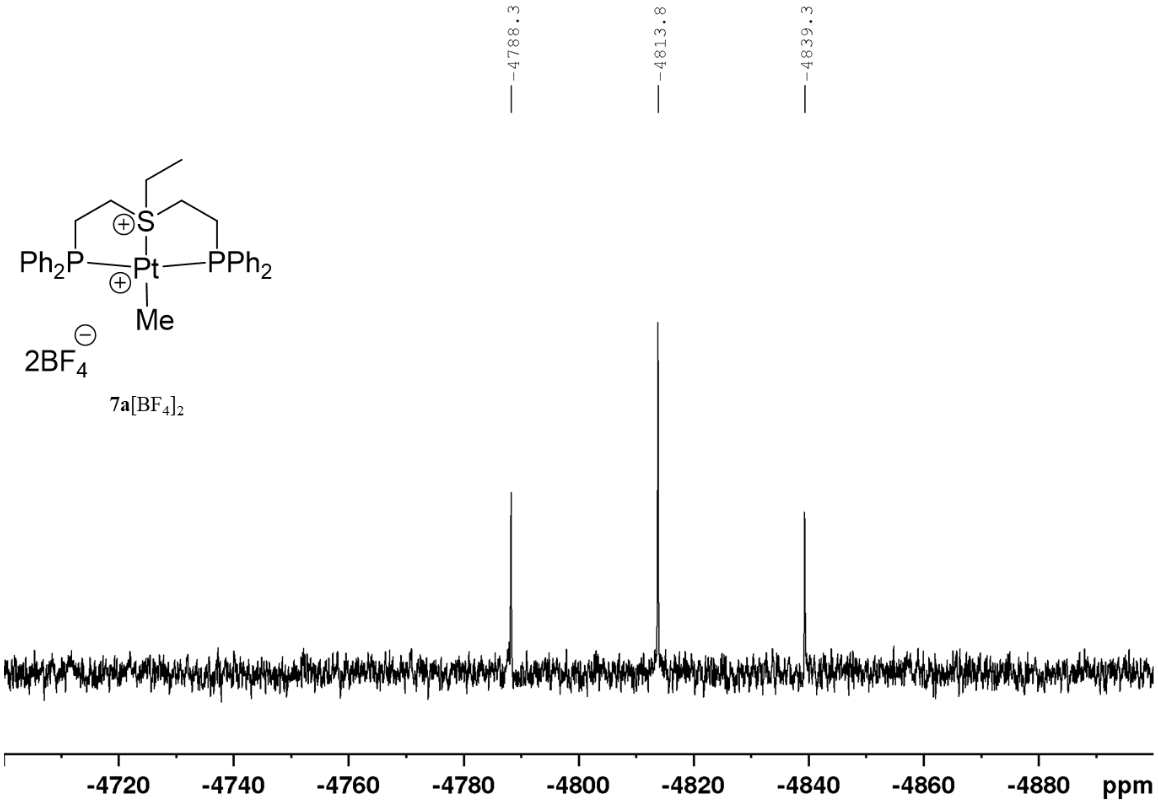


**Figure S72**: $^{195}$Pt{H} NMR (108 MHz) of **7a**[BF$_4$]$_2$ in $CD_3NO_2$.

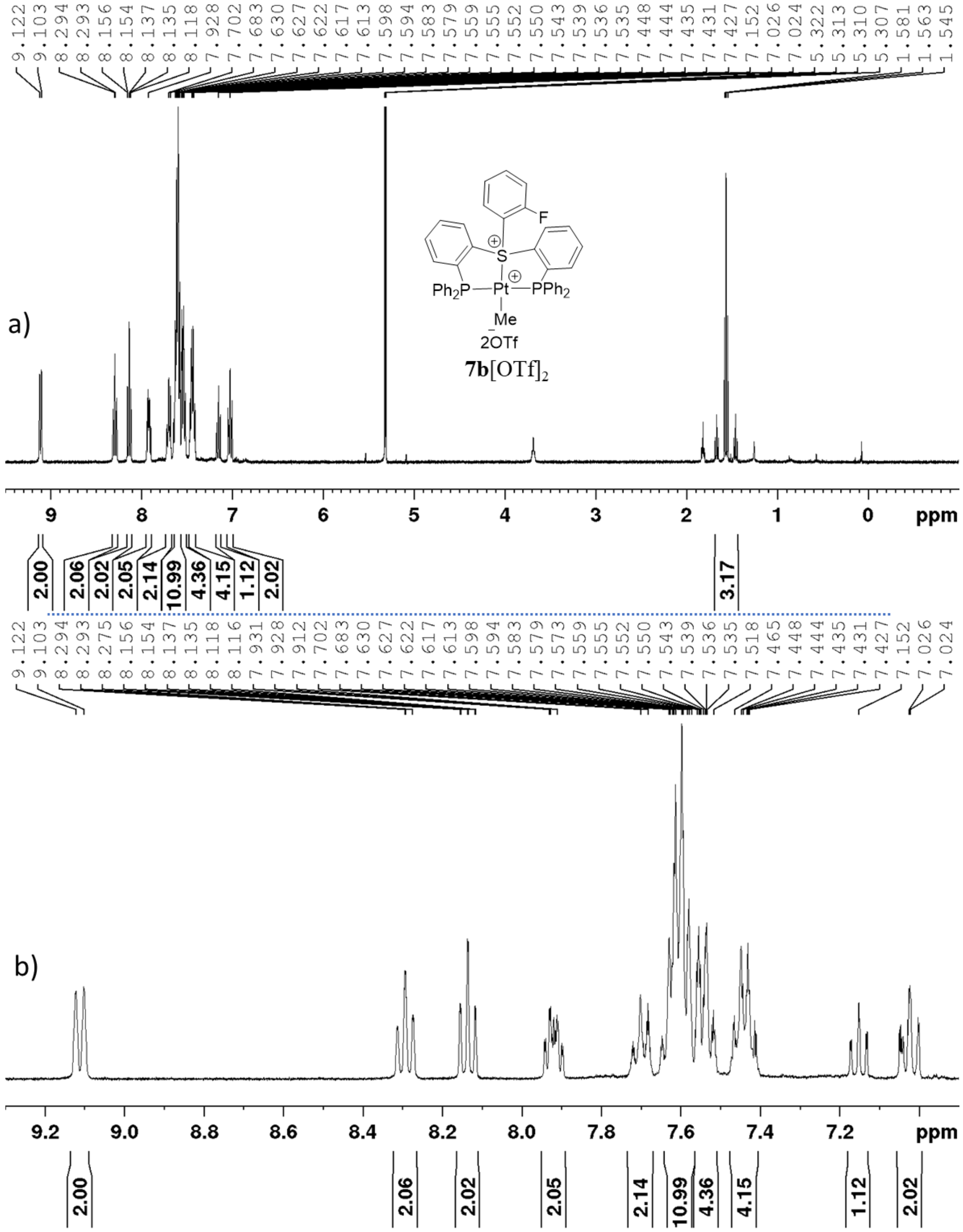

**Figure S73**: $^{1}H$ NMR (500 MHz) of **7b**[OTf]$_2$ in $CD_2Cl_2$. a) full spectrum, b) aromatic region expanded.

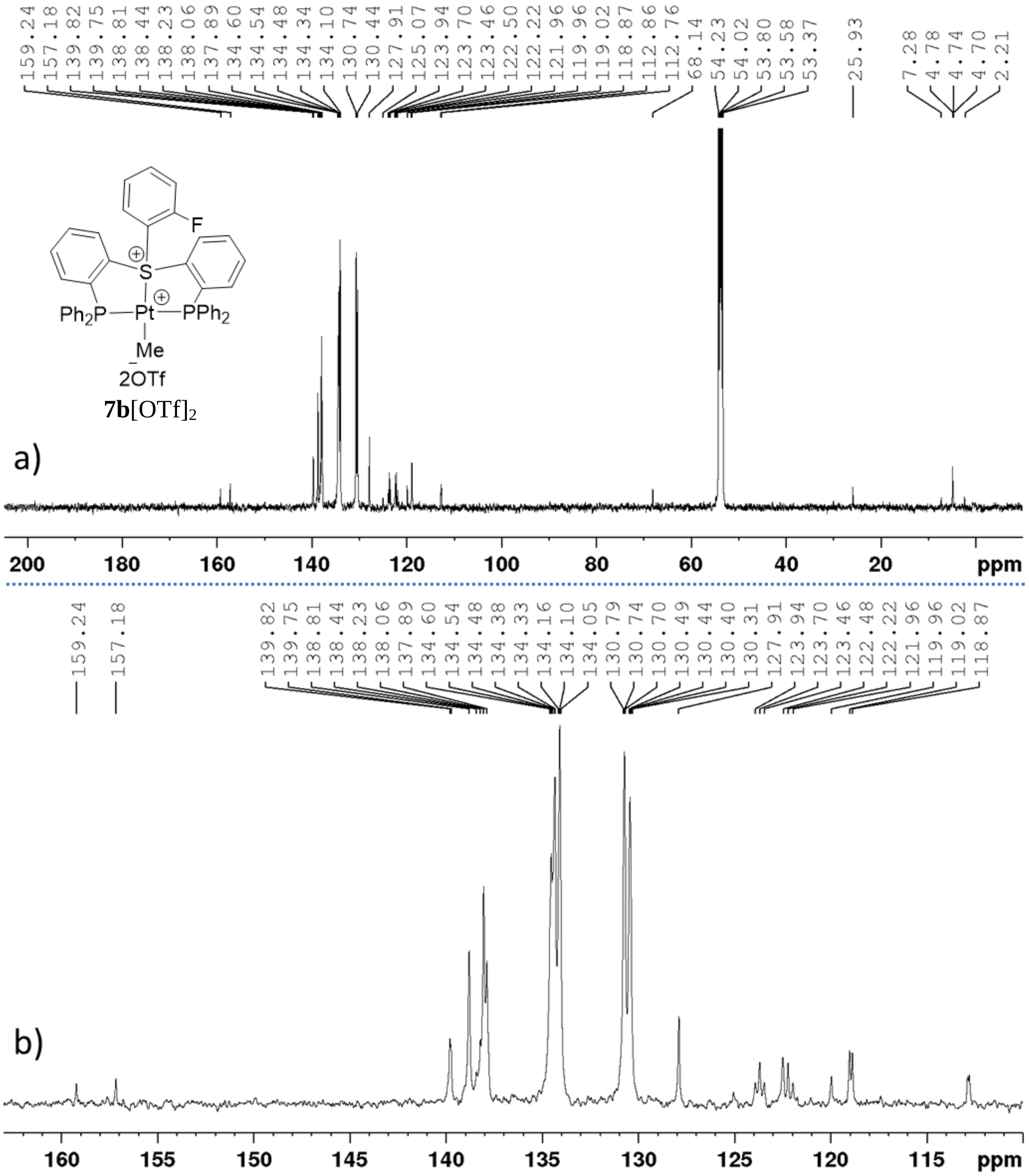

**Figure S74**: $^{31}$C NMR (126 MHz) of **7b**[OTf]$_2$ in $CD_2Cl_2$. a) full spectrum, b) aromatic region expanded.

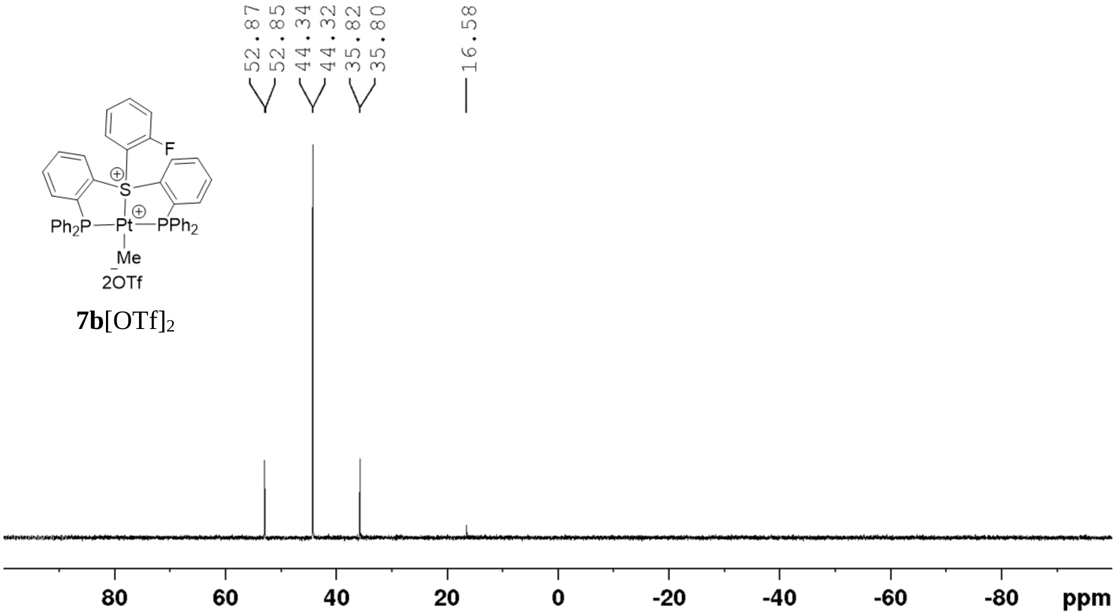


**7b**[OTf]$_2$

**Figure S75**: $^{31}$P{H} NMR (162 MHz) of **7b**[OTf]$_2$ in $CD_2Cl_2$.

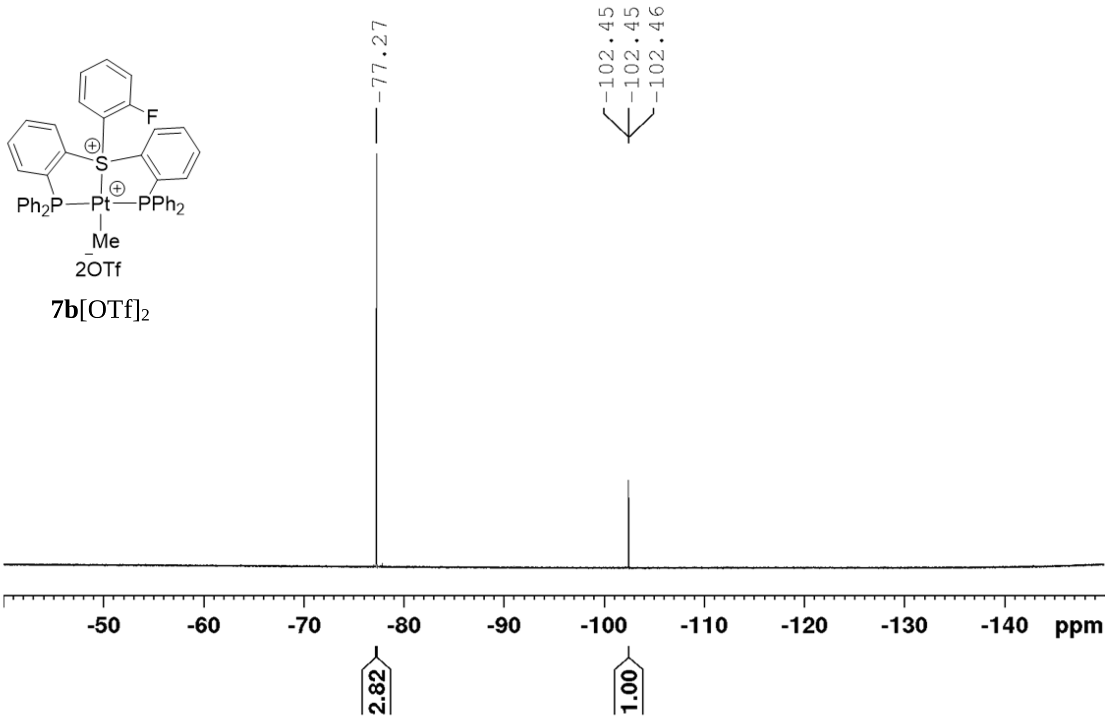


**7b**[OTf]$_2$

**Figure S76**: $^{19}$F{H} NMR (376 MHz) of **7b**[OTf]$_2$ in $CD_2Cl_2$.

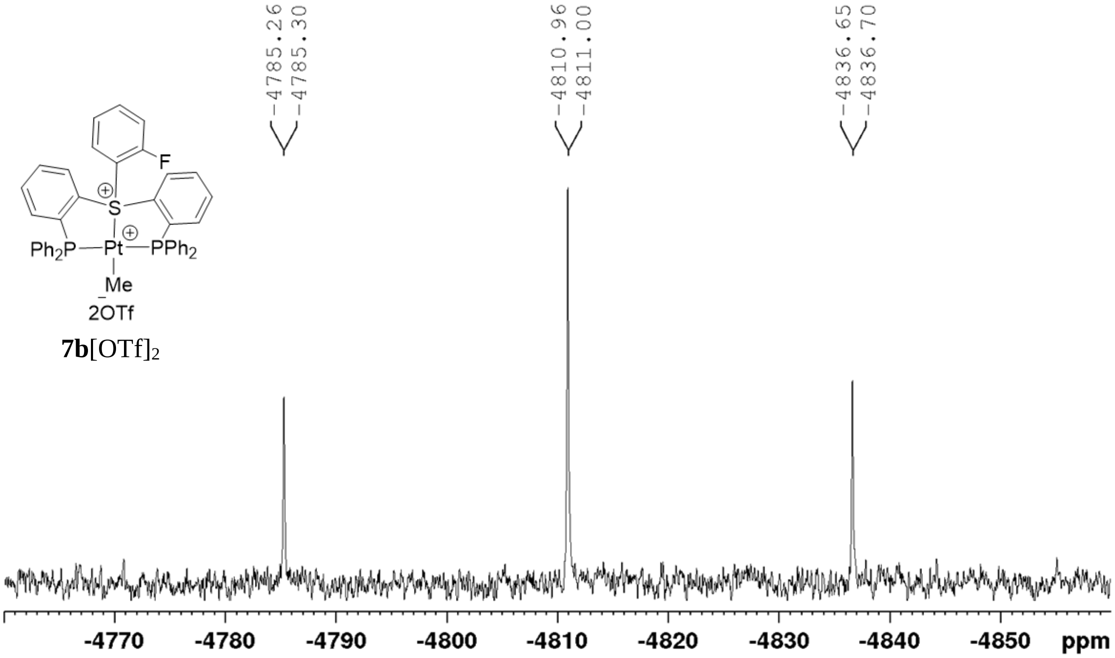


**Figure S77**: $^{195}$Pt{H} NMR (108 MHz) of **7b**[OTf]$_2$ in $CD_2Cl_2$.

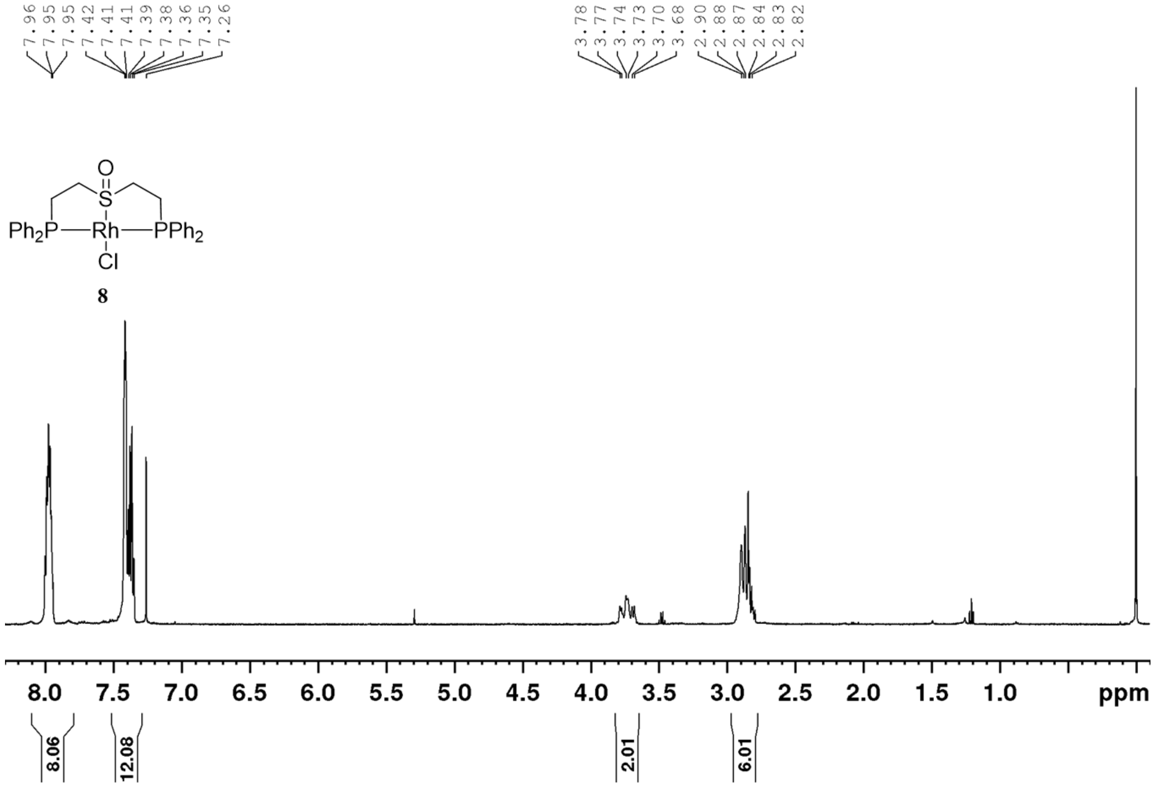


**Figure S78**: $^{1}$H NMR (500 MHz) of **8** in $CDCl_3$.

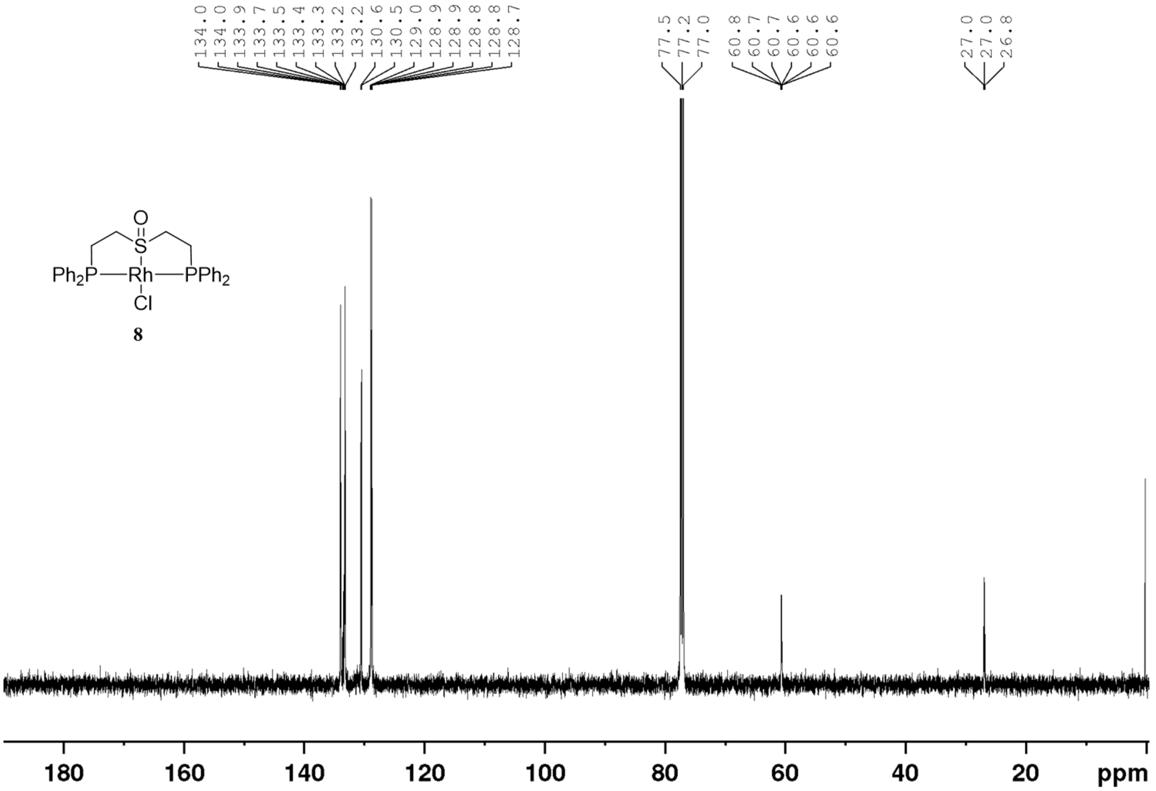


**Figure S79**: $^{13}$C NMR (126 MHz) of **8** in $CDCl_3$.

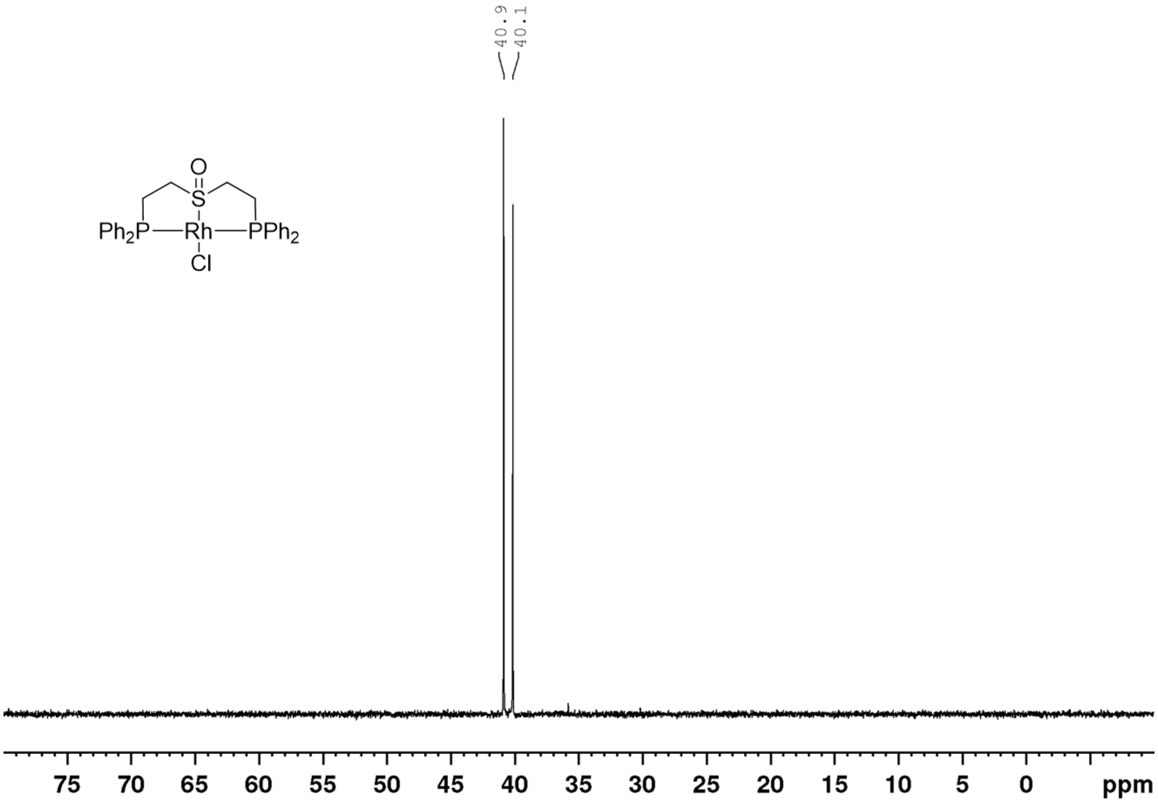


**Figure S80**: $^{31}$P{H} NMR (202 MHz) of **8** in $CDCl_3$.

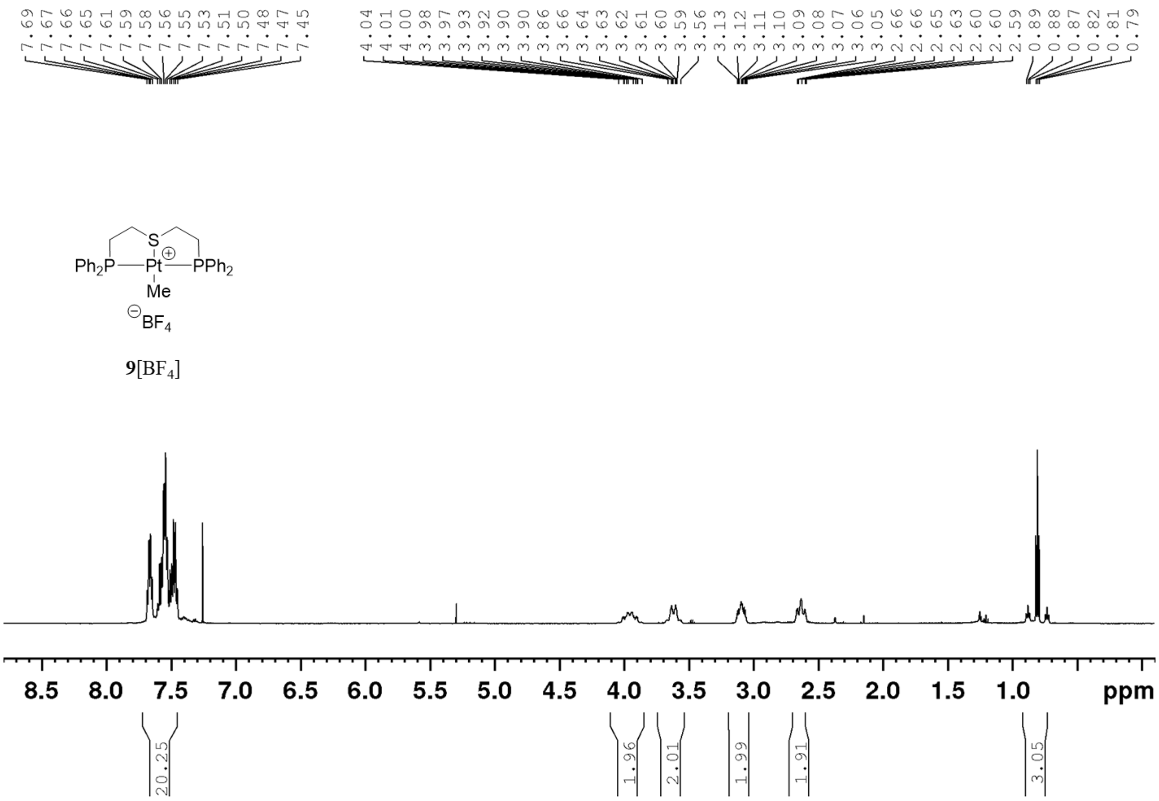

**Figure S81**: $^{1}$H NMR (400 MHz) of **9**[$BF_4$] in $CDCl_3$.

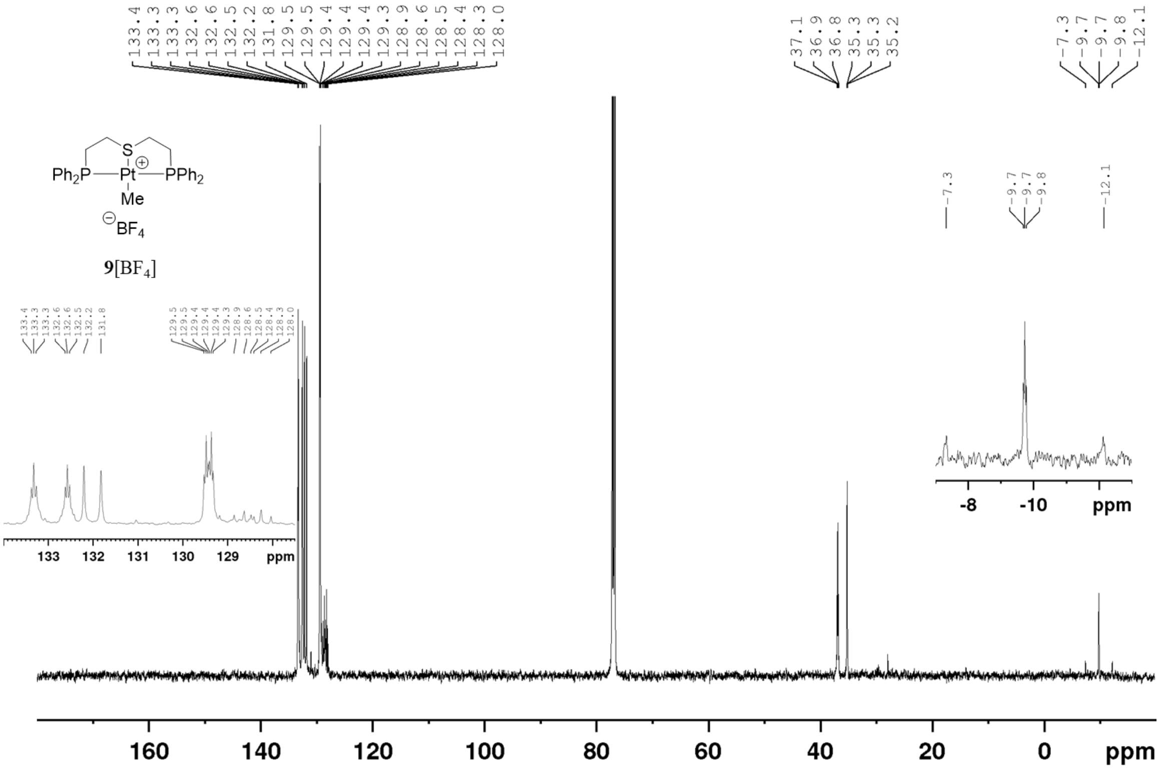

**Figure S82**: $^{13}$C NMR (101 MHz) of **9**[$BF_4$] in $CDCl_3$.

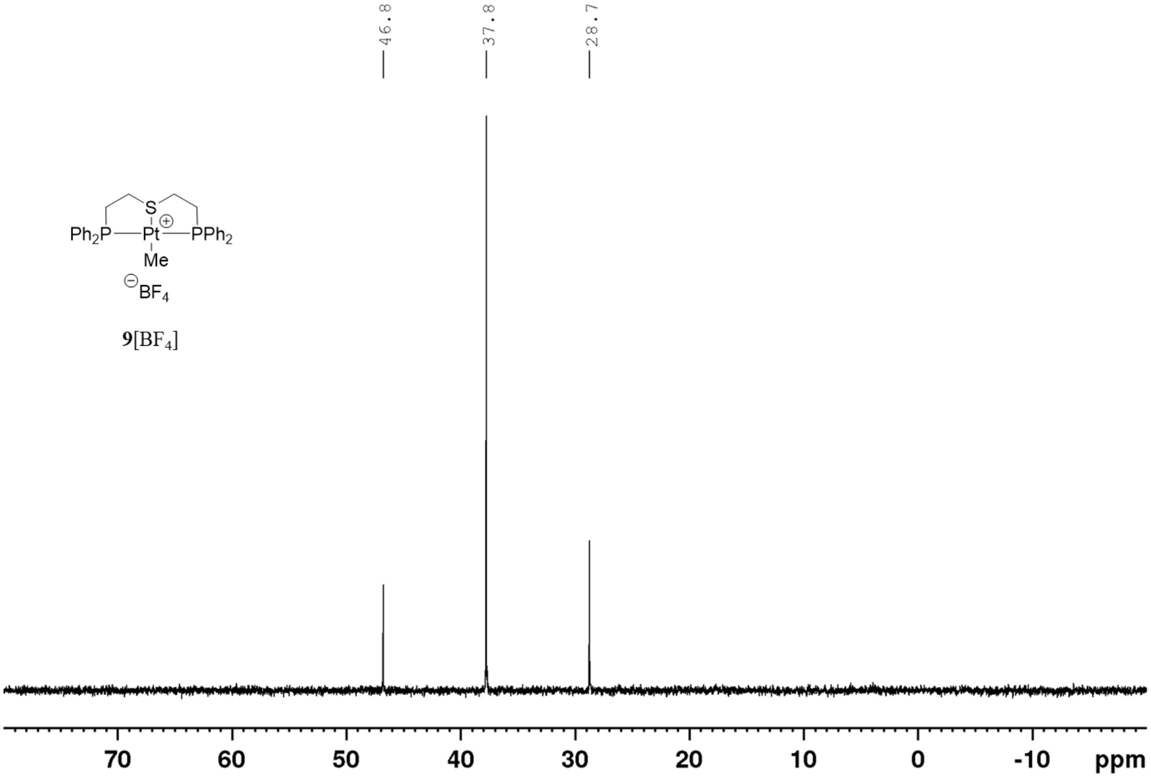


**Figure S83**: $^{31}$P{H} NMR (162 MHz) of **9**[BF$_4$] in $CD_2Cl_2$.

---

5 References:


[1] Persson, R.; Stchedroff, M. J.; Gobetto, R.; Carrano, C. J.; Richmond, M. G.; Monari, M.; Nordlander, E., *Eur. J. of Inorg. Chem.* **2013,** 13, 2447-59.

[2] Gulyas, H.; Benet-Buchholz, J.; Escudero-Adan, E. C.; Freixa, Z.; van Leeuwen, P. W., *Chem. Eur. J.* **2007,** *13*, 3424-30.

[3] Li, Y.; Nie, C.; Wang, H.; Li, X.; Verpoort, F.; Duan, C. *Eur. J. Org. Chem.* **2011**, *2011*, 7331.

[4] Bielawski, M.; Aili, D.; Olofsson, B. *J. Org. Chem.* **2008**, *73*, 4602– 4607.

[5] Siah, S.-Y.; Leung, P.-H.; Mok, K. F. Polyhedron **1994**, 23, 3253– 3255.

[6] Harris, R. K.; Becker, E. D.; Cabral De Menezes, S. M.; Granger, P.; Hoffman, R. E.; Zilm, K. W., *Solid State Nucl. Magn. Reson.* **2008,** *33*, 41-56.

[7] a) Erken, C.; Hindemith, C.; Weyhermüller, T.; Hölscher, M.; Werlé, C.; Leitner, W. *ACS Omega* **2020**, *5*, 8912–8918; b) De-Botton, S.; Romm, R.; Bensoussan, G.; Hitrik, M.; Musa, S.; Gelman, D. *Dalton Trans* **2016**, *45*, 16040– 16046

[8] Yanez, C. O.; Andrade, C. D.; Belfield, K. D. Chem. Commun. **2009**, 827– 829.

[9] Schirmer, M. L.; Jopp, S.; Holz, J.; Spannenberg, A.; Werner, T. *Adv. Synth. Catal.* **2016**, *358*, 26– 29

[10] GaussView, Version 6, Dennington, R.; Keith, T. A.; Millam, J. M. Semichem Inc., Shawnee Mission, KS, 2016.

[11] Gaussian 16, Revision C.01, Frisch, M. J.; Trucks, G. W.; Schlegel, H. B.; Scuseria, G. E.; Robb, M. A.; Cheeseman, J. R.; Scalmani, G.; Barone, V.; Petersson, G. A.; Nakatsuji, H.; Li, X.; Caricato, M.; Marenich, A. V.; Bloino, J.; Janesko, B. G.; Gomperts, R.; Mennucci, B.; Hratchian, H. P.; Ortiz, J. V.; Izmaylov, A. F.; Sonnenberg, J. L.; Williams-Young, D.; Ding, F.; Lipparini, F.; Egidi, F.; Goings, J.; Peng, B.; Petrone, A.; Henderson, T.; Ranasinghe, D.; Zakrzewski, V. G.; Gao, J.; Rega, N.; Zheng, G.; Liang, W.; Hada, M.; Ehara, M.; Toyota, K.; Fukuda, R.; Hasegawa, J.; Ishida, M.; Nakajima, T.; Honda, Y.; Kitao, O.; Nakai, H.; Vreven, T.; Throssell, K.; Montgomery, J. A., Jr.; Peralta, J. E.; Ogliaro, F.; Bearpark, M. J.; Heyd, J. J.; Brothers, E. N.; Kudin, K. N.; Staroverov, V. N.; Keith, T. A.; Kobayashi, R.; Normand, J.; Raghavachari, K.; Rendell, A. P.; Burant, J. C.; Iyengar, S. S.; Tomasi, J.; Cossi, M.; Millam, J. M.; Klene, M.; Adamo, C.; Cammi, R.; Ochterski, J. W.; Martin, R. L.; Morokuma, K.; Farkas, O.; Foresman, J. B.; Fox, D. J. Gaussian, Inc., Wallingford CT, 2016.

[12] Chai, J.-D.; Head-Gordon, M. *Phys. Chem. Chem. Phys.* **2008**, *10*, 6615

[13] Weigend, F.; Ahlrichs, R. *Phys. Chem. Chem. Phys.,* **2005**,*7*, 3297-3305

[14] a) Horn, P. R.; Head-Gordon, M. *J. Chem. Phys.* **2015**, *143*, 114111. b) Horn, P. R.; Mao, Y.; Head-Gordon, M. *J. Chem. Phys.* **2016**, *144*, 114107. c) Horn, P. R.; Mao, Y.; Head-Gordon, M. *Phys. Chem. Chem. Phys.* **2016**, *18*, 23067– 23079.

[15] Shao, Y.; Gan, Z.; Epifanovsky, E.; Gilbert, A. T. B.; Wormit, M.; Kussmann, J.; Lange, A. W.; Behn, A.; Deng, J.; Feng, X.;

Ghosh, D.; Goldey, M.; Horn, P. R.; Jacobson, L. D.; Kaliman, I.; Khaliullin, R. Z.; Landau, A.; Liu, J.; Proynov, E. I.; Rhee, Y. M.; Richard, R. M.; Rohrdanz, M. A.; Steele, R. P.; Sundstrom, E. J.; Woodcock, H. L.; Zimmerman, P. M.; Zuev, D.; Albrecht, B.; Alguire, E.; Austin, B.; Beran, G. J. O.; Bernard, Y. A.; Berquist, E.; Brandhorst, K.; Bravaya, K. B.; Brown, S. T.; Casanova, D.; Chang, C. M.; Chen, Y.; Chien, S. H.; Closser, K. D.; Crittenden, D. L.; Diedenhofen, M.; Distasio, R. A.; Do, H.; Dutoi, A. D.; Edgar, R. G.; Fatehi, S.; Fusti-Molnar, L.; Ghysels, A.; Golubeva-Zadorozhnaya, A.; Gomes, J.; Hanson-Heine, M. W. D.; Harbach, P. H. P.; Hauser, A. W.; Hohenstein, E. G.; Holden, Z. C.; Jagau, T. C.; Ji, H.; Kaduk, B.; Khistyaev, K.; Kim, J.; Kim, J.; King, R. A.; Klunzinger, P.; Kosenkov, D.; Kowalczyk, T.; Krauter, C. M.; Lao, K. U.; Laurent, A. D.; Lawler, K. V.; Levchenko, S. V.; Lin, C. Y.; Liu, F.; Livshits, E.; Lochan, R. C.; Luenser, A.; Manohar, P.; Manzer, S. F.; Mao, S. P.; Mardirossian, N.; Marenich, A. V.; Maurer, S. A.; Mayhall, N. J.; Neuscamman, E.; Oana, C. M.; Olivares-Amaya, R.; Oneill, D. P.; Parkhill, J. A.; Perrine, T. M.; Peverati, R.; Prociuk, A.; Rehn, D. R.; Rosta, E.; Russ, N. J.; Sharada, S. M.; Sharma, S.; Small, D. W.; Sodt, A.; Stein, T.; Stück, D.; Su, Y. C.; Thom, A. J. W.; Tsuchimochi, T.; Vanovschi, V.; Vogt, L.; Vydrov, O.; Wang, T.; Watson, M. A.; Wenzel, J.; White, A.; Williams, C. F.; Yang, J.; Yeganeh, S.; Yost, S. R.; You, Z. Q.; Zhang, I. Y.; Zhang, X.; Zhao, Y.; Brooks, B. R.; Chan, G. K. L.; Chipman, D. M.; Cramer, C. J.; Goddard, W. A.; Gordon, M. S.; Hehre, W. J.; Klamt, A.; Schaefer, H. F.; Schmidt, M. W.; Sherrill, C. D.; Truhlar, D. G.; Warshel, A.; Xu, X.; Aspuru-Guzik, A.; Baer, R.; Bell, A. T.; Besley, N. A.; Da Chai, J.; Dreuw, A.; Dunietz, B. D.; Furlani, T. R.; Gwaltney, S. R.; Hsu, C. P.; Jung, Y.; Kong, J.; Lambrecht, D. S.; Liang, W.; Ochsenfeld, C.; Rassolov, V. A.; Slipchenko, L. V.; Subotnik, J. E.; Van Voorhis, T.; Herbert, J. M.; Krylov, A. I.; Gill, P. M. W.; Head-Gordon, M. Advances in Molecular Quantum Chemistry Contained in the Q-Chem 4 Program Package. Mol. Phys. 2015, 113, 184– 215.

[16] Chai, J.-D.; Head-Gordon, M. *J. Chem. Phys.* **2008**, *128*, 084106.

[17] Gilbert, A. IQmol, http://iqmol.org/ (accessed on 21 Oct 2021)
